# Introducing Prism[4]arene: A Macrocycle with Enantiomerically Resolvable Inherent Chirality and Intriguing Chiroptical Properties

**DOI:** 10.1021/jacs.5c04512

**Published:** 2025-06-04

**Authors:** Paolo Della Sala, Carmen Talotta, Margherita De Rosa, Stefano Superchi, Ernesto Santoro, Silvano Geremia, Neal Hickey, Marco Fusè, Sergio Abbate, Giuseppe Mazzeo, Giovanna Longhi, Carmine Gaeta

**Affiliations:** † Laboratory of Supramolecular Chemistry, Dipartimento di Chimica e Biologia “A. Zambelli”, 19028 Università di Salerno, Via Giovanni Paolo II 132, 84084 Fisciano, Salerno, Italy; ‡ Dipartimento di Scienze di Base e Applicate, 19006 Università della Basilicata, Via dell’Ateneo Lucano 10, 85100 Potenza, Italy; § Centro di Eccellenza in Biocristallografia, Dipartimento di Scienze Chimiche e Farmaceutiche, 9315 Università di Trieste, Via L. Giorgieri 1, I-34127 Trieste, Italy; ∥ Dipartimento di Medicina Molecolare e Traslazionale, 9297 Università di Brescia, Viale Europa 11, 25123 Brescia, Italy; ⊥ Istituto Nazionale di Ottica (INO), CNR, Research Unit of Brescia, c/o CSMT, Via Branze 45, 25123 Brescia, Italy

## Abstract

This study presents the first report of an inherently chiral prismarene with resolved enantiomers. Prism[4]­arenes, synthesized via a thermodynamic template approach using a tailor-made selective cation, effectively maintain their chirality due to their strained macrorings and narrow annuli, which prevent the flipping of naphthalene rings. The solid-state structure of the synthesized **PrS­[4]** ^***iPe***^ revealed a racemic crystal composed of all-p*R* and all-p*S* enantiomeric pairs, forming supramolecular polymeric chains of homochiral molecules interlinked by intermolecular host–guest interactions. Both enantiomers were resolved by using chiral high-performance liquid chromatography (HPLC), and their chiroptical properties were thoroughly investigated. Configurational assignment was achieved through time-dependent density functional theory (TDDFT) computations alongside electronic circular dichroism/ultraviolet–visible (ECD/UV–vis) spectral analysis. Notably, the circularly polarized luminescence (CPL) properties exhibited a significant dissymmetry ratio of 0.008 for these prism[4]­arenes, due to electric and magnetic dipole transition moments both directed along the cylinder axis. Furthermore, the ability of **PrS­[4]** ^***iPe***^ to achieve enantioselective recognition with chiral ammonium guests was demonstrated.

## Graphical abstract

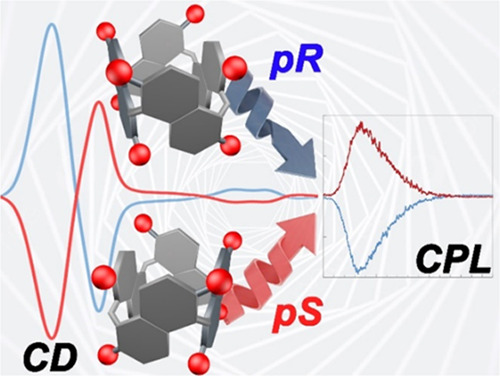

## Introduction

The design and synthesis of macrocycles have been a significant challenge in supramolecular chemistry for decades.^1,2^ Researchers have devoted considerable efforts to creating artificial systems that mimic natural molecules like proteins and enzymes.^1,2^ Among them, macrocyclic arenes with deep cavities,^3–8^ chiral structures, and peculiar (chiro)­optical properties have emerged as particularly attractive biomimetic hosts due to their potential applications across various fields.^1,2^

Additionally, from the perspective of emissive chiroptical spectroscopies, such as circularly polarized luminescence (CPL), there has been considerable interest in macrocycles with strong CPL responses,^9^ which is beneficial for sensing and electro-optical applications.^10–12^ Notably, examples have been reported by Sato^13^ and Fukunaga,^14^ who described macrocycles exhibiting chirality in their cylindrical molecular structures, resulting in extremely large dissymmetry factors associated with circularly polarized light. Prismarenes^7,8,15–25^ (Figure 1) represent a novel class of macrocycles constituted of methylene-bridged 2,6-dialkoxynaphthalene units, featuring distinctive deep cavities. Recently, there has been a surge of research focused on the synthesis and investigation of the supramolecular properties of these intriguing compounds.^15–25^ To date, the synthesis of prismarenes comprising five and six naphthalene units (**PrS­[5]** ^***R***^ and **PrS­[6]** ^***R***^ in Figure 1) has been reported.^7,8,15–25^

**Figure 1:**
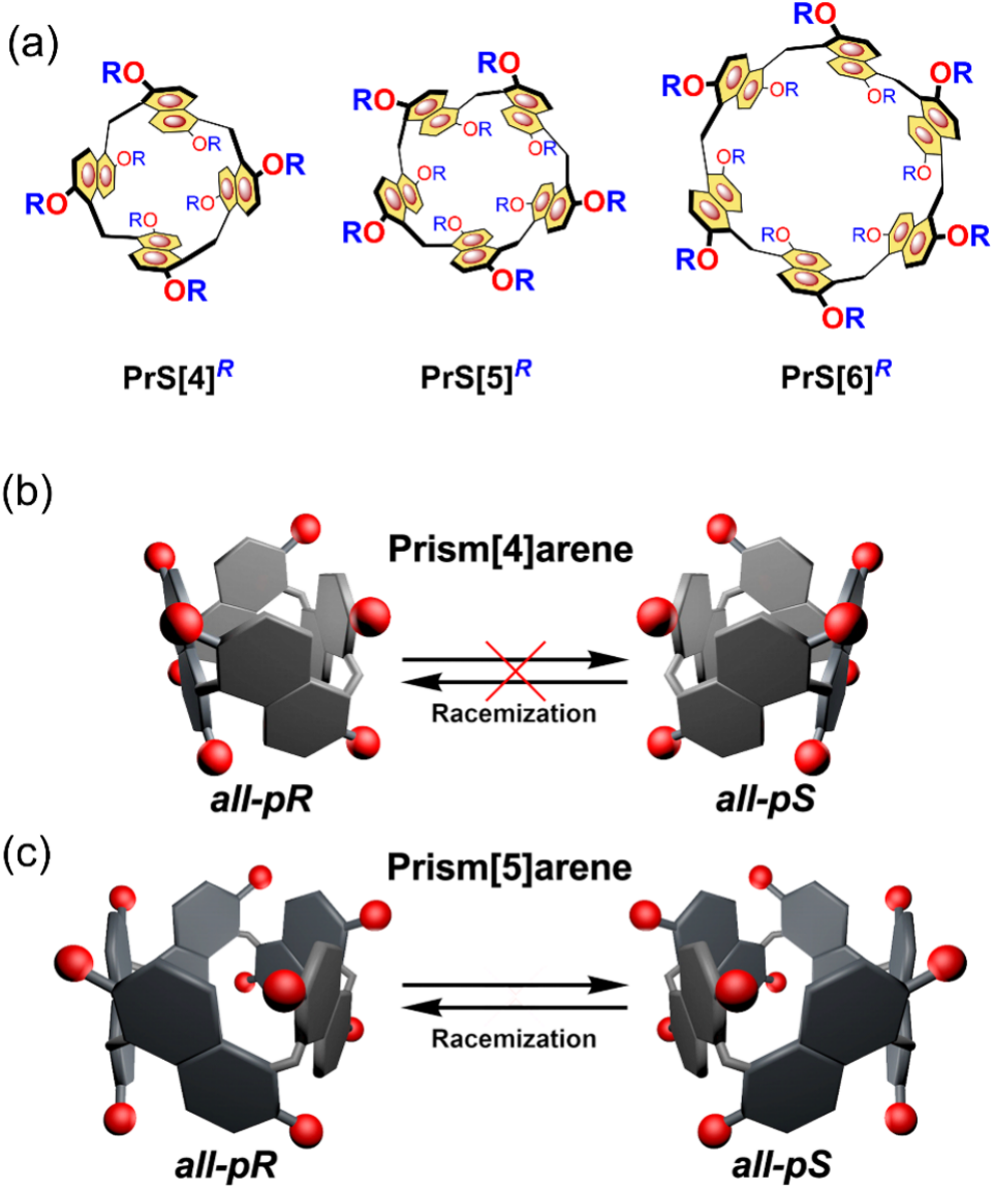
(a) Chemical drawings of the prism­[*n*]­arene family, highlighting various members. (b, c) Flipping-induced inversion of planar chirality (FIIPC) in prismarenes by oxygen-through-the-annulus passage. (b) FIIPC mechanism inhibited in prism[4]­arene (this work). (c) FIIPC active in prism[5]­arene.

Prismarenes display planar chirality^18^ of naphthalene moieties established by the curvature of the macroring (Figure 1).^18,25^ Each 2,6-dialkoxynaphthalene ring can adopt one of two configurations, referred to as p*R* and p*S* (Figure 1), with the most stable isomers being the homochiral all-p*R*/all-p*S*.^18^ These conformational isomers are capable of interconverting by oxygen-mediated concerted rotations of the naphthalene units through the annulus (Figure 1c).^16,18,25^

Notably, the all-p*S* and all-p*R* enantiomers of permethylated prismarenes (**PrS­[5]** ^***R***^ and **PrS­[6]** ^***R***^ in Figure 1) can interconvert via this mechanism, a process that occurs rapidly on the NMR time scale.^16,18,25^ However, the presence of long substituents or branched alkyl groups at both rims of prism[5]­arenes slows down inversion without completely hindering it.^16^ This is particularly evident considering that, while cyclohexylmethyl groups are sufficiently large to enable enantiomeric separation of pillar[5]­arene macrocycles^26^ without racemization, they still allow the passage of naphthalene units in prism[5]­arenes.^16^ These considerations clearly indicate that resolving prismarene enantiomers is challenging due to the large ring size of the prismacyclic structure.^8^

However, successfully blocking the planar chirality in these macrocycles would be highly beneficial, as it would pave the way for numerous applications, especially in materials chemistry and chiral sensing.^27–30^ Among macrocycles with stable chirality, Ogoshi and colleagues have recently demonstrated that 2-benzofuranyl groups are sufficiently large to induce stable chirality in pillar[5]­arene molecules.^30d^ An additional example of conformationally stable chiral macrocycles is provided by Chen, who reported the synthesis of octopus[3]­arenes.^30e^ Most recently, Cai and colleagues have developed a water-soluble macrocycle exhibiting chiral stability with effective enantioselective recognition properties.^5^ In 2020, Wang^9b^ and colleagues reported an intriguing example of inherently chiral tetraazacalix[4]­aromatics. These new macrocycles exhibited stable chirality, along with pH-triggered, switchable circular dichroism, and circularly polarized luminescence.

Finally, Chuan-Feng Chen previously reported the synthesis of anthracene-based planar chiral macrocycles, termed pagod[4]­arene,^6^ which are formed from four 2,6-dimethoxyanthracene units. These macrocycles exhibit stable planar chirality due to the narrow cavity that impedes the interconversion of the two enantiomers via the oxygen-through-the-annulus passage.

Therefore, to achieve the goal of impeding the racemization of prismarenes, we restricted the size of the cavity by reducing the number of monomers in the macrocycle. In this work, we achieved the synthesis of the first racemic prismarene, which can be resolved into its individual enantiomers, allowing investigation of enantiospecific recognition and chiroptical properties of this class of chiral macrorings.

In detail, we demonstrated that a thermodynamically templated macrocyclization,^7,17,31^ facilitated by a tailor-made templating agent,^17^ allows the isolation of the first example of prism[4]­arene from the reaction mixture.

The racemic **PrS­[4]** ^***iPe***^ was characterized by NMR and single-crystal X-ray diffraction. The two enantiomers of prism[4]­arene were separated using chiral high-performance liquid chromatography (HPLC), and their chiroptical properties, i.e., electronic circular dichroism (ECD) and circular polarized luminescence (CPL), were investigated.

Density functional theory (DFT) calculations of ECD and CPL enabled us to determine the absolute configurations and conformational properties of the macrocycle in both the ground and excited states. Finally, we investigated the chiral recognition capabilities of this novel macrocycle.

## Result and Discussion

### Templated Synthesis of Prism[4]­arenes

To date, the tetramer prism[4]­arene has not been observed during the macrocyclization process involving 2,6-dialkoxynaphthalene units. Our previous research demonstrated that the main factors which direct the product distribution of prism­[*n*]­arene cyclooligomers are the solvent, the alkoxy groups, and the presence of templates.^7,8,16,17^ For example, prism[5]­arene are preferentially formed in halogenated solvents, such as 1,2-dichloroethane (DCE), particularly when templating agents like DABCO cations are utilized.^7,17^ The formation of prism[6]­arene is significantly enhanced, reaching 75–90%, when cyclohexane is used as the solvent and the alkyl chains are of suitable size and length to facilitate effective self-filling of the hexamer’s cavity (typically ethyl and propyl).^8^ In our systematic study on the investigation of the effects of these factors, when 2,6-bis­(isopentyloxy)­naphthalene **1a** underwent macrocyclization with paraformaldehyde, trifluoroacetic acid (TFA), in cyclohexane solution (Scheme 1), the desired **PrS­[4]** ^*iPe*^ was obtained with a yield of 5% (Table 1). Additionally, chromatographic purification and spectrometric analysis identified linear oligomers as well as a complicated mixture of other prismarene cyclooligomers.

**Scheme 1:**
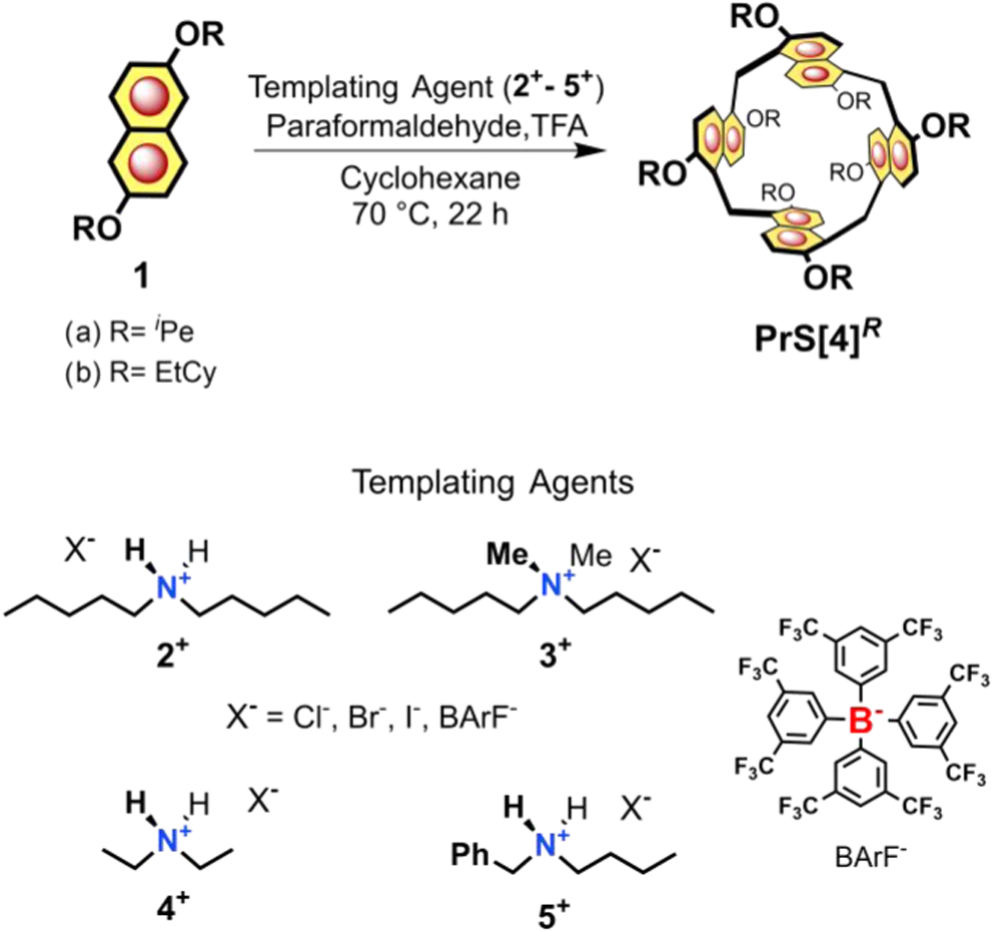
Tailor-Made Template Synthesis of Prism[4]­arenes

**Table 1:** Synthesis of **PrS­[4]** ^***R***^ (R = *i*Pe and EtCy) from Starting Monomers (**1a,b** in Scheme 1) and Templating Agents (**2**–**5**)^+^ as Chloride Salts, along with Binding Constant Values (*K*_ass_, M^–1^) Determined by ^1^H NMR Experiments (See the SI for Details; Mean Values of Three Measurements, Estimated Errors <15%,)

templating agent (**G** ^**+**^)	R	**PrS[4]**^*R*^ (yield %)	***K***_ass_**G**^+^@ **PrS[4]**^*R*^
–	*i*Pe	5	
**2** ^**+**^ ^c^	*i*Pe	20	45,000^b^
**3** ^**+**^	*i*Pe	5	125^a^
**4** ^**+**^	*i*Pe	4	4200^b^
**5** ^**+**^	*i*Pe	4	5600^b^
**2** ^**+**^	EtCy	15	1000^b^

a Calculated by integrating the free and complexed ^1^H NMR signals of the host.

b Calculated using a ^1^H NMR competition experiment with methoxy-prism[5]­arene as a competitive host.

c Guest **2** ^**+**^ was tested also with I^–^ and Br^–^ as counterions, but no significant differences in yield were observed.

The high-resolution Fourier transform ion cyclotron resonance (FT-ICR) mass spectrum confirmed the molecular mass of **PrS­[4]** ^*iPe*^, with an observed molecular ion peak at *m*/*z* of 1248.8354, matching the calculated value of 1248.8352 for [M]^+^. Detailed one-dimensional (1D) and two-dimensional (2D) NMR analyses (SI) demonstrated that all naphthalene rings of **PrS­[4]** ^*iPe*^ are interconnected at their 1,5-positions, exhibiting *D*_4_ symmetry. This structural arrangement was further confirmed by X-ray crystallographic analysis (vide infra). The ^1^H NMR spectrum (Figure 2e, 600 MHz, 298 K, CD_2_Cl_2_) of **PrS­[4]** ^*iPe*^ displayed an aromatic AX system at 8.19 and 6.89 ppm (*J* = 9.0 Hz), a singlet attributable to the methylene bridges at 4.68 ppm, and an AB system at 4.20 and 4.10 ppm associated with diastereotopic OCH_2_ groups (Figure 2e). Moreover, ultraviolet–visible (UV–vis) and emission spectra were recorded (SI, dichloromethane, 25 °C). Absorption spectrum shows three bands at 237, 286, and 352 nm and exhibits a fluorescence maximum at λ_em_= 385 nm (fluorescence quantum yield ϕ = 0.23). With these results in hand, the objective was to identify an effective templating guest that could enhance the yield of prism[4]­arenes during the macrocyclization process.^7,17^ Given the smaller cavity of prism[4]­arene compared to its pentameric and hexameric counterparts (see Figure 3 and the Solid-State Studies section), the study focused on the complexation abilities toward axle-type dialkylammonium and tetraalkylammonium ions **2** ^**+**^–**5** ^**+**^ (Scheme 1).

**Figure 2:**
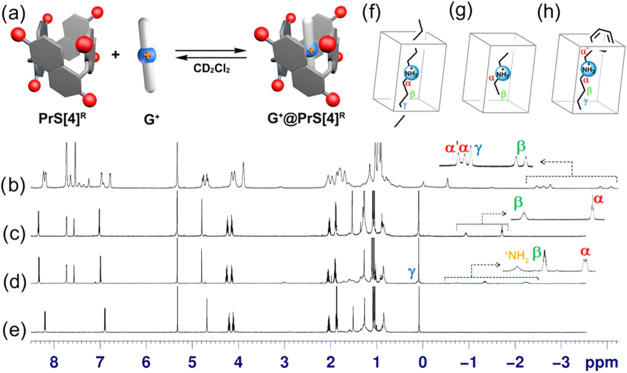
(a) Schematic illustration of the complexation between **PrS­[4]** ^***R***^ and ammonium guests; (b–e) ^1^H NMR spectra (CD_2_Cl_2_, 600 MHz) of: (b) a 1:1 mixture of **PrS­[4]** ^***iPe***^ and **5** ^**+**^ at 233 K (4.56 mM), with assignments delineated in (h); (c) a 1:1 mixture of **PrS­[4]** ^***iPe***^ and **4** ^**+**^ at 298 K (4.00 mM), with assignments shown in (g); (d) a 1:1 mixture of **PrS­[4]** ^***iPe***^ and **2** ^**+**^ at 298 K (2.67 mM), with assignments depicted in (f); (e) **PrS­[4]** ^***iPe***^ at 298 K. (f–h) Cartoon representations of the *endo*-cavity complexes **G** ^**+**^@**PrS­[4]** ^***iPe***^.

**Figure 3:**
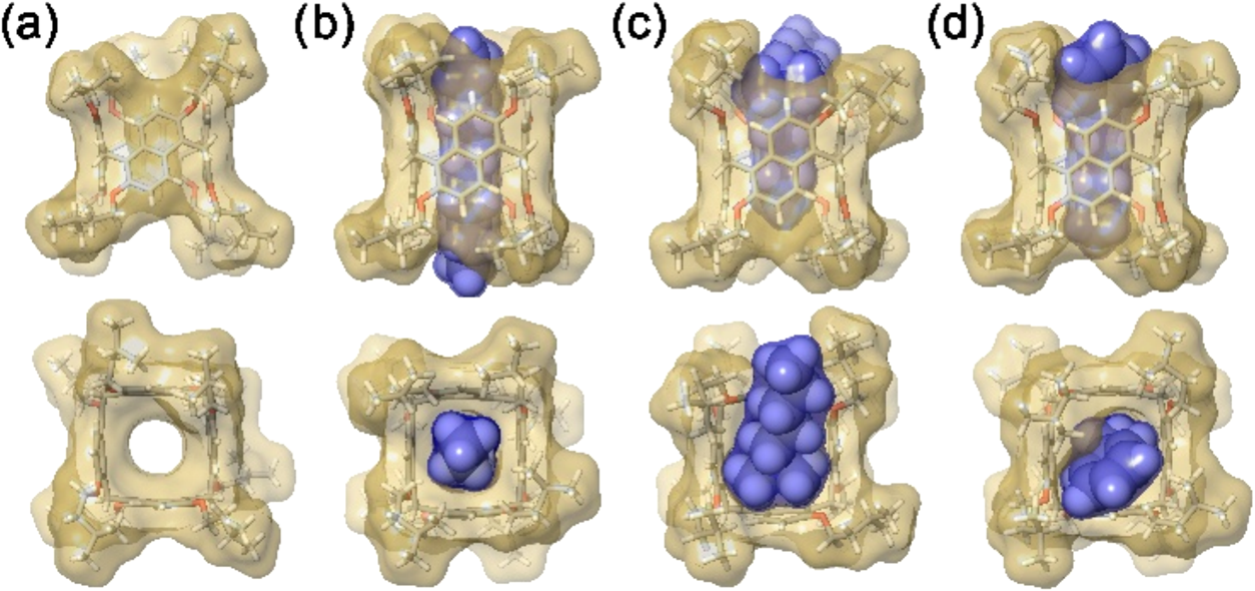
Representation with molecular surface of DFT-optimized structures at B97D3/SVP/SVPFIT level of theory of: (a) **PrS­[4]** ^***iPe***^, (b) **2** ^**+**^ **@PrS­[4]** ^***iPe***^, (c) **3** ^**+**^ **@PrS­[4]** ^***iPe***^, and (d) **5** ^**+**^ **@PrS­[4]** ^***iPe***^ complexes in side view (top) and top view (bottom).

In accordance with a standardized protocol,^7,17^ we conducted ^1^H NMR spectroscopic investigations to evaluate the binding affinity of **PrS­[4]** ^*iPe*^ with the selected cations **2** ^**+**^–**5** ^**+**^ as barfate salts (BArF^–^ in Scheme 1).

When **2** ^**+**^ as a barfate salt was mixed in equimolar ratios with **PrS­[4]** ^*iPe*^ in a CD_2_Cl_2_ solution, the ^1^H NMR spectra of both the host and guest (Figure 2d) exhibited significant changes, indicating the formation of the *endo*-cavity complex **2** ^**+**^@**PrS­[4]** ^*iPe*^. The ^1^H NMR spectrum of **2** ^**+**^ **@PrS­[4]** ^*iPe*^ (Figure 2d) exhibits two aromatic signals at 8.32 and 6.99 ppm (Δδ = 1.33 ppm), and diastereotopic hydrogens of OC*H*_2_ groups were found at 4.24 and 4.13 ppm. The rod-like **2** ^**+**^ cation is threaded through the central cavity of **PrS­[4]** ^*iPe*^, forming a pseudo[2]­rotaxane architecture (Figure 3b). This structure was confirmed by 1D and 2D NMR analyses (SI). In particular, the ^1^H NMR spectrum (Figure 2d) shows signals at negative chemical shifts, which can be assigned to guest **2** ^**+**^. Specifically, broad signals at −0.74, −1.35, and −2.25 ppm are attributed to the ^+^NH_2_ group and the CH_2_ groups in the β and α positions, respectively. In the HSQC spectrum, the β and α signals at −1.35 and −2.25 ppm correlate with carbon signals at 26.3 and 44.0 ppm (SI).

The complexation process between **2** ^**+**^ and **PrS­[4]** ^*iPe*^ occurs slowly on the NMR time scale. The association constant (*K*_ass_) for the formation of the **2** ^**+**^ **@ PrS[4]** ^*iPe*^ complex was determined through an NMR competition experiment against the previously characterized **PrS­[5]** ^*Me*^,^7^ yielding a value of 4.5 × 10^4^ M^–1^ (Table 1 and SI).

Comparable spectral features were found in the ^1^H NMR spectrum of the **3** ^**+**^ **@PrS­[4]** ^*iPe*^ complex (SI). The signals of guest ion **3** ^**+**^, threaded within the macrocyclic cavity, experienced shielding effects, resulting in negative chemical shift values. Specifically, the ^+^NMe signal was observed at 2.96 ppm, while the α–CH_2_ signal appeared at −2.24 ppm, as confirmed by COSY and HSQC experiments (SI). A binding constant of 125 M^–1^ was calculated for the formation of the **3** ^**+**^ **@PrS­[4]** ^*iPe*^ complex (Table 1), which is significantly lower than that for the **2** ^**+**^ **@PrS­[4]** ^*iPe*^ complex.

This indicates that the interaction between the **3** ^**+**^ cation and the **PrS­[4]** ^*iPe*^ host is weaker than that of the **2** ^**+**^ cation. This reduced binding affinity may stem from the steric hindrance introduced by the N­(Me)_2_ group (Figure 3c) within the cavity of prismarene, which can limit the accessibility and optimal fitting of the **3** ^**+**^ cation within the host structure.

Notably, **PrS­[4]** ^*iPe*^ exhibits no conformational changes upon *endo*-cavity complexation with guests **2** ^+^–**5** ^+^. In contrast, larger prism[5]­arene typically undergoes conformational adaptation during complexation with cationic guests, as evidenced by ^1^H NMR studies.^7,8^ For example, the spacing Δδ between the aromatic doublets shifts from approximately 1.2 ppm in the free prism[5]­arene **PrS­[5]** ^*R*^ to 1.6 ppm when complexed **G** ^+^@**PrS­[5]** ^*R*^, indicating an induced fit process to accommodate the guest.^7,8^ Comparatively, the Δδ of the aromatic protons of the complexed **PrS­[4]** ^*iPe*^ in the **G** ^**+**^@**PrS­[4]** ^*iPe*^ complex remains identical to that of the free macrocycle **PrS­[4]** ^*iPe*^ (about 1.3 ppm). This result suggests that the rigid structure of **PrS­[4]** ^*iPe*^ does not experience significant conformational changes upon guest threading (see the comparison between panels (a) and (b) of Figure 3, top view). These structural features invoke Cram’s preorganization principle,^32^ indicating that the internal cavity of the prism[4]­arene host exhibits a high degree of preorganization, thereby facilitating the complexation of suitable guest molecules.

Upon addition of one equivalent of the diethylammonium guest **4** ^**+**^ as a barfate salt to a CD_2_Cl_2_ solution of **PrS­[4]** ^*iPe*^, the ^1^H NMR spectrum (Figure 2c) of the resulting mixture exclusively displayed signals corresponding to the **4** ^**+**^ **@ PrS[4]** ^*iPe*^ complex. Signals were observed at −1.73 and −0.94 ppm, corresponding to the CH_3_ and CH_2_ protons of the guest, respectively. Additionally, a signal at −0.13 ppm was observed for the ^+^NH_2_ group. A binding constant of 4200 M^–1^ was calculated for the complexation of **4** ^**+**^, which is significantly higher than that observed for the complexation of **3** ^**+**^. This result confirms that prism[4]­arene prefers rod-shaped linear guests.

The ^1^H NMR spectrum in Figure 2b highlights the complexation of butylbenzylammonium **5** ^**+**^, resulting in the formation of **5** ^**+**^@**PrS­[4]** ^*iPe*^. Specifically, confirmed by the DFT-optimized structure of the **5** ^**+**^@**PrS­[4]** ^*iPe*^ complex, illustrated in Figure 3d, the butyl chain is deeply embedded within the cavity of **PrS­[4]** ^*iPe*^. Meanwhile, the bulky benzyl moiety is located externally, in contact with the rim, facilitating C–H···π interactions with the isopentyl groups. The interlocking of the benzyl group among the isopentyl chains likely restricts the rotational freedom of guest **5** ^**+**^ within the cavity of **PrS­[4]** ^*iPe*^, which may explain the presence of diastereotopic signals for the β–H of **5** ^**+**^, as depicted in Figures 2b and 3d. A binding constant of 5600 M^–1^ was calculated for the formation of **5** ^**+**^@**PrS­[4]** ^*iPe*^, which is comparable to that of **4** ^**+**^@**PrS­[4]** ^*iPe*^ (Table 1).

Natural Bond Orbital (NBO) and Noncovalent Interaction (NCI) calculations^7,8^ were conducted on the **2** ^**+**^–**4** ^+^ complexes utilizing the B97D3/SVP/SVPFIT level of theory (see the SI). The dipentylammonium cation **2** ^**+**^ establishes CH···π and ^+^N–H···π interactions with **PrS­[4]** ^*iPe*^, contributing 72 and 14% to the total interaction energy, respectively. According to the DFT-optimized structures presented in Figure 3, calculations indicate that **2** ^**+**^ occupies 92% of the internal volume upon complexation. In comparison, the volumes occupied by the **3** ^**+**^, **4** ^**+**^, and **5** ^**+**^ cations are 60, 52, and 59%, respectively. The NBO analysis of the **3** ^**+**^@**PrS­[4]** ^***iPe***^ complex reveals a similar proportion of CH···π interactions; however, the cation’s ^+^N···π interactions account for only 1%. The presence of the N–Me group in **3** ^**+**^ hinders the optimal fitting of the axis within the cavity, thereby limiting the access of the positively charged nitrogen.

Based on the data presented in Table 1, the dipentylammonium cation **2** ^**+**^ appears to be the best candidate as a template for the directed synthesis of **PrS­[4]** ^***iPe***^.

In cyclohexane as solvent, 2,6-bis­(isopentyloxy)­naphthalene **1a** (5.0 mM) was reacted with paraformaldehyde and trifluoroacetic acid (TFA) in the presence of 1 equiv of the dipentylammonium **2** ^**+**^ as chloride salt. The reaction progress was monitored by thin-layer chromatography (TLC). After 22 h, the reaction was quenched by adding a saturated aqueous solution of sodium bicarbonate (NaHCO_3_). Following standard workup procedures and chromatographic purification, **PrS­[4]** ^*iPe*^ was obtained with a yield of 20%, along with a mixture of linear oligomers, and a complex mixture of other prismarene compounds. The macrocyclization in Scheme 1 was also conducted in the presence of **2** ^**+**^ as I^–^ or Br^–^ salts (Table 1). No significant differences in yields were observed with the iodide or bromide salt of **2** ^**+**^ (Table 1). To confirm the hypothesized relationship between the affinity of **2** ^**+**^–**5** ^**+**^ for **PrS­[4]** ^*iPe*^—quantified as the binding constant values for their prismarene complexes (Table 1)—and their efficiency as templates, which is assessed through macrocyclization yield, we also conducted reactions using the cations **3** ^**+**^–**5** ^**+**^ (see Table 1).

Specifically, when 2,6-bis­(isopentyloxy)­naphthalene **1a** was subjected to macrocyclization under the conditions outlined in Scheme 1, the isolation of **PrS­[4]** ^*iPe*^ in the presence of template **3** ^**+**^ yielded only 5% (Table 1). This yield is significantly lower than that achieved with template **2** ^**+**^ and is comparable to the yield observed when the reaction was conducted without any template. Similarly, low yields of **PrS­[4]** ^*iPe*^ were observed in the presence of the templating agents **4** ^**+**^ and **5** ^**+**^ (Table 1), which exhibit a lower affinity for the prismarene **PrS­[4]** ^*iPe*^ compared to **2** ^**+**^. Therefore, only guest **2** ^**+**^ shows a significant template effect, while all the other guests, having a binding affinity at least 1 order of magnitude lower, show no template effect in the synthesis (Table 1). These findings indicate that in the thermodynamically driven macrocyclization of prismarenes, the design of the templating agent should be based on its binding affinity for the macrocycle. Finally, the cation **2** ^**+**^ was effective as templating agent even in the macrocyclization of 2,6-bis­(2-cyclohexylethoxy)­naphthalene **1b**. When **1b** was reacted with paraformaldehyde in the presence of TFA in cyclohexane and **2** ^**+**^·Cl^–^ as templating agent the reaction afforded **PrS­[4]** ^*EtCy*^ with a yield of 15% (Table 1).

### Solid-State Studies

Single crystals suitable for X-ray diffraction (XRD) analysis were obtained through the slow evaporation of a methanol/dichloromethane solution containing **PrS­[4]** ^*iPe*^.

While the prismarene molecules lie on *C*_2_ crystallographic symmetry axes which pass through opposite methylene bridges, the prism[4]­arene skeletons show *pseudo D*_4_ point symmetry (Figure 4a,b). Therefore, all of the naphthalene units of each molecule have the same planar chirality. The centrosymmetric crystal is composed of a racemic mixture of all-p*R* and all-p*S* enantiomeric pairs (Figure 4e). The *pseudo D*_4_ point symmetry of the scaffold is stabilized by eight weak intramolecular C–H···O hydrogen bonds (C···O distances ranging from 3.05 to 3.22 Å, SI), involving the hydrogen atoms of the equivalent 4 and 8 naphthalene positions pointing toward the oxygen lone pair of the neighboring alkoxy groups.

**Figure 4:**
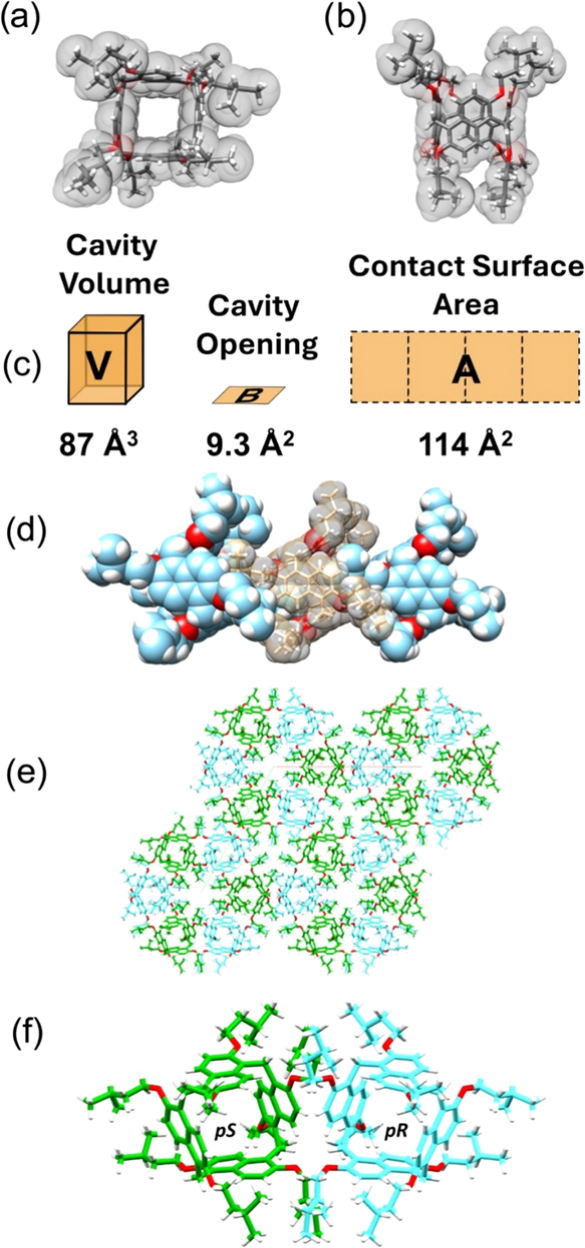
Top view (a) and side view (b) of the solid-state structure of **PrS­[4]** ^*iPe*^. The molecule is shown as a capped stick representation inside its van der Waals surface. (c) Geometric characteristics of the internal cavity of **PrS­[4]** ^*iPe*^: void volume (V), opening (B), and contact surface area (A). (d) Linear homochiral polymeric assembly of **PrS­[4]** ^*iPe*^, formed by the mutual threading of isopentyl chains. Each cavity hosts two isopentyl threads from adjacent prismarenes. The central molecule (brown) is shown as a capped stick representation inside its transparent van der Waals surface, to aid visualization of the threaded isopentyl chains of the adjacent molecules (cyan). (e) Crystal packing of **PrS­[4]** ^*iPe*^ enantiomeric pairs: all-p*R* (cyan) and all-p*S* (green), as viewed along the 3̅-axes. (f) Detail of one enantiomeric pair.

The crystal packing shows the formation of supramolecular polymeric chains composed of homochiral **PrS­[4]** ^*iPe*^ molecules (Figure 4d). In particular, the **PrS­[4]** ^*iPe*^ macrocycles form a linear homopolymeric assembly along the *c*-axis by mutual threading of isopentyl chains inside the cavity of adjacent prismarenes, related by translation of the unit cell and therefore with the same planar chirality (Figure 4d). The hexagonal arrangement of these linear homochiral polymeric assemblies along the rotoinversion 3̅-axes is characterized by the alternate disposition of all-p*R* and all-p*S* chains (Figure 4e). The prism[4]­arene scaffold shows only a small deviation from a regular square prism, as indicated by the dihedral angles between the mean planes of the naphthalene rings.

These angles are 93 and 96° for the naphthalene moieties related by symmetry (SI), while the two angles between the independent naphthalene moieties are 86°. This deformation, with two opposite dihedral angles being obtuse and two being acute, is apparent in the two diagonal distances between the opposite methylene bridges, which are 9.24 and 8.86 Å (SI).

All naphthalene planes are slightly bent (with a dihedral angle of 9–10° between the mean planes of the two fused aromatic rings) outward from the cavity, forming a saddle-like deformation. The distances between the adjacent methylene bridges, which represent the base of the prism, are 6.36 and 6.44 Å (SI).

The surface area, *A*, and volume, *V*, of the regular square prism enclosed by the aromatic walls of **PrS­[4]** ^*iPe*^ have been evaluated based on the geometrical method reported in a previous paper (Figure 4c and SI).^8^ In particular, the volume *V* of the regular square prism enclosed by the macrocycle was calculated as the product of the area of the square base *B*, which represents the cavity opening, and the geometric height (SI). In addition, the potential contact surface area, *A*, was calculated as the total internal area of the four rectangular prism faces (Figure 4c). The calculated internal volume of 87 Å^3^ for the prism[4]­arene scaffold is approximately 1/3 of the volume enclosed by prism[5]­arene (255 Å^3^) and less than 1/5 of that of prism[6]­arene (490 Å^3^).^8^ This volume is also less than half of the enclosed volume of the analogous pagoda[4]­arene^6^ based on 2,6-dialkoxylanthracene (206 Å^3^). The cavity opening, *B*, is strictly related to the number of monomers in the macrocycle. Thus, prism[4]­arene shows a narrower opening (9.3 Å^2^) than prism[5]­arene and prism[6]­arene (27.3 and 52.4 Å^2^, respectively).^8^ Interestingly, the comparison with the analogous anthracene tetramer, pagoda[4]­arene,^6^ shows that the smaller enclosed volume of the naphthalene derivative, prism[4]­arene is mainly due to the smaller opening of the cavity (19.1 Å^2^ in pagoda[4]­arene), while the depth of the cavity is similar (9.35 and 10.78 Å in prismarene and pagodarene, respectively). Another important geometric feature is the potential contact surface area A, derived from the total area of the rectangular prism faces. In this case, the prism[4]­arene also exhibits a smaller potential contact area (114 Å^2^) than the other macrocycles evaluated: prism[5]­arene (186 Å^2^), pagoda[4]­arene (188 Å^2^), and prism[6]­arene (252 Å^2^).^8^

### Chiral Resolution, Determination of Absolute Configuration, and Chiroptical Properties of Prism[4]­arene

Starting from the racemic mixtures of **PrS­[4]** ^*iPe*^ and **PrS­[4]** ^*EtCy*^, we proceeded to separate their enantiomers using HPLC on a cellulose phenyl carbamate (OD) chiral stationary phase (SI and Figure 5). When *rac*-**PrS­[4]** ^*iPe*^ was separated onto the chiral column, the chromatogram (Figure S75) revealed two distinct peaks of equal area, confirming that *rac*- **PrS­[4]** ^*iPe*^ consisted of an equimolar mixture of all-p*S*-**PrS­[4]** ^*iPe*^ and all-p*R*-**PrS­[4]** ^*iPe*^ (Figure 5). We isolated both fractions and measured their optical rotations in dichloromethane. The first eluted fraction exhibited a specific rotation of [α]_D_ = −14.9° (*c* = 1.1 mg·mL^–1^), while the second showed [α]_D_ = +15.0° (*c* = 1.1 mg·mL^–1^). Furthermore, ECD analysis of these isolated fractions produced mirror-image spectra (Figure 5c), providing additional evidence that the resolution of the enantiomers was successfully achieved, and no subsequent racemization occurred. The racemization of (−)-**PrS­[4]** ^*iPe*^ and (+)-**PrS­[4]** ^*iPe*^ was not observed even when their solution in cyclohexane was heated to 70 °C for 48 h (Figure S75). Analogously, the resolution of *rac*-**PrS­[4]** ^*EtCy*^ was efficiently achieved by chiral HPLC (Figure S76). Both fractions of *rac*-**PrS­[4]** ^*EtCy*^ were separated and collected.

**Figure 5:**
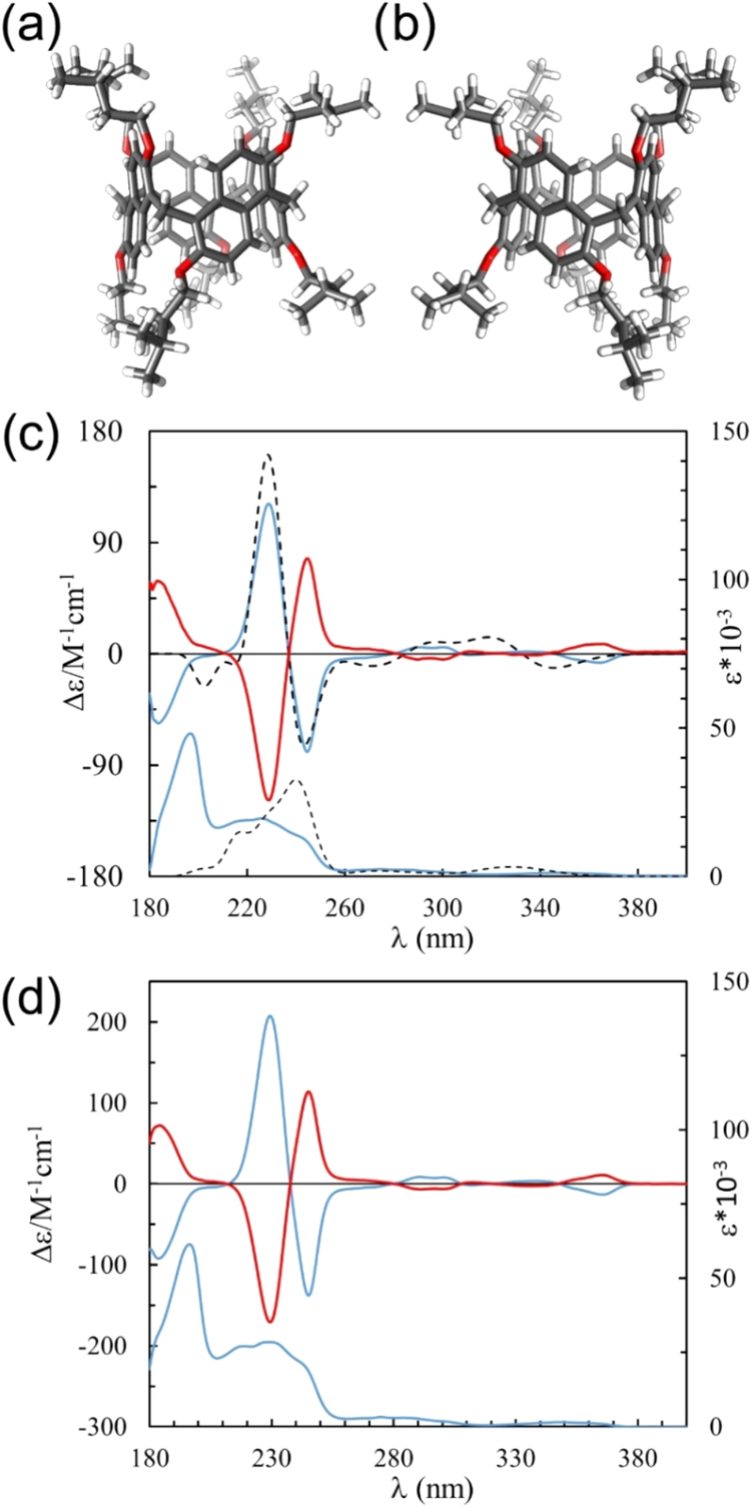
Representation of the X-ray molecular structures of (a) all-p*S*-**PrS­[4]** ^*iPe*^ and (b) all-p*R*-**PrS­[4]** ^*iPe*^. (c) Experimental ECD (top trace) and UV (bottom trace) spectra of (−)-**PrS­[4]** ^*iPe*^ (blue, first eluted) and (+)-**PrS­[4]** ^*iPe*^ (red, second eluted) and TDDFT/CAM-B3LYP/6–311G­(d,p) computed ECD (top trace) and UV (bottom trace) spectra for all-p*S*-**PrS­[4]** ^*iPe*^ (dashed Black). (d) Experimental ECD (top trace) and UV (bottom trace) (−)-**PrS­[4]** ^*EtCy*^ (blue, first eluted) and (+)-**PrS­[4]** ^*EtCy*^ (red, second eluted).

The first eluted showed a specific rotation of [α]_D_ = −4.1° (*c* = 2.0 mg·mL^–1^), while the second eluted showed [α]_D_ = +4.0° (*c* = 2.0 mg·mL^–1^). ECD analysis of these isolated fractions also produced mirror-image spectra (Figure 5d). The ECD spectra recorded in the 180–400 nm wavelength range show several Cotton effects (CEs) that are very similar for both **PrS­[4]** ^*iPe*^ and **PrS­[4]** ^*EtCy*^ compounds (Figure 5c,d). For (−)-**PrS­[4]** ^*iPe*^, at high wavelength, a negative band is visible at 364 nm followed by a weaker positive one at 335 nm, a weak negative one at 311 nm, and a positive band at 296 nm.

However, the main features of the spectrum are at lower wavelengths, with the two very strong CEs at 245 nm (negative) and 229 nm (positive), followed by the negative one at 184 nm. As expected, the ECD of the second eluted (+)-**PrS­[4]** ^*iPe*^ enantiomer is a mirror image of the (−)-**PrS­[4]** ^*iPe*^ spectrum.

The enantiomers of **PrS­[4]** ^***EtCy***^ exhibit ECD spectra that are nearly superimposable with the corresponding enantiomers of **PrS­[4]** ^*iPe*^ with the same [α]_D_ sign. As we have previously demonstrated for **PrS­[5]** ^***R***^ and **PrS­[6]** ^***R***^ prismarenes,^18^ the main observed bands can be ascribed to three distinct couplet features generated by exciton coupling^33^ between the long- and short-axis polarized transitions of naphthalene chromophores centered at 345, 269, and 228 nm, respectively (Figures S88 and S89).^34^

The CE at higher energy is associated with the π–π* transitions of HOMO −2, −5, −6 to LUMO, +2, +4, and +5 of the naphthalene chromophores (Figure S90, Tables S4 and S5). Accordingly, application of the Harada-Nakanishi exciton chirality rule^33^ to the higher-energy long-axis polarized transition of the naphthalene chromophore at 228 nm can allow absolute configuration assignment to the macrocycle enantiomers (SI). In fact, we have previously shown^18^ that a negative couplet (negative at lower-energy and positive at higher-energy Cotton effect) can be associated with the all-p*S* enantiomer and a positive couplet with the all-p*R* enantiomer (Figures S88 and S89). Therefore, the first eluted enantiomers (−)-**PrS­[4]** ^*iPe*^ and (−)-**PrS­[4]** ^*EtCy*^, both displaying a negative couplet at around 230 nm, are expected to have an all-p*S* absolute configuration. This preliminary configurational assignment was further supported by TDDFT computations of [α]_D_ and ECD/UV–vis spectra^35^ on p*S*-**PrS­[4]** ^*iPe*^ (SI). The starting geometry for TDDFT calculations on **PrS­[4]** ^*iPe*^ was retained by coordinates of the crystal structure and optimized at the DFT/B3LYP/6–311G­(d,p)/gas-phase level of theory. A computed [α]_D_ value of −23.9° was obtained for all-p*S*-**PrS­[4]** ^*iPe*^ at the DFT/B3LYP/TZVP level of theory, in agreement with [α]_D_ of the levorotatory first eluted enantiomer. The TDDFT/CAM-B3LYP/6–311G­(d,p)/gas^36^ phase computed ECD spectrum for all-p*S*-**PrS­[4]** ^*iPe*^ (Figure 5c, black dashed trace), was also in good agreement with the experimental, particularly in reproducing the most intense negative couplet at 237 nm, and thus confirming the all-p*S*-**PrS­[4]** ^*iPe*^ absolute configuration for the first eluted (−)-**PrS­[4]** ^*iPe*^ enantiomer. The less intense negative couplet at 346 nm is also well reproduced in terms of negative CE intensities but not so well in terms of wavelength position, mostly due to intrinsic limitations of the CAM-B3LYP functional.^36^ The structural and spectral similarity between the two resolved prismarenes can also allow the assignment of the absolute configuration for the **PrS­[4]** ^*EtCy*^ enantiomers.

### Circularly Polarized Luminescence of Prism[4]­arenes

CPL^37^ spectra in Figure 6 have been recorded for both eluted enantiomers of **PrS­[4]** ^*EtCy*^ and **PrS­[4]** ^*iPe*^ in 4.5 × 10^–4^ M hexane solutions in 2 mm cuvette, with a homemade apparatus using an excitation wavelength of 345 nm. Spectra in Figure 6 are reported after normalization of the corresponding fluorescence band.^38^ The two compounds show quite similar CPL features with practically the same intensity.

**Figure 6:**
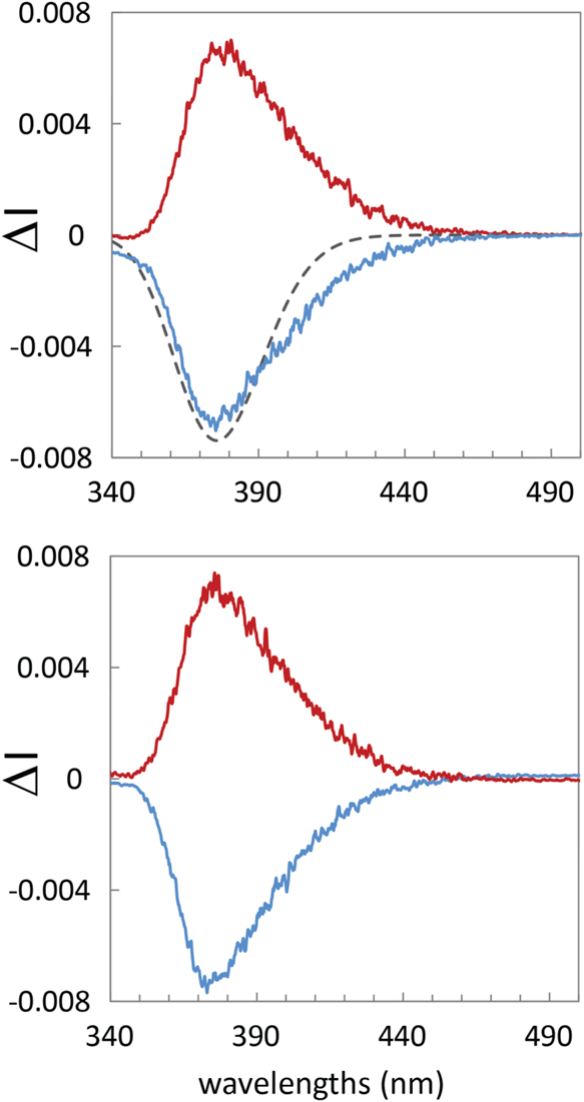
CPL spectra of: (top) (−)-**PrS­[4]** ^***iPe***^ (blue), (+)-**PrS­[4]** ^***iPe***^ (red), and TD-DFT/M06/6–311g­(d,p) computed for all-p*S*-**PrS­[4]** ^***Me***^ model molecule (dashed); (bottom) CPL spectra of (−)-**PrS­[4]** ^***EtCy***^ (blue) and (+)-**PrS­[4]** ^***EtCy***^ (red). CPL has been plotted after normalizing the fluorescence signal recorded with the same apparatus. Excitation wavelength: 345 nm.

The sign of the CPL band correlates with the sign of the longest-wavelength ECD, as expected in all cases when the first excited state is similar in geometry and electronic properties to the ground state.^39^ The dissymmetry ratio for CPL, *g*_lum_ = Δ*I*/*I* = 2­(*I*_L_ – *I*_R_)/(*I*_L_ + *I*_R_), is about 0.008 (reaching 0.01 at 375 nm), comparable to *g*_abs_ of the first ECD band. The value is relatively high for an organic compound in the case of electric dipole-allowed transitions and is significantly higher than the value of 0.002 reported for prism[5]­arenes.^25,40^ Such a high CPL dissymmetry ratio value can be ascribed to the *D*_4_ symmetry of the chromophoric system. In fact, the first chiral molecular square with *D*_4_ symmetry reported in the literature also has a significantly high *g*_abs_.^41^ Furthermore, exceptionally high dissymmetry ratios have been reported for [4]­cyclochrysene derivatives with *D*_4_ symmetry by Sato^13^ and Fukunaga.^14^ This was attributed to “cylindrical chirality” responsible for generating a strong magnetic dipole transition moment.^42^ In this context, our newly synthesized prism[4]­arenes represent active chromophores with *D*_4_ symmetry. To simulate the CPL spectra, we conducted the TD-DFT analysis on a simpler **PrS­[4]** ^*Me*^ (see Figures S91–S95).

The model compound **PrS­[4]** ^*Me*^ reproduces very well the observed ECD on the two molecules, thus confirming that the pendants do not play any significant role in the low-energy bands of the spectrum (see Figure S92).

After optimization of ground and excited state at M06/6–311G­(d,p) level, we conclude that the first bright (i.e., symmetry allowed) transition is an A1→A2 transition both for absorption and emission (see Figure S95), with negative rotational strength for all-p*S* configuration. Ground and excited state structures are quite similar, which requires a low value of the Stokes shift, as observed (17 nm), and quite similar electronic level sequence, dipole and rotational strengths, and configuration interaction pattern. The bright transitions present parallel (antiparallel) electric and magnetic dipole transition moments and are directed along the cylinder axis, like the A1→A2 transition, or perpendicular to it (in this case with degeneracy, see Table S7).

### Chiral Recognition Properties of Prism[4]­arenes

With these results in hand, the chiral recognition^43,44^ abilities of prism[4]­arene were investigated (Table 2). To determine the affinity of racemic **PrS­[4]** ^*iPe*^ for enantiopure ammonium guests, ^1^H NMR titration experiments were conducted using the guests as their barfate salts (**6**–**9** in Figure 7).

**Table 2:** Binding Constant Values (*K*_ass_, M^–1^) Determined by NMR Experiments (SI) and Chiral Selectivity Ratio Calculated by HR-MS Spectrometry Experiments

guest	*K*_ass_ G^+^@ PrS[4]^*iPe*^	chiral selectivity *R*_L_/*R*_D_
**6** ^**2+**^	1900^a^	2.01
**7** ^**+**^	40,000^b^	1.22
**8** ^**+**^	290^a^	0.94
**9** ^**+**^	690^a^	0.70

a Calculated by integrating the free and complexed ^1^H NMR signals of the host.

b Calculated using a ^1^H NMR competition experiment with methoxy-prism[5]­arene as a competitive host.

**Figure 7:**
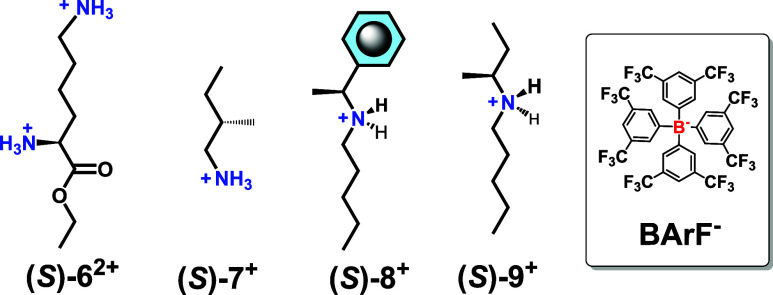
Chemical drawings of enantiopure chiral guests **G** investigated in this study and barfate counterion (BArF^–^).

The selected guests comprised the dicationic ethyl ester of the amino acid Lysine **6** ^2+^, a chiral primary ammonium cation **7** ^+^, a secondary aliphatic ammonium cation **9** ^+^, and a secondary ammonium cation with an aromatic group **8** ^+^ (Figure 7). We began our investigation of the chiral binding affinity with (l)-lysine ethyl ester **6** ^2^+^^. Complexation studies in CD_2_Cl_2_ of *rac*-**PrS­[4]** ^***iPe***^ with (*S*)-**6** ^2^+^^ revealed *endo*-cavity complexation of the −CH_2_CH_2_CH_2_CH_2_NH_3_^+^ moiety within the **PrS­[4]** ^***iPe***^ (see the SI and DFT structures in Figure 8a,e).

**Figure 8:**
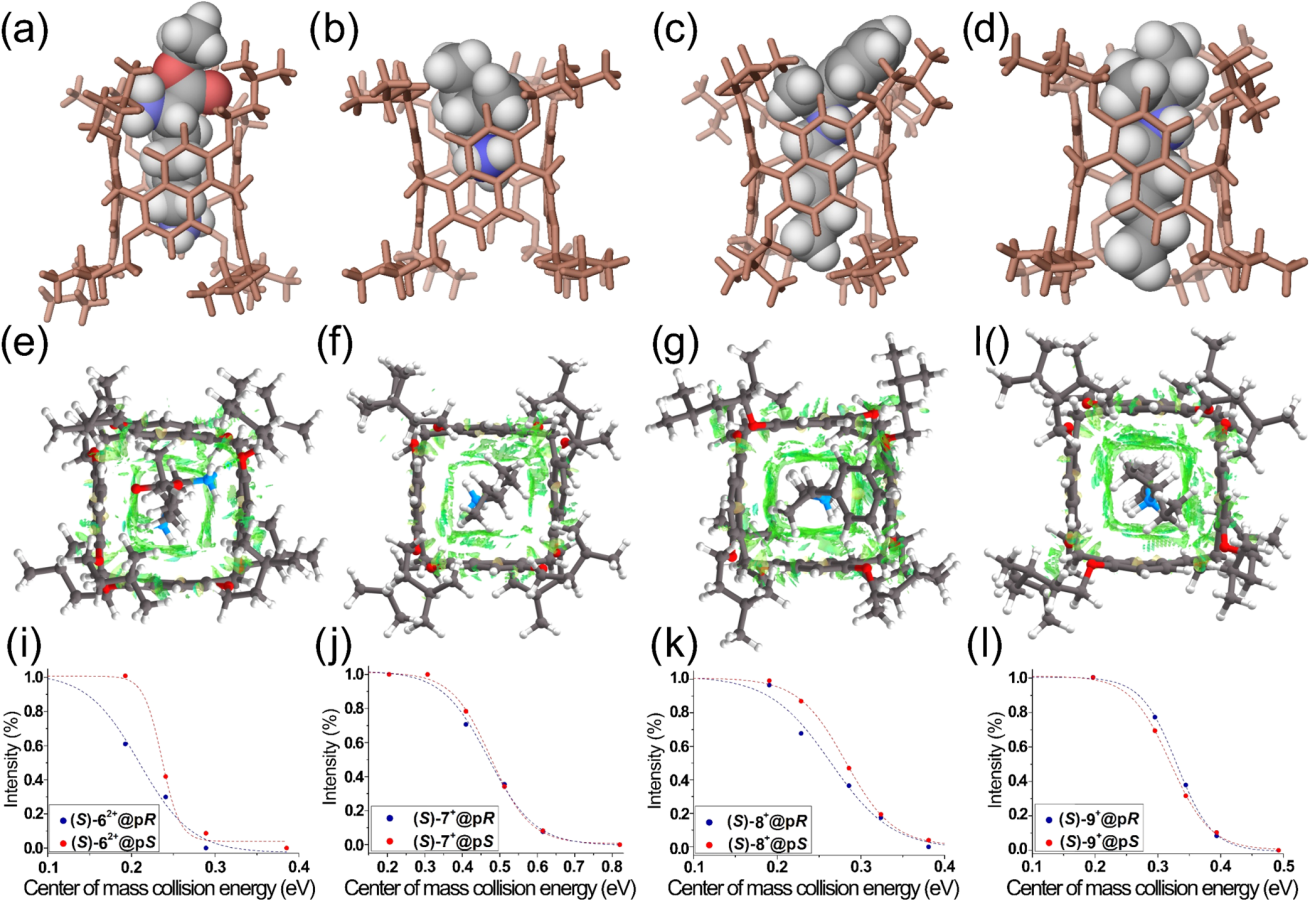
DFT-optimized structure of complexes obtained at B97D3/SVP/SVPFIT level of theory of (a) (*S*)-**6** ^**2**^ **^+^** @*pS*-**PrS­[4]** ^***iPe***^, (b) (*S*)-**7** ^**+**^@*pS*-**PrS­[4]** ^***iPe***^, (c) (*S*)-**8** ^**+**^@*pS*-**PrS­[4]** ^***iPe***^, and (d) (*S*)-**9** ^**+**^@*pS*-**PrS­[4]** ^***iPe***^. Gradient RDG isosurfaces (0.25) for the noncovalent interaction (NCI) regions of (e) (*S*)-**6** ^**2**^ **^+^** @*pS*-**PrS­[4]** ^***iPe***^, (f) (*S*)-**7** ^**+**^@*pS*-**PrS­[4]** ^***iPe***^, (g) (*S*)-**8** ^**+**^@*pS*-**PrS­[4]** ^***iPe***^, and (h) (*S*)-**9** ^**+**^@*pS*-**PrS­[4]** ^***iPe***^. Normalized percentages of the intact (*S*)@*pR* (blue) and (*S*)@*pS* (red) complexes, plotted against collision energy in the center-of-mass frame: (i) **6** ^**2**^ **^+^** @*pS*-**PrS­[4]** ^***iPe***^, (j) **7** ^**+**^@*pS*-**PrS­[4]** ^***iPe***^, (k) **8** ^**+**^@*pS*-**PrS­[4]** ^***iPe***^, (l) **9** ^**+**^@*pS*-**PrS­[4]** ^***iPe***^.

This was evidenced by NMR signals exhibiting negative chemical shifts between 0 and −2.5 ppm (SI). An apparent binding constant of 1900 M^–1^ was determined for the complexation of (*S*)-**6** ^**2** ^**+**^^ with *rac*- **PrS­[4]** ^***iPe***^ in CD_2_Cl_2_. Analysis of the 1D and 2D NMR spectra revealed considerable difficulty in distinguishing the signals of the two (*S*)-**6** ^**2**^ **^+^** @*pR*-**PrS­[4]** ^***iPe***^ and (*S*)-**6** ^**2**^ **^+^** @*pS*-**PrS­[4]** ^***iPe***^ diastereomers. This isochronicity is likely favored by the conformational rigidity of the prismarenic framework.

Given the difficulty in distinguishing the two diastereomeric complexes using 1D and 2D NMR in solution, we employed MS/MS techniques in the gas phase, utilizing soft ionization methods, specifically, electrospray ionization (ESI). This approach facilitated a detailed analysis of the gas-phase stability and chiral recognition of guests **6**–**9** with **PrS­[4]** ^***iPe***^.^17,45,46^ The high-resolution electrospray ionization Fourier transform ion cyclotron resonance (HR ESI-FT-ICR) mass spectrum (Figure S37) of an equimolar mixture of *rac*-**PrS­[4]** ^***iPe***^ and **6** ^**2**^ **^+^** in CH_2_Cl_2_ displayed a molecular ion peak at *m*/*z* 712.4947, consistent with the molecular formula of the complex (SI). To further investigate the relative stability of the (*S*)-**6** ^**2**+^@all-p*S*-**PrS­[4]** ^***iPe***^ complex (Figure 8a) in comparison to the (*S*)-**6** ^**2**+^@all-p*R*-**PrS­[4]** ^***iPe***^ diastereoisomeric complex in the gas phase, collision-induced dissociation (CID) experiments were conducted at collision energies ranging from 0.1 to 0.4 eV for each **PrS­[4]** ^***iPe***^ enantiomer (Figure 8).^46,47^ An equimolar solution of all-p*S*-**PrS­[4]** ^***iPe***^ (or all-p*R*-**PrS­[4]** ^***iPe***^) and (*S*)-**6** ^**2**+^ in dichloromethane was prepared, and collision experiments were conducted by isolating the complex and subjecting it to collisions at different energies.

As illustrated in Figure 8i, the normalized percentage of the intact complex was plotted against the collision energy in the center-of-mass frame.^45^ (*S*)-**6** ^**2**+^@all-p*S*-**PrS­[4]** ^***iPe***^ exhibited greater resistance to dissociation compared to the (*S*)-**6** ^**2**+^@all-p*R*-**PrS­[4]** ^***iPe***^ complex. Specifically, while 50% of the (*S*)-**6** ^**2**+^@all-p*R*-**PrS­[4]** ^***iPe***^ complex dissociated at 0.21 eV, only 7% of the (*S*)-**6** ^**2**+^@all-p*S*-**PrS­[4]** ^***iPe***^ complex fragmented under the same conditions. To estimate the ability of all-p*R*-**PrS­[4]** ^***iPe***^ and all-p*S*-**PrS­[4]** ^***iPe***^ to discern the chirality of l-lysine methyl ester, we have calculated the *R*_chiral_ ratio (*R*_L_/*R*_D_).^47^ Here, *R*_L_ is defined as *I*_[(*S*)‑**6** ^++^@all‑p*S*‑**PrS[4]** ^***iPe***^]_/ *I*_[all‑p*S*‑**PrS[4]** ^***iPe***^]_ and *R*_D_ as *I*_[(*S*)‑**6** ^++^@all‑p*R*‑**PrS[4]** ^***iPe***^]_/ *I*_[all‑p*R*‑**PrS[4]** ^***iPe***^]_ (*I* = signal intensity in mass spectra).^46,47^ An *R*_chiral_ value approaching 1 indicates a diminished capacity for chiral recognition, while values greater than or less than 1 suggest relatively high chiral recognition capability.^47^ Mass spectra for equimolar solutions (1 × 10^–4^ mol/L) of l-lysine methyl ester and all-*pS*-**PrS­[4]** ^***iPe***^, and l-lysine methyl ester and all-*pR*-**PrS­[4]** ^***iPe***^ were analyzed, yielding an *R*_chiral_ of 2.01 (Table 2). Thus, in agreement with the results of the CID experiments, the *R*_chiral_ measurements also indicate that the binding strength of the (*S*)-**6** ^**2**+^@all-p*S*-**PrS­[4]** ^***iPe***^ is greater than that of (*S*)-**6** ^**2**+^@all-p*R*-**PrS­[4]** ^***iPe***^.^18^

Density functional theory (DFT) calculations (B97D3/SVP/SVPFIT level of theory, Figure 8a,e) show that the (*S*)-**6** ^**2**^ **^+^** @all-*pS*-**PrS­[4]** ^***iPe***^ complex is more stable than the (*S*)-**6** ^**2**^ **^+^** @all-*pR*-**PrS­[4]** ^***iPe***^ by 1.31 kcal/mol. Specifically, a stronger H2N^+^–H···O^prismarene^ H-bond interaction (NH···O distance of 2.7 Å, angle of 173°) was identified in the (*S*)-**6** ^**2**^ **^+^** @all-*pS*-**PrS­[4]** ^***iPe***^ complex, in comparison to the (*S*)-**6** ^2^+^^@all-*pR*-**PrS­[4]** ^***iPe***^ complex (NH···O distance = 2.8 Å, angle = 161°). NBO analysis corroborated this, showing that hydrogen bonding interaction contributed 29% of the total interaction energy in the (*S*)-**6** ^**2**^ **^+^** @all-*pS*-**PrS­[4]** ^***iPe***^ complex, compared to 17% in the (*S*)-**6** ^**2**^ **^+^** @all-*pR*-**PrS­[4]** ^***iPe***^. The molecular model shows that the ester group is significantly outside the cavity (Figure 8a).

With these results in hand, we investigated the chiral recognition abilities of simpler and smaller (*S*)-**7** ^+^. The addition of one equivalent of (*S*)-**7** ^+^ to a dichloromethane solution of racemic **PrS­[4]** ^*iPe*^ resulted in significant changes in their ^1^H NMR spectra. The observed upfield shift of the guest’s ^+^N–CH_2_ signal to 1.00 ppm (see the SI) indicates the formation of the (*S*)-**7** ^+^@**PrS­[4]** ^***iPe***^ complex. In contrast, no significant chemical shift change was observed for the CH_3_ and CH_3_CH_2_ protons, indicating that this portion of the chain remains outside the cavity (see Figure 8b). In the ^1^H NMR spectrum of the equimolar *rac*-**PrS­[4]** ^*iPe*^/(*S*)-**7** ^+^ mixture, the signals for the free host were absent. The apparent binding constant for the complexation of *rac*-**PrS­[4]** ^***iPe***^ in CD_2_Cl_2_ with (*S*)-**7** ^**+**^ was determined to be 40000 M^–1^ via a competition experiment using **PrS­[5]** ^***Me***^. The HR ESI-FT-ICR mass spectrum (Figure S41) of an equimolar mixture of **PrS­[4]** ^***iPe***^ and **7** ^**+**^ in CH_2_Cl_2_ displayed a molecular ion peak at *m*/*z* 1336.9503, which aligns with the molecular formula of the complex. Gas-phase collision-induced dissociation (CID) and *R*_chiral_ ratio for the diastereoisomeric complexes reveal that the (*S*)-**7** ^+^@all-p*S*-**PrS­[4]** ^*iPe*^ complex is only slightly more stable than the (*S*)-**7** ^+^@all-p*R*-**PrS­[4]** ^*iPe*^ complex (Figure 8j). Specifically, 50% dissociation of (*S*)-**7** ^+^@all-p*R*-**PrS­[4]** ^***iPe***^ occurred at 0.47 eV, while approximately 45% of (*S*)-**7** ^+^@all-p*S*-**PrS­[4]** ^***iPe***^ dissociated under the same conditions. This translates to a *R*_chiral_ value of 1.22 (Table 2). DFT calculations (Figure 8b) show that the (*S*)-**7** ^**+**^@all-*pS*-**PrS­[4]** ^***iPe***^ complex is more stable than (*S*)-**7** ^**+**^@all-*pR*-**PrS­[4]** ^***iPe***^ by 1.10 kcal/mol, confirming the generally greater stability of the (*S*)@all-*pS* complexes.

Natural Bond Orbital (NBO) analysis (Figure 8f) indicates that approximately 70% of the total interaction energy of the complex between **7** ^**+**^ and **PrS­[4]** ^***iPe***^ arises from CH···π and van der Waals interactions (Figure 8f). NBO calculations suggest significant contributions from the interactions of the chiral center with the aromatic cavity of **PrS­[4]** ^***iPe***^. In particular, the CH group of the chiral center exhibits slightly stronger CH···π interactions in the (*S*)-**7** ^**+**^@all-p*S*- **PrS­[4]** ^***iPe***^ complex (10% of the total interaction energy) compared to the (*S*)-**7** ^**+**^@all-p*R*- **PrS­[4]** ^***iPe***^ complex (7% of the total interaction energy). In the (*S*)-**7** ^**+**^@all-p*S*- **PrS­[4]** ^***iPe***^ complex, the CH···π interaction of the ^+^NCH_2_ group contributes approximately 26% of the total interaction energy. The interaction angles (^+^NCH···π) differ significantly, measuring 129.4° in (*S*)-**7** ^**+**^@all-p*S*-**PrS­[4]** ^***iPe***^ versus 106.3° in (*S*)-**7** ^**+**^@all-p*R*- **PrS­[4]** ^***iPe***^, indicating a stronger and more directional interaction in the (*S*)-**7** ^**+**^@all-p*S*- **PrS­[4]** ^***iPe***^ complex. The CH_3_ and ethyl groups interact primarily through van der Waals forces, remaining peripheral, whereas the NH_3_^+^ group is deeply embedded within the aromatic cavity (Figure 8b,f).

The chiral recognition properties of **PrS­[4]** ^*iPe*^ toward the secondary ammonium cation with the phenyl group (*S*)-**8** ^**+**^ in CD_2_Cl_2_ were also studied using NMR analysis, which indicated *endo*-cavity complexation of the pentyl chain within the **PrS­[4]** ^*iPe*^ cavity (Figure 8c). An apparent binding constant of 290 M^–1^ was calculated for the formation of the complex. In the ^1^H NMR spectrum (SI) of the equimolar (S)-**8** ^**+**^/**PrS­[4]** ^*iPe*^ mixture, the pentyl chain protons of **8** ^**+**^displayed significantly shielded signals with chemical shifts at −2.27, −1.34, −0.09, 1.10, and 1.60 ppm (corresponding to the α, β, γ, δ, and ε protons, as identified by COSY and HSQC). 1D and 2D NMR studies (Supporting Information), corroborated by DFT calculations (Figure 8c,g), indicate that the chiral center of **8** ^**+**^ resides outside the **PrS­[4]** ^***iPe***^ cavity. CID investigations revealed that 50% of the (*S*)-**8** ^**+**^@*all*-p*R*-**PrS­[4]** ^***iPe***^ complex dissociates at 0.26 eV, while 31% of the (*S*)-**8** ^**+**^@*all*-p*S*-**PrS­[4]** ^***iPe***^ complex undergoes fragmentation under the same conditions. DFT calculations (Figure 8c) indicate that the (*S*)-**8** ^**+**^@*all*-p*S*-**PrS­[4]** ^***iPe***^ complex is slightly more stable than (*S*)-**8** ^**+**^@*all*-p*R*-**PrS­[4]** ^***iPe***^ by 0.51 kcal/mol. Analysis of mass spectra for equimolar solutions (1 × 10^–4^ mol/L) of (S)-**8** ^+^ and (*S*)-**8** ^**+**^@*all*-p*S*-**PrS­[4]** ^***iPe***^/(*S*)-**8** ^**+**^@*all*-p*R*-**PrS­[4]** ^***iPe***^ yielded an *R*_chiral_ value very close to unity (Table 2).

NMR spectroscopic analysis of the secondary aliphatic ammonium cation (*S*)-**9** ^+^ reveals *endo*-cavity complexation of its linear moiety, evidenced by α-CH_2_ and α-CH chemical shifts at −2.24 and 3.19 ppm, respectively. The *sec*-butyl group remains outside the cavity (Figure 8d). The (*S*)-**9** ^+^/*rac*-**PrS­[4]** ^***iPe***^ apparent binding constant is 690 M^–1^. As shown in Figure 8h, the (*S*)-**9** ^**+**^@*all*-p*R*-**PrS­[4]** ^***iPe***^ complex exhibits marginally greater dissociation resistance than its counterpart, (*S*)-**9** ^+^@*all*-p*S*-**PrS­[4]** ^***iPe***^, with approximately 50 and 55% dissociation observed at 0.33 eV, respectively. This is supported by a calculated *R*_chiral_ value of 0.7 (Table 2), indicating a slight preference for the (*S*)-**9** ^**+**^@*all*-p*R*-**PrS­[4]** ^***iPe***^ complex. The DFT calculations (Figure 8d,h) show a negligible energy difference between the two diastereomeric complexes (0.06 kcal/mol).

Regarding the chiral recognition properties of this new **PrS­[4]** ^***iPe***^ macrocycle, the results indicate a preference toward the formation of *S*-(guest)@*all*-p*S*-**PrS­[4]** ^***iPe***^ complexes. Interestingly, the highest stereoselectivity has been observed for amino acid derivative **6** ^**2+**^, which could lead to specific biological applications of this new member of the prismarene family.

## Conclusions

In this study, we report for the first time inherently chiral prismarenes with resolvable enantiomers **PrS­[4]** ^*iPe*^ and **PrS­[4]** ^*EtCy*^. Prism[4]­arenes were synthesized through a thermodynamic template approach using a tailor-made selective cation, designed for the prism[4]­arene cavity. The prism[4]­arene scaffold, characterized by its narrow annulus, effectively prevents the flipping of the naphthalene rings observed for the other members of the prismarene family (PrS[5] and PrS[6]), thereby exhibiting persistent conformational chirality. The solid-state structure of this new macrocycle revealed that the centrosymmetric crystal of **PrS­[4]** ^*iPe*^ is composed of a racemic mixture of all-p*R* and all-p*S* enantiomeric pairs. The crystal packing demonstrates the formation of supramolecular polymeric chains of homochiral **PrS­[4]** ^*iPe*^ molecules in the solid state.

The enantiomers were successfully isolated using chiral HPLC, and their chiroptical properties were thoroughly investigated. Configurational assignment was carried out through TDDFT computations of [α]_D_ and ECD/UV–vis spectra. The circularly polarized luminescence (CPL) properties of these new prism[4]­arenes were also explored, yielding a dissymmetry ratio for CPL of 0.008. This value is notably high for an organic compound exhibiting electric dipole-allowed transitions. The correlation between the *g*_lum_ value and the molecular structure underscores the significance of the *D*_4_ symmetry of the chromophore. This distinctive attribute is essential for generating a strong magnetic dipole transition moment and an intense associated rotational strength. Finally, the chiral recognition properties of the prism[4]­arene toward chiral enantiopure guests were assessed using NMR and gas-phase techniques, specifically, HR-ESI-FT-ICR mass spectrometry and MS/MS methods. Interestingly, the formation of *S*-(guest)@*all*-p*S*-**PrS­[4]** ^***iPe***^ complexes is favored, and the most significant enantioselective recognition was observed for the (*S*)-Lysine derivative. The results presented here could pave the way for the development of new chiral materials with intriguing chiroptical properties based on prism[4]­arene.

## Supplementary Material



## Data Availability

The data underlying this study are available in the published article and its Supporting Information

## Notes

Detailed synthetic procedures, complexation studies, NMR and HR MS spectra, and details of calculations (PDF)
