# Supplementary material for: Introducing Prism[4]arene: A Macrocycle with Enantiomerically Resolvable Inherent Chirality and Intriguing Chiroptical Properties
Source: J Am Chem Soc. 2025 Jun 4;147(24):20843–54. doi: 10.1021/jacs.5c04512 (PMC12186475; doi:10.1021/jacs.5c04512)
Supplement: Supplementary file 1 [file ja5c04512_si_001.pdf]

Supporting Information

Introducing Prism[4]arene: A Macrocycle with Enantiomerically Resolvable Inherent Chirality and Intriguing Chiroptical Properties

Paolo Della Sala,[a] Carmen Talotta,[a] Margherita De Rosa,[a] Stefano Superchi,[*,b] Ernesto Santoro,[b] Silvano Geremia,[*,c] Neal Hickey,[c] Marco Fusè,[d] Sergio Abbate,[d] Giuseppe Mazzeo,[d] Giovanna Longhi, [*,d] Carmine Gaeta [*,a]

[a]Laboratory of Supramolecular Chemistry, Dipartimento di Chimica e Biologia "A. Zambelli", Università di Salerno, Via Giovanni Paolo II 132, 84084 Fisciano, Salerno, Italy. *Email: cgaeta@unisa.it.

[b]Dipartimento di Scienze di Base e Applicate, Università della Basilicata, Via dell'Ateneo Lucano 10, 85100 Potenza, Italy; *Email: stefano.superchi@unibas.it.

[c]Centro di Eccellenza in Biocristallografia, Dipartimento di Scienze Chimiche e Farmaceutiche, Università di Trieste, Via L. Giorgieri 1, I-34127 Trieste, Italy; *Email: sgeremia@units.it.

[d]Dipartimento di Medicina Molecolare e Traslazionale, Università di Brescia, Viale Europa 11, 25123 Brescia, Italy. *Email: giovanna.longhi@unibs.it.

Table of Contents	Pages
General Experimental Details	S2
General procedure for the synthesis of Prism[4]arenes.	S3
Copies of NMR and HR Mass Spectra of Prism[4]arenes	S5
1D and 2D NMR Studies on the Complexation of Prism[4]arenes with Achiral Guests (**2**$^+$ to **5**$^+$)	S15
Details on the Calculation of Association Constants for the Complexation of Prism[4]arenes with Achiral Guests (**2**$^+$ to **5**$^+$)	S30
1D and 2D NMR Studies on the Complexation of **PrS[4]**^{iPe} with Chiral Guests (*S*)-**6**$^{2+}$,(*S*)-**7**$^+$,(*S*)-**8**$^+$ and (*S*)-**9**$^+$.	S39
Details on the Calculation of Association Constants for the Complexation of **PrS[4]**^{iPe} with Chiral Guests (*S*)-**6**$^{2+}$,(*S*)-**7**$^+$,(*S*)-**8**$^+$ and (*S*)-**9**$^+$	S55
DFT - optimized structures of Complexes	S61
NBO and NCI analysis	S105
Chiral HPLC separation of enantiomers **PrS[4]**^{EtCy} and **PrS[4]**^{iPe}	S108
Crystallographic structure determination of **PrS[4]**^{iPe}	S110
UV-Vis and fluorescence characterization	S116
ECD STUDIES	S117
CPL STUDIES	S125
Collision-induced dissociation (CID) experiment and chiral selectivity	S130
References	S131

General Experimental Details

All chemical reagents were used as obtained by TCI, Fluorochem and Merck, with no additional purification. Reaction temperatures were measured externally and were monitored by Merck TLC silica gel plates (0.25 mm) and visualized by UV light at 254 nm, or by spraying with H_2SO_4-$Ce(SO_4)_2$. NMR spectra were recorded on a Bruker Avance-600 [600 (^1H) and 150 MHz (^{13}C)] and Avance-400 [400 (^1H) and 100 MHz (^{13}C)] spectrometers. Chemical shifts are reported relative to the residual solvent peak. Standard pulse programs, provided by the manufacturer, were used for 2D COSY (cosygpqf) and 2D HSQC (hsqcedetgpsisp2.2) experiments. Structural assignments were made with additional information from gCOSY and gHSQC experiments.
HR MALDI mass spectra of hosts were recorded on a Bruker Solaris XR Fourier transform ion cyclotron resonance mass spectrometer equipped with a 7T refrigerated actively shielded superconducting magnet. Each sample (1 mg/mL in dichloromethane) was mixed with DHB (10 mg/mL in acetone) and 1 µL of solution was deposited on MALDI Plate. The samples were ionized in positive ion mode using the MALDI ion source, and 16 laser shots were used for each scan. The mass spectra were calibrated externally using NaTFA and a linear calibration was applied.
The UV−Vis spectra were recorded in dichloromethane on a Varian Cary 50 UV-Vis spectrophotometer and fluorescence spectra were recorded on a Varian Cary Eclipse Spectrophotometer at room temperature, 10 mm quartz cells.
Optical rotation measurements were performed on a JASCO DIP-370 polarimeter. Electronic circular dichroism (ECD) spectra were recorded with a JASCO J815 spectropolarimeter, employing *n*-hexane solvent, room temperature in quartz cells.

General procedure for the synthesis of Prism[4]arenes.

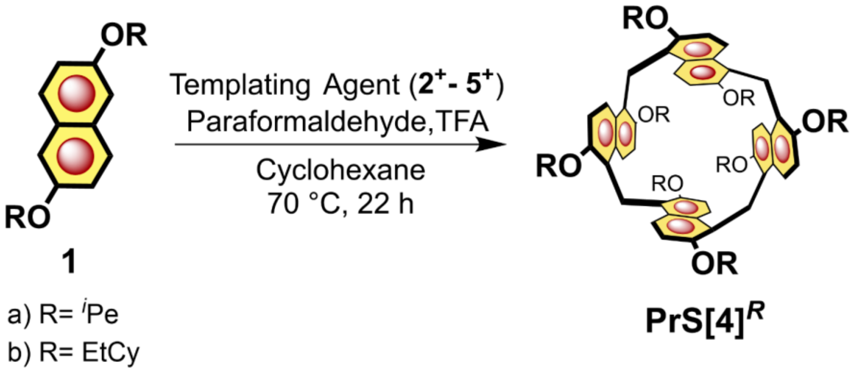

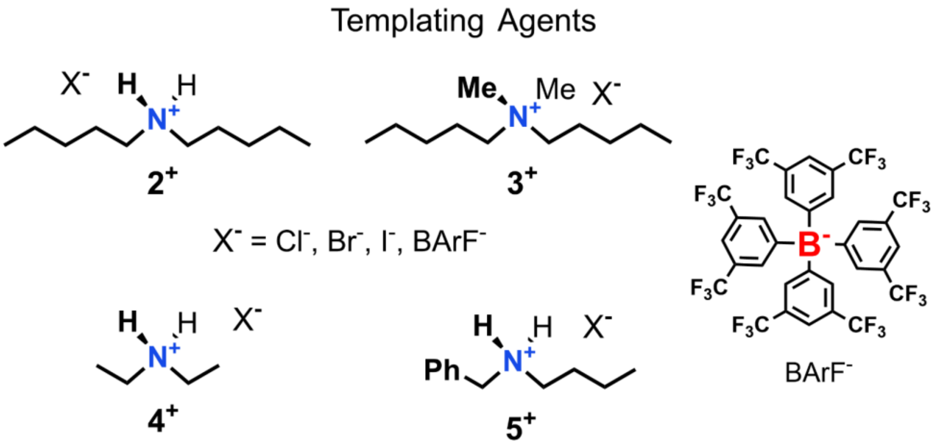

A solution of 2,6-bis(isopentyloxy)naphthalene (0.100 g, 0.33 mmol), paraformaldehyde (12.0 mg, 0.40 mmol, 1.2 equiv), and templating agent, **2^{+}·Cl^{-}** , **3^{+}·Cl^{-}**, **4^{+}·Cl^{-}** or **5^{+}·Cl^{-}** (0.33 mmol, 1.0 equiv) in 67 mL of cyclohexane was heated to 70° C, then trifluoroacetic acid (0.38 mL, 5.0 mmol, 15 equiv) was added. The solution was stirred for 22 h at 70° C and subsequently the solvent evaporated under reduced pressure. The residue was dissolved in CH_2Cl_2 (30 mL) and the mixture was washed with an aqueous saturated solution of $NaHCO_3$ (30 mL). Finally, the organic layer was washed with brine (2x20 mL), and the organic phases were dried over sodium sulfate and concentrated to give a solid light brown. The crude product was purified by chromatographic column on silica gel (hexane/dichloromethane = 7/3).

Template synthesis with 2^{+}·Cl^{-} salt: **PrS[4]** ^{iPe} was obtained in 20% yield.

Template synthesis with 3^{+}· Cl^{-} salt: **PrS[4]** ^{iPe} was obtained in 5% yield.

Template synthesis with 4^{+}· Cl^{-} salt: **PrS[4]** ^{iPe} was obtained in 4% yield.

Template synthesis with 5^{+}· Cl^{-} salt: **PrS[4]** ^{iPe} was obtained in 4% yield.

A solution of 2,6-bis(2-cyclohexylethoxy)naphthalene (0.100 g, 0.26 mmol), paraformaldehyde (12.0 mg, 0.31 mmol, 1.2 equiv), and templating agent, **2^{+}·Cl^{-}** (50.0 mg, 0.26 mmol, 1.0 equiv) in 53 mL of cyclohexane was heated to 70° C, then trifluoroacetic acid (0.30 mL, 3.9 mmol, 15 equiv) was added. The solution was stirred for 22 h at 70° C and subsequently the solvent evaporated under reduced pressure. The residue was dissolved in CH_2Cl_2 (30 mL) and the mixture was washed with an aqueous saturated solution of

$NaHCO_3$ (30 mL). Finally, the organic layer was washed with brine (2x20 mL), and the organic phases were dried over sodium sulfate and concentrated to give a solid light brown. The crude product was purified by chromatographic column on silica gel (hexane/dichloromethane = 8/2). **PrS[4]^{EtCy}** was obtained in 15 % yield (14 mg).

PrS[4]^{iPe}:

Mp: >263 °C dec.

^{1}H NMR (CD_2Cl_2, 600 MHz, 298 K): *δ* 8.19 (*d*, 8H, Ar-*H,* *J* = 9.4 Hz), 6.89 (*d*, 8H, Ar-*H,* *J* = 9.4 Hz), 4.68 (*s*, 8H, ArC*H$_2$*Ar), 4,20 (*m*, 8H, OC*H$_2$*) and 4.10 (*m*, 8H, OC*H$_2$*), 2.04 (*m*, 8H, C*H*(CH_3)$_2$), 1.86 (q, 16H, J_1 = 13.6 Hz and J_2 = 6.9 Hz, C*H$_2$*CH), 1.07 (d, 24H, *J* = 6.7 Hz, C*H$_3$*), 1.04 (d, 24H, *J* = 6.7 Hz, C*H$_3$*).
^{13}C NMR (CD_2Cl_2, 150 MHz, 298 K): *δ* 151.1, 130.5, 125.1, 124.1,114.3, 68.4, 39.2, 30.1, 25.7, 22.9, 20.3.
HRMS (MALDI) m/z [M]$^+$ calcd for $C_{84}H_{112}O_8$: 1248.8352. found: 1248.8354.

PrS[6] ^{iPe}:

Mp: 211.3-212.0 °C

^{1}H NMR (CD_2Cl_2, 600 MHz, 233 K): *δ* 8.39 (d, 4H, Ar-*H*, *J* = 9.2 Hz), 7.99 (d, 4H, Ar-*H*, *J* = 9.2 Hz), 7.65 (d, 4H, Ar-*H*, *J* = 9.2 Hz), 7.34 (d, 4H, Ar-*H*, *J* = 9.2 Hz), 7.05 (d, 4H, Ar-*H*, *J* = 9.2 Hz), 6.43 (d, 4H, Ar-*H*, *J* = 9.2 Hz), 4.78 (m, 8H, ArC*H$_2$*Ar), 4.40 (s, 4H, ArC*H$_2$*Ar), 4.29 (overlapped, 8H, OC*H$_2$*), 4.17-4.10 (overlapped, 8H, OC*H$_2$*), 3.15 (broad, 4H, OC*H$_2$*), 2.64 (broad, 4H, OC*H$_2$*), 2.07- 1.75 (overlapped, 32H, C*H$_2$*CH and C*H*(CH_3)$_2$), 1.13 (broad, 4H, C*H$_2$*), 1.02 - 0.87 (overlapped, 60H, C*H$_3$*), 0.55-0.46 (overlapped, 12H, C*H$_3$*).

^{13}C NMR (TCDE, 150 MHz, 213 K): *δ* 151.8, 129.9, 124.5, 124.5, 114.7, 67.7, 38.3, 25.0, 24.8, 22.6, 22.5, 22.5, 22.4.

HRMS (MALDI) m/z [M]$^+$ calcd for $C_{126}H_{168}O_{12}$: 1873.2530. found: 1873.2530.

PrS[4]^{EtCy}:

Mp: >185 °C dec.

^{1}H NMR (CD_2Cl_2, 600 MHz, 298 K): *δ* 8.19 (*d*, 8H, Ar-*H,* *J* = 9.4 Hz), 6.89 (*d*, 8H, Ar-*H,* *J* = 9.4 Hz), 4.68 (*s*, 8H, ArC*H$_2$*Ar), 4,20 (*m*, 8H, OC*H$_2$*) and 4.10 (*m*, 8H, OC*H$_2$*), 1.88-1.70 (*overlapped*, 24H, C*H*$_2$), 1.29 - 0.86 (overlapped, 80 H, C*H$_2$ and* C*H*).

^{13}C NMR (CD_2Cl_2, 150 MHz, 298 K): *δ* 151.0, 130.5, 125.0, 124.0, 114.2, 67.8, 37.8, 35.2, 34.0, 33.8, 27.0, 26.9, 26.8, 20.2.

HRMS (MALDI) m/z [M]$^+$ calcd for $C_{108}H_{144}O_8$: 1569.0856. found: 1569.0890.

Copies of NMR and HR Mass Spectra of Prism[4]arenes

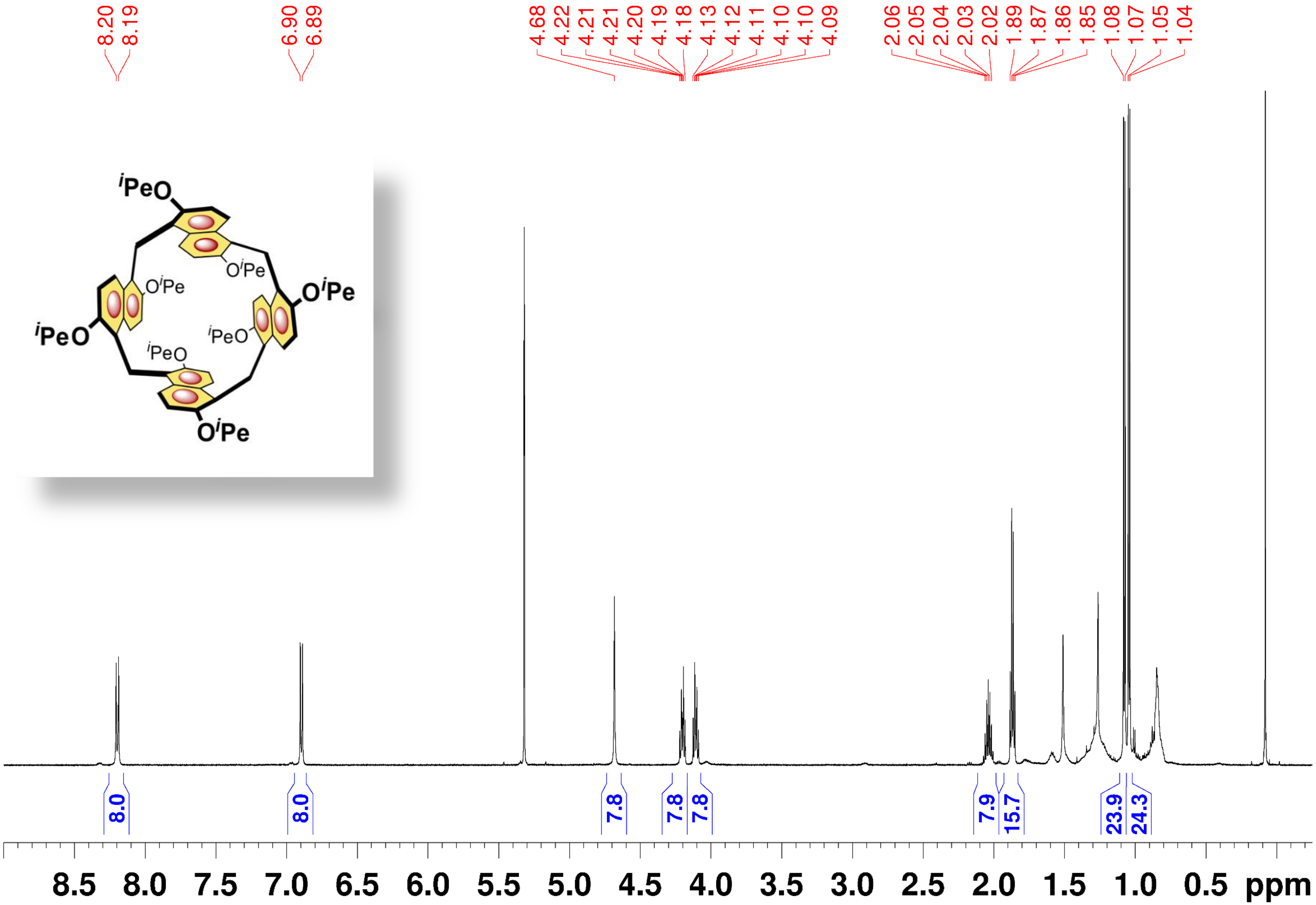

Figure S1: ^{1}H NMR spectrum of **PrS[4]^{iPe}** (CD_2Cl_2, 600 MHz, 298 K).

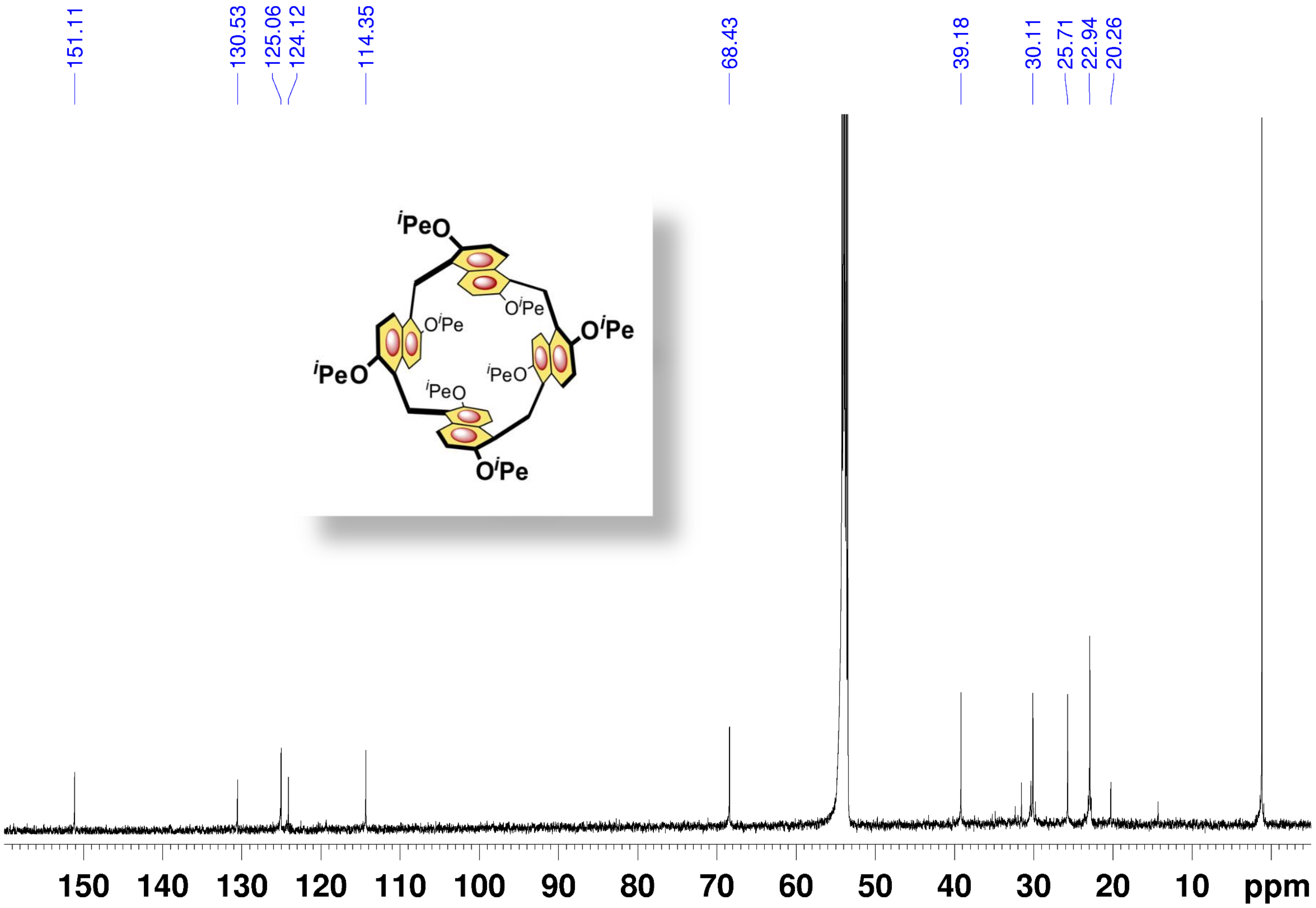

Figure S2: {^{1}H} ^{13}C NMR spectrum of **PrS[4]^{iPe}** (CD_2Cl_2, 150 MHz, 298 K).

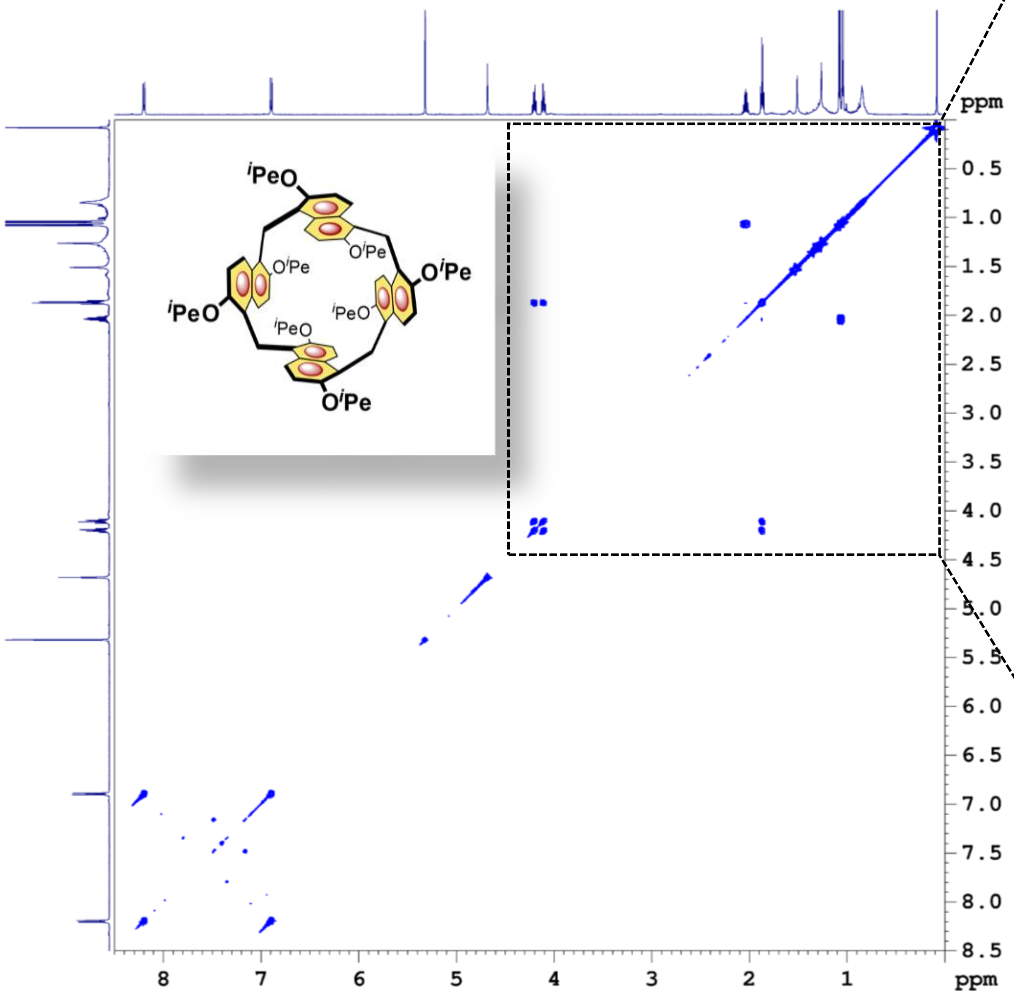

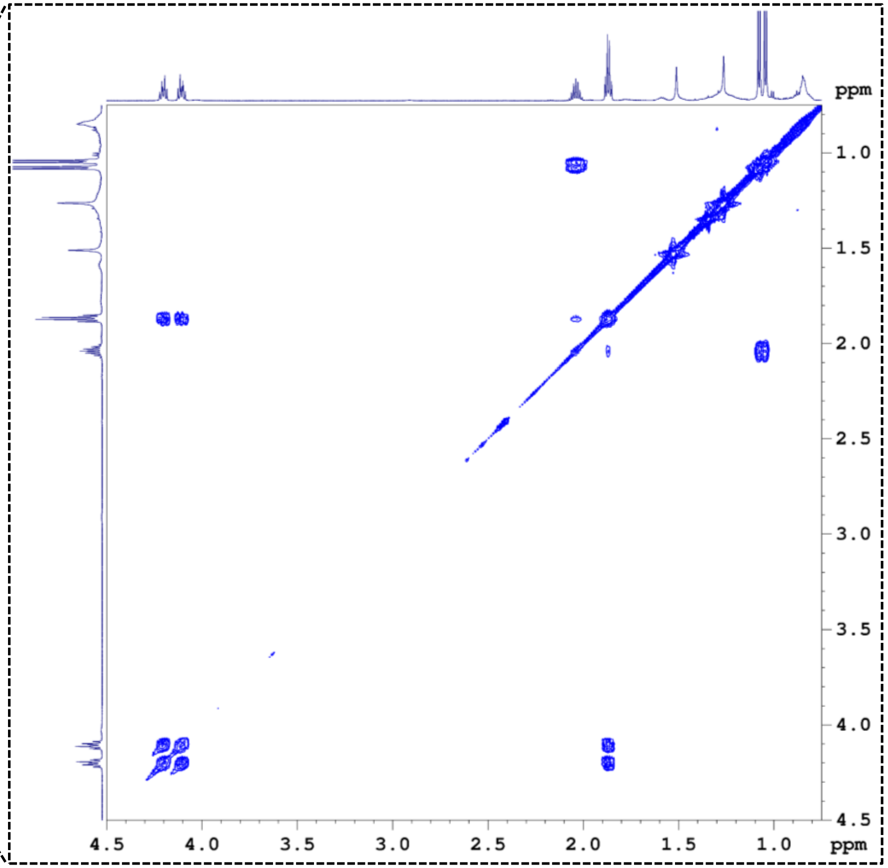

Figure S3: 2D-DQF COSY spectrum of **PrS[4]**^{iPe} (CD_2Cl_2, 600 MHz, 298 K).

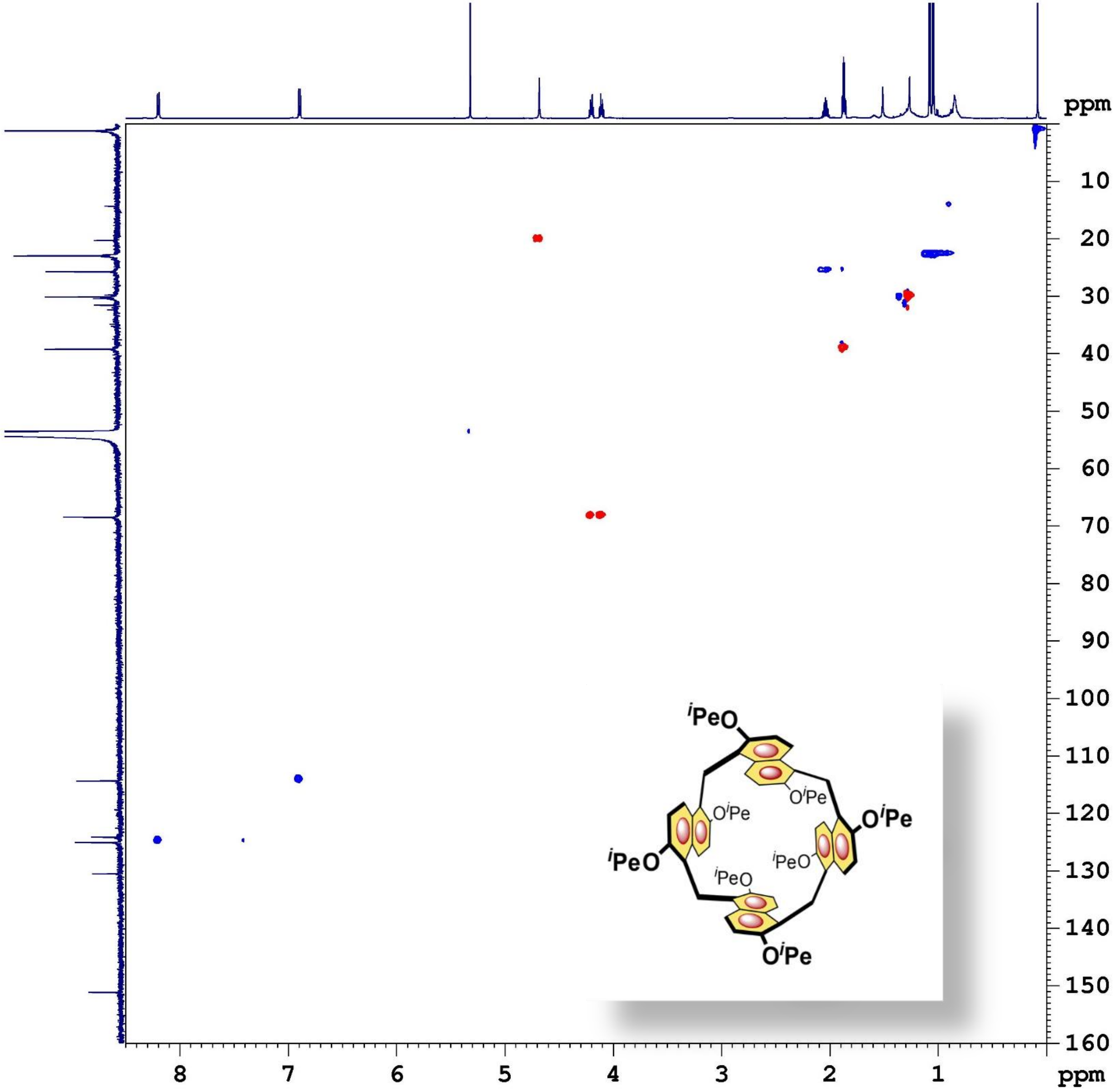

Figure S4: 2D-HSQC spectrum of **PrS[4]**^{iPe} (CD_2Cl_2, 600 MHz, 298 K).

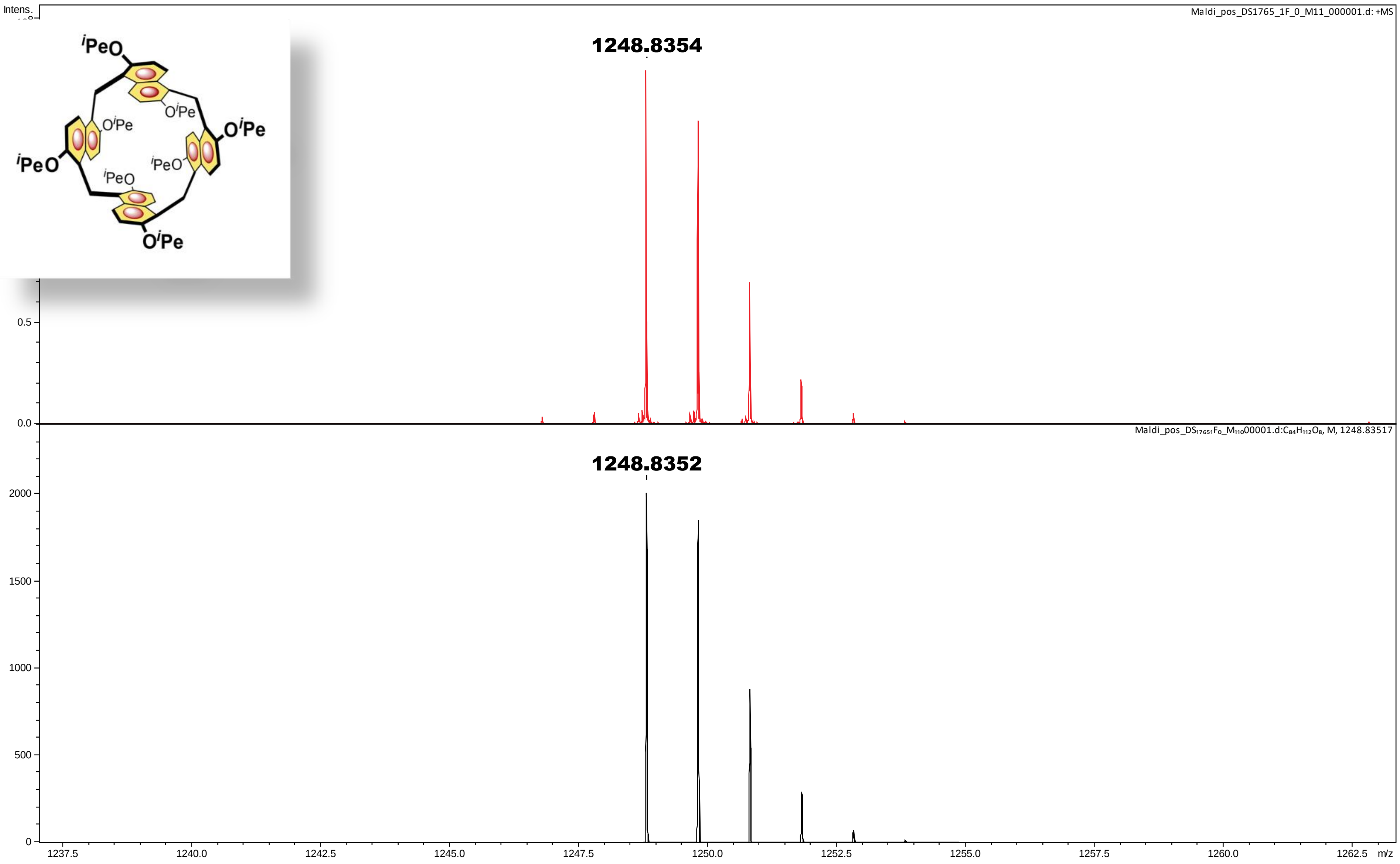

Figure S5: Comparison of experimental (top) and simulated (bottom) HR FT-ICR mass spectra of **PrS[4]**^{iPe}.

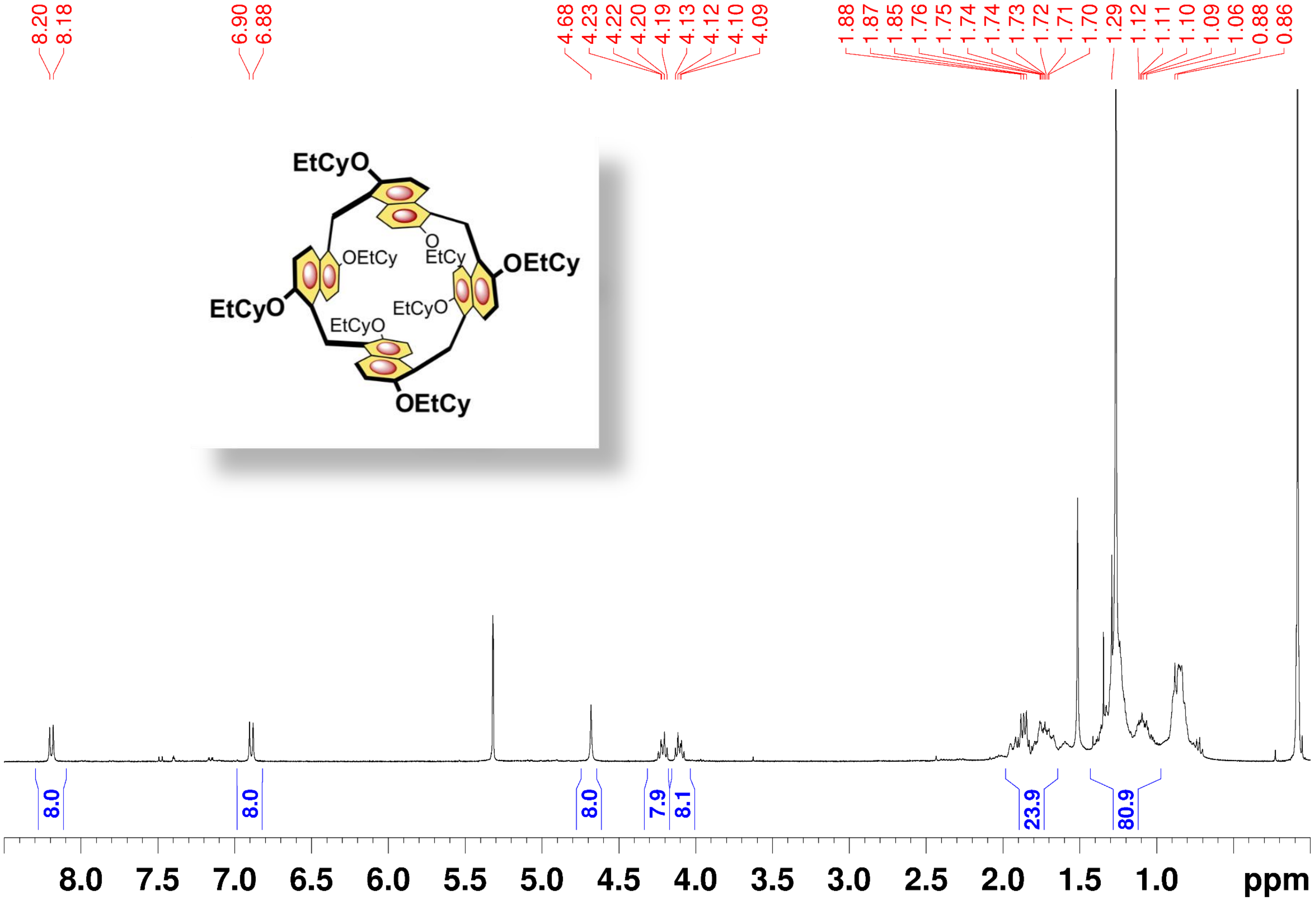

Figure S6: ^{1}H NMR spectrum of **PrS[4]**$^{\textit{EtCy}}$ (CD_2Cl_2, 600 MHz, 298 K).

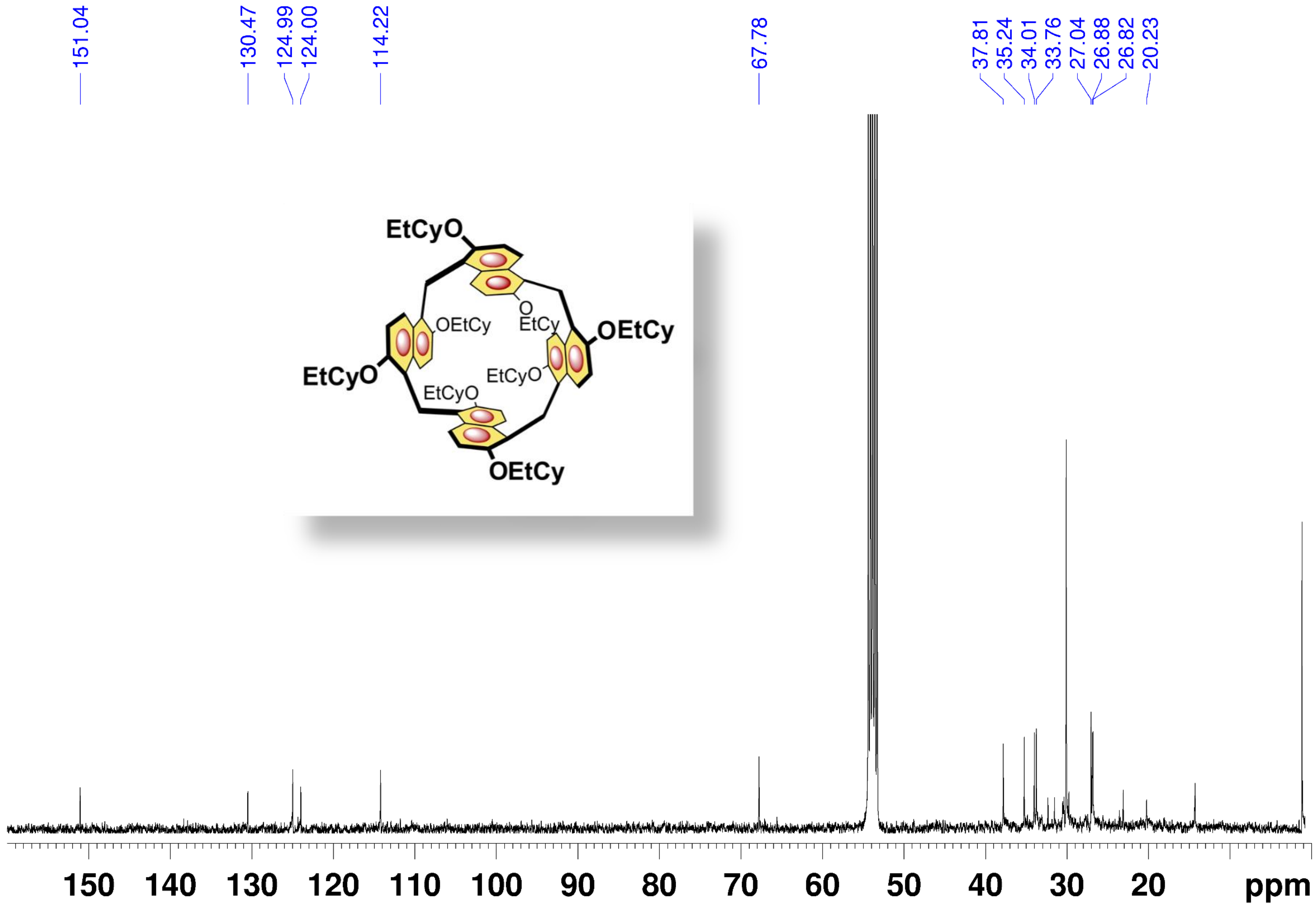

Figure S7: {^{1}H} ^{13}C NMR spectrum of **PrS[4]**^{EtCy} (CD_2Cl_2, 150 MHz, 298 K).

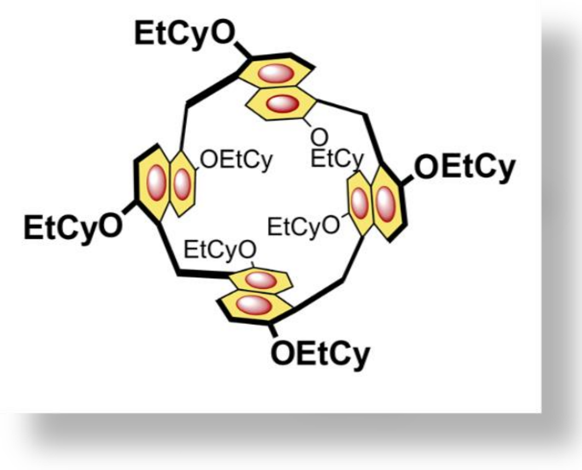

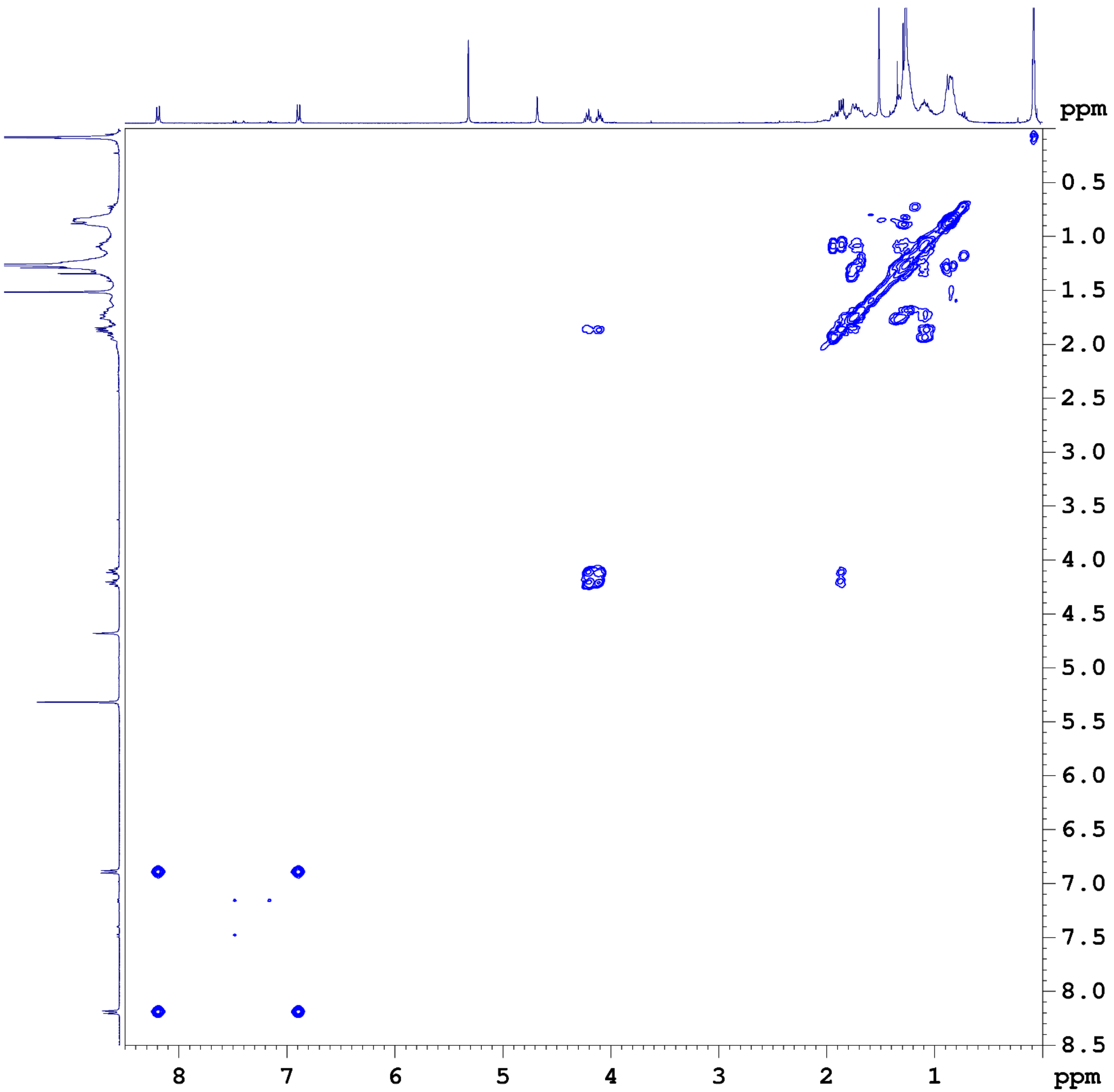

Figure S8: 2D-DQF COSY spectrum of **PrS[4]*EtCy*** (CD_2Cl_2, 600 MHz, 298 K).

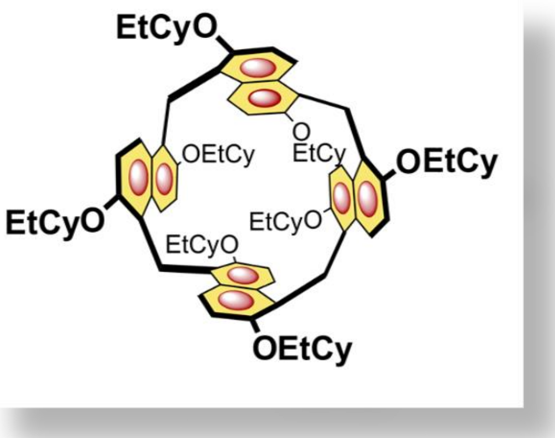

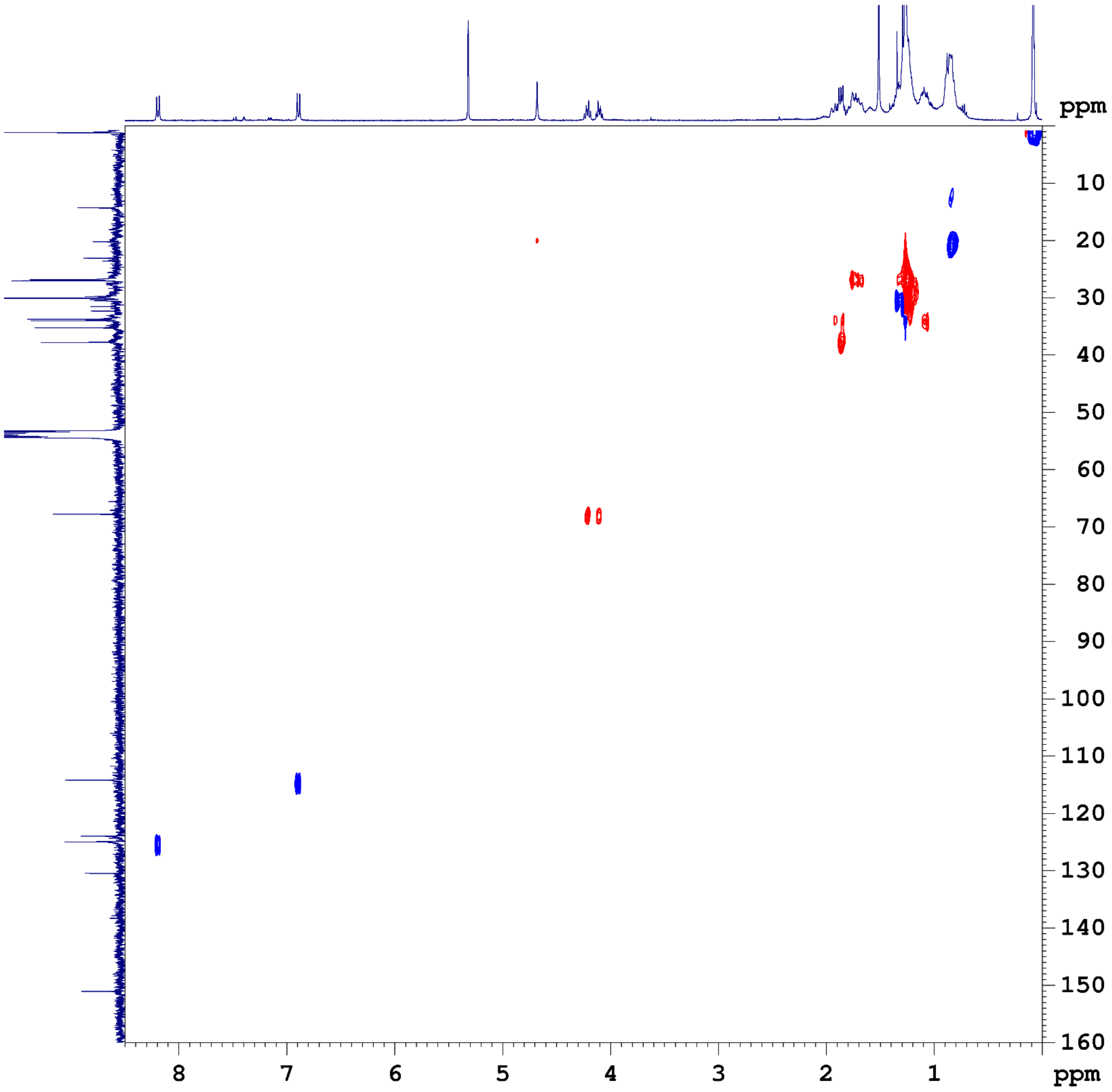

Figure S9: 2D-HSQC spectrum of **PrS[4]**^{EtCy} (CD_2Cl_2, 600 MHz, 298 K).

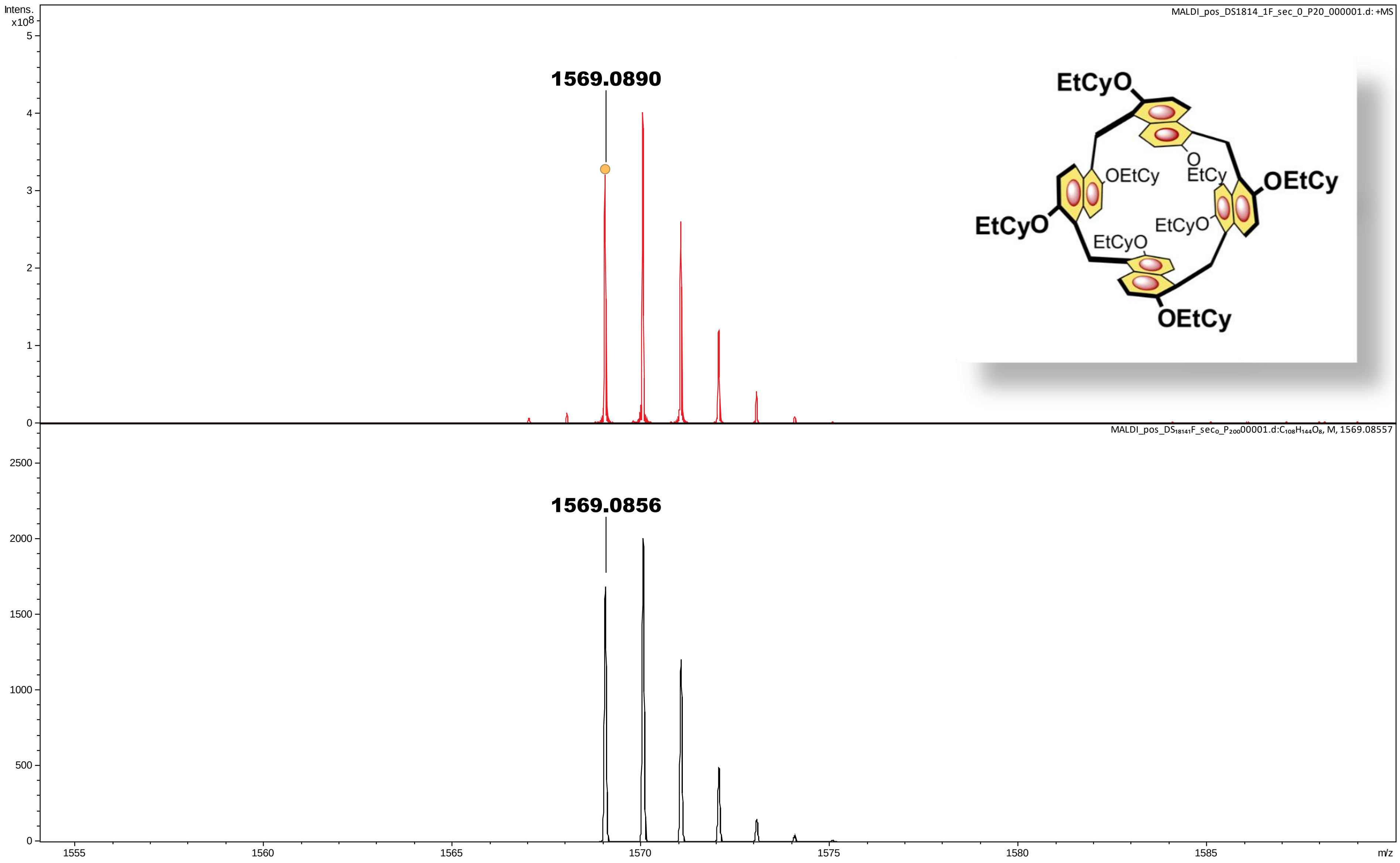

Figure S10: Comparison of experimental (top) and simulated (bottom) HR FT-ICR mass spectra of **PrS[4]**^{EtCy}.

1D and 2D NMR Studies on the Complexation of Prism[4]arene with Achiral Guests (**2^+** to **5^+**)

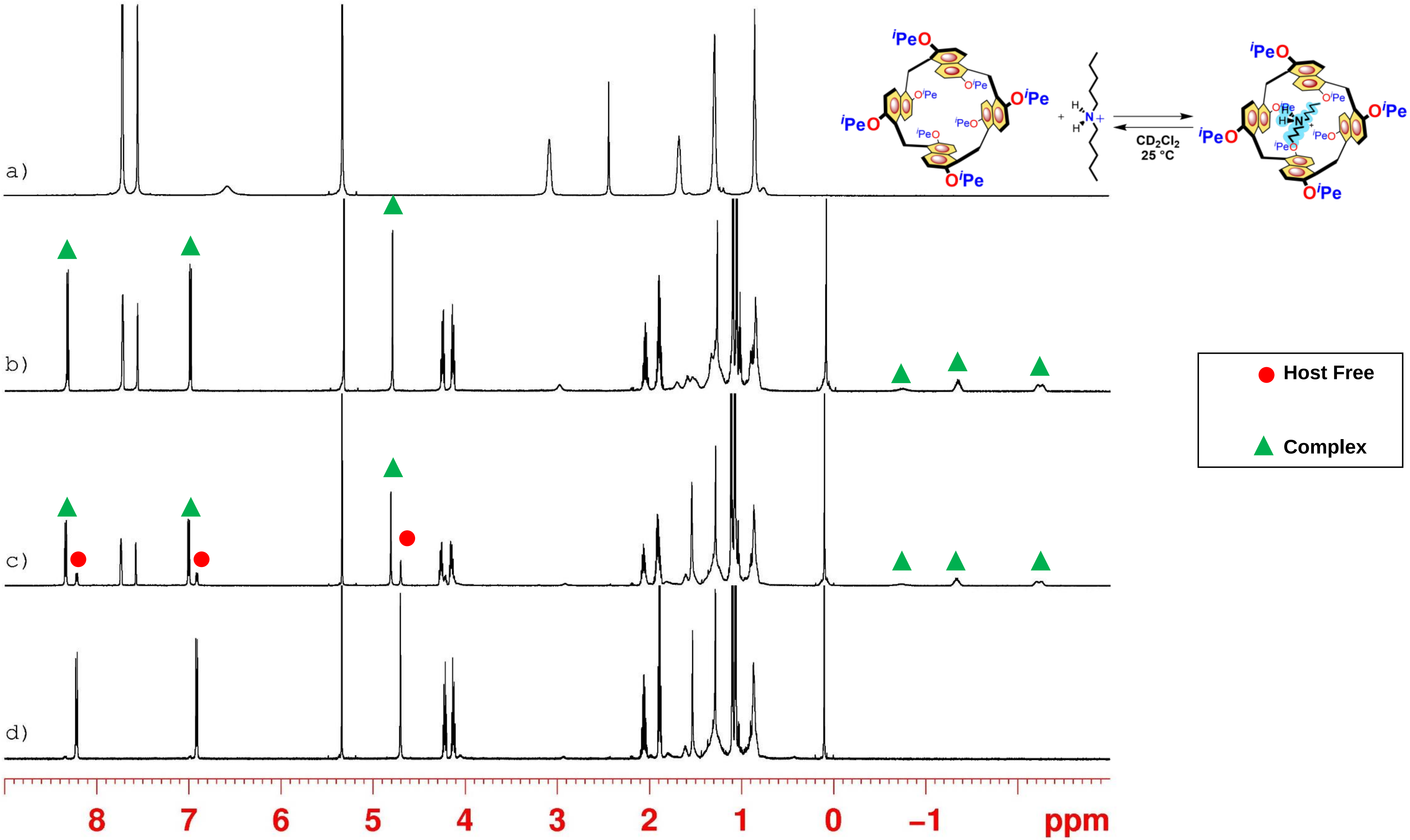

Figure S11: ^{1}H NMR spectra (600 MHz, CD_2Cl_2, 298 K) of: (a) **2^+·BArF**$^-$, (b) an equimolar solution (4.10 mM) of **PrS[4]**^{iPe} and **2^+·BArF**$^-$, (c) a 1:0.75 mixture of **PrS[4]**^{iPe}/**2^+·BArF**$^-$ and (d) **PrS[4]**^{iPe}.

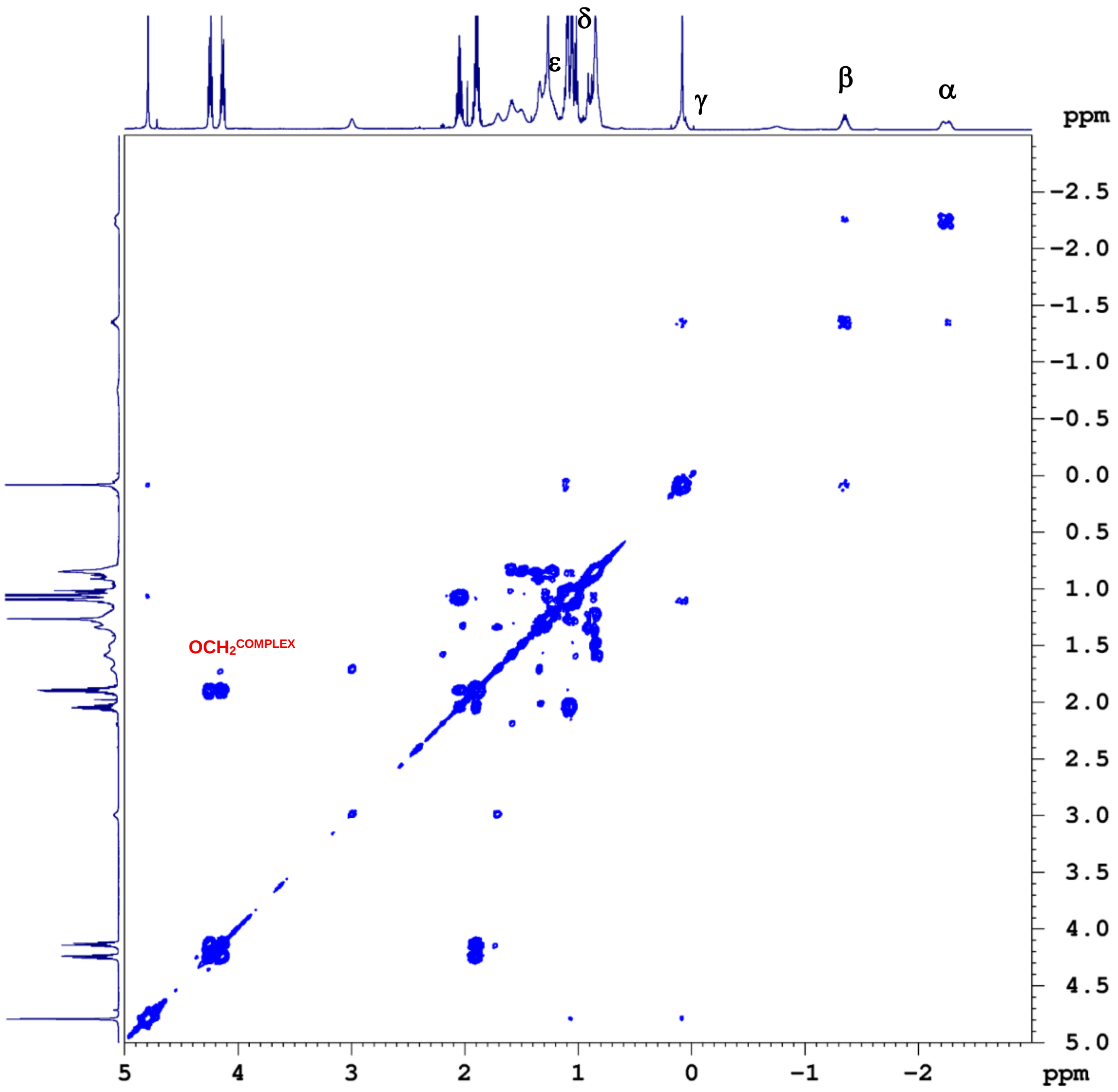

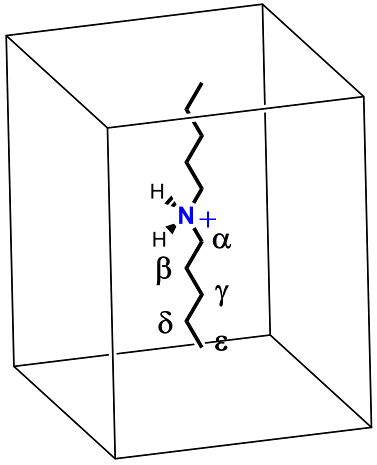

Figure S12: Portion of 2D-DQF COSY spectrum of **2⁺@PrS[4]*iPe*** (CD_2Cl_2, 600 MHz, 298 K).

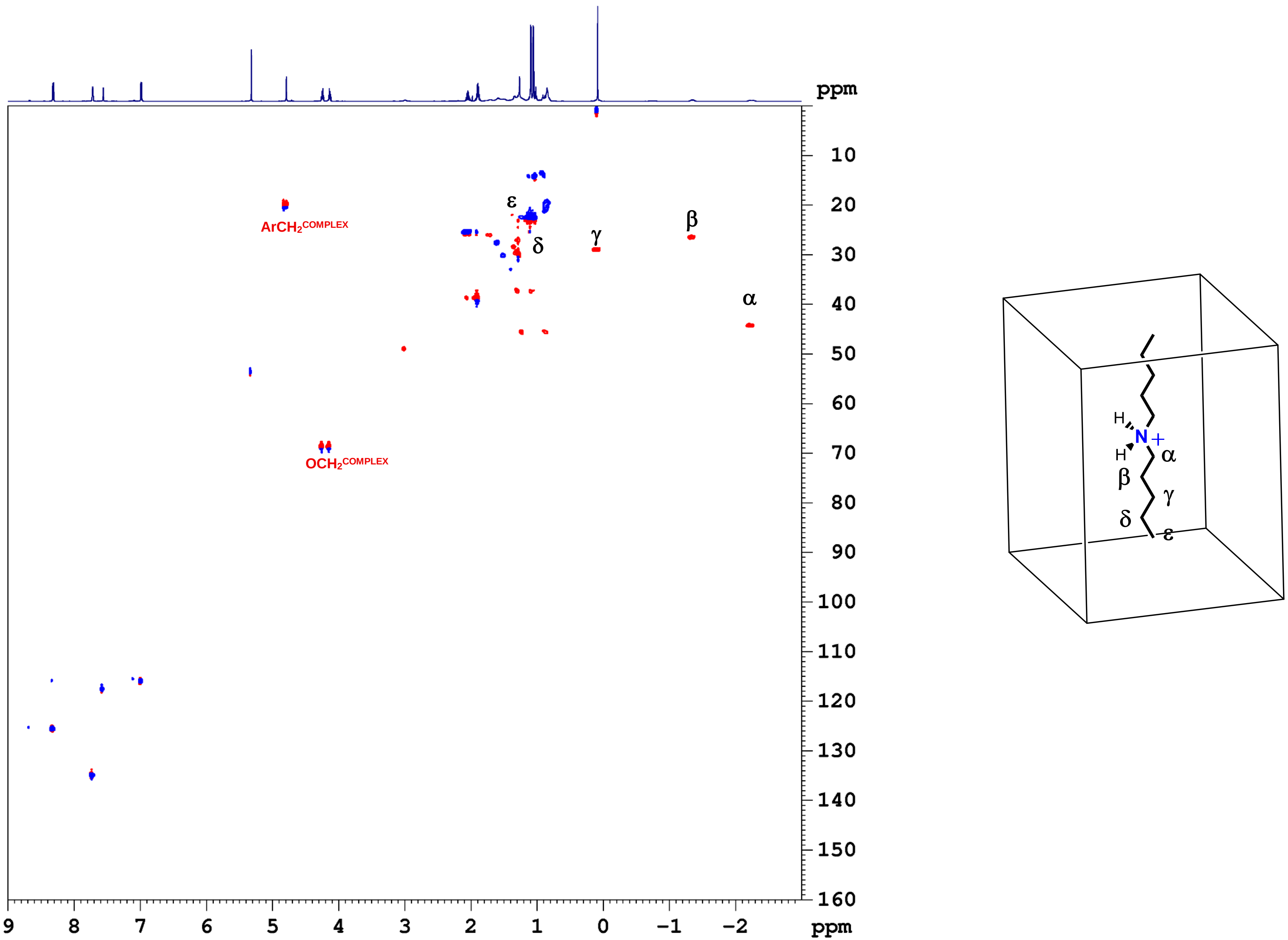

Figure S13: 2D-HSQC spectrum of **2⁺@PrS[4]*iPe*** (CD_2Cl_2, 600 MHz, 298 K).

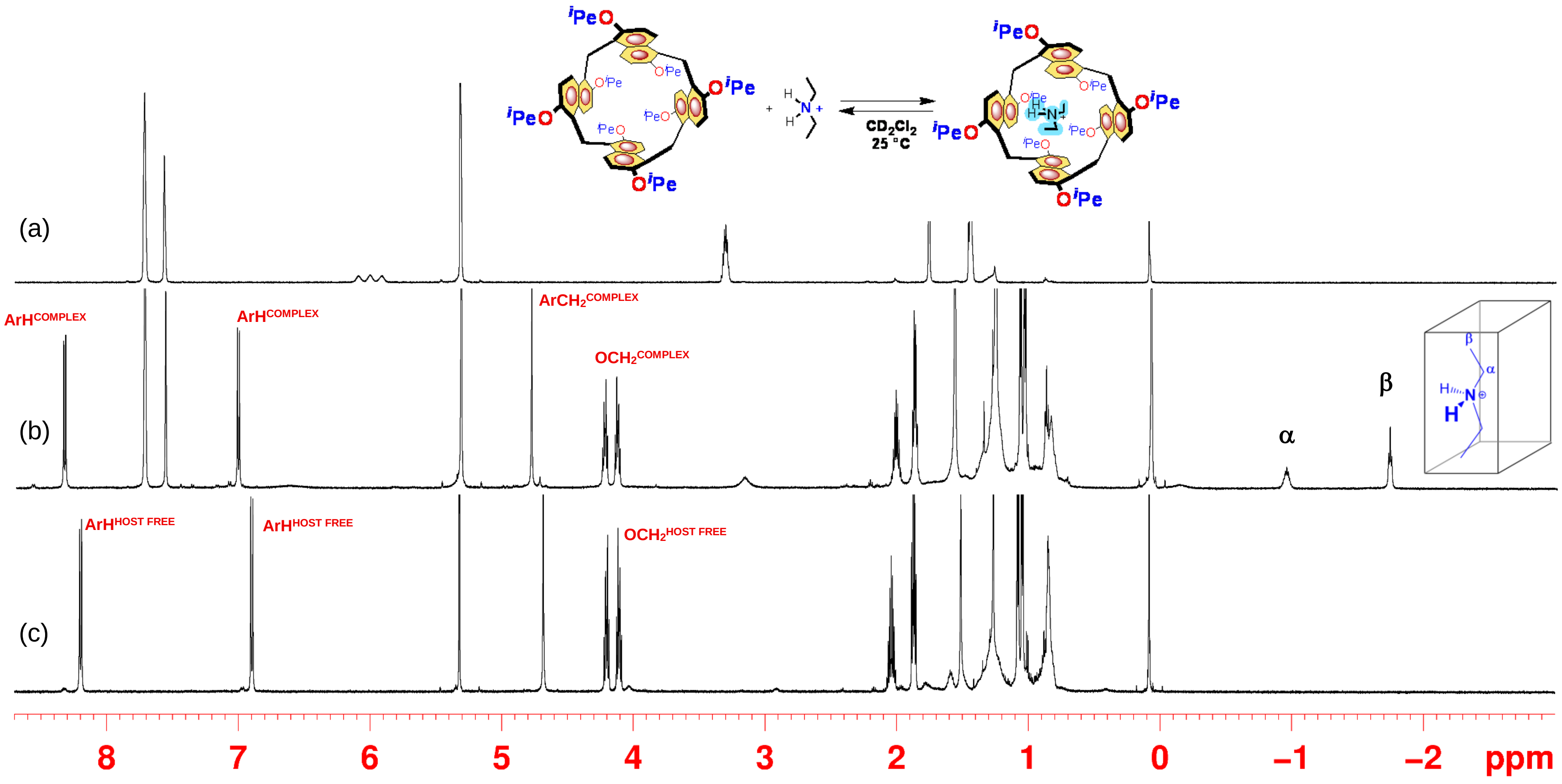

Figure S14: ^{1}H NMR spectra (600 MHz, CD_2Cl_2, 298 K) of: (a) $\mathbf{4^{+}}$**·BArF**$^{–}$, (b) an equimolar solution (4.10 mM) of **PrS[4]**^{iPe} and $\mathbf{4^{+}}$**·BArF**$^{–}$, and (c) **PrS[4]**^{iPe}.

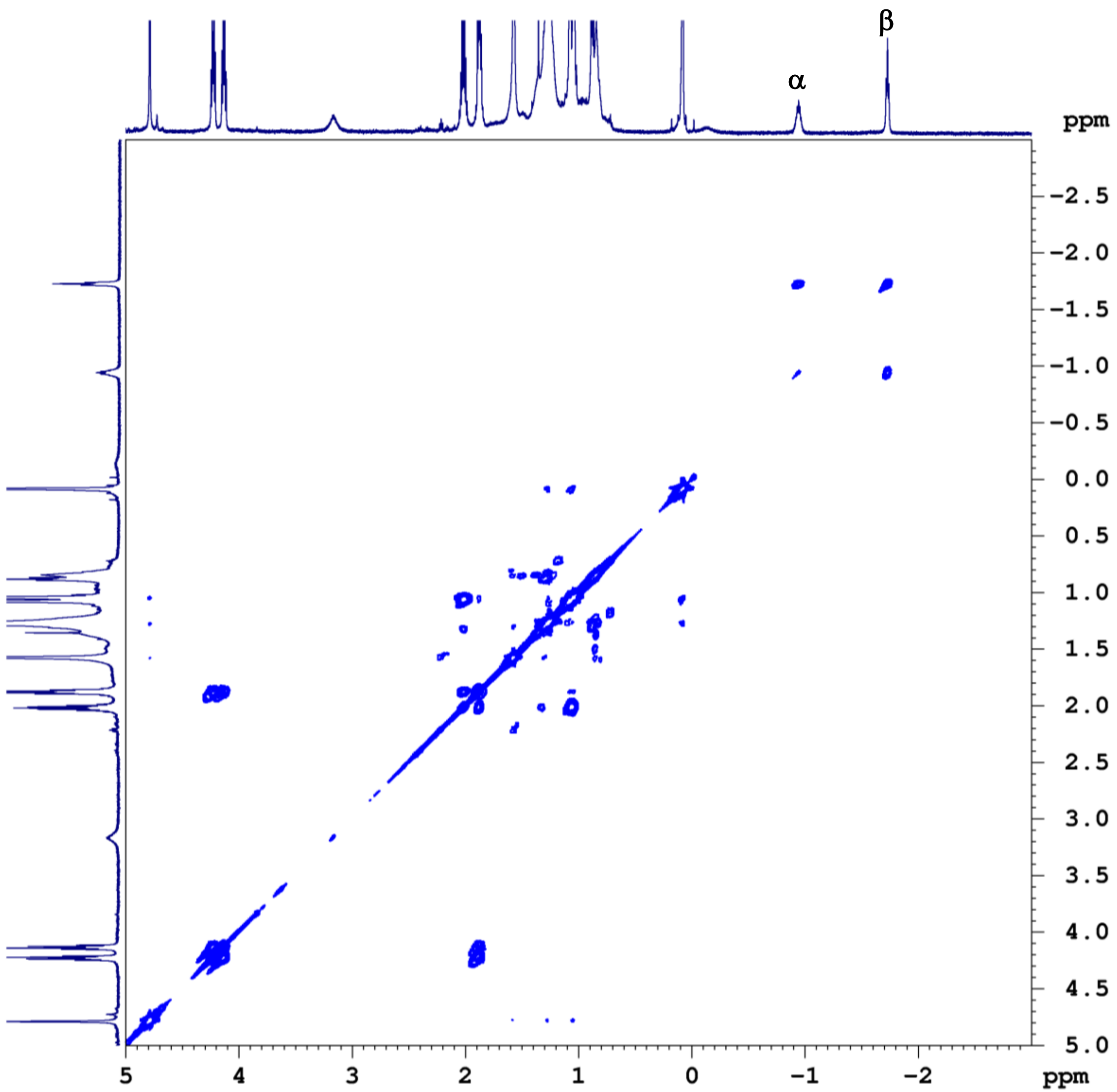

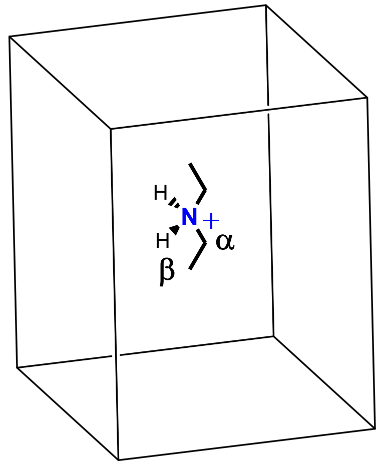

Figure S15: Portion of 2D-DQF COSY spectrum of **4**$^{+}$**@PrS[4]**^{iPe} (CD_2Cl_2, 600 MHz, 298 K).

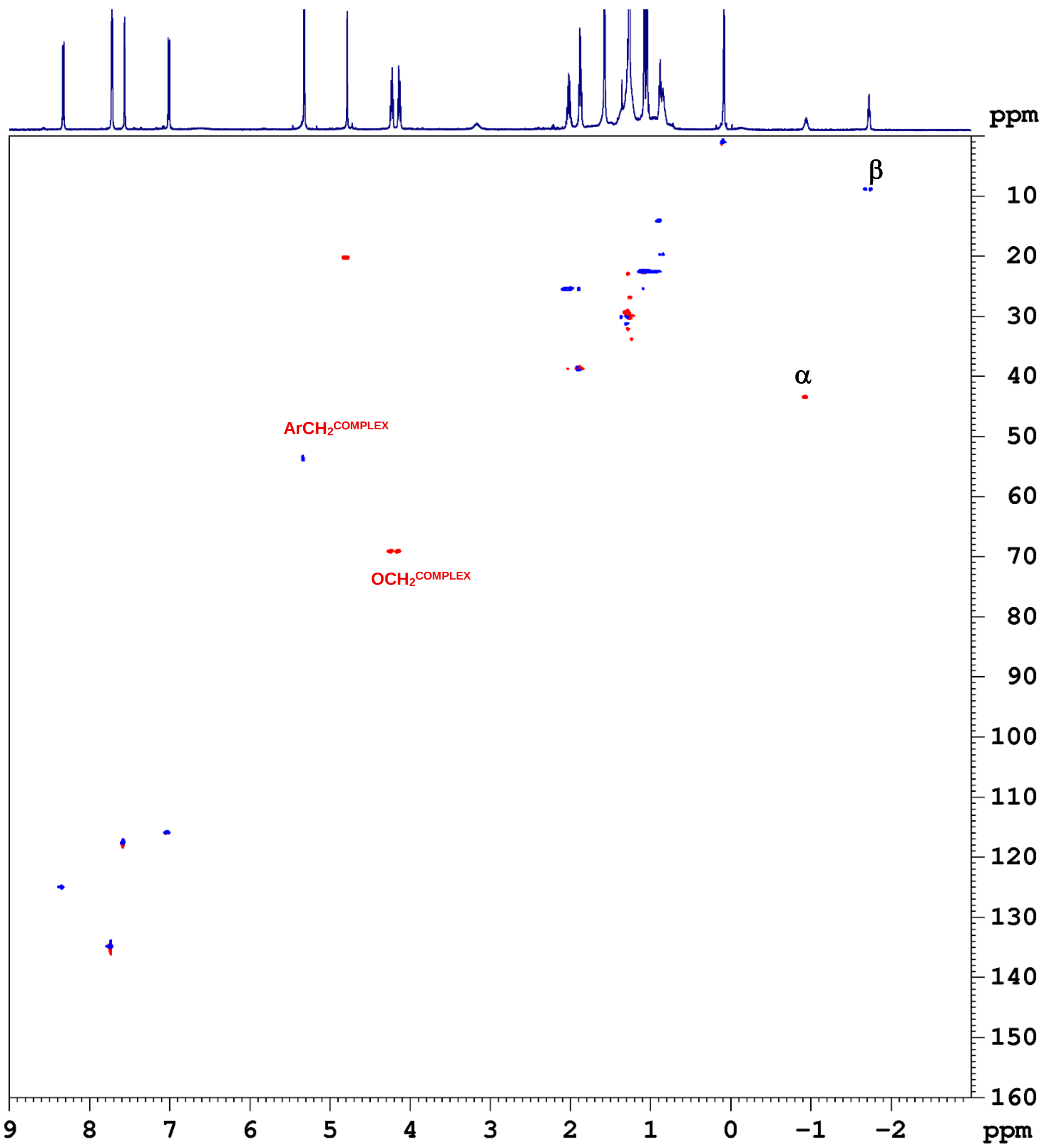

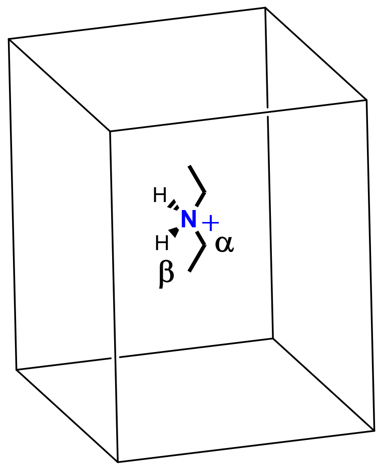

Figure S16: 2D-HSQC spectrum of **4⁺@PrS[4]***^{iPe}* (CD_2Cl_2, 600 MHz, 298 K).

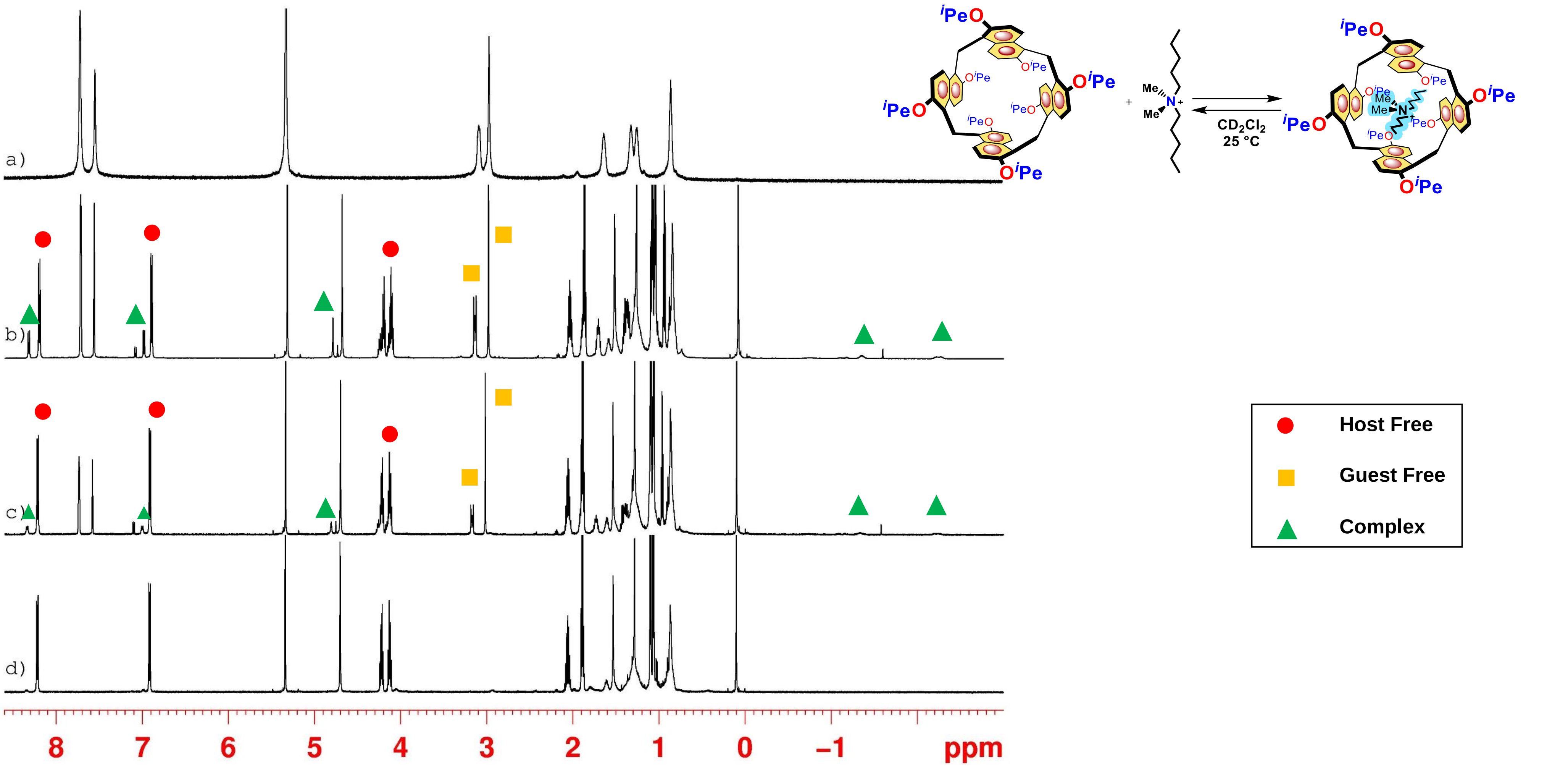

Figure S17: ^{1}H NMR spectra (600 MHz, CD_2Cl_2, 298 K) of: (a) **3^{+}·BArF**$^{–}$, (b) an equimolar solution (4.10 mM) of **PrS[4]^{iPe}** and **3^{+}·BArF**$^{–}$**,** (c) a 1 : 0.75 mixture of **PrS[4]^{iPe}/3^{+}·BArF**$^{–}$ and (d) **PrS[4]^{iPe}**.

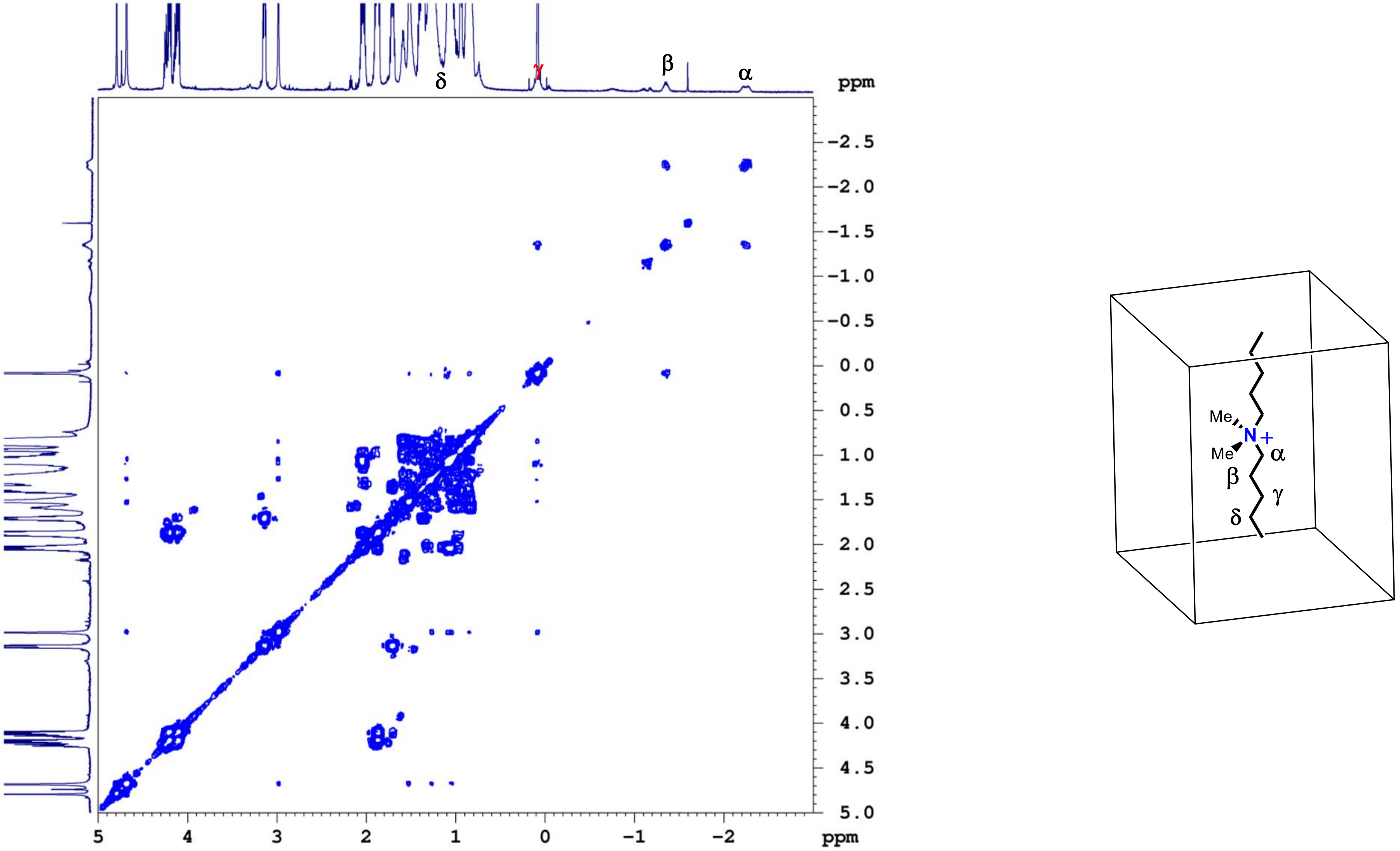

Figure S18: Portion of 2D-DQF COSY spectrum of **3⁺@PrS[4]*^iPe^*** (CD_2Cl_2, 600 MHz, 298 K).

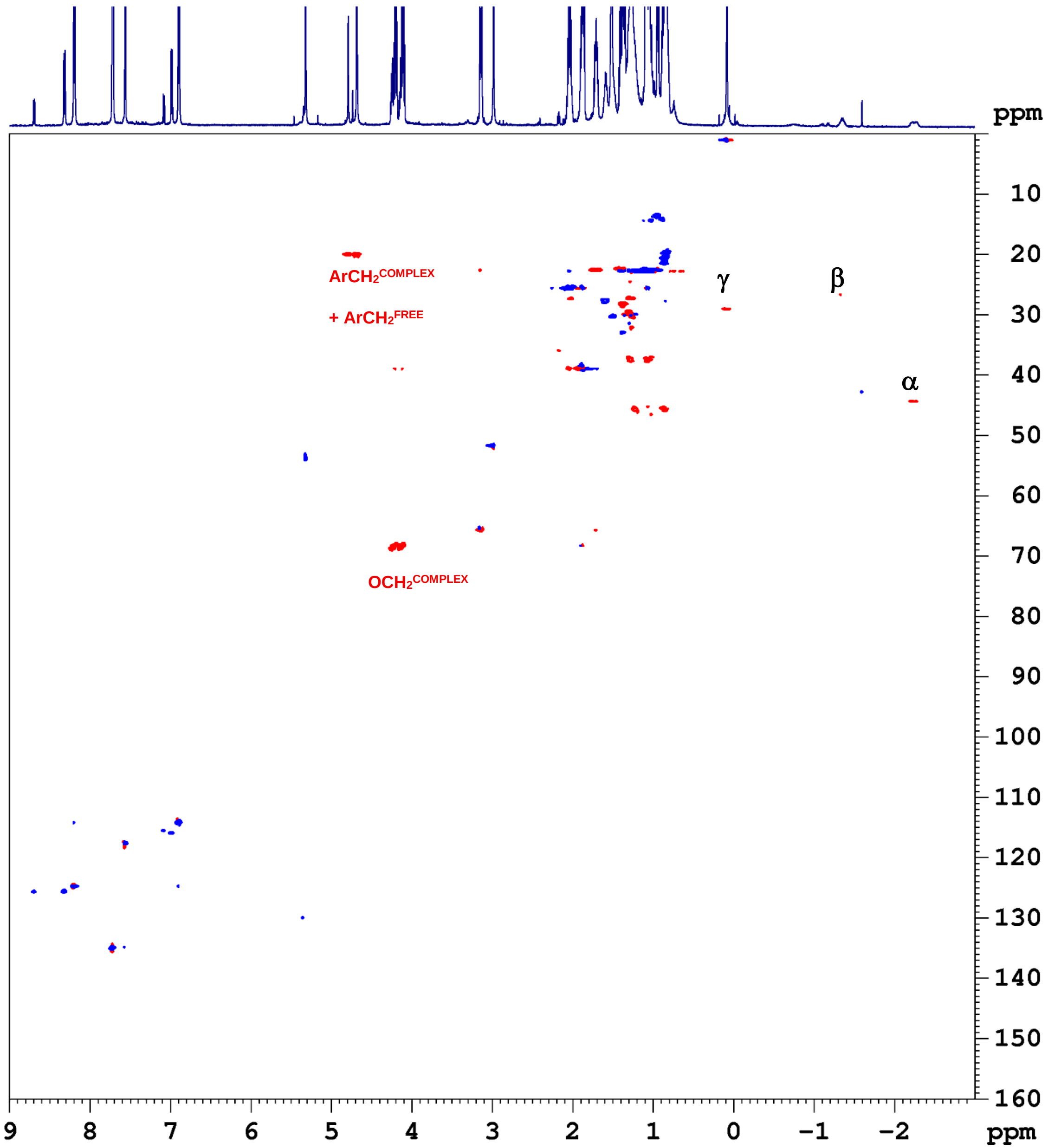

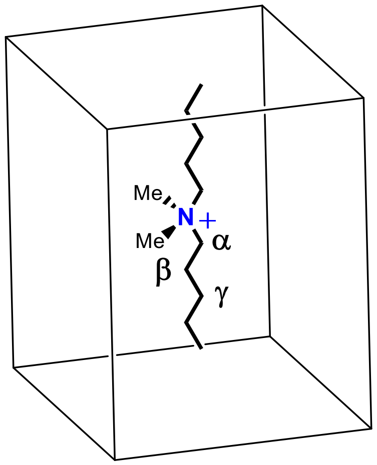

Figure S19: 2D-HSQC spectrum of $\mathbf{3^{+}@PrS[4]^{\mathit{iPe}}}$ (CD_2Cl_2, 600 MHz, 298 K).

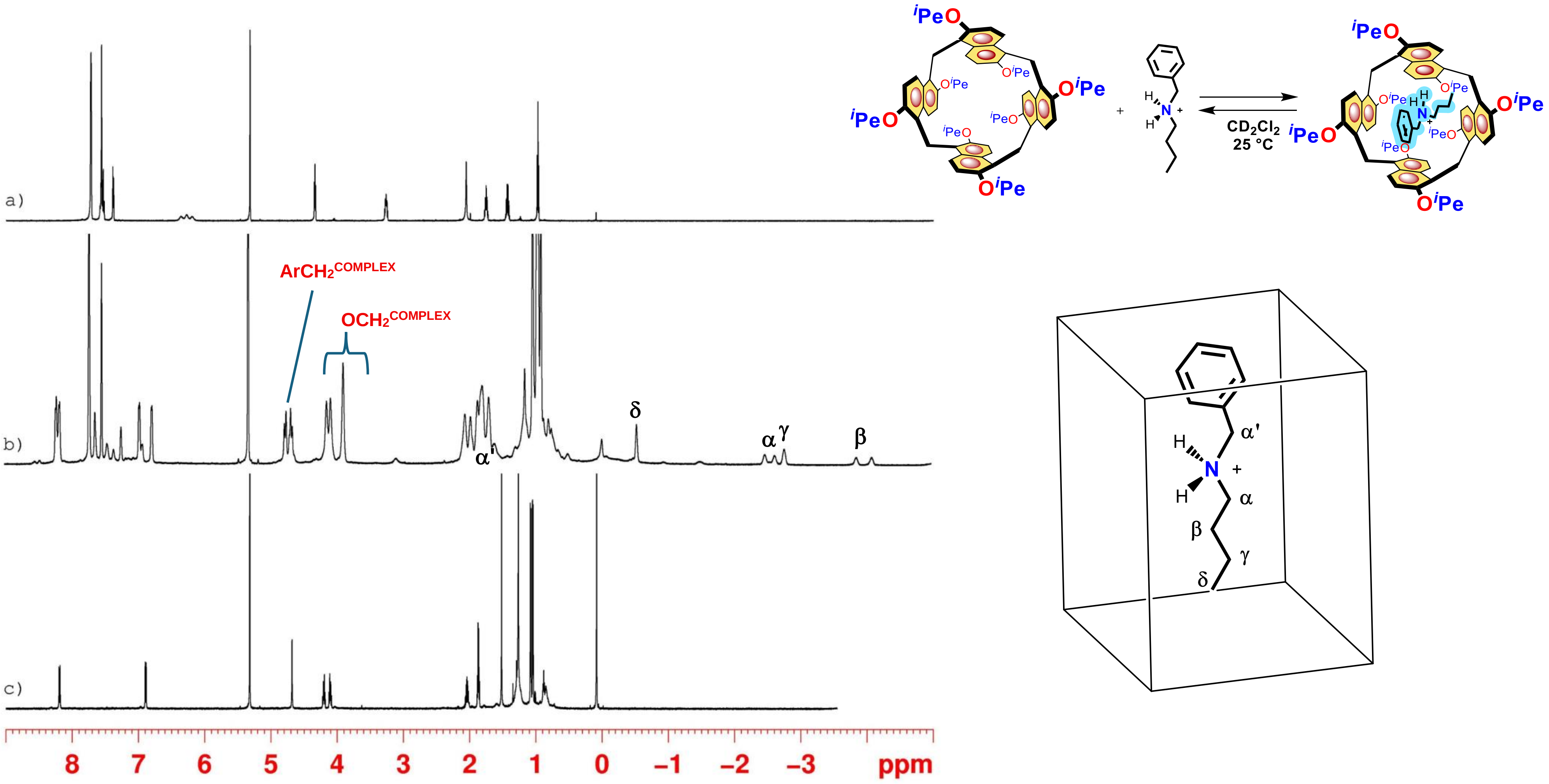

Figure S20: ^{1}H NMR spectra (600 MHz, CD_2Cl_2, 298 K) of: (a) **5^+·BArF$^-$**, (b) an equimolar solution (4.10 mM) of **PrS[4]*^{iPe}*** and **5^+·BArF$^-$,** (c) **PrS[4]*^{iPe}***.

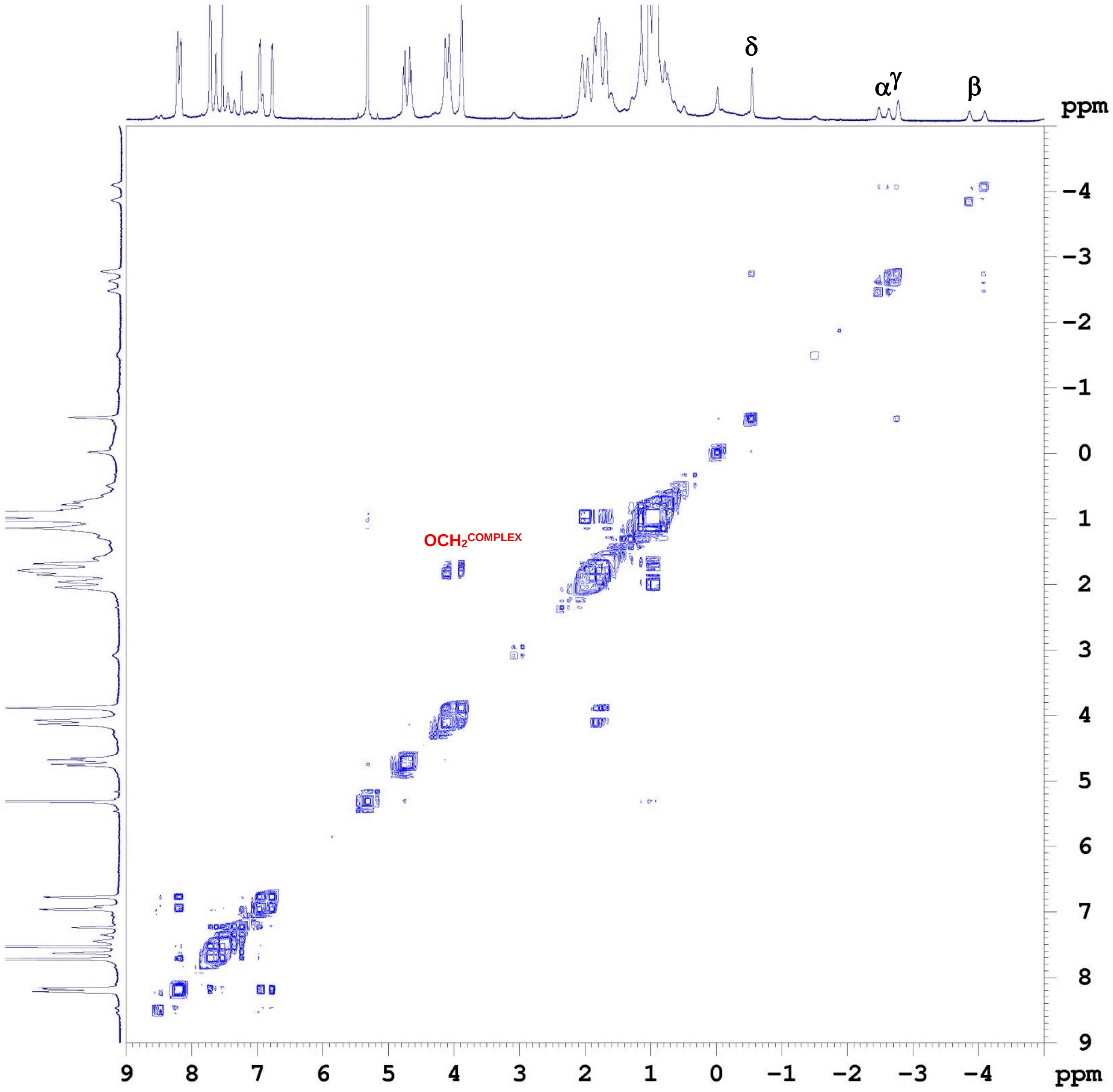

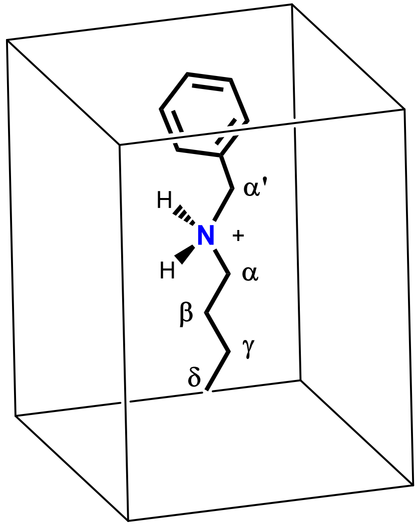

Figure S21: Portion of 2D-DQF COSY spectrum of **5⁺@PrS[4]*iPe*** (CD_2Cl_2, 600 MHz, 298 K).

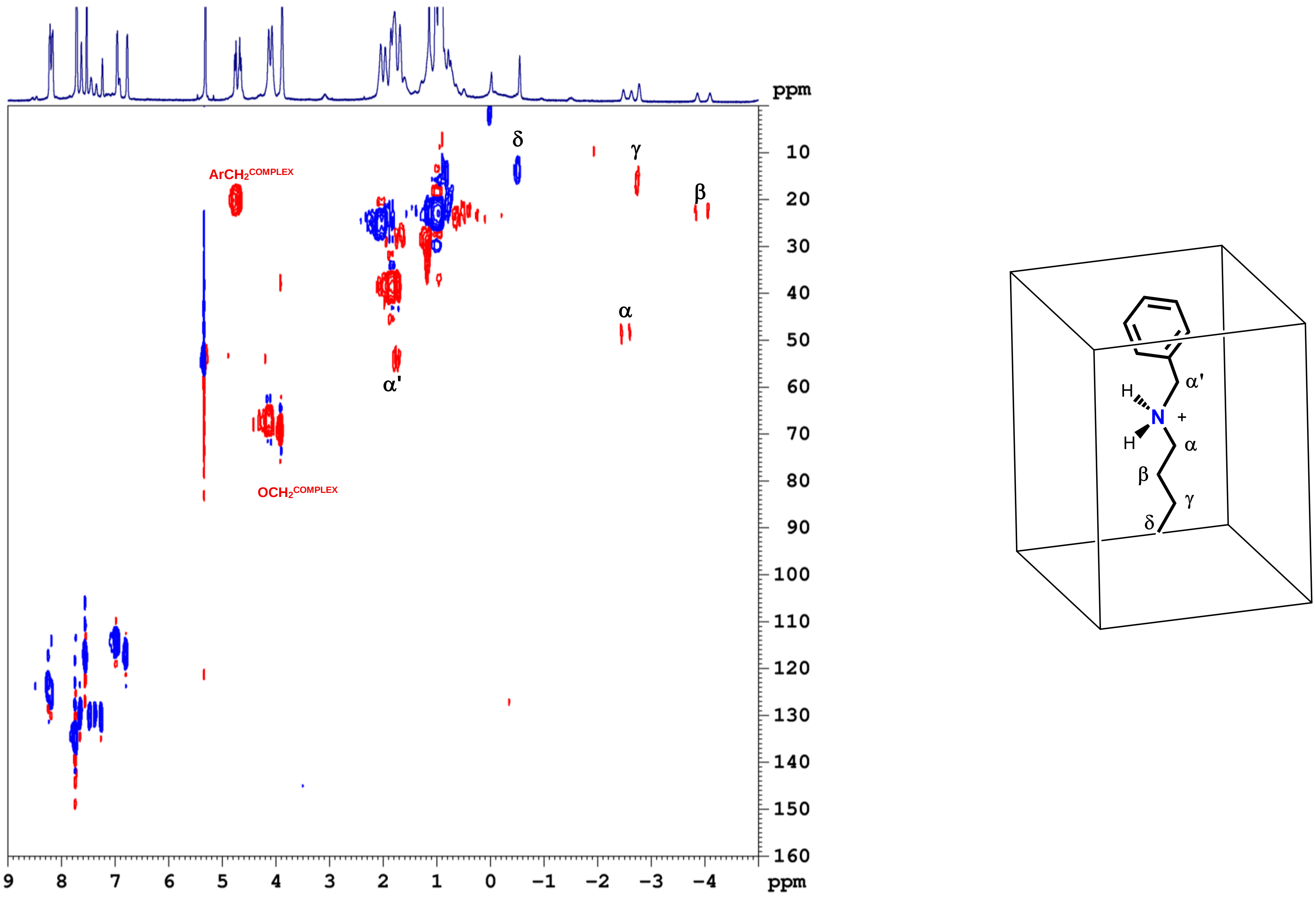

Figure S22: 2D-HSQC spectrum of **5⁺@PrS[4]**^{iPe} (CD_2Cl_2, 600 MHz, 298 K).

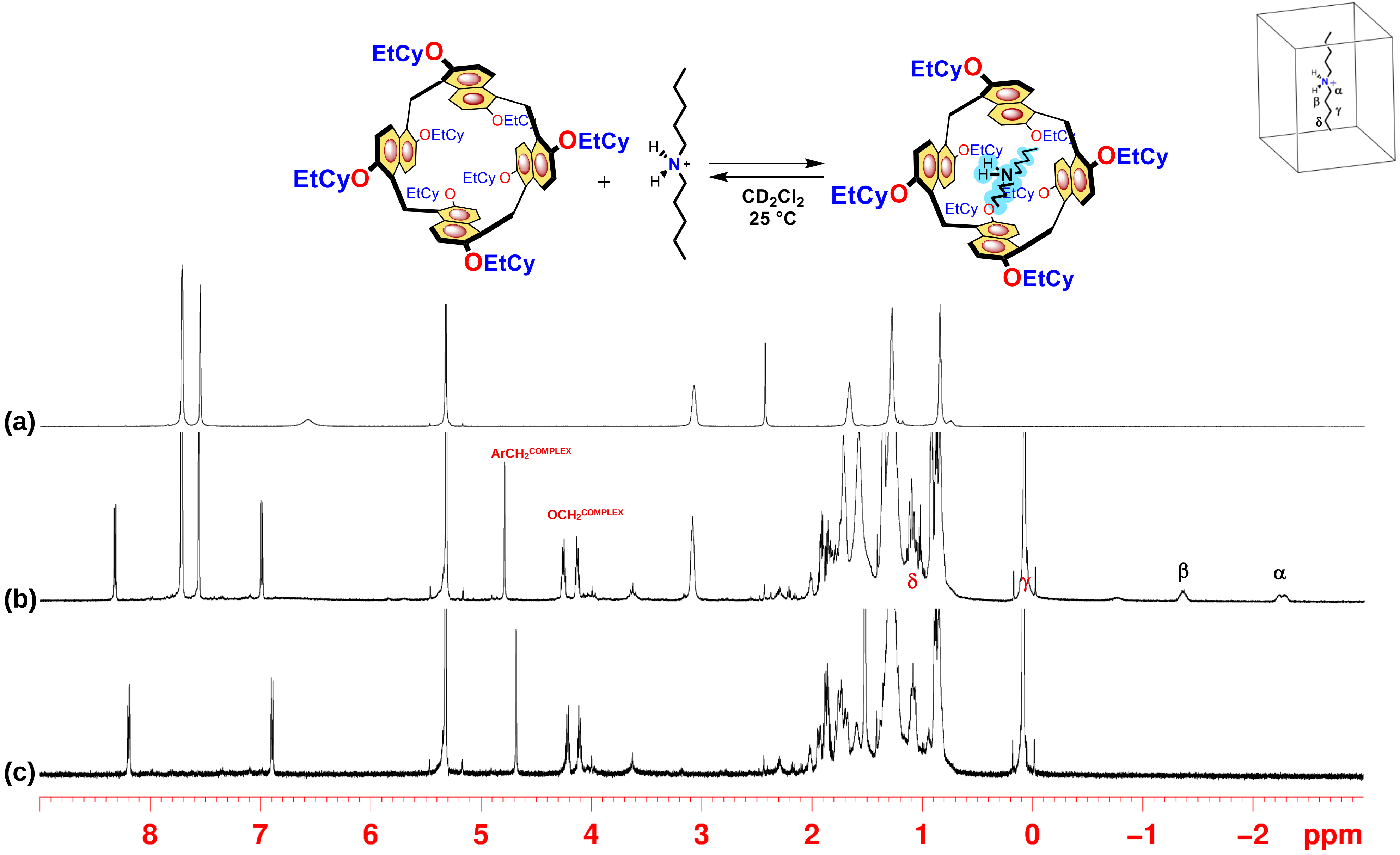

Figure S23: ^{1}H NMR spectra (600 MHz, CD_2Cl_2, 298 K) of: (a) **2^+·BArF$^-$**, (b) an equimolar solution of **PrS[4]^{EtCy}** and **2^+·BArF$^-$** and (c) **PrS[4]^{EtCy}**.

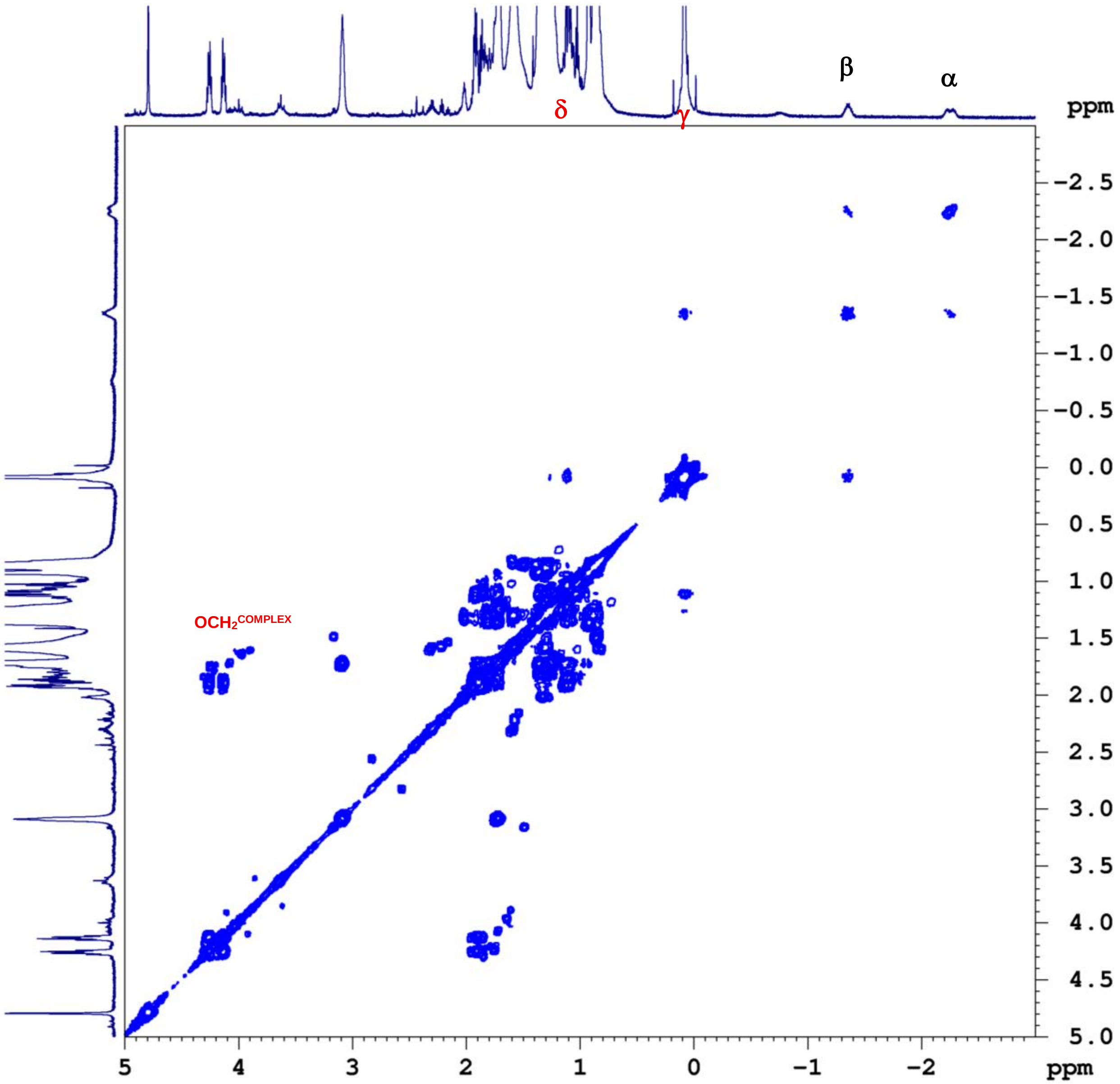

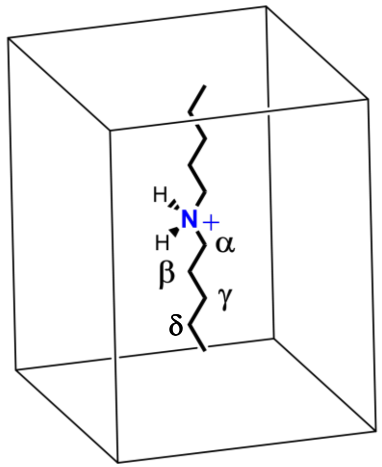

Figure S24: Portion of 2D-DQF COSY spectrum of **2**$^{+}$@ **PrS[4]**^{EtCy} (CD_2Cl_2, 600 MHz, 298 K).

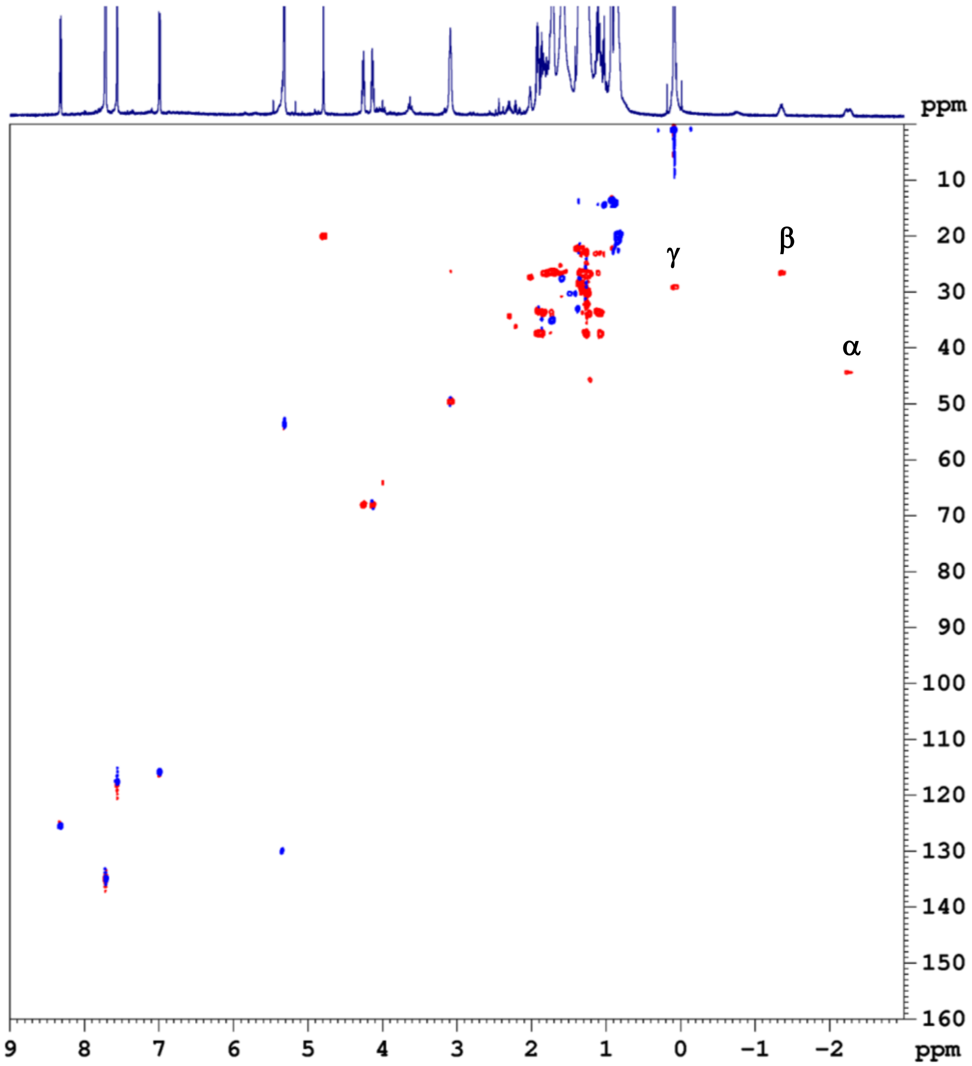

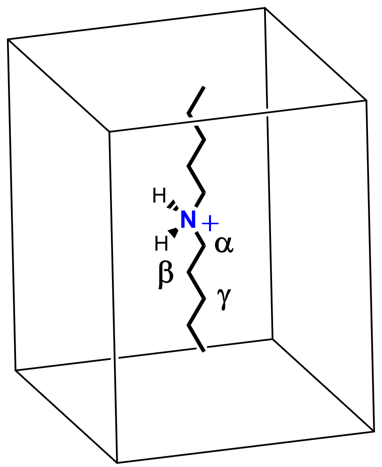

Figure S25: 2D-HSQC spectrum of **2**$^{+}$@ **PrS[4]**^{EtCy} (CD_2Cl_2, 600 MHz, 298 K).

Details on the Calculation of Association Constants for the Complexation of Prism[4]arene with Achiral Guests (**2**$^{+}$ to **5**$^{+}$)

Table S1: Association constant (K_{ass}, M^{-1}) values for the formation of the complexes between the guests $\mathbf{2^+}$ – $\mathbf{5^+}$ as barfate ($BArF^-$) salts and $\mathbf{PrS[4]^{R}}$, $\mathbf{PrS[5]^{Me}}$. Determined by ^1H NMR experiments in CD_2Cl_2. Errors < 15% calculated as mean values of three measures.

	$\mathbf{2^+ \cdot BArF^-}$ 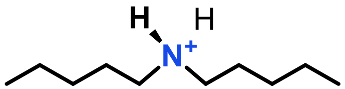	$\mathbf{3^+ \cdot BArF^-}$ 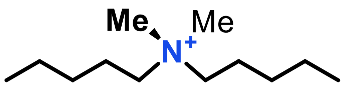	$\mathbf{4^+ \cdot BArF^-}$ 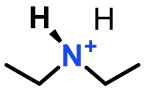	$\mathbf{5^+ \cdot BArF^-}$ 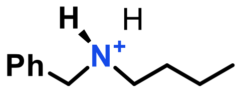
$\mathbf{PrS[4]^{iPe}}$	**45000** $^{(b)}$ – Fig. S29	**125** $^{(a)}$ - Fig. S31	**4200** $^{(b)}$ – Fig. S30	**5600** $^{(b)}$ – Fig. S32
$\mathbf{PrS[4]^{EtCy}}$	**1000** $^{(b)}$ – Fig. S33	---	---	---
$\mathbf{PrS[5]^{Me}}$	**8500** $^{(a)}$ – Fig. S26	---	**1800** $^{(a)}$ – Fig. S27	**4440** $^{(a)}$ – Fig. S28

a and **b** are the method used for the K_{ass} determination, see below.

The association constant values of complexes were calculated by means of two methods, **a** and **b**:[1]

(a) **Method A**: Integration of the ^1H NMR signals of both free and complexed host in an equimolar solution of hosts and guests solubilized in CD_2Cl_2 (please refer to the experimental details provided in the captions for Figures S26-S28 and S31). This method was used for the experiment in Figure S31 to calculate the association constant for the formation of the complex $\mathbf{3^+@PrS[4]^{iPe}}$. In all other cases, the signals of the free host were not visible; thus, we used method **B**, which involves NMR competition experiments. In this case, we employed $\mathbf{PrS[5]^{Me}}$ as the competitive host. However, we first calculated the binding constants for the formation of the complexes between guests $\mathbf{2^+}$, $\mathbf{4^+}$, and $\mathbf{5^+}$ with $\mathbf{PrS[5]^{Me}}$ using method **A** (Figures S26-S28). The obtained values were then used as references for method **B**, as presented in Figures S29, S30, S32, and S33.

$$K_{ass} = \frac{\left\{\dfrac{integral\ complex}{integral\ complex + integral\ free} \cdot [Guest]\right\}}{\left\{\dfrac{integral\ free}{integral\ complex + integral\ free} \cdot [Guest]\right\}^2}$$

(b) **Method B:** ^1H NMR competition experiments. In this case, an analysis was performed of a 1:1:1 mixture of $\mathbf{PrS[4]^{R}}$, $\mathbf{PrS[5]^{Me}}$ and guests in an NMR tube using CD_2Cl_2 as solvent (please refer to the experimental details provided in the captions for Figures S29, S30, S32 and S33). The integrals were normalized to the number of protons.

$$\mathrm{K_{rel}} = \frac{Kass_A}{Kass_B}\ ; \qquad \mathrm{K_{rel}} = \frac{[H_AG]^2}{[H_BG]^2} = \frac{\left\{\dfrac{integral\ A}{integral\ A + integral\ B} \cdot [Guest]\right\}^2}{\left\{\dfrac{integral\ B}{integral\ A + integral\ B} \cdot [Guest]\right\}^2}$$

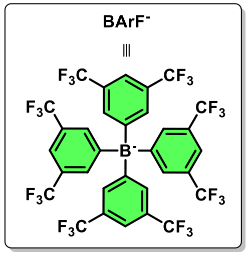

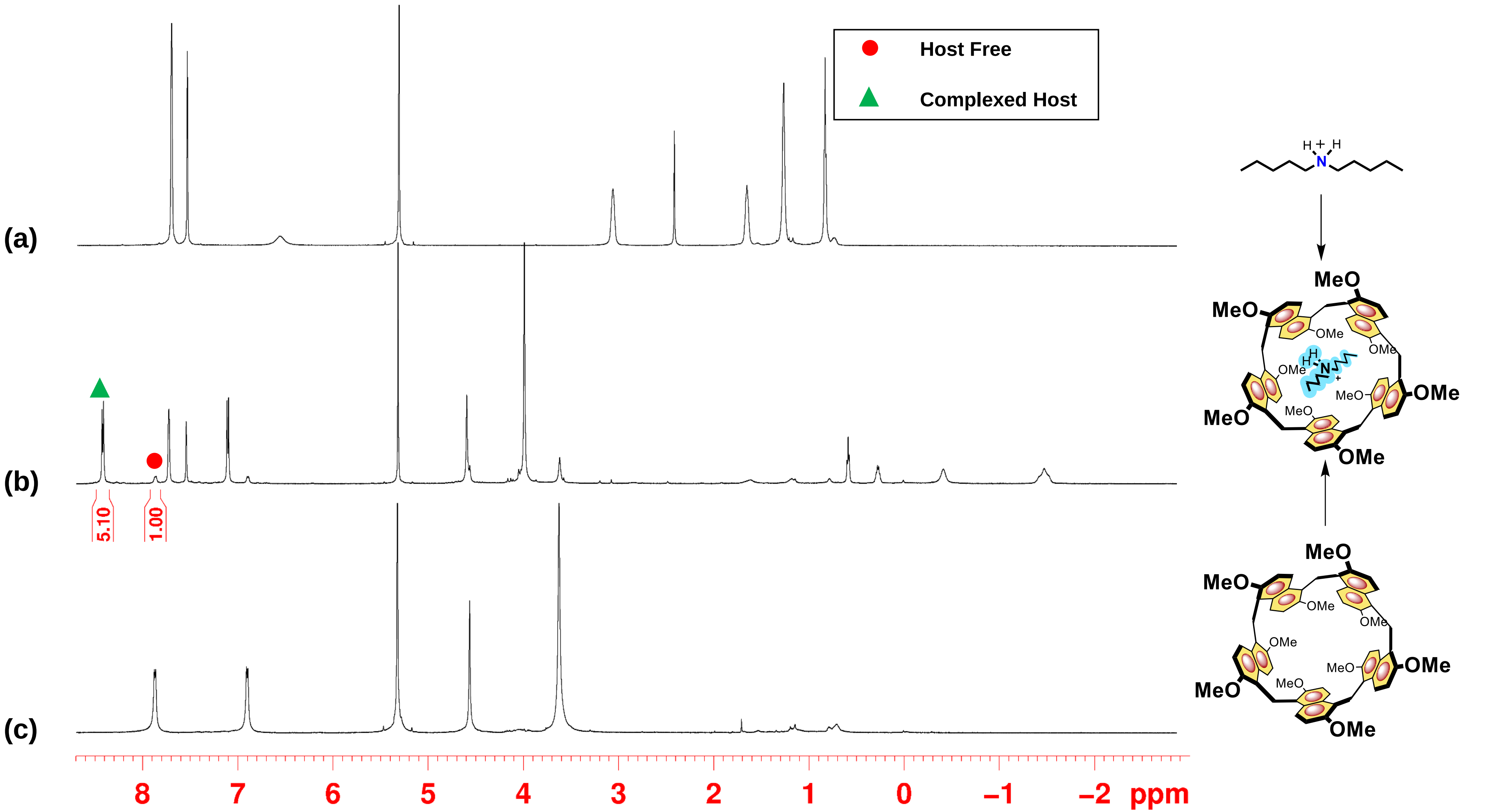

Figure S26: ^{1}H NMR spectra (600 MHz, CD_2Cl_2,193 K) of: (a) a solution of **2^{+}·BArF^{-}** (b) an equimolar solution (4.10 mM) of **PrS[5]^{Me}** and **2^{+}·BArF^{-}** in 0.5 mL of CD_2Cl_2 and (c) **PrS[5]^{Me}**.

$$K_{ass} = \frac{\left\{\frac{5.10}{6.10} \cdot 4.10 \cdot 10^{-3} M\right\}}{\left\{\frac{1.00}{6.10} \cdot 4.10 \cdot 10^{-3} M\right\}^{2}} = \mathbf{8500\ M^{-1}}$$

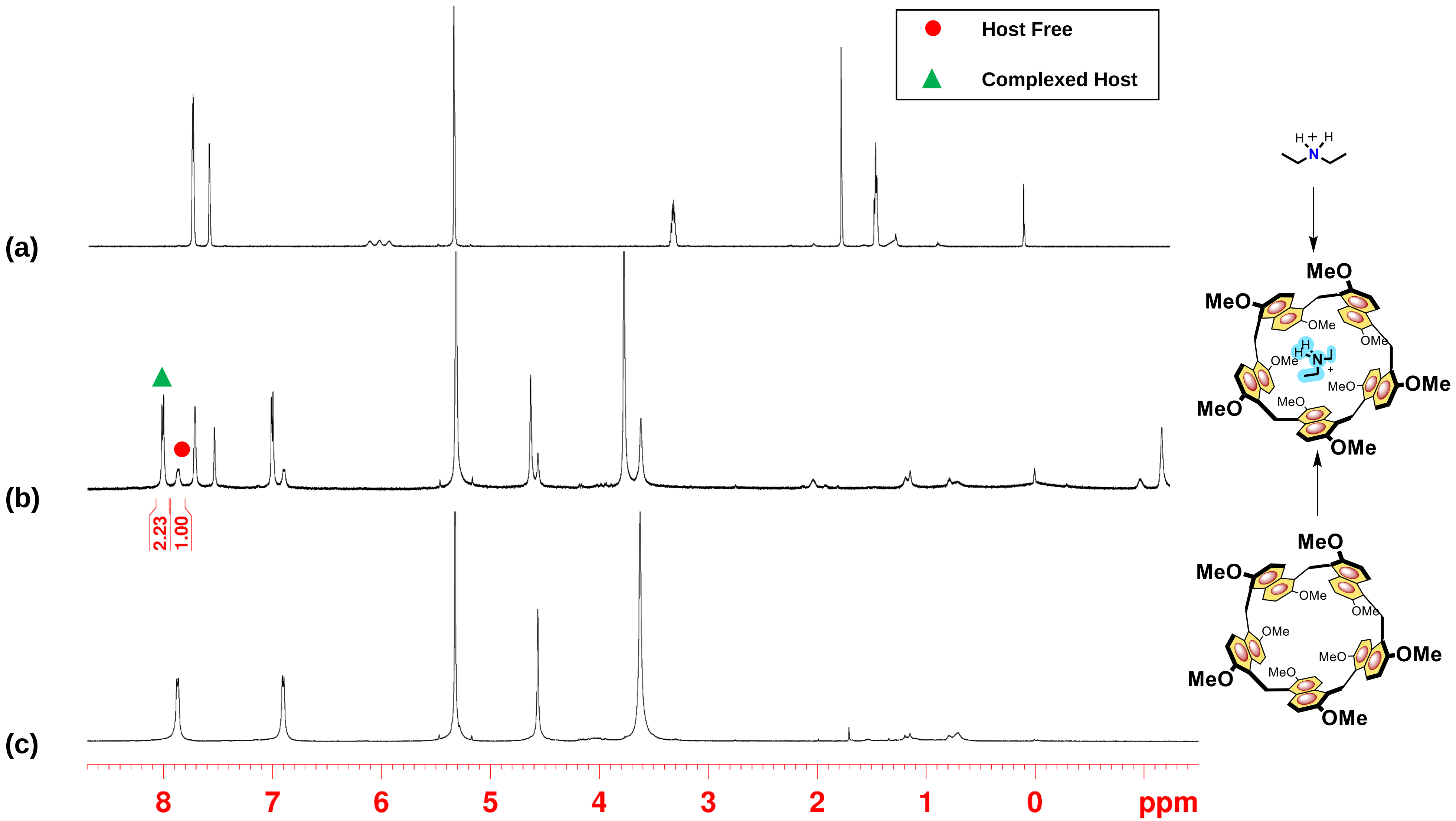

Figure S27: ^{1}H NMR spectra (600 MHz, CD_2Cl_2,193 K) of: (a) a solution of $\mathbf{4^{+}{\cdot}BArF^{-}}$(b) an equimolar solution (4.10 mM) of **PrS[5]*Me*** and $\mathbf{4^{+}{\cdot}BArF^{-}}$ in 0.5 mL of CD_2Cl_2 and (c) **PrS[5]*Me***.

$$K_{ass} = \frac{\left\{\frac{2.23}{3.23} \cdot 4.10 \cdot 10^{-3}M\right\}}{\left\{\frac{1.00}{3.23} \cdot 4.10 \cdot 10^{-3}M\right\}^2} = \mathbf{1800\ M^{-1}}$$

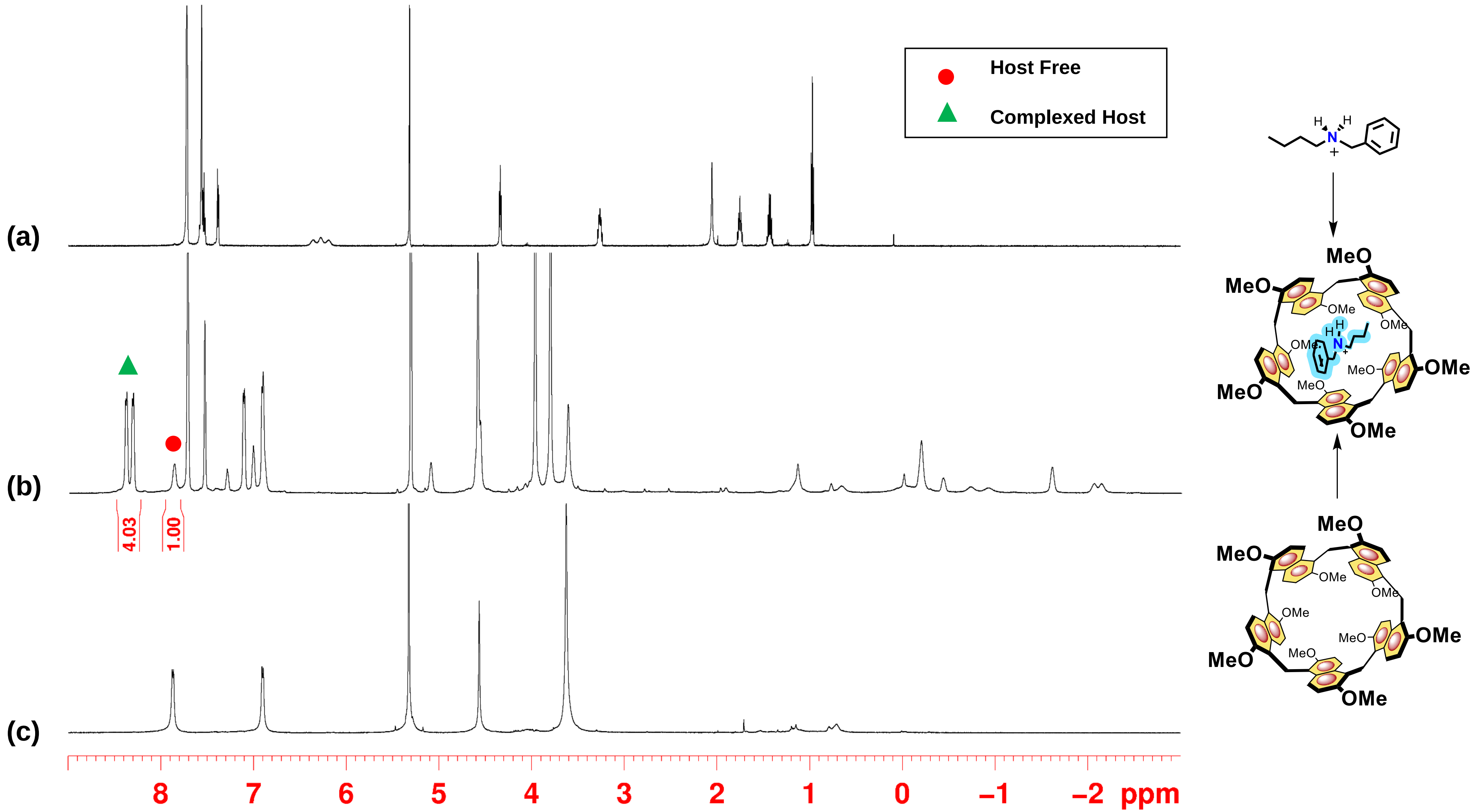

Figure S28: ^{1}H NMR spectra (600 MHz, CD_2Cl_2,193 K) of: (a) a solution of **5^+·BArF$^-$** (b) an equimolar solution (4.56 mM) of **PrS[5]^{Me}** and **5^+·BArF$^-$** in 0.5 mL of CD_2Cl_2 and (c) **PrS[5]^{Me}**.

$$K_{ass} = \frac{\left\{\frac{4.03}{5.03} \cdot 4.56 \cdot 10^{-3} M\right\}}{\left\{\frac{1.00}{5.03} \cdot 4.56 \cdot 10^{-3} M\right\}^2} = \mathbf{4440\ M^{-1}}$$

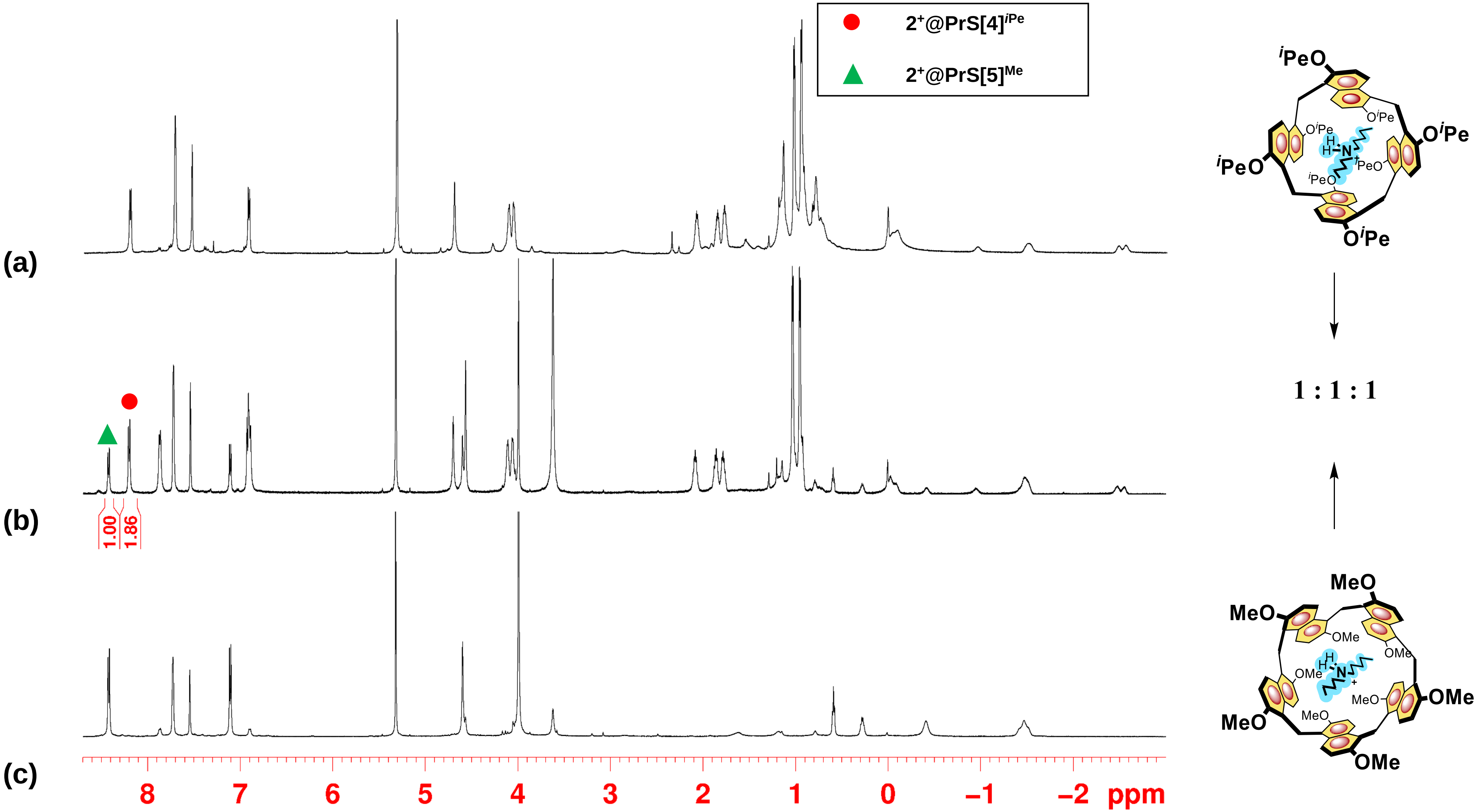

Figure S29: ^{1}H NMR spectra (600 MHz, CD_2Cl_2, 193 K) of: (a) an equimolar solution (2.67 mM) of **PrS[4]^{iPe}** and **2^{+}·BArF^{-}** in 0.5 mL of CD_2Cl_2, (b) **PrS[4]^{iPe}** in the presence of 1 equivalent of **PrS[5]^{Me}** and 1 equivalent of **2^{+}·BArF^{-}** and (c) an equimolar solution (2.67 mM) of **PrS[5]^{Me}** and **2^{+}·BArF^{-}** in 0.5 mL of CD_2Cl_2.

$$\mathrm{K_{rel}} = \frac{Kass_A}{8500\ M^{-1}} = \frac{\left\{\frac{0.23}{0.33}\cdot 2.67\cdot 10^{-3}M\right\}^2}{\left\{\frac{0.10}{0.33}\cdot 2.67\cdot 10^{-3}M\right\}^2} = \frac{3.46\cdot 10^{-6}}{6.55\cdot 10^{-7}} = 5.3;\ \ K_{ass} = \mathbf{45000\ M^{-1}}$$

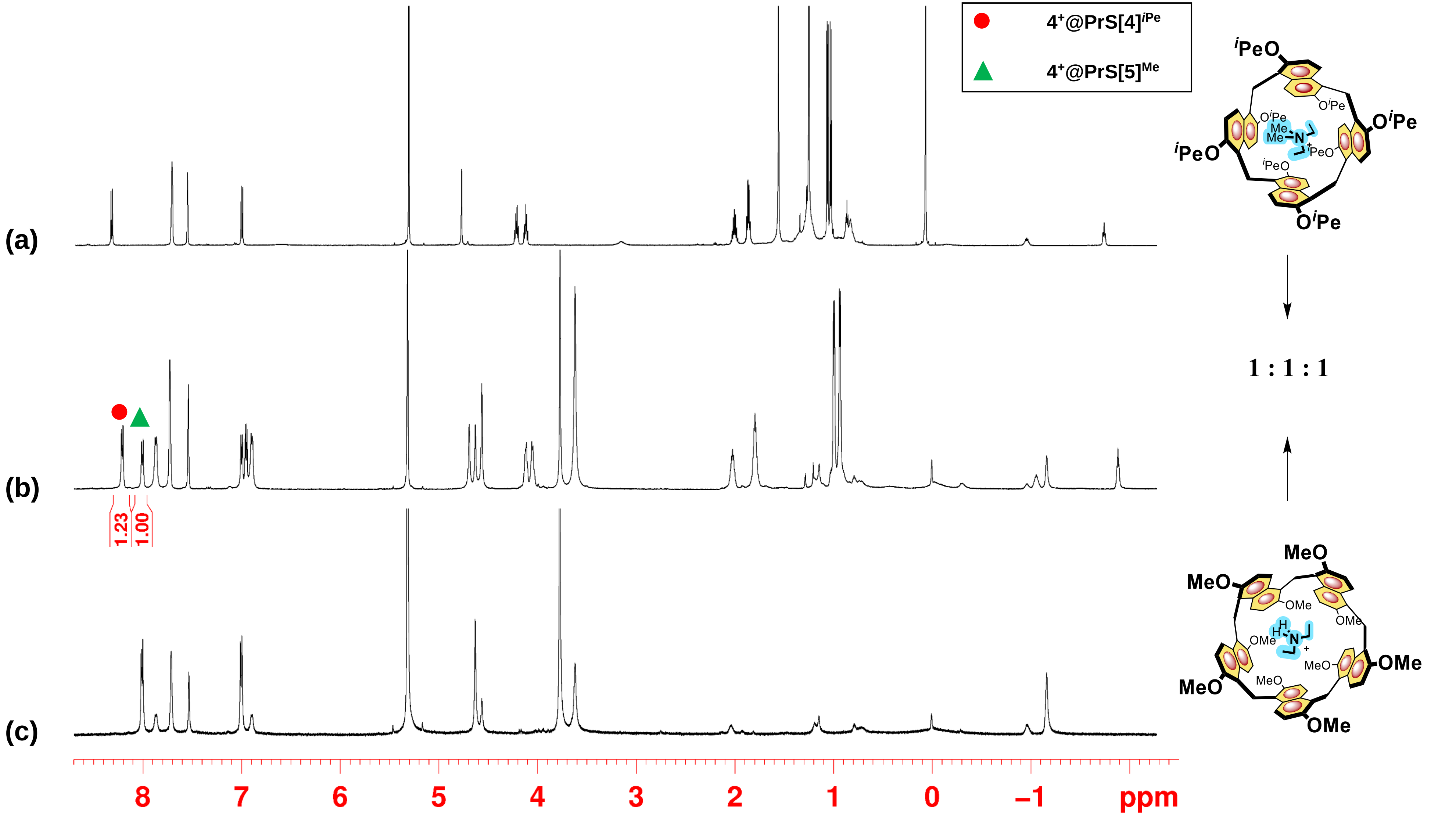

Figure S30: ^{1}H NMR spectra (600 MHz, CD_2Cl_2, 193 K) of: (a) an equimolar solution (2.67 mM) of **PrS[4]^{iPe}** and **4^{+}·BArF^{-}** in 0.5 mL of CD_2Cl_2, (b) **PrS[4]^{iPe}** in the presence of 1 equivalent of **PrS[5]^{Me}** and 1 equivalent of **4^{+}·BArF^{-}** and (c) an equimolar solution (2.67 mM) of **PrS[5]^{Me}** and **4^{+}·BArF^{-}** in 0.5 mL of CD_2Cl_2.

$$K_{rel} = \frac{Kass_A}{1800\ M^{-1}} = \frac{\left\{\frac{0.15}{0.25}\cdot 2.67\cdot 10^{-3} M\right\}^2}{\left\{\frac{0.10}{0.25}\cdot 2.67\cdot 10^{-3} M\right\}^2} = \frac{2.57\cdot 10^{-6}}{1.14\cdot 10^{-6}} = 2.3;\ \ K_{ass} = \mathbf{4200\ M^{-1}}$$

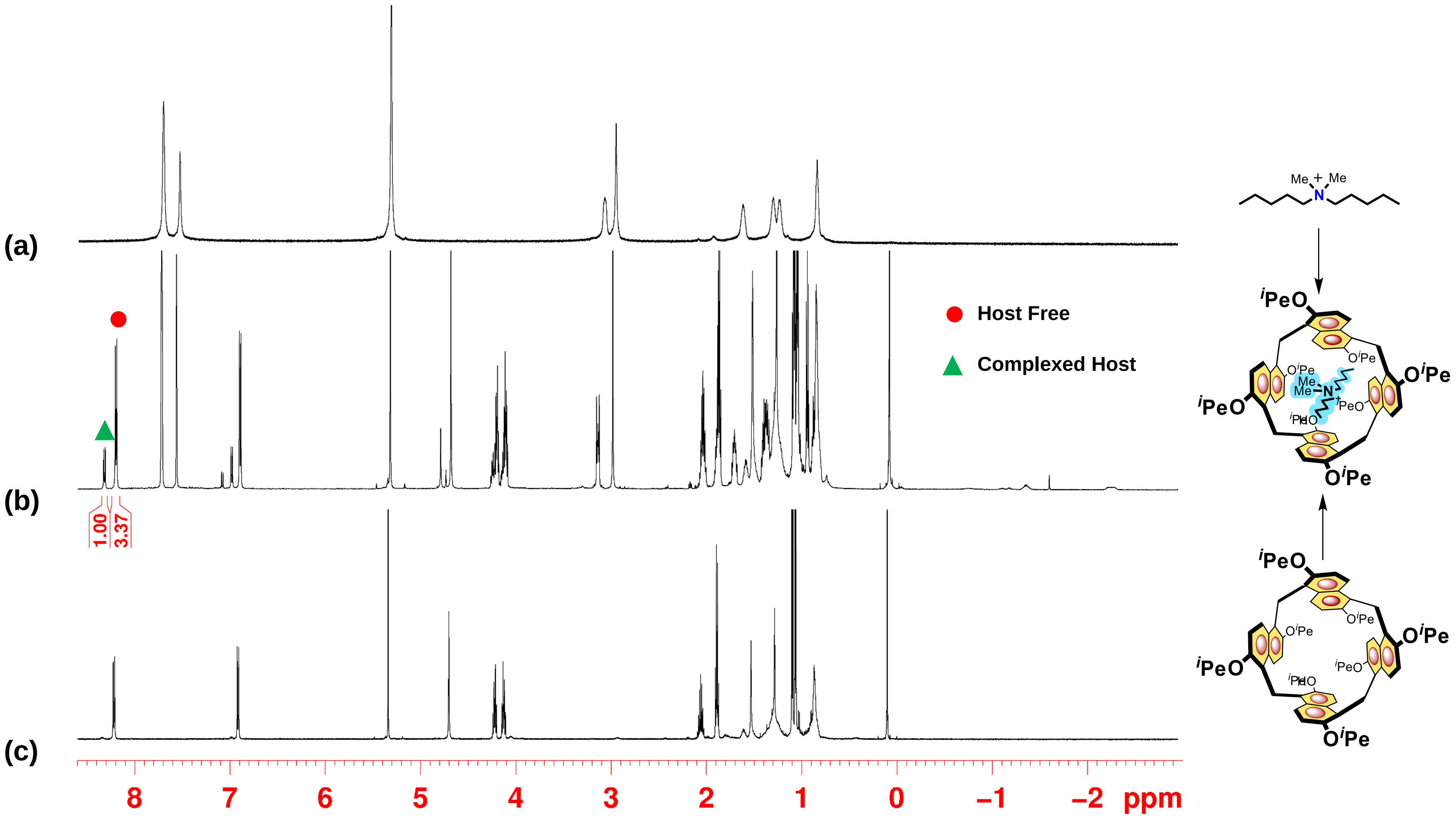

Figure S31: ^{1}H NMR spectra (600 MHz, CD_2Cl_2, 298 K) of: (a) a solution of **3^+·BArF$^-$** (b) an equimolar solution (3.20 mM) of **PrS[4]^{iPe}** and **3^+·BArF$^-$** in 0.5 mL of CD_2Cl_2 and (c) a solution of **PrS[4]^{iPe}**.

$$K_{ass} = \frac{\left\{\frac{1.00}{4.37} \cdot 3.20 \cdot 10^{-3} M\right\}}{\left\{\frac{3.37}{4.37} \cdot 3.20 \cdot 10^{-3} M\right\}^2} = \mathbf{125\ M^{-1}}$$

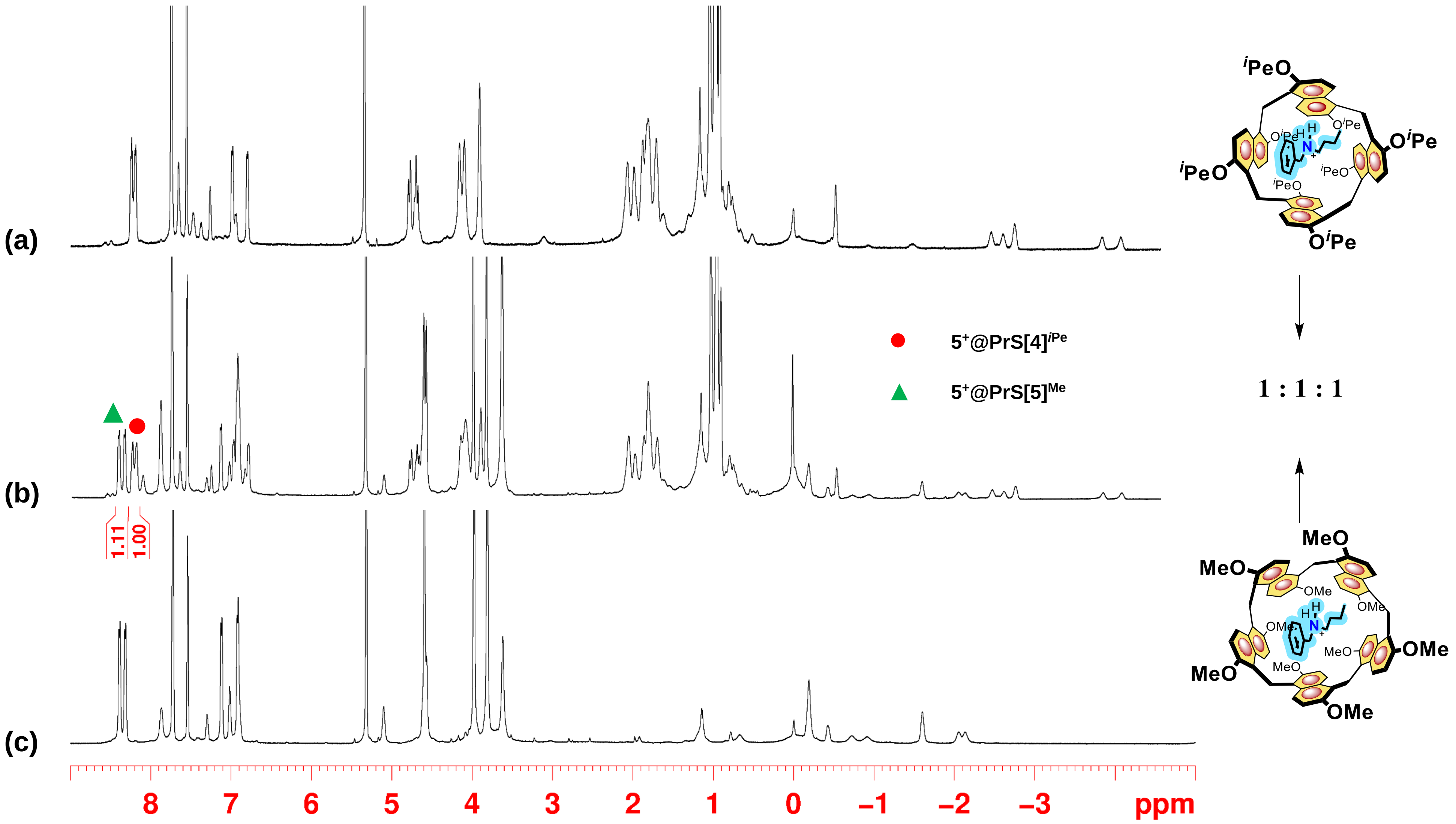

Figure S32: ^{1}H NMR spectra (600 MHz, CD_2Cl_2, 193 K) of: (a) an equimolar solution (4.56 mM) of **PrS[4]*iPe*** and **5^{+}·BArF^{-}** in 0.5 mL of CD_2Cl_2, (b) **PrS[4]*iPe*** in the presence of 1 equivalent of **PrS[5]*Me*** and 1 equivalent of **5^{+}·BArF^{-}** and (c) an equimolar solution (4.56 mM) of **PrS[5]*Me*** and **5^{+}·BArF^{-}** in 0.5 mL of CD_2Cl_2.

$$K_{rel} = \frac{Kass_A}{4440\ M^{-1}} = \frac{\left\{\frac{0.12}{0.23} \cdot 4.56 \cdot 10^{-3} M\right\}^2}{\left\{\frac{0.11}{0.23} \cdot 4.56 \cdot 10^{-3} M\right\}^2} = \frac{5.66 \cdot 10^{-6}}{4.76 \cdot 10^{-6}} = 1.2;\ \ K_{ass} = \mathbf{5600\ M^{-1}}$$

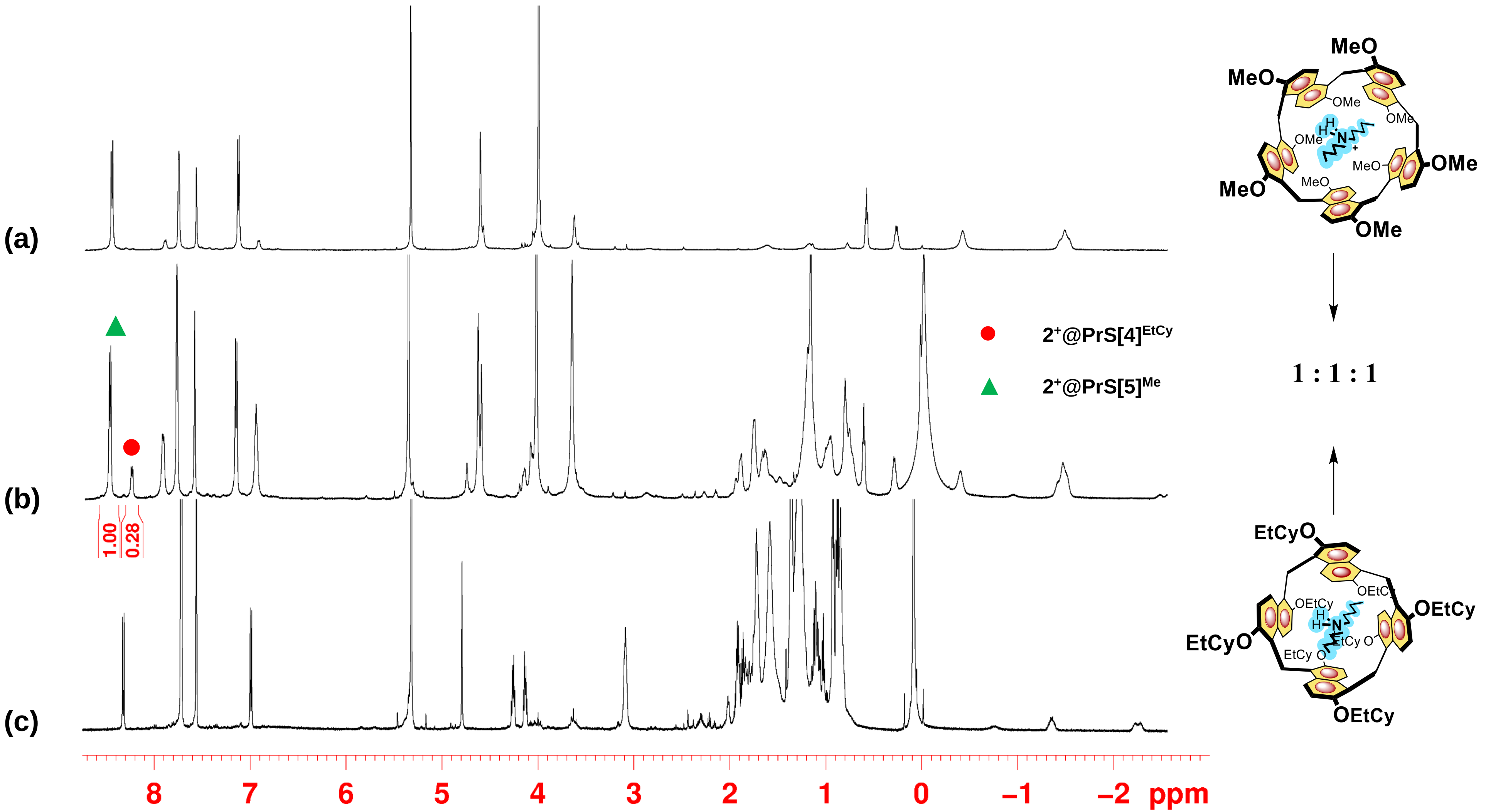

Figure S33: ^{1}H NMR spectra (600 MHz, CD_2Cl_2, 193 K) of: (a) an equimolar solution (4.56 mM) of **PrS[4]*EtCy*** and **2^+·BArF$^–$** in 0.5 mL of CD_2Cl_2, (b) **PrS[4]*EtCy*** in the presence of 1 equivalent of **PrS[5]*Me*** and 1 equivalent of **2^+·BArF$^–$** and (c) an equimolar solution (4.56 mM) of **PrS[5]*Me*** and **2^+·BArF$^–$** in 0.5 mL of CD_2Cl_2.

$$\mathrm{K_{rel}} = \frac{Kass_A}{8500\ M^{-1}} = \frac{\left\{\frac{0.035}{0.135} \cdot 4.56 \cdot 10^{-3} M\right\}^2}{\left\{\frac{0.100}{0.135} \cdot 4.56 \cdot 10^{-3} M\right\}^2} = \frac{1.40 \cdot 10^{-6}}{1.14 \cdot 10^{-5}} = 0.12;\ \ K_{ass} = \mathbf{1000\ M^{-1}}$$

1D and 2D NMR Studies on the Complexation of **PrS[4]*^{iPe}*** with Chiral Guests (*S*)-**6^{2+}**,(*S*)-**7^{+}**,(*S*)-**8^{+} and** (*S*)-**9^{+}.**

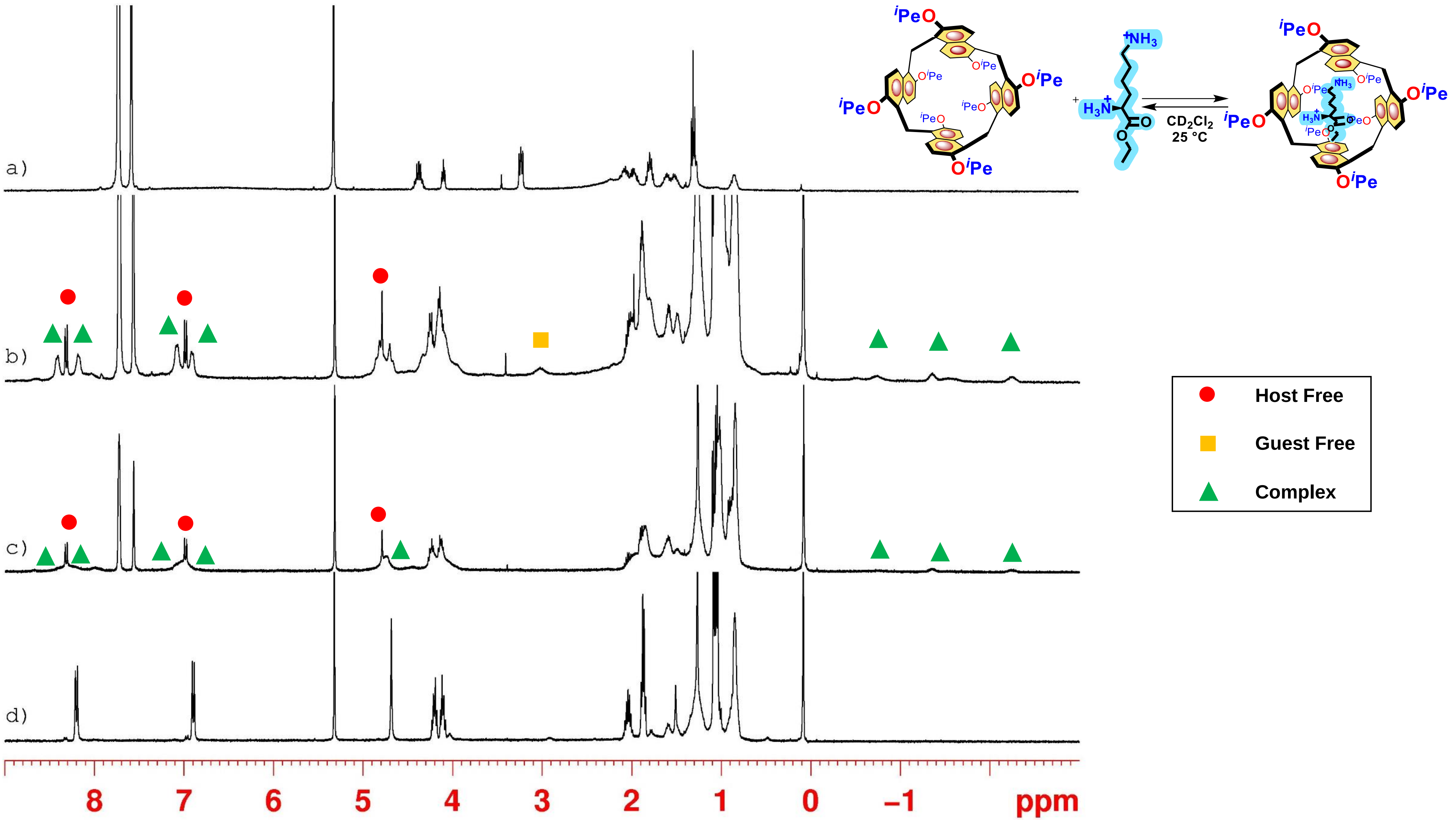

Figure S34: ^{1}H NMR spectra (400 MHz, CD_2Cl_2, 298 K) of: (a) (*S*)-**6^{2+}·2BArF^{-}**, (b) an equimolar solution (4.10 mM) of **PrS[4]*^{iPe}*** and (*S*)-**6^{2+}·2BArF^{-}**, (c) a 1 : 0.5 mixture of **PrS[4]*^{iPe}*** and (*S*)-**6^{2+}·2BArF^{-}**. (d) **PrS[4]*^{iPe}***

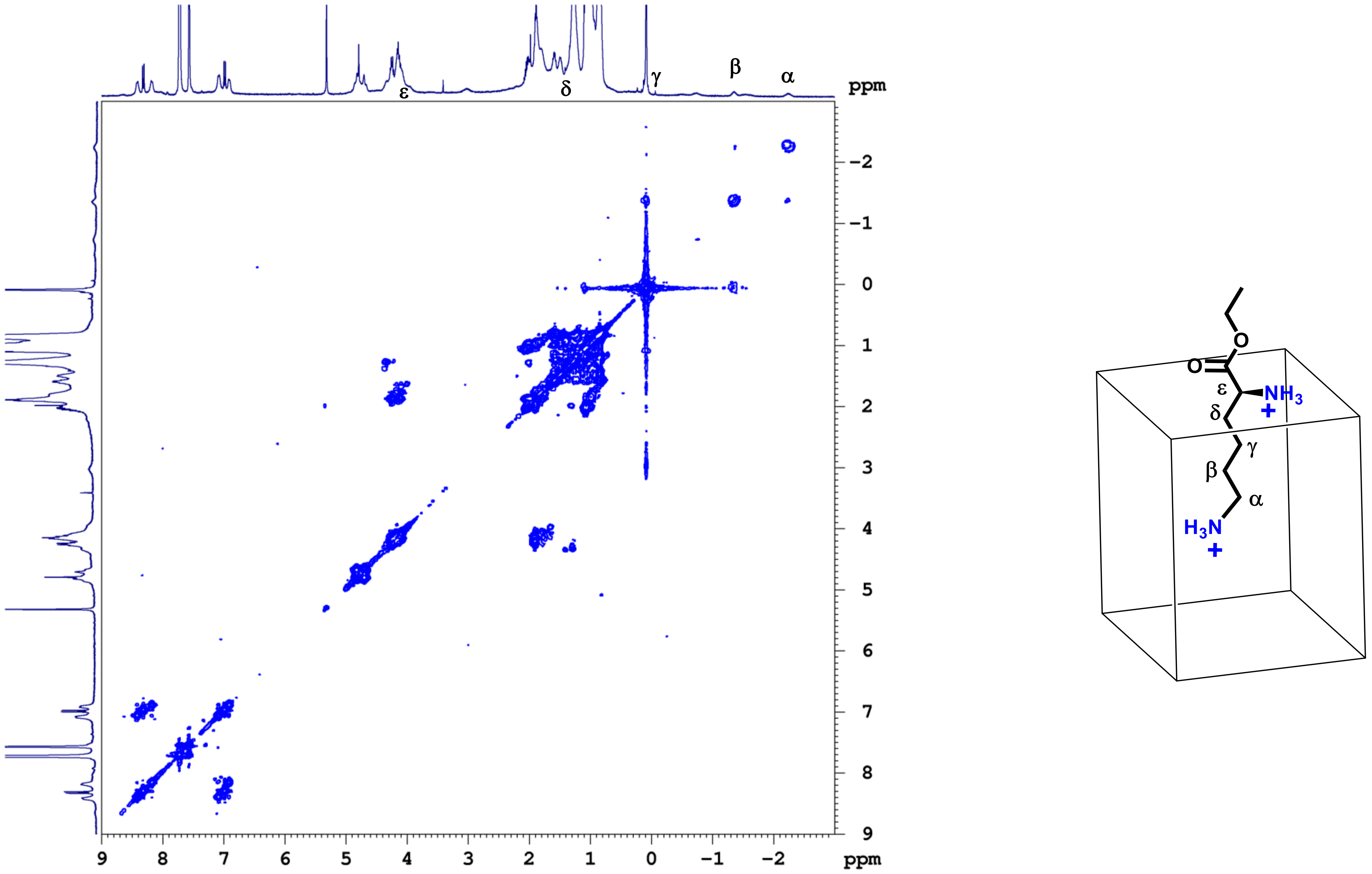

Figure S35: Portion of 2D-DQF COSY spectrum of (*S*)-**6**$^{2+}$@ **PrS[4]**^{iPe} (CD_2Cl_2, 400 MHz, 298 K).

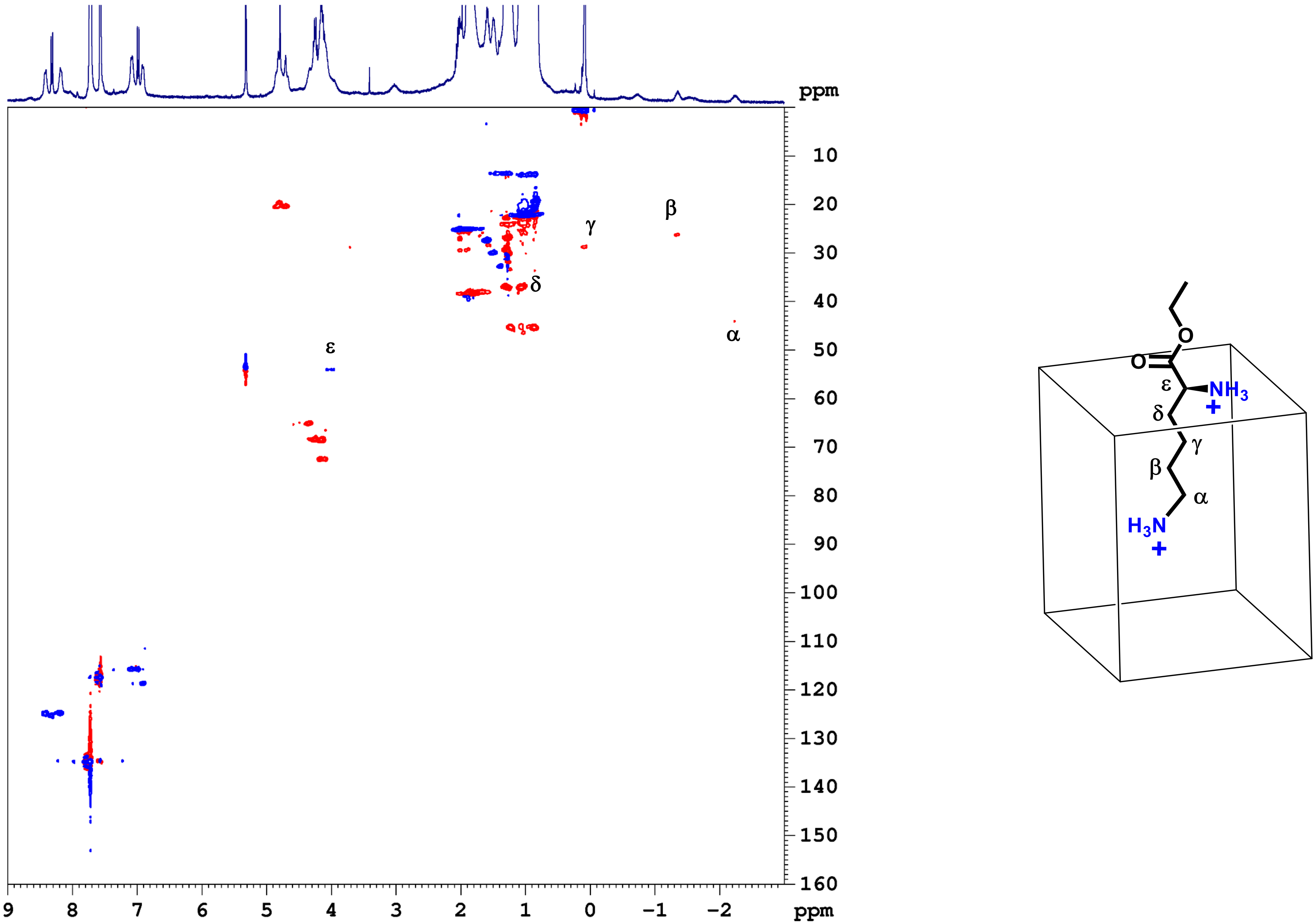

Figure S36: 2D-HSQC spectrum of (*S*)-**6**$^{2+}$@ **PrS[4]**^{iPe} (CD_2Cl_2, 400 MHz, 298 K).

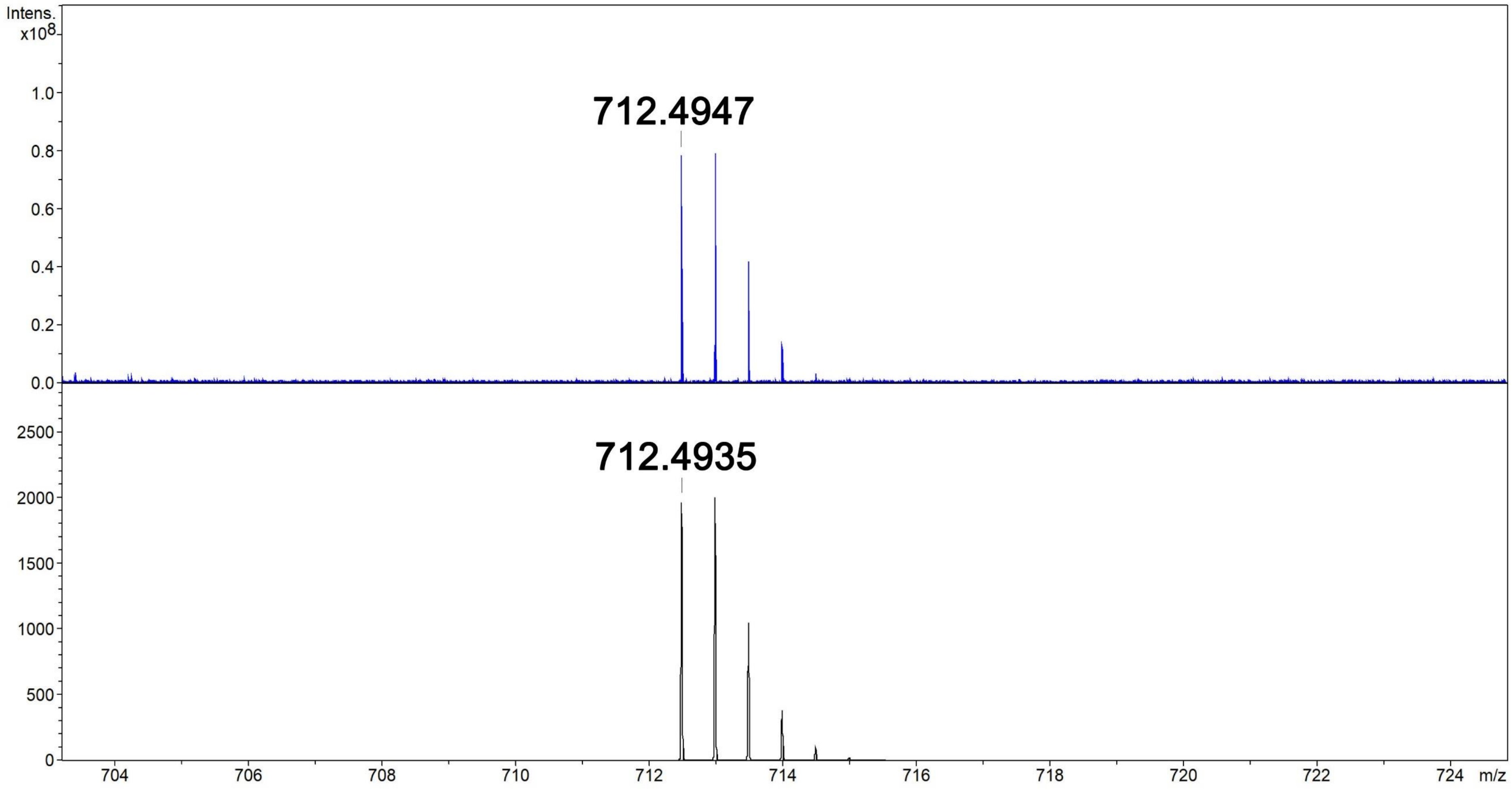

Figure S37: Comparison of experimental (top) and simulated (bottom) HR FT-ICR mass spectra of (*S*)-**6**$^{2+}$@ **PrS[4]**^{iPe}**.**

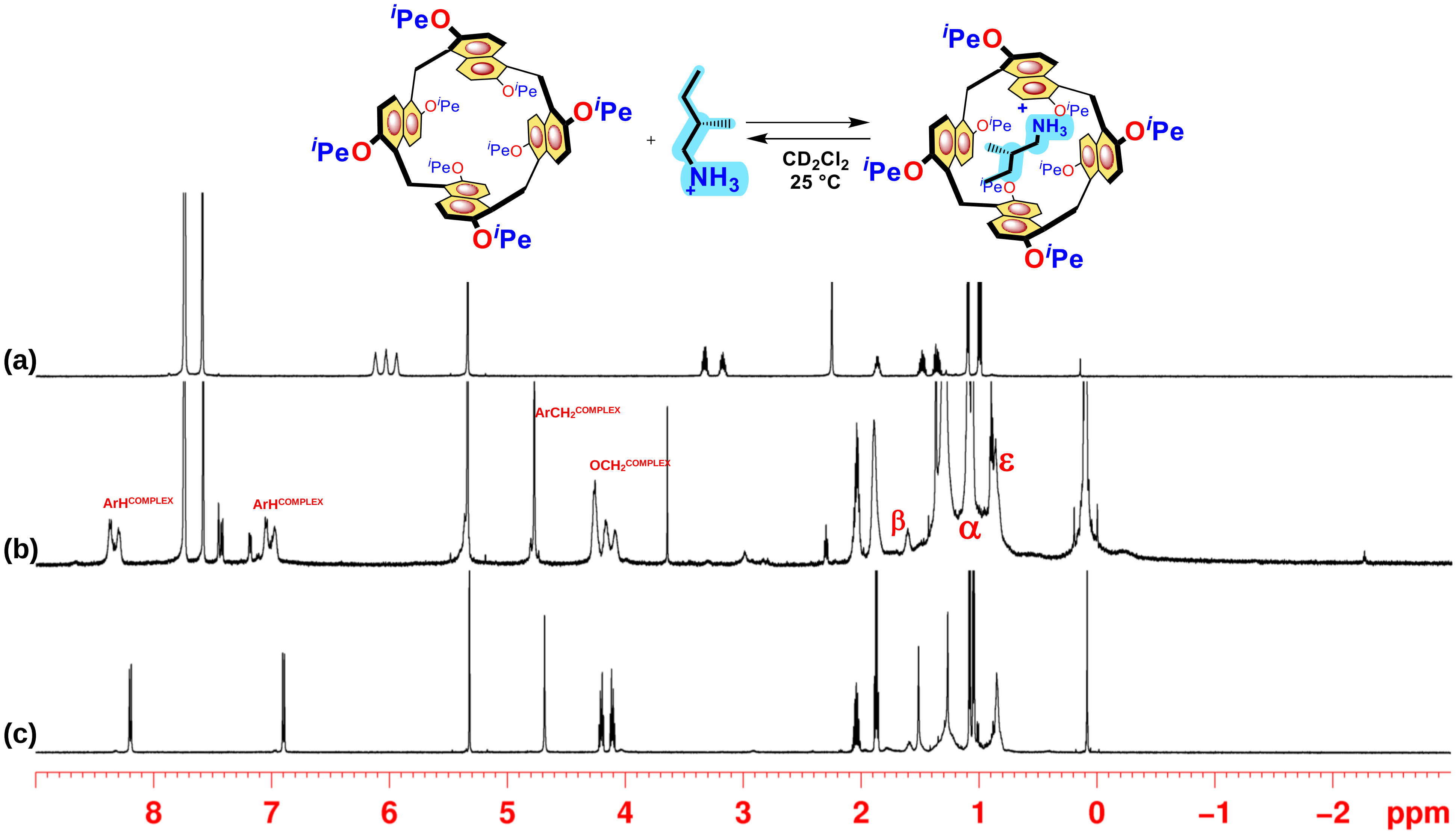

Figure S38: ^{1}H NMR spectra (600 MHz, CD_2Cl_2, 298 K) of: (a) (*S*)-**7^+·BArF**$^-$, (b) an equimolar solution (4.10 mM) of **PrS[4]**^{iPe} and (*S*)-**7^+·BArF**$^-$ and (c) **PrS[4]**^{iPe}.

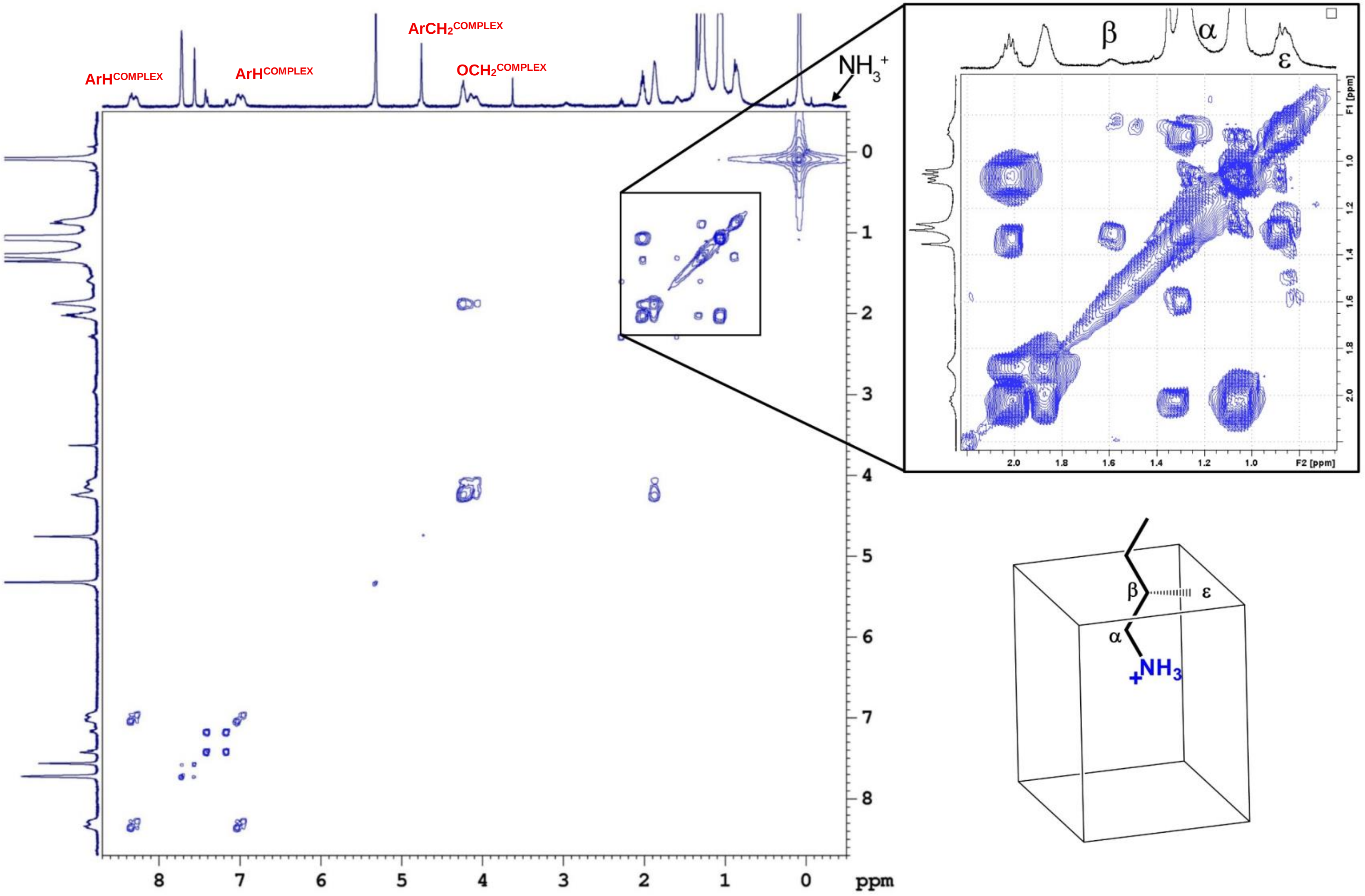

Figure S39: Portion of 2D-COSY spectrum of (*S*)-**7**$^{+}$@ **PrS[4]**^{iPe} (CD_2Cl_2, 400 MHz, 298 K).

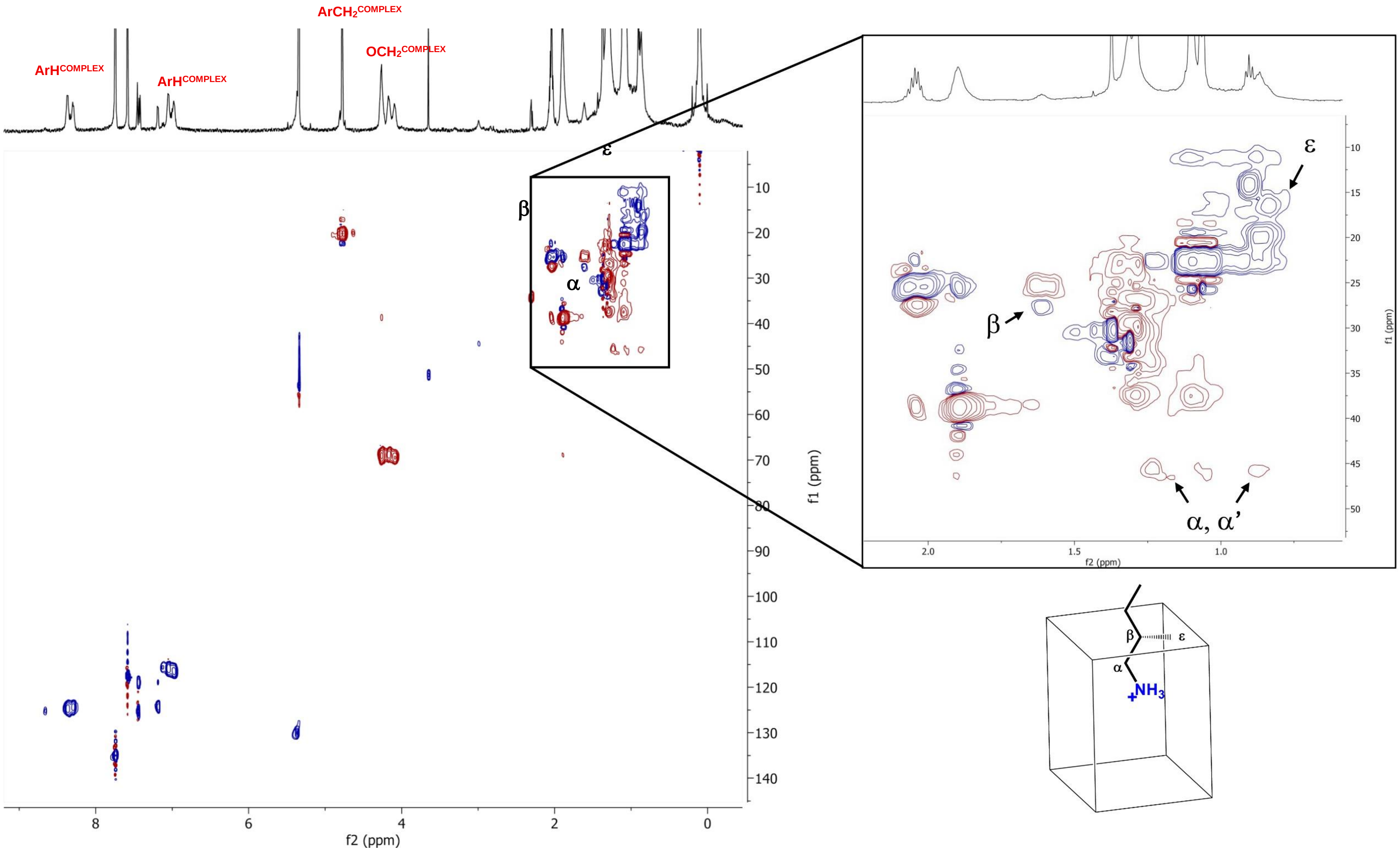

Figure S40: 2D-HSQC spectrum of (*S*)-**7^+**@ **PrS[4]*^{iPe}*** (CD_2Cl_2, 600 MHz, 298 K).

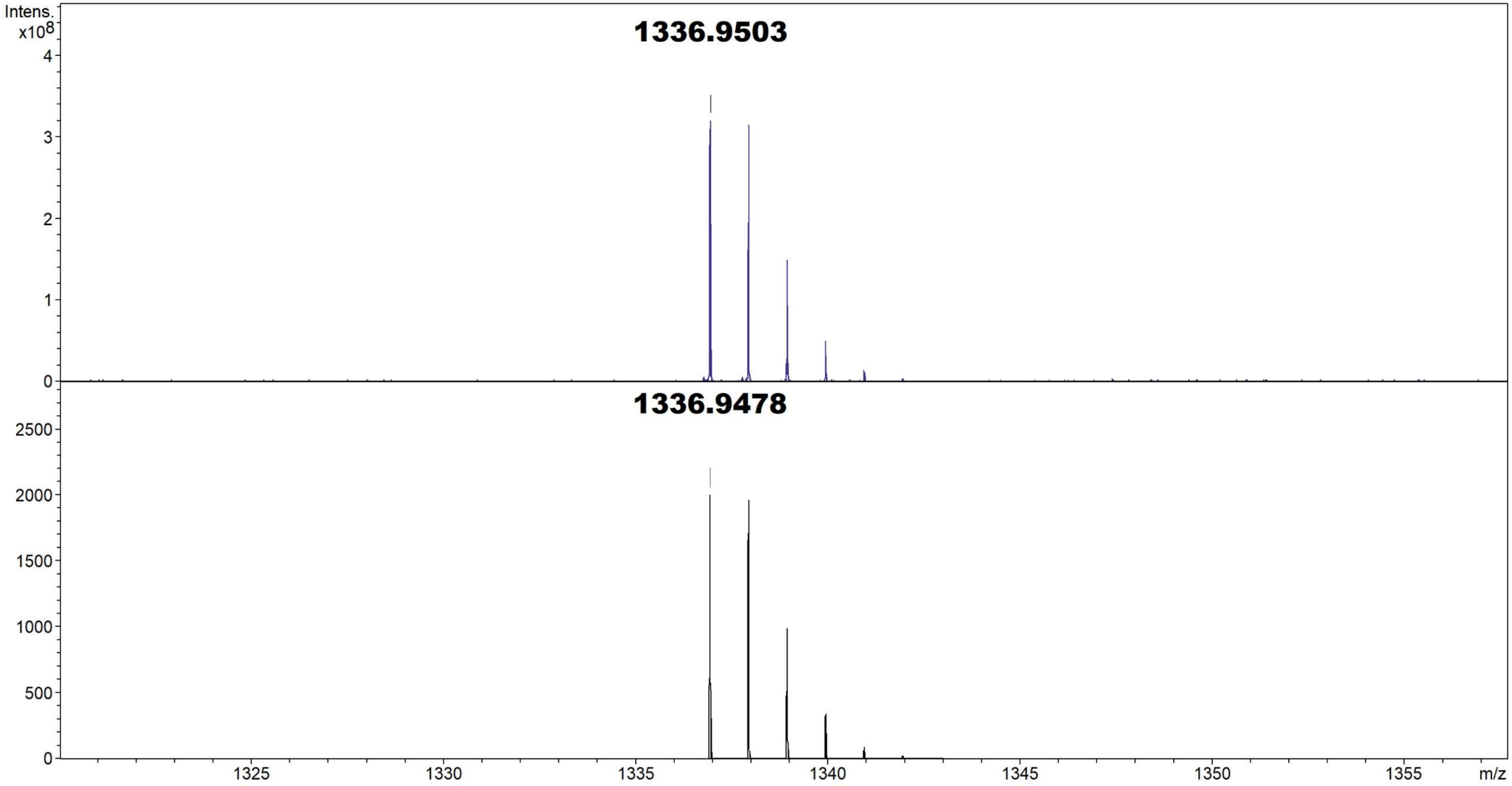

Figure S41: Comparison of experimental (Top) and simulated (Bottom) HR FT-ICR mass spectra of (*S*)-**7**$^{+}$@ **PrS[4]**^{iPe}**.**

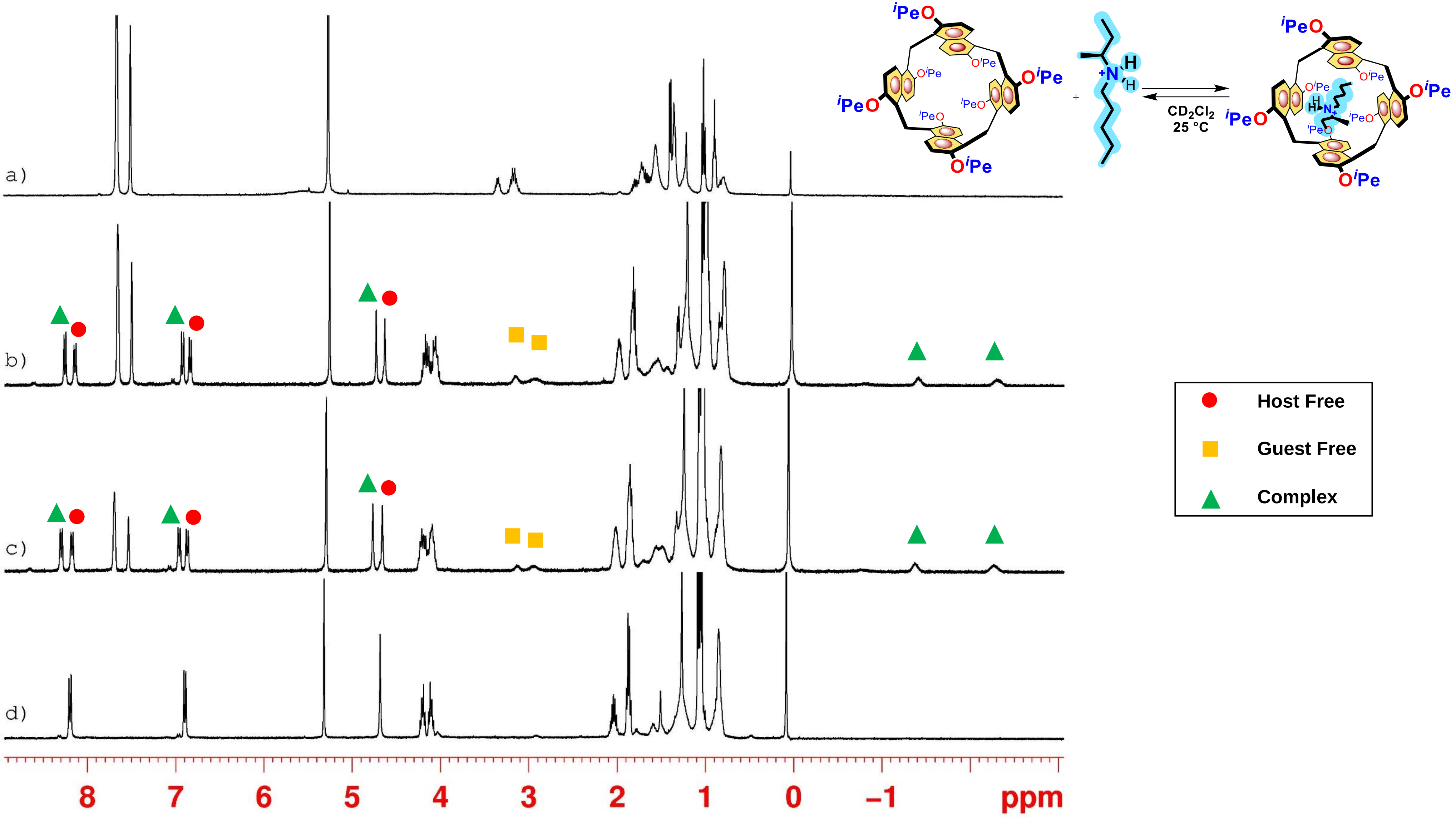

Figure S42: ^{1}H NMR spectra (400 MHz, CD_2Cl_2, 298 K) of: (a) (*S*)-**9**$^{+}$·**BArF**$^{-}$, (b) an equimolar solution (4.10 mM) of **PrS[4]**^{iPe} and (*S*)-**9**$^{+}$·**BArF**$^{-}$**,** (c) a 1 : 0.5 mixture of **PrS[4]**^{iPe},and (*S*)-**9**$^{+}$·**BArF**$^{-}$and (d) **PrS[4]**^{iPe}.

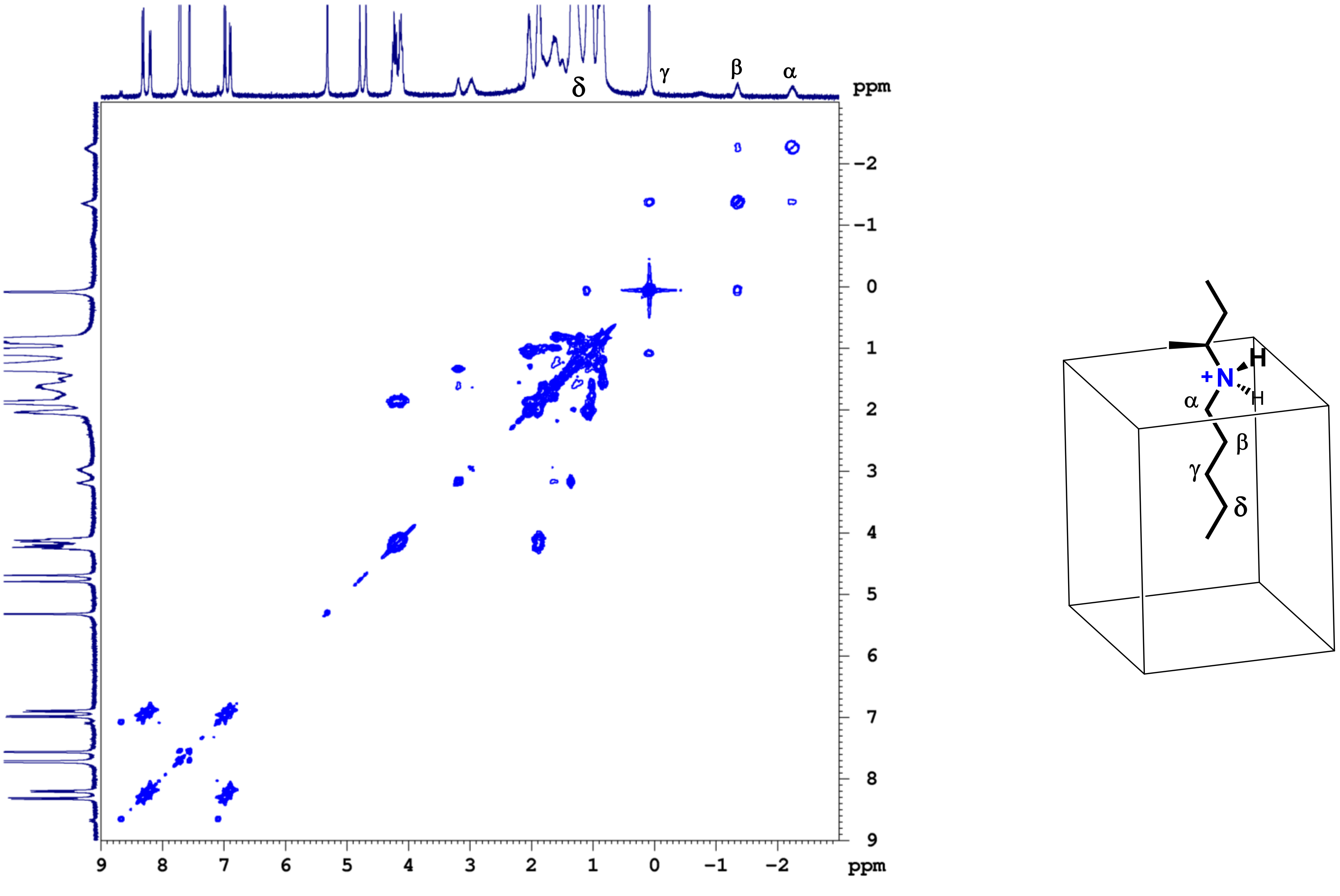

Figure S43: 2D-DQF COSY spectrum of (*S*)-**9**⁺@ **PrS[4]**^{iPe} (CD_2Cl_2, 400 MHz, 298 K).

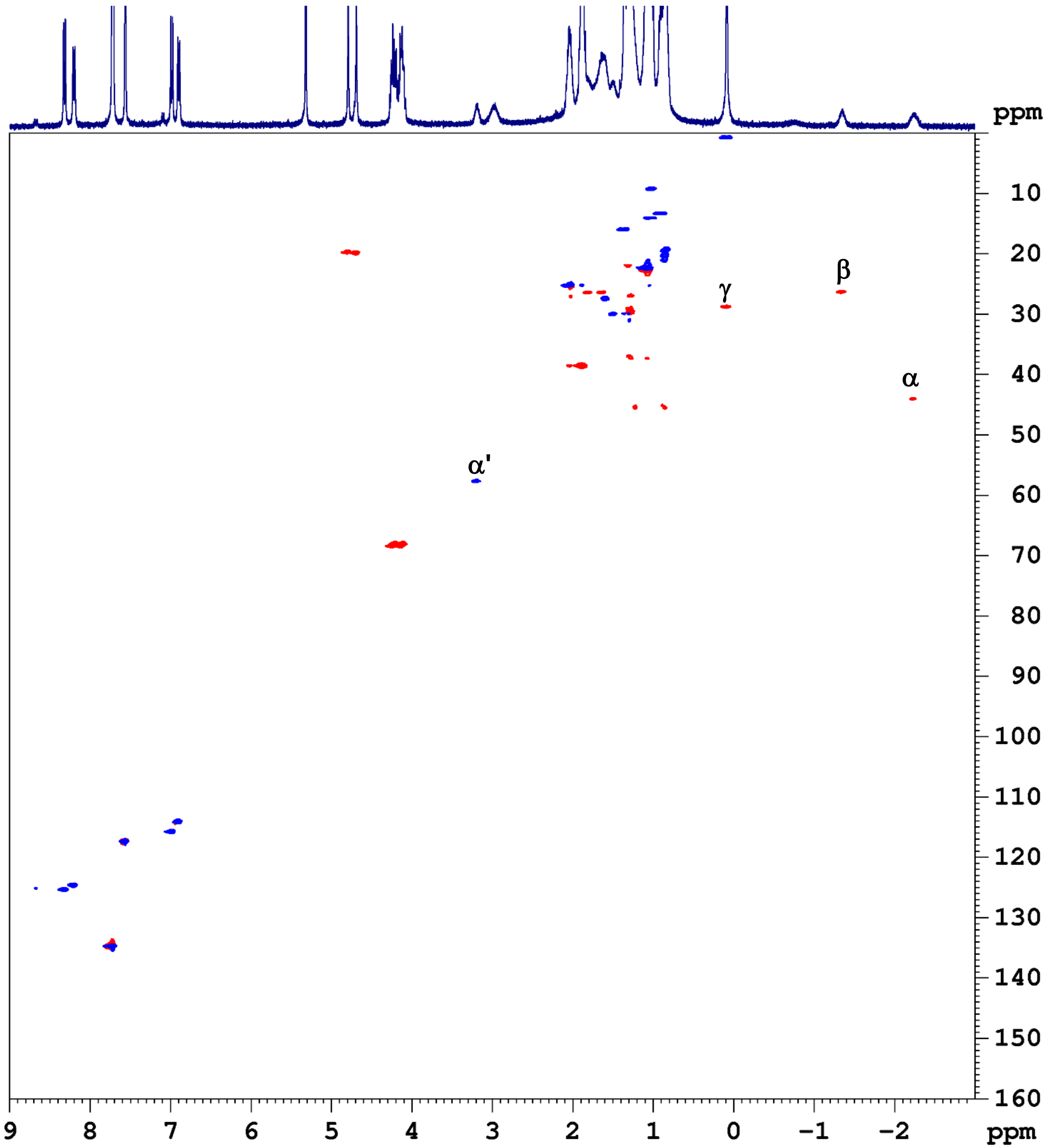

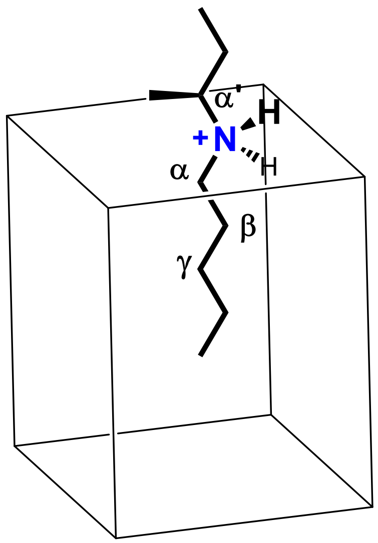

Figure S44: 2D-HSQC spectrum of (*S*)-**9**$^{+}$@ **PrS[4]**^{iPe} (CD_2Cl_2, 400 MHz, 298 K).

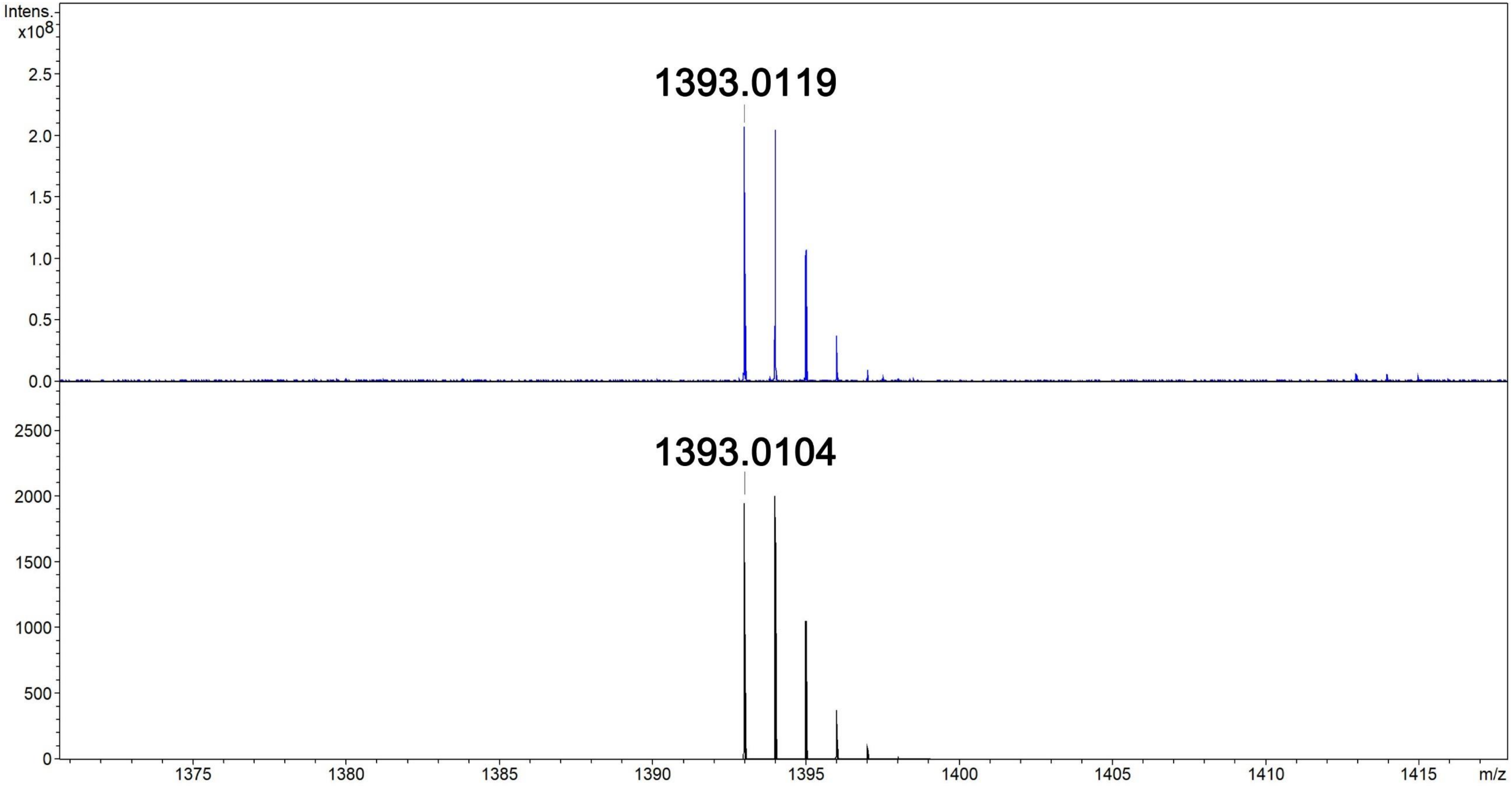

Figure S45: Comparison of experimental (Top) and simulated (Bottom) HR FT-ICR mass spectra (*S*)-**9**$^{+}$@ **PrS[4]**^{iPe}.

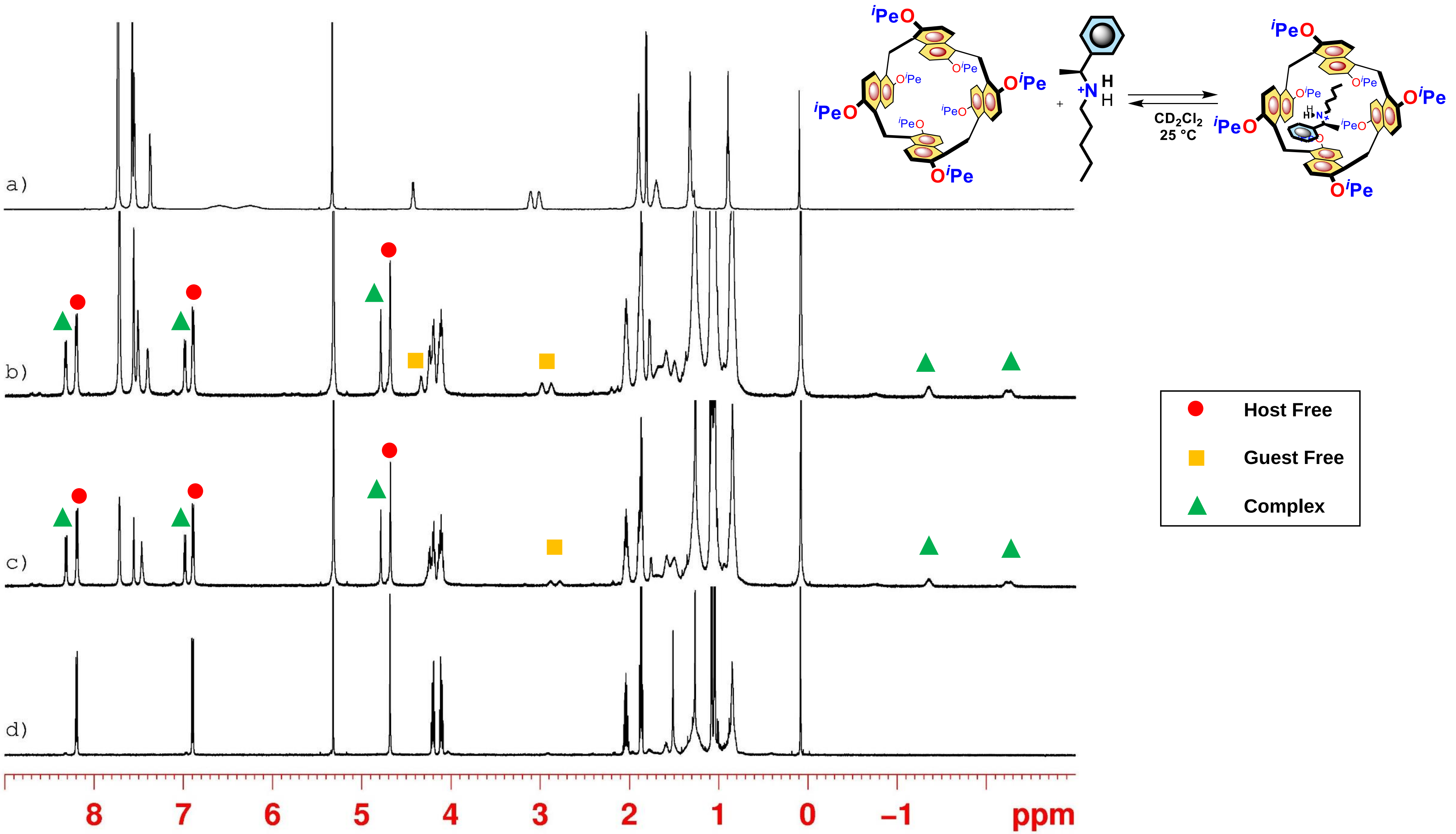

Figure S46: ^{1}H NMR spectra (600 MHz, CD_2Cl_2, 298 K) of: (a) (*S*)-**8^+·BArF**$^-$, (b) an equimolar solution (4.10 mM) of **PrS[4]**^{iPe} and (*S*)-**8^+·BArF**$^-$, (c) a 1 : 0.5 mixture of **PrS[4]**^{iPe} and (*S*)-**8^+·BArF**$^-$ and (d) **PrS[4]**^{iPe}.

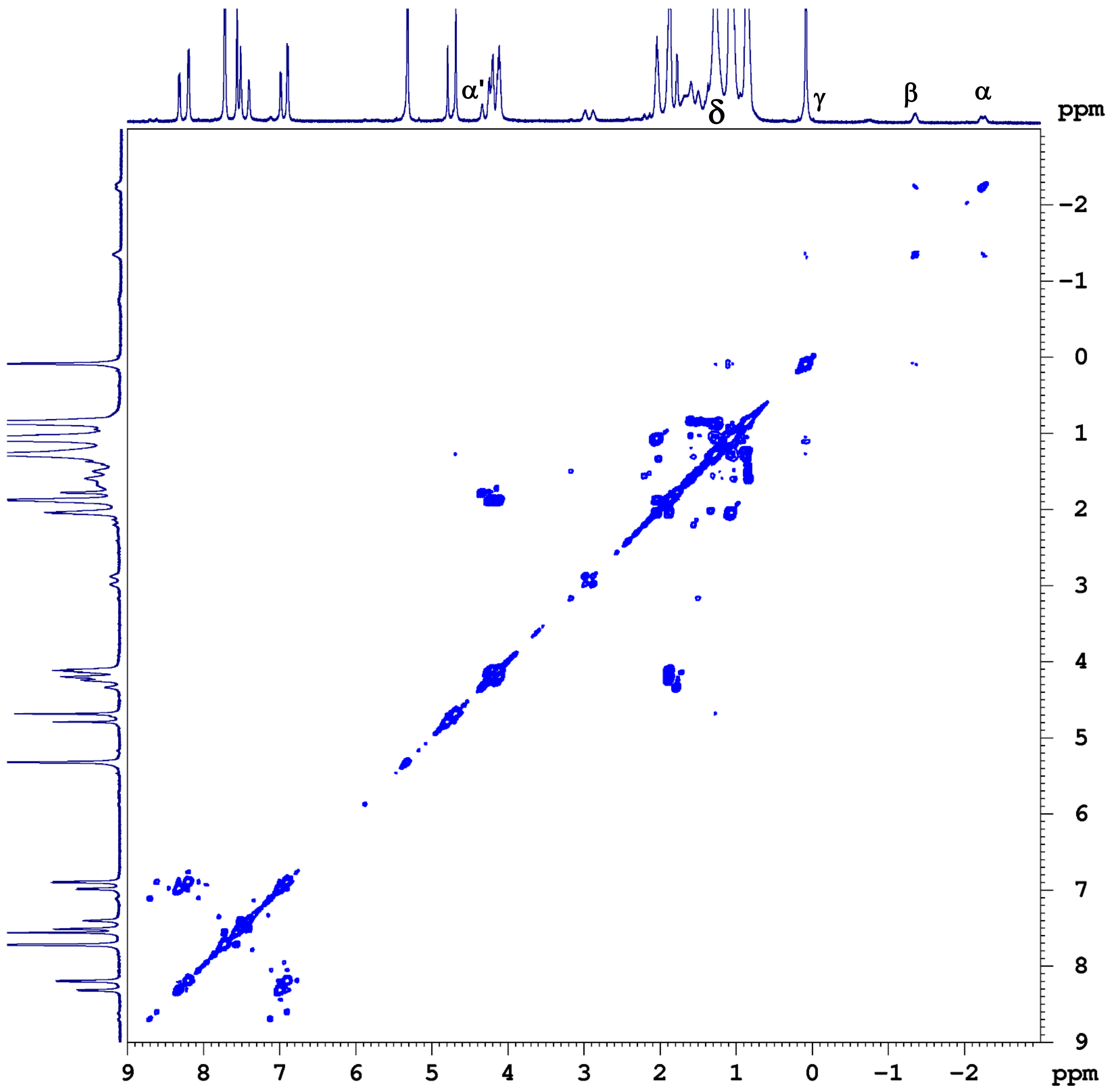

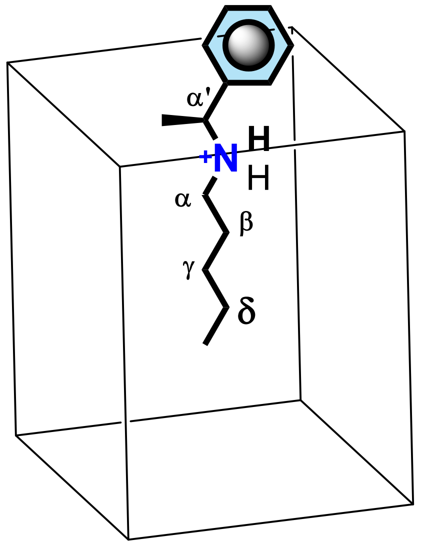

Figure S47: 2D-DQF COSY spectrum of (*S*)-**8**$^{+}$@ **PrS[4]**^{iPe} (CD_2Cl_2, 600 MHz, 298 K).

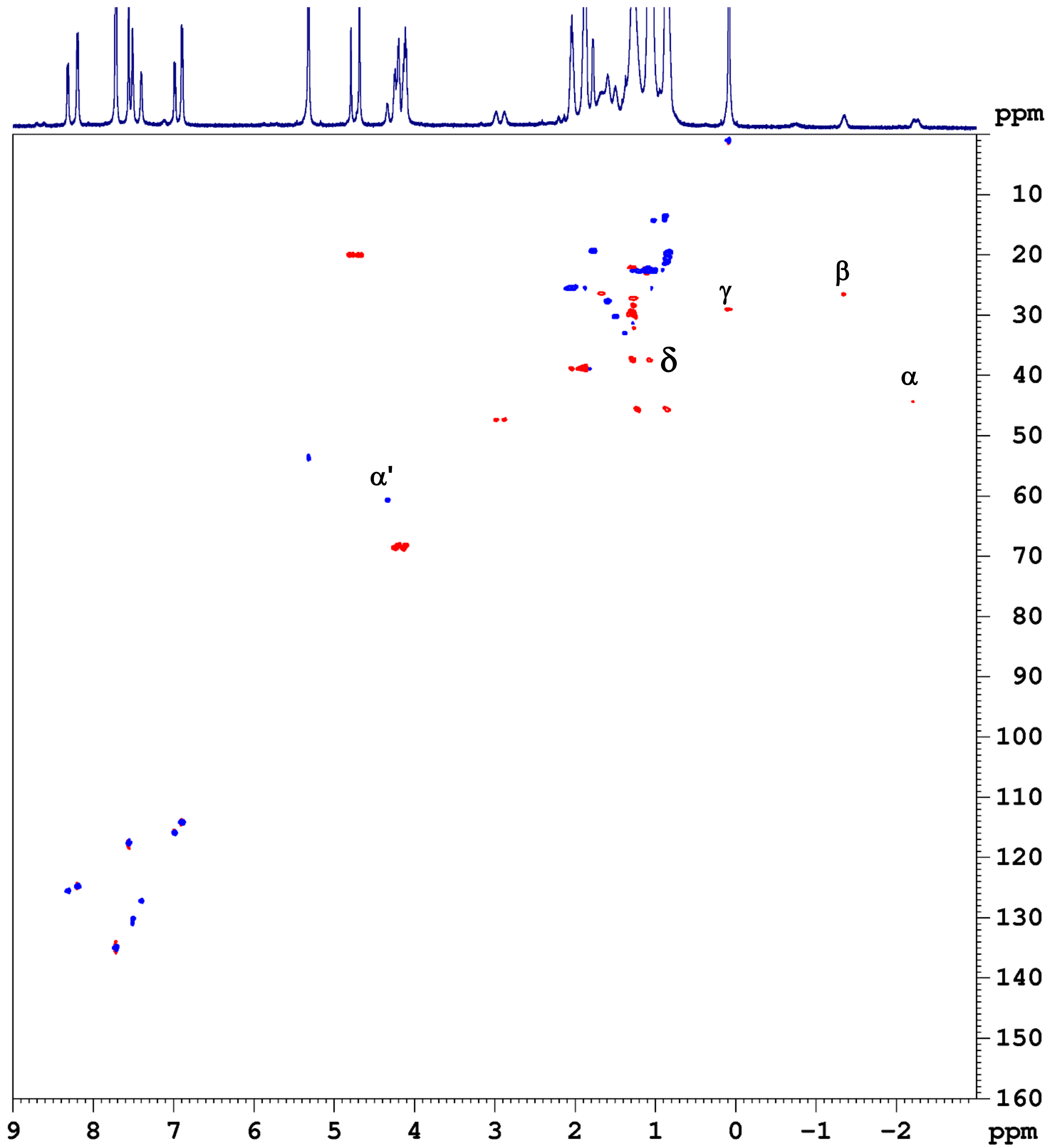

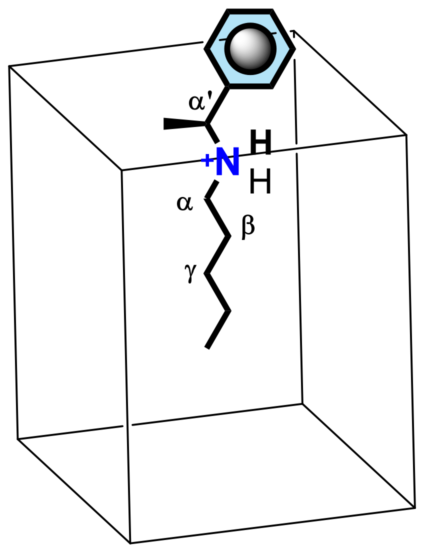

Figure S48: 2D-HSQC spectrum of (*S*)-**8**$^{+}$@ **PrS[4]**^{iPe} (CD_2Cl_2, 600 MHz, 298 K).

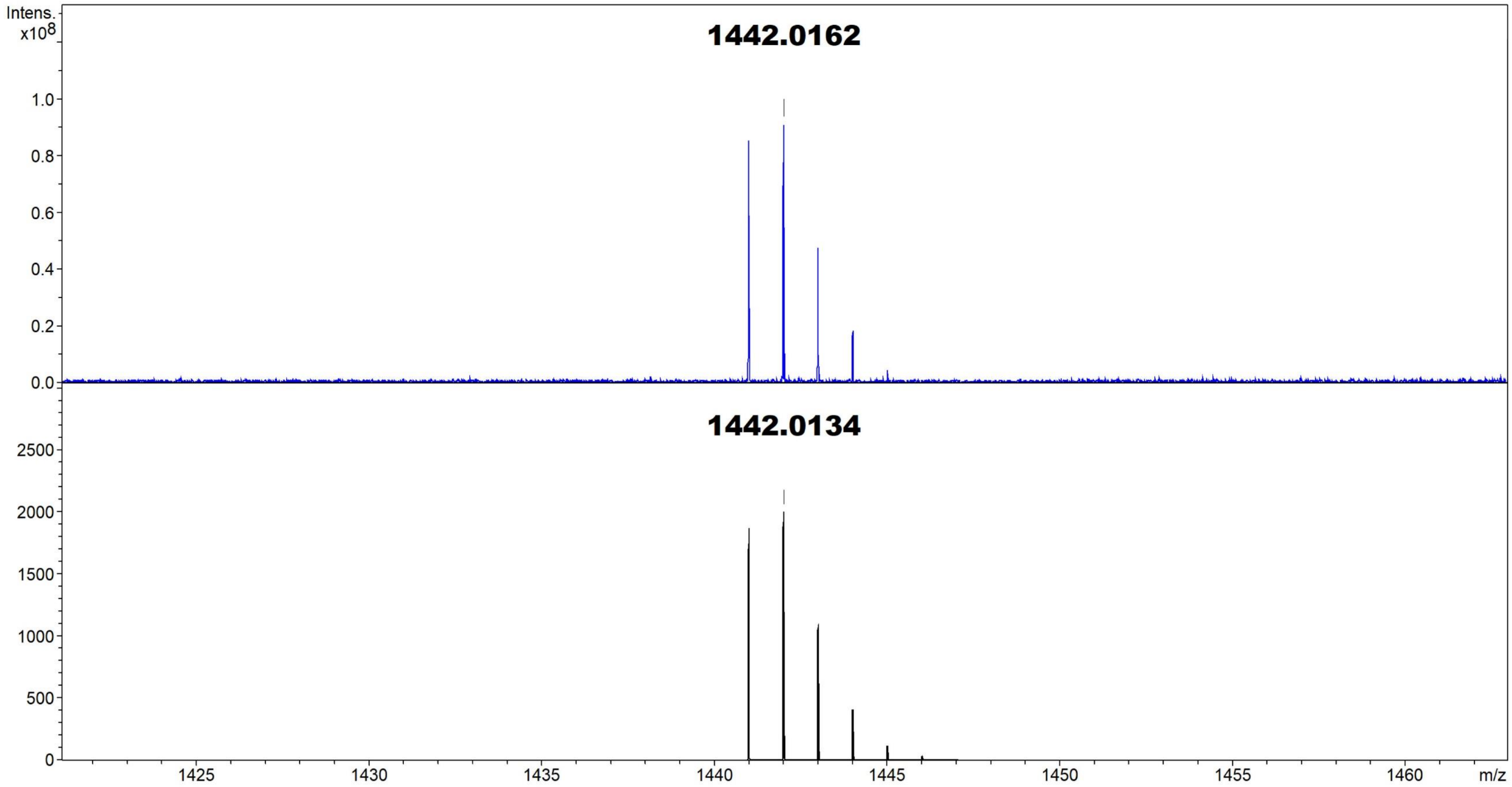

Figure S49: Comparison of experimental (Top) and simulated (Bottom) HR FT-ICR mass spectra of (*S*)-**8**$^{+}$@ **PrS[4]**^{iPe}**.**

Details on the Calculation of Association Constants for the Complexation of **PrS[4]**^{iPe} with Chiral Guests (*S*)-**6**$^{2+}$,(*S*)-**7**$^{+}$,(*S*)-**8**$^{+}$ and (*S*)-**9**$^{+}$

Table S2: Association constant (K_{ass}, M^{-1}) values for the formation of the complexes between the chiral guests (*S*)-**6**$^{2+}$,(*S*)-**7**$^{+}$,(*S*)-**8**$^{+}$ and (*S*)-**9**$^{+}$ as BArF^{-} salts and the prism[n]arenes. Determined by ^{1}H NMR experiments in CD_2Cl_2. Errors < 15% calculated as mean values of three measures.

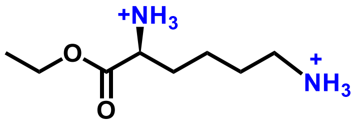
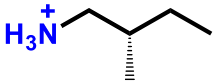
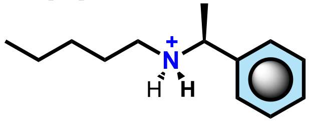
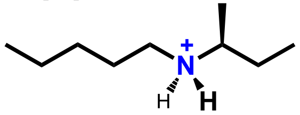

	**(*S*)-6^{2+} · 2(BArF^{-})**	**(*S*)-7^{+} · BArF^{-}**	**(*S*)-8^{+} · BArF^{-}**	**(*S*)-9^{+} · BArF^{-}**
**PrS[4]**^{iPe}	**1900** (a) – Fig. S53	**40000** (b) – Fig. S51	**290** (a) – Fig. S54	**690** (a) – Fig. S52
**PrS[5]**^{Me}	---	**9000** (a) – Fig. S50	---	---

a and **b** are the methods used for the K_{ass} determination, as reported on page S30. For method (**b**), ^{1}H NMR competition experiments were conducted using a 1:1:1 mixture of **PrS[4]**^{iPe}, **PrS[5]**^{Me}, and guest molecules in an NMR tube, with CD_2Cl_2 as the solvent. The integrals were normalized to account for the number of protons.

Method A: Integration of the ^{1}H NMR signals of both free and complexed host in an equimolar solution of hosts and guests solubilized in CD_2Cl_2 (see captions in Figures S50, S52, S53, S54).
Method B: An NMR competition experiment was employed to determine the *K*ass for the formation of (*S*)-**7**$^{+}$@**PrS[4]**^{iPe}, using **PrS[5]**^{Me} as the competitive host. First, we calculated the binding constants for the formation of the complexes between the guest (*S*)-**7**$^{+}$ and **PrS[5]**^{Me} using Method A (Figure S50). The obtained values were then used as references for Method B, as presented in Figure S51.

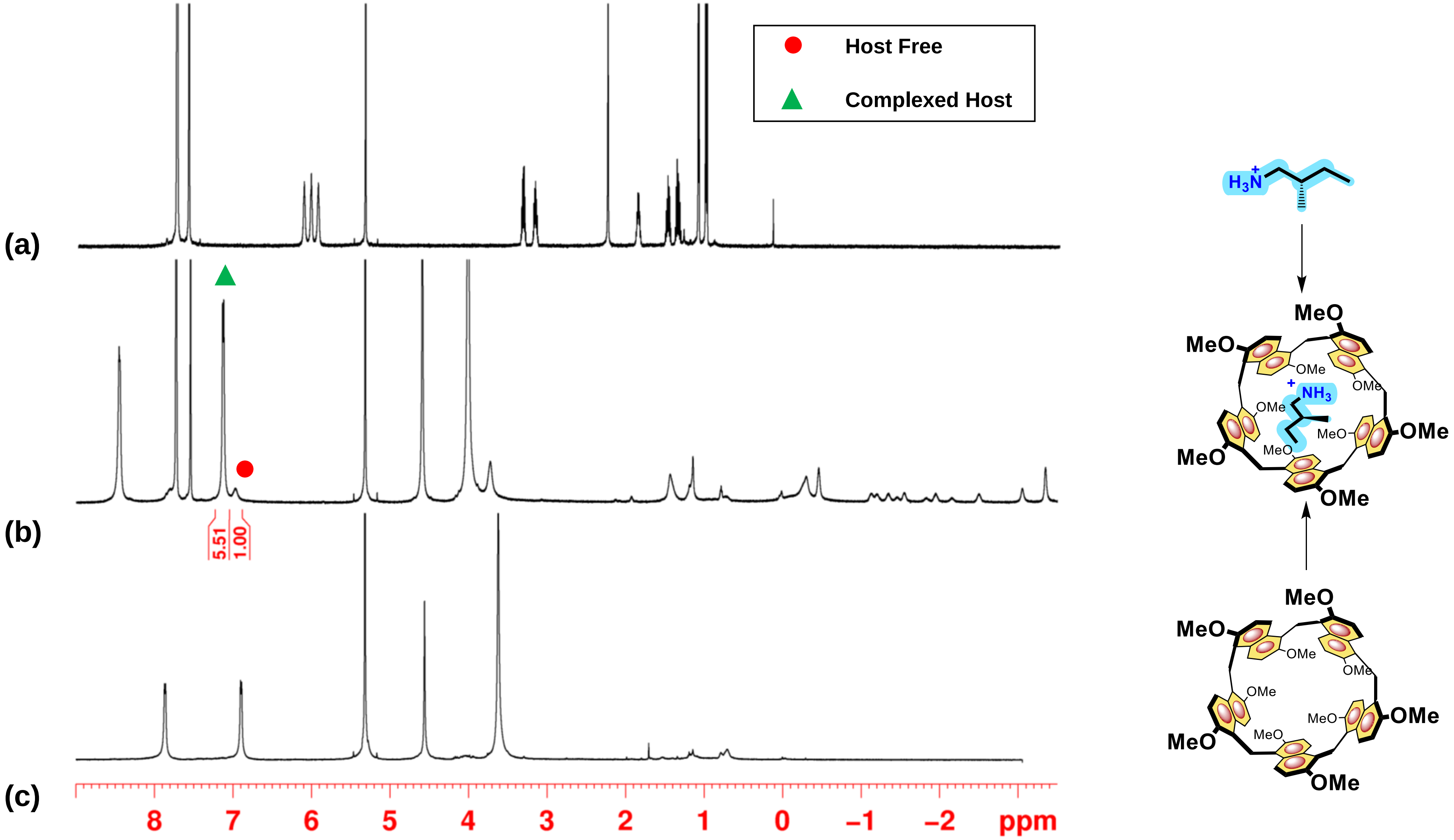

Figure S50: ^{1}H NMR spectra (600 MHz, CD_2Cl_2,193 K) of: (a) a solution of (*S*)-**7**$^{+}$**·BArF**$^{-}$ (b) an equimolar solution (4.00 mM) of **PrS[5]**^{Me} and (*S*)-**7**$^{+}$**·BArF**$^{-}$ in 0.5 mL of CD_2Cl_2 and (c) a solution of **PrS[5]**^{Me}.

$$K_{ass} = \frac{\left\{\frac{5.51}{6.51} \cdot 4.00 \cdot 10^{-3} M\right\}}{\left\{\frac{1.00}{6.51} \cdot 4.00 \cdot 10^{-3} M\right\}^2} = \mathbf{9000\ M^{-1}}$$

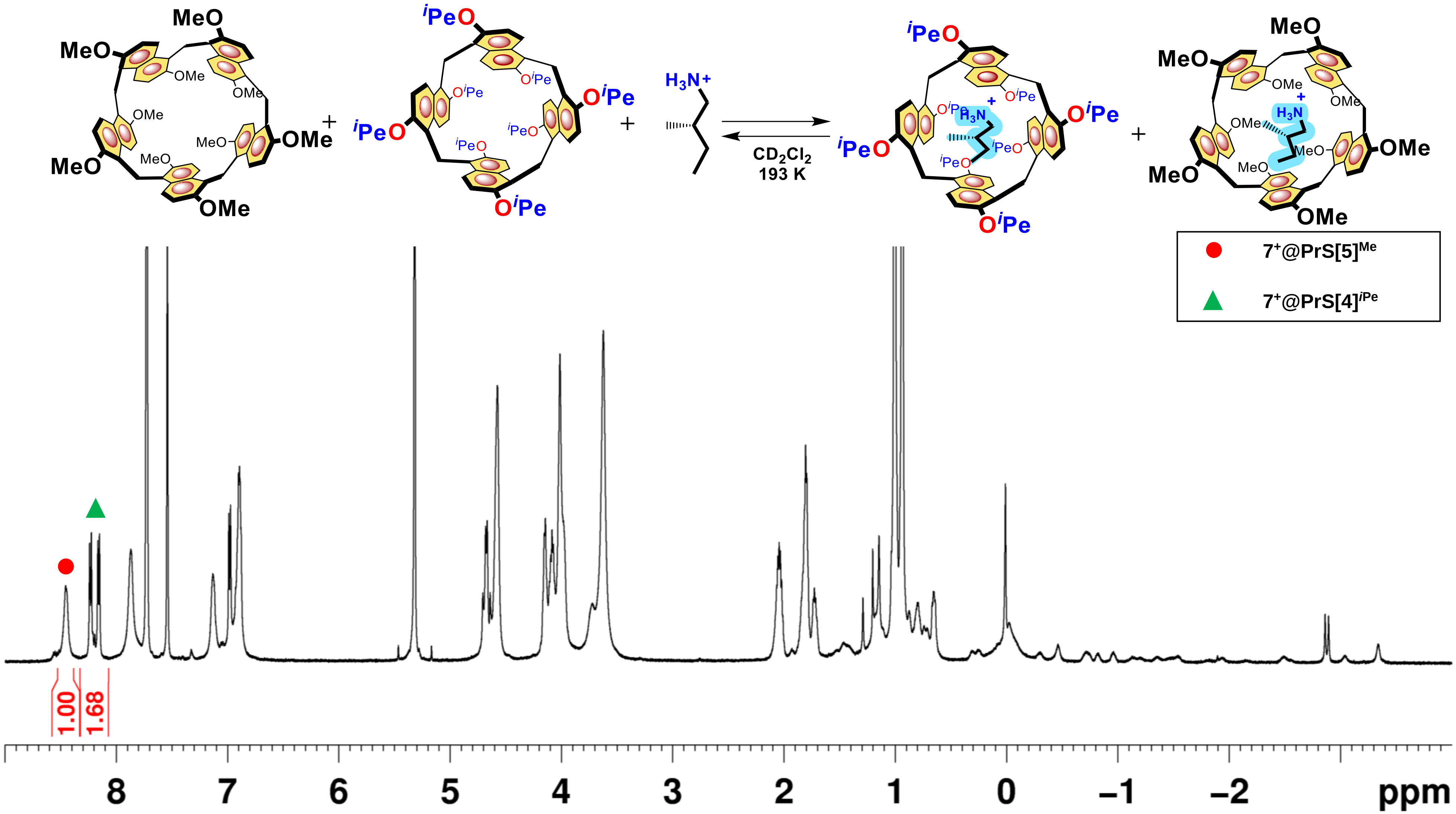

Figure S51: ^{1}H NMR spectrum (600 MHz, CD_2Cl_2,193 K) of an equimolar solution (2.67 mM) of **PrS[4]^{iPe}, PrS[5]^{Me}** and (*S*)-**7^{+}·BArF**$^{-}$ in 0.5 mL of CD_2Cl_2.

$$K_{\mathrm{rel}} = \frac{Kass_A}{9000\ M^{-1}} = \frac{\left\{\frac{0.21}{0.31} \cdot 2.67 \cdot 10^{-3} M\right\}^2}{\left\{\frac{0.10}{0.31} \cdot 2.67 \cdot 10^{-3} M\right\}^2} = \frac{3.27 \cdot 10^{-6}}{7.42 \cdot 10^{-7}} = 4.41; \quad K_{ass} = \mathbf{40000\ M^{-1}}$$

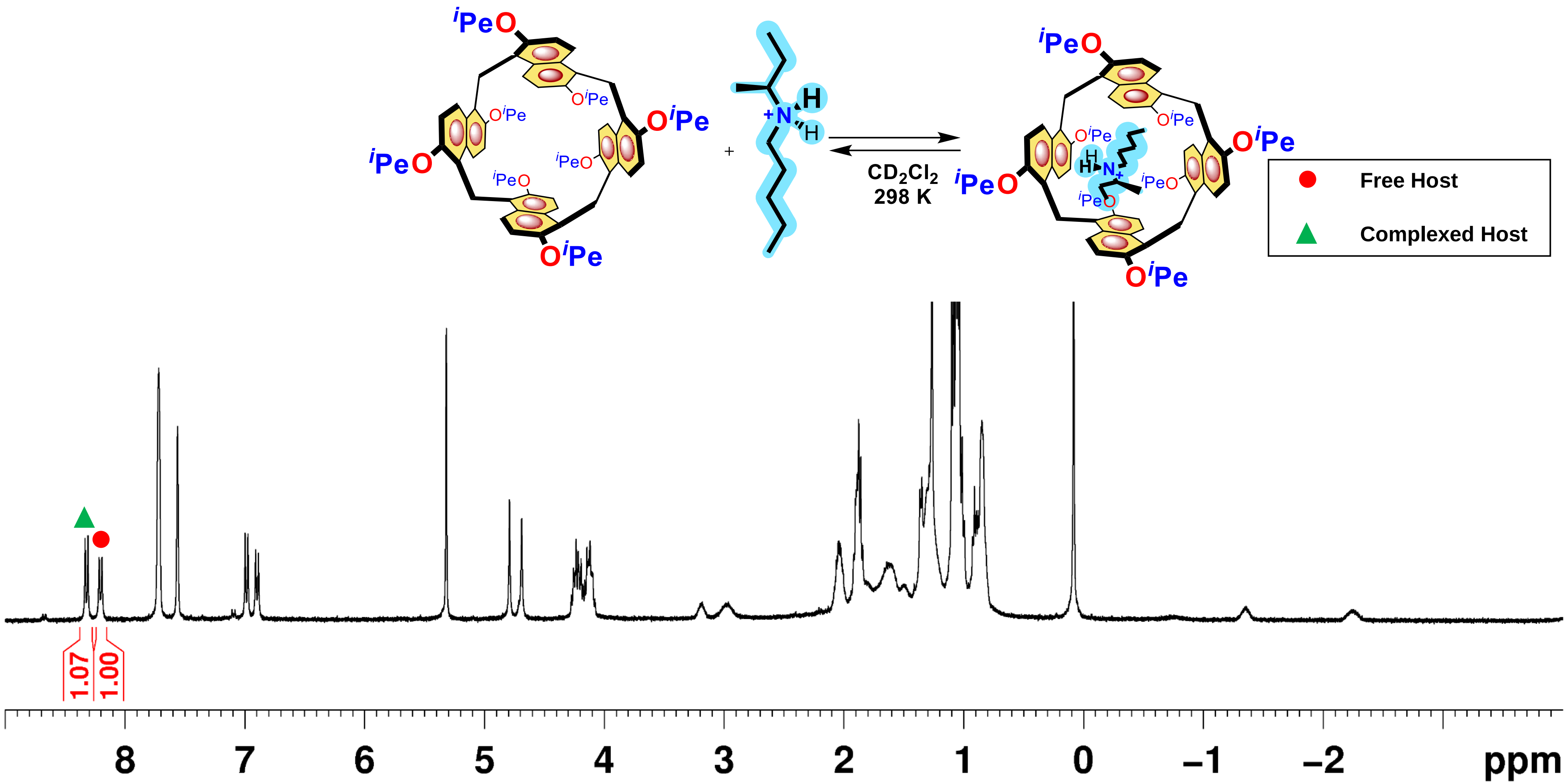

Figure S52: ^{1}H NMR spectrum (400 MHz, CD_2Cl_2, 298 K) of an equimolar solution (3.20 mM) of **PrS[4]^{iPe}** and (*S*)-**9^{+}·BArF^{-}** in 0.5 mL of CD_2Cl_2.

$$K_{ass} = \frac{\left\{\frac{1.07}{2.07} \cdot 3.20 \cdot 10^{-3} M\right\}}{\left\{\frac{1.00}{2.07} \cdot 3.20 \cdot 10^{-3} M\right\}^2} = \mathbf{690\ M^{-1}}$$

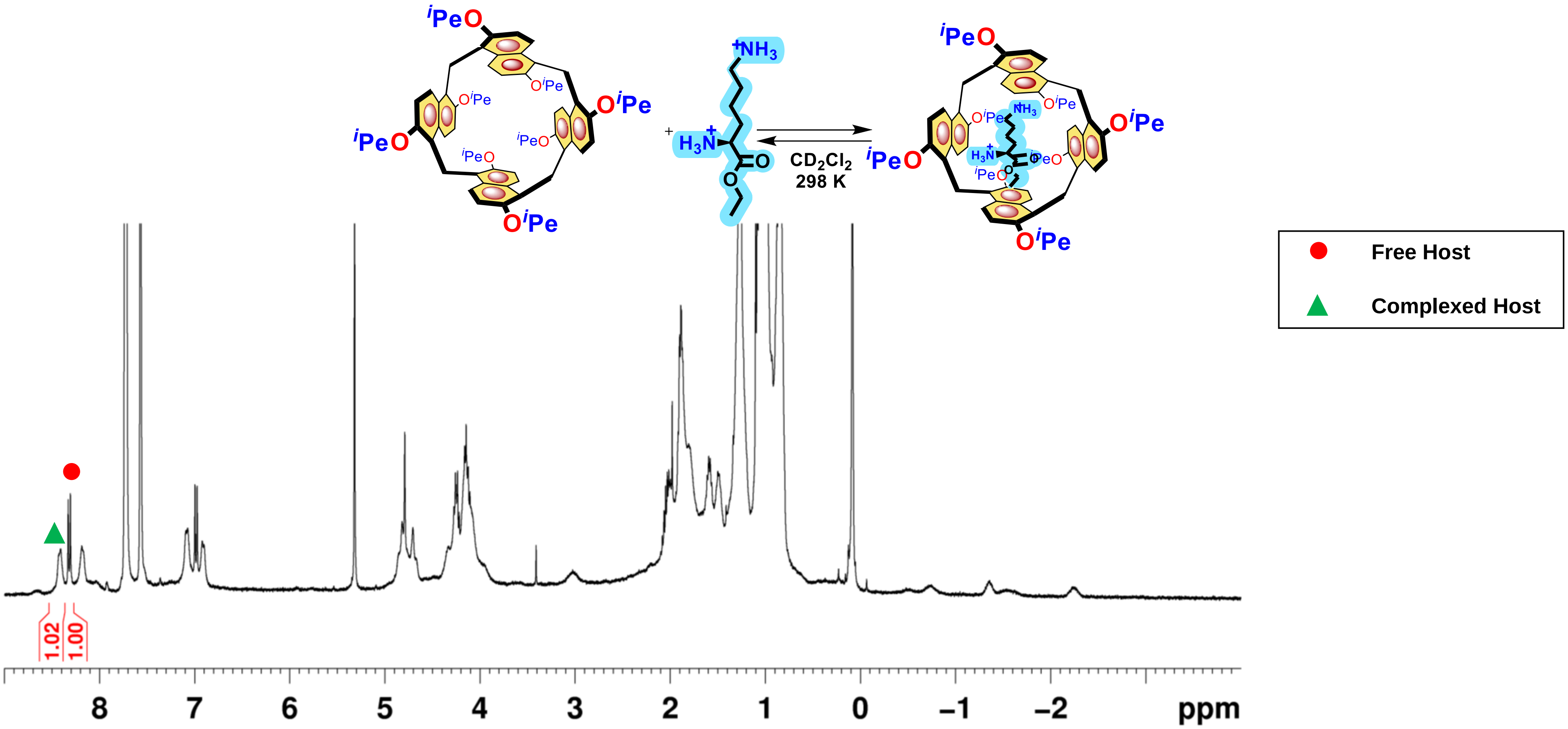

Figure S53: ^{1}H NMR spectrum (400 MHz, CD_2Cl_2, 298 K) of an equimolar solution (3.20 mM) of **PrS[4]^{iPe}** and (S)-**6^{2+}·BArF$^-$** in 0.5 mL of CD_2Cl_2.

$$K_{ass} = \frac{\left\{\frac{1.02}{1.52} \cdot 3.20 \cdot 10^{-3} M\right\}}{\left\{\frac{0.50}{1.52} \cdot 3.20 \cdot 10^{-3} M\right\}^2} = \mathbf{1900\ M^{-1}}$$

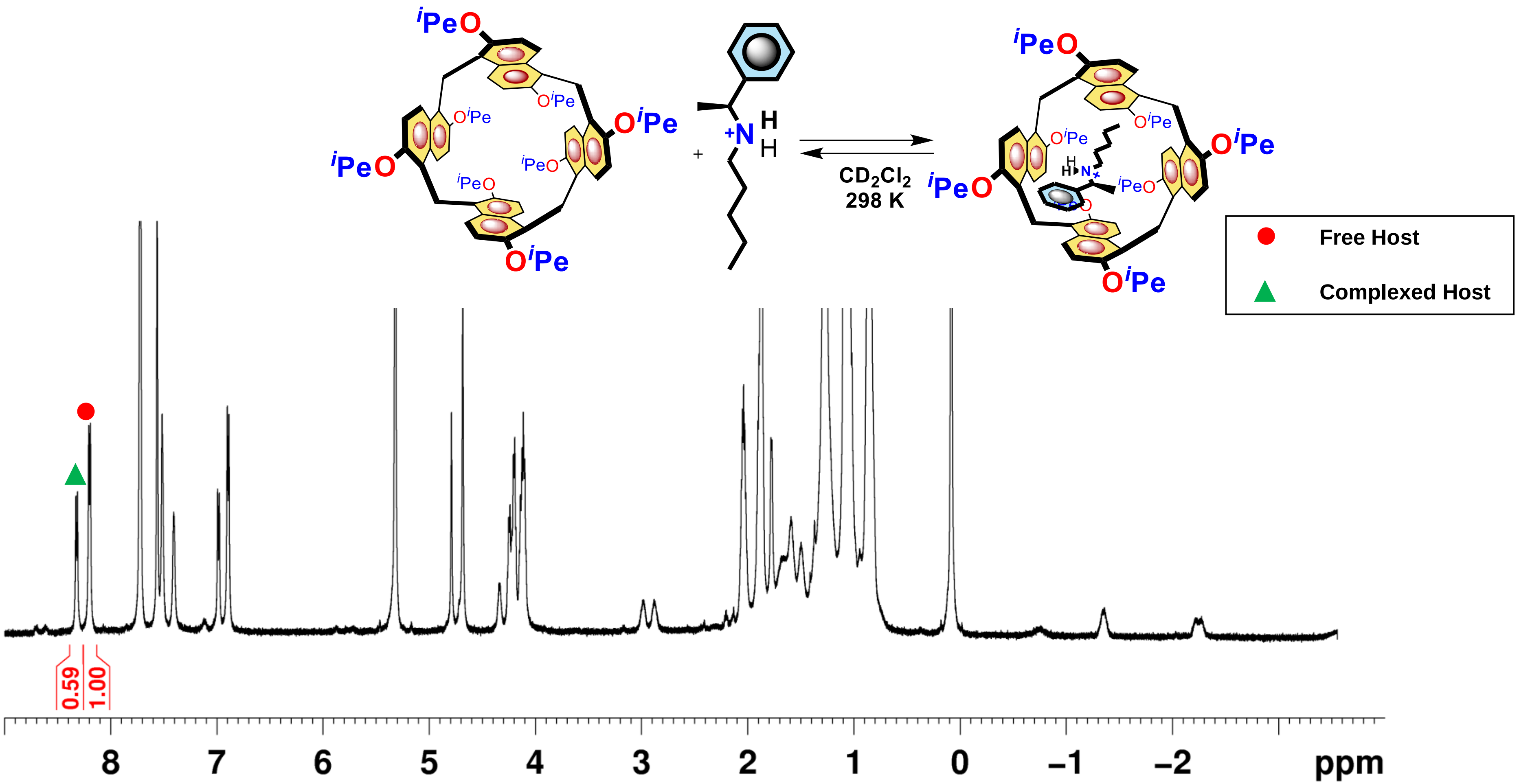

Figure S54: ^{1}H NMR spectrum (400 MHz, CD_2Cl_2,298 K) of an equimolar solution (3.20 mM) of **PrS[4]^{iPe}** and (*S*)-**8^{+}·BArF**$^{-}$ in 0.5 mL of CD_2Cl_2.

$$K_{ass} = \frac{\left\{\frac{0.59}{1.59} \cdot 3.20 \cdot 10^{-3} M\right\}}{\left\{\frac{1.00}{1.59} \cdot 3.20 \cdot 10^{-3} M\right\}^2} = \mathbf{290\ M^{-1}}$$

DFT - optimized structures of Complexes

Conformational studies have been performed using the DFT method incorporated in the Gaussian 16 package[2] and using B97D3/SVP/SVPFIT level of theory. The starting structure for DFT calculations was obtained by molecular mechanics calculation performed by YASARA software.[3] All optimized structures were characterized by 0 imaginary frequency**.**

Cartesian coordinates of **2⁺ @ PrS[4]*iPe***

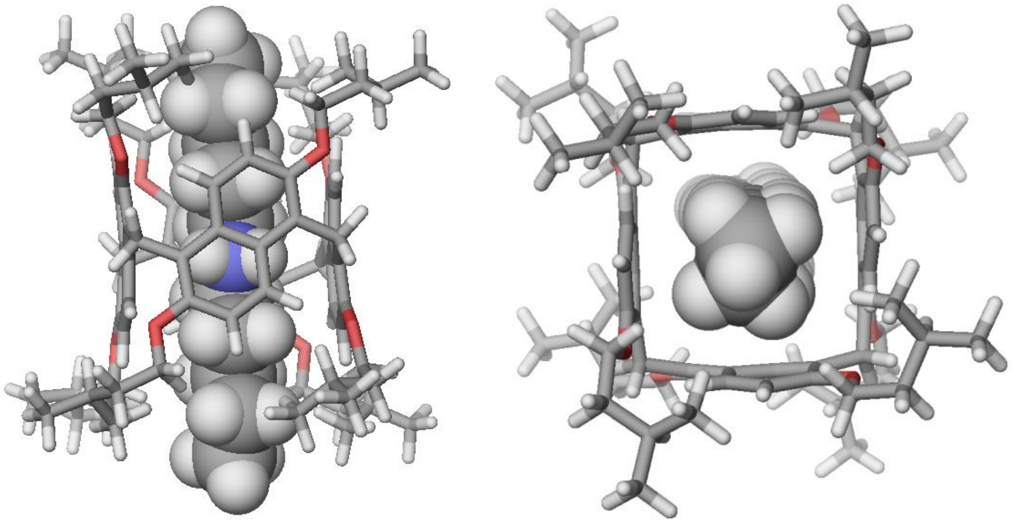

Figure S55: Side and top view of DFT-optimized structure (B97D3/SVP/SVPFIT) of the **2⁺ @ PrS[4]*iPe*** complex.

C	0.66117200	-0.68492900	-3.83596200
C	2.03695900	-0.94542600	-3.74186100
C	2.51927200	-1.97823600	-2.89160000
C	1.65304300	-2.76992600	-2.16156200
C	0.24123000	-2.62859700	-2.28863100
C	-0.24506000	-1.58202800	-3.16803700
C	-0.66783000	-3.52752000	-1.62272600
C	-2.03195700	-3.45175500	-1.94920400
C	-2.51115100	-2.42834700	-2.81305000
C	-1.65063100	-1.50508300	-3.37581700
O	-2.87353800	-4.37771800	-1.39959900
O	2.88761400	-0.18154400	-4.49004700
C	-4.24120300	-4.43718100	-1.82350900
C	-4.93266500	-5.58338000	-1.10064900
C	-5.08253800	-5.43613900	0.42752400
C	-5.70610100	-6.70798500	1.02048800
C	-5.89777000	-4.19353300	0.81758800
C	4.27014400	-0.10199500	-4.12360500
C	4.91954700	1.02319900	-4.91805000
C	6.36375200	1.33189700	-4.46985400
C	6.40581900	2.02977100	-3.09901000
C	7.08736800	2.17770500	-5.52773700
C	0.14802300	0.52894200	-4.61099300
H	3.59151600	-2.15957000	-2.80986600
H	2.07079200	-3.54218000	-1.51535100
H	-3.57542400	-2.35515600	-3.03730200
H	-2.06787600	-0.72591700	-4.01391200
H	-4.27546900	-4.59516000	-2.91968100
H	-4.74277000	-3.47290200	-1.60968800
H	-4.38532000	-6.51705200	-1.32852200
H	-5.93808300	-5.69322600	-1.55344400
H	-4.06813000	-5.32945000	0.85727800
H	-6.72744100	-6.86802600	0.62621000
H	-5.10728400	-7.60275700	0.77321700
H	-5.77786800	-6.64235300	2.12080000
H	-6.91507000	-4.23495400	0.38438300
H	-6.00917300	-4.12592200	1.91481700
H	-5.42722000	-3.25567500	0.47389000
H	4.77771500	-1.06468800	-4.33467400
H	4.34377300	0.08353500	-3.03496900
H	4.30397200	1.93800800	-4.82212900
H	4.90534200	0.74934300	-5.98880200
H	6.90525400	0.36678100	-4.37877700
H	5.92071600	3.02260000	-3.15783700
H	5.89512000	1.45003900	-2.31082100
H	7.44654000	2.18920900	-2.76584000
H	6.56597100	3.14154000	-5.68008200
H	8.12412400	2.40204400	-5.22003000
H	7.12609400	1.65914900	-6.50214000
C	0.29776000	0.52990100	3.74847900
C	1.68210200	0.72659300	3.86637700
C	2.32611200	1.76373600	3.13336200
C	1.60813300	2.60586400	2.30477800
C	0.19054300	2.51693000	2.20180700
C	-0.46326300	1.47131500	2.96626800
C	-0.57113900	3.45982700	1.42411700
C	-1.96993500	3.44518300	1.54987100
C	-2.61544700	2.43846600	2.31701100
C	-1.88747500	1.46175400	2.97120100
O	-2.67320800	4.41982900	0.90476900
O	2.37387500	-0.11893500	4.68678800
C	-4.09990500	4.46706600	0.99097300
C	-4.59385400	5.61534200	0.12207600
C	-4.01344400	7.00499600	0.45472500
C	-4.26329000	7.41013800	1.91453600
C	-4.57342200	8.05280200	-0.51786600
C	3.73532300	0.16449300	5.02852100
C	4.19490100	-0.83453600	6.08088200
C	4.21147700	-2.31791600	5.65828600
C	4.62344100	-3.19810800	6.84733500
C	5.11905500	-2.57323800	4.44556600
C	-0.37934100	-0.64573300	4.45288100
H	3.40813500	1.89751700	3.20818100
H	2.14280000	3.37393100	1.74663100
H	-3.70281200	2.42164700	2.39330500
H	-2.42786200	0.70516500	3.53895900
H	-4.40179500	4.59976600	2.04839600
H	-4.53172900	3.50894300	0.63731900

H	-5.69781800	5.64398000	0.21635200
H	-4.37908000	5.37600400	-0.93750300
H	-2.91797000	6.94758600	0.30167700
H	-3.88006600	8.42741800	2.10905700
H	-5.34557100	7.41225700	2.14718300
H	-3.76162300	6.73094500	2.62635900
H	-5.66888600	8.15632400	-0.39981400
H	-4.12278100	9.04488000	-0.33793600
H	-4.37384700	7.77656200	-1.56936100
H	3.80686600	1.19700300	5.42379600
H	4.36949000	0.10813200	4.12166000
H	3.55298700	-0.71693200	6.97395600
H	5.21604400	-0.53390000	6.38906200
H	3.17812600	-2.59827300	5.37680600
H	5.65509000	-2.96504600	7.17233600
H	3.95603300	-3.04304400	7.71394000
H	4.58970300	-4.26974100	6.58167200
H	6.16141100	-2.26946800	4.65925700
H	5.13441300	-3.64749700	4.18744700
H	4.78588100	-2.02181700	3.54983600
C	0.34818100	-3.96969100	0.68320800
C	1.69927300	-4.06909900	1.04794200
C	2.17968500	-3.40988500	2.21461500
C	1.35197000	-2.59930900	2.96833800
C	-0.03774900	-2.49416500	2.68110600
C	-0.54470100	-3.27664700	1.57288700
C	-0.92376300	-1.68620300	3.47909200
C	-2.30742700	-1.87005700	3.32470100
C	-2.80990000	-2.72554300	2.30690900
C	-1.95704600	-3.35981100	1.42429900
O	-3.13993300	-1.18161600	4.16218300
O	2.52662900	-4.78160300	0.22621800
C	-4.54959100	-1.43901400	4.13338500
C	-5.22964100	-0.57141200	5.18199400
C	-5.19781400	0.95072000	4.93360200
C	-5.80651600	1.69354100	6.13181700
C	-5.90869200	1.34351700	3.62921400
C	3.88479500	-5.02058000	0.61466500
C	4.57661800	-5.82114500	-0.47830100
C	4.74495300	-5.11384700	-1.83902700
C	5.39517200	-6.06864700	-2.85076300
C	5.54619500	-3.80751800	-1.72466600
C	-0.16296700	-4.56950700	-0.62413600
H	3.22868700	-3.49471800	2.50003300
H	1.77973500	-2.04743400	3.80554600
H	-3.88464100	-2.85965300	2.18393000
H	-2.38768000	-3.95861700	0.62248500
H	-4.72710800	-2.51186100	4.34607800
H	-4.94929600	-1.22958100	3.12160100
H	-4.77118000	-0.79422500	6.16348600
H	-6.28418800	-0.90520800	5.24765500
H	-4.13738200	1.25741500	4.85279400
H	-6.87143000	1.42410900	6.26430800
H	-5.27848700	1.44492000	7.06971900
H	-5.75230300	2.78805400	5.99291100
H	-6.96913100	1.02842000	3.64492900
H	-5.89234800	2.43910200	3.48691500
H	-5.43895300	0.88750900	2.73988200
H	3.89686300	-5.57994800	1.57117500
H	4.40207100	-4.05651700	0.78585600
H	4.02131500	-6.76676100	-0.62225800
H	5.57599800	-6.10239300	-0.09089300
H	3.73464200	-4.86380100	-2.21558100
H	6.41643800	-6.34879900	-2.53011300
H	4.81053700	-6.99974500	-2.95849400
H	5.47309500	-5.60102300	-3.84849500
H	6.54636000	-3.99101900	-1.28844000
H	5.70062500	-3.35370500	-2.72031100
H	5.03883300	-3.05662300	-1.09422600
C	0.69938300	3.78275900	-0.75879600
C	2.09080200	3.68352100	-0.91218500
C	2.64713000	2.93322500	-1.98477600
C	1.83310300	2.25088500	-2.86707100
C	0.41645100	2.33779400	-2.79074600
C	-0.14250500	3.19538700	-1.76924000
C	-0.42826400	1.61717100	-3.70467400
C	-1.79764000	1.92234500	-3.72321200
C	-2.33771000	2.85752600	-2.79569100
C	-1.54328200	3.43953500	-1.82441800
O	-2.58059000	1.27383800	-4.64004500
O	2.88026600	4.32350000	0.00326400
C	-3.93299100	1.69266600	-4.85040500
C	-4.53400400	0.86520200	-5.97745500
C	-4.65769000	-0.65108600	-5.72196500
C	-5.22257100	-1.35001200	-6.96713600
C	-5.50652600	-0.96912600	-4.48167300
C	4.29515400	4.11486300	-0.01567600
C	4.89994000	4.77845900	1.21350600
C	6.37005400	4.38590200	1.46303600
C	6.88573500	5.04750500	2.74901400
C	7.28102700	4.71217400	0.26855600
C	0.10496700	4.45344200	0.47869400
H	3.72908300	2.87250900	-2.11204000
H	2.29173000	1.66179700	-3.65825000
H	-3.40141900	3.10538200	-2.82193300
H	-2.00714800	4.11103100	-1.10221200
H	-3.94588800	2.76865900	-5.11453100
H	-4.51546400	1.57164400	-3.91566800
H	-3.93368500	1.03350900	-6.89097400
H	-5.54019800	1.28101400	-6.18421100
H	-3.63855100	-1.04689400	-5.54890900
H	-6.24859600	-0.99860900	-7.18627900
H	-4.60174200	-1.14824100	-7.85828000
H	-5.26590600	-2.44480300	-6.82604500
H	-6.52371900	-0.54387100	-4.57515300
H	-5.61762900	-2.06078600	-4.35099100
H	-5.06030900	-0.56841500	-3.55546300
H	4.71842900	4.53096400	-0.94970900
H	4.51191200	3.02760900	-0.00634500
H	4.29546900	4.49239000	2.09480300
H	4.81320100	5.87794900	1.11910700
H	6.39537200	3.28530600	1.61537000
H	6.89402100	6.14878000	2.64714600
H	6.24862500	4.79689400	3.61669700
H	7.91669900	4.72458200	2.97853100
H	7.23151800	5.78911000	0.01955500
H	8.33377300	4.47157900	0.50036200
H	7.00613900	4.14269700	-0.63708700
H	-0.63547700	5.21078200	0.18204800
H	0.90728800	4.99072800	1.00524300
H	-0.98254900	-5.27512500	-0.41865400
H	0.64866700	-5.15805300	-1.07745800
H	-0.62778200	0.22262600	-5.32811300
H	0.97919400	0.93574900	-5.20631300
H	-1.20957300	-0.28252800	5.07720600
H	0.34909300	-1.10591800	5.13681700
C	0.94453600	0.16581900	-0.26881000
H	0.99903900	0.03466700	-1.35731400
H	0.91338500	1.24256800	-0.05353400
N	-0.38434000	-0.39024000	0.15960400
C	-1.55060200	0.35213100	-0.42005000
H	-1.47379000	1.38238600	-0.04853800
H	-1.39475400	0.36975200	-1.50753700
C	-2.88210000	-0.27803800	-0.05776300
H	-2.90332900	-1.32628400	-0.40133700
H	-2.99561300	-0.29933900	1.03954600
C	2.08678000	-0.51985600	0.45073400
H	1.94784100	-0.41473000	1.53813200
H	2.06247900	-1.59849800	0.22983000
C	-4.04353000	0.49549600	-0.68247200
H	-4.02332200	1.53812700	-0.31222100
H	-3.88848900	0.56605000	-1.77533500
C	3.44814500	0.04909000	0.05917400
H	3.60894200	-0.09719500	-1.02497300
H	3.44885700	1.14220800	0.22146700
C	-5.41169500	-0.13018600	-0.39603000
H	-5.45267700	-1.13500200	-0.86068000
H	-5.51355400	-0.30067500	0.69379900

C	4.59349100	-0.58922200	0.84890900
H	4.44253200	-0.37421700	1.92391100
H	4.52555500	-1.69037000	0.75797800
C	-6.58032500	0.72222200	-0.89530800
H	-7.55311000	0.24162800	-0.69091600
H	-6.58647900	1.71344000	-0.40438100
H	-6.51414800	0.89207400	-1.98561400
C	5.98162700	-0.12006600	0.41134800
H	6.77770000	-0.57255500	1.02875900
H	6.18121800	-0.39311400	-0.64109700
H	6.07893400	0.97846100	0.49179100
H	-0.43477300	-1.39033100	-0.10387000
H	-0.44566500	-0.37624300	1.19323200

1 2 1.5 6 1.5 23 1.0
2 3 1.5 12 1.0
3 4 2.0 24 1.0
4 5 1.5 25 1.0
5 6 1.0 7 1.5
6 10 1.5
7 8 1.5 121 1.0
8 9 1.5 11 1.0
9 10 2.0 26 1.0
10 27 1.0
11 13 1.0
12 18 1.0
13 14 1.0 28 1.0 29 1.0
14 15 1.0 30 1.0 31 1.0
15 16 1.0 17 1.0 32 1.0
16 33 1.0 34 1.0 35 1.0
17 36 1.0 37 1.0 38 1.0
18 19 1.0 39 1.0 40 1.0
19 20 1.0 41 1.0 42 1.0
20 21 1.0 22 1.0 43 1.0
21 44 1.0 45 1.0 46 1.0
22 47 1.0 48 1.0 49 1.0
23 154 1.0 201 1.0 202 1.0
24
25
26
27
28
29
30
31
32
33
34
35
36
37
38
39
40
41
42
43
44
45
46
47
48
49
50 51 1.5 55 1.5 72 1.0
51 52 1.5 61 1.0
52 53 2.0 73 1.0
53 54 1.5 74 1.0
54 55 1.0 56 1.5
55 59 1.5
56 57 1.5 170 1.0
57 58 1.5 60 1.0
58 59 2.0 75 1.0
59 76 1.0
60 62 1.0
61 67 1.0
62 63 1.0 77 1.0 78 1.0
63 64 1.0 79 1.0 80 1.0
64 65 1.0 66 1.0 81 1.0
65 82 1.0 83 1.0 84 1.0
66 85 1.0 86 1.0 87 1.0
67 68 1.0 88 1.0 89 1.0
68 69 1.0 90 1.0 91 1.0
69 70 1.0 71 1.0 92 1.0
70 93 1.0 94 1.0 95 1.0
71 96 1.0 97 1.0 98 1.0
72 105 1.0 203 1.0 204 1.0
73
74
75
76
77
78
79
80
81
82
83
84
85
86
87
88
89
90
91
92
93
94
95
96
97
98
99 100 1.5 104 1.5 121 1.0
100 101 1.5 110 1.0
101 102 2.0 122 1.0
102 103 1.5 123 1.0
103 104 1.0 105 1.5
104 108 1.5
105 106 1.5
106 107 1.5 109 1.0
107 108 2.0 124 1.0
108 125 1.0
109 111 1.0
110 116 1.0
111 112 1.0 126 1.0 127 1.0
112 113 1.0 128 1.0 129 1.0
113 114 1.0 115 1.0 130 1.0
114 131 1.0 132 1.0 133 1.0
115 134 1.0 135 1.0 136 1.0
116 117 1.0 137 1.0 138 1.0
117 118 1.0 139 1.0 140 1.0
118 119 1.0 120 1.0 141 1.0
119 142 1.0 143 1.0 144 1.0
120 145 1.0 146 1.0 147 1.0
121 199 1.0 200 1.0
122
123
124
125
126
127
128
129
130
131
132
133
134

135
136
137
138
139
140
141
142
143
144
145
146
147
148 149 1.5 153 1.5 170 1.0
149 150 1.5 159 1.0
150 151 2.0 171 1.0
151 152 1.5 172 1.0
152 153 1.5 154 1.5
153 157 1.5
154 155 1.5
155 156 1.5 158 1.0
156 157 2.0 173 1.0
157 174 1.0
158 160 1.0
159 165 1.0
160 161 1.0 175 1.0 176 1.0
161 162 1.0 177 1.0 178 1.0
162 163 1.0 164 1.0 179 1.0
163 180 1.0 181 1.0 182 1.0
164 183 1.0 184 1.0 185 1.0
165 166 1.0 186 1.0 187 1.0
166 167 1.0 188 1.0 189 1.0
167 168 1.0 169 1.0 190 1.0
168 191 1.0 192 1.0 193 1.0
169 194 1.0 195 1.0 196 1.0
170 197 1.0 198 1.0
171
172
173
174
175
176
177
178
179
180
181
182
183
184
185
186
187
188
189
190
191
192
193
194
195
196
197
198
199
200
201
202
203
204
205 206 1.0 207 1.0 208 1.0 215 1.0
206
207
208 209 1.0 238 1.0 239 1.0
209 210 1.0 211 1.0 212 1.0
210
211
212 213 1.0 214 1.0 218 1.0
213
214
215 216 1.0 217 1.0 221 1.0
216
217
218 219 1.0 220 1.0 224 1.0
219
220
221 222 1.0 223 1.0 227 1.0
222
223
224 225 1.0 226 1.0 230 1.0
225
226
227 228 1.0 229 1.0 234 1.0
228
229
230 231 1.0 232 1.0 233 1.0
231
232
233
234 235 1.0 236 1.0 237 1.0
235
236
237
238
239

---

0 imaginary frequency
Energy: -4314.7737 Hartree

Cartesian coordinates of **5**$^{+}$ @ **PrS[4]**^{iPe}

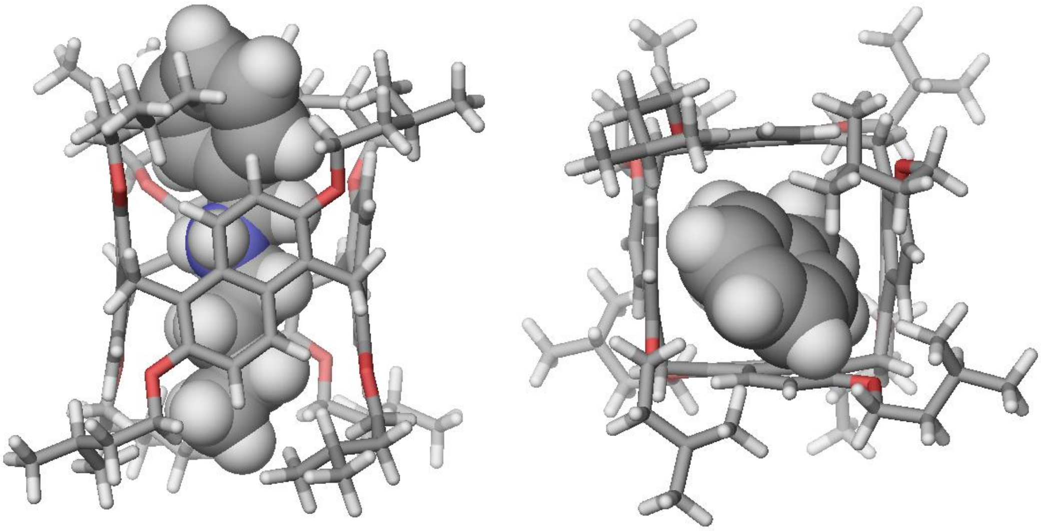

Figure S56: Side and top view of DFT-optimized structure (B97D3/SVP/SVPFIT) of the **5**$^{+}$ @ **PrS[4]**^{iPe} complex.

C	0.74207900	2.58044600	2.74780700
C	2.13315500	2.61800700	2.56807800
C	2.69095700	2.99486800	1.31705000
C	1.88616700	3.33280500	0.24378100
C	0.46930900	3.41121200	0.38230200
C	-0.09141600	3.05968900	1.67442200
C	-0.36406600	3.88489800	-0.69237300
C	-1.71175700	4.15680200	-0.41445900
C	-2.25833300	3.84854800	0.86174600
C	-1.48553600	3.27691400	1.85455800
O	-2.47214000	4.69622500	-1.41270800
O	2.92752000	2.26424500	3.62545400
C	-3.79515500	5.16692700	-1.12675900
C	-4.36706800	5.81424900	-2.37947200
C	-4.58854700	4.88425900	-3.59005600
C	-5.07237200	5.69980400	-4.79797700
C	-5.56202100	3.73740200	-3.27789000
C	4.29672500	2.68725400	3.66577900
C	4.85981700	2.38255500	5.04668000
C	4.84361000	0.90160800	5.47953300
C	5.40169900	0.76590100	6.90384000
C	5.60219700	-0.01049000	4.50399700
C	0.15169900	2.07287000	4.06470900
H	3.77227000	3.01160800	1.18500800
H	2.36175000	3.59812600	-0.70026400
H	-3.31323100	4.03459500	1.06230000
H	-1.95857900	3.02141900	2.80245100
H	-3.74926600	5.90319600	-0.29993900
H	-4.42908300	4.32403000	-0.78852400
H	-3.70077900	6.64660000	-2.67393200
H	-5.33410600	6.27455900	-2.09496700
H	-3.61005500	4.44047900	-3.85664900
H	-6.05245000	6.16940100	-4.59021900
H	-4.36104500	6.50647200	-5.05056400
H	-5.18979900	5.06024000	-5.69092500
H	-5.19566900	3.08281500	-2.46796100
H	-6.55018200	4.12947000	-2.97121500
H	-5.72062900	3.10453700	-4.16970700
H	4.34946400	3.77347900	3.45467200
H	4.87795500	2.16849000	2.87954600
H	4.30424500	2.98310500	5.79097400
H	5.90347800	2.75518300	5.06052200
H	3.78707900	0.57192100	5.50142200
H	6.46374200	1.07369100	6.94305100
H	4.84307200	1.39760200	7.61751700
H	5.34207600	-0.27860300	7.25805700
H	6.65738900	0.30655200	4.40015000

H	5.60563300	-1.05435500	4.86555000
H	5.15300200	-0.01599900	3.49664500
C	-0.14642800	-2.68956500	-2.72863000
C	1.18232900	-3.11910000	-2.69249400
C	1.72911100	-3.73311900	-1.53338500
C	0.97452300	-3.89257300	-0.38763100
C	-0.40089800	-3.50954900	-0.35964400
C	-0.96843800	-2.94739500	-1.57123600
C	-1.20843100	-3.70120200	0.81544700
C	-2.59917300	-3.54626100	0.68746400
C	-3.16647900	-3.04367300	-0.51464600
C	-2.36924000	-2.71004600	-1.59312100
O	-3.37349100	-3.88634800	1.75625400
O	2.00822800	-2.89174200	-3.77168600
C	-4.77437800	-3.59027000	1.76020400
C	-5.33909100	-3.96263300	3.12403500
C	-5.12503000	-5.42686000	3.55911800
C	-5.70474300	-6.42899900	2.55035800
C	-5.71039100	-5.64710900	4.96134300
C	2.48730300	-4.04872900	-4.48176500
C	3.49015100	-3.59171600	-5.52927800
C	4.70664100	-2.81843600	-4.98135700
C	5.61118300	-2.36043500	-6.13408900
C	5.49643300	-3.63020400	-3.94457200
C	-0.70450500	-2.01017800	-3.97883300
H	2.77604400	-4.04714400	-1.54916300
H	1.44166200	-4.31893200	0.50127800
H	-4.24445500	-2.89948400	-0.59363800
H	-2.84109100	-2.29444600	-2.48337500
H	-5.27536500	-4.15660000	0.95119200
H	-4.92595300	-2.51017400	1.55962900
H	-6.42463000	-3.74033500	3.09887600
H	-4.90184900	-3.28988100	3.88689900
H	-4.03175700	-5.59536500	3.61416800
H	-5.57916000	-7.46481100	2.91215600
H	-6.78794800	-6.25877000	2.39811800
H	-5.20660200	-6.36465800	1.56679600
H	-6.80696800	-5.49788900	4.95984300
H	-5.51340800	-6.67324900	5.31904900
H	-5.27706100	-4.94393800	5.69588100
H	1.62510900	-4.56046000	-4.95462700
H	2.94480100	-4.76104500	-3.76851000
H	2.96489300	-2.96288100	-6.27305600
H	3.83834400	-4.49264200	-6.07198600
H	4.31165200	-1.91314300	-4.47913200
H	6.03989300	-3.22869300	-6.66900700
H	5.05177800	-1.75615600	-6.87096700

H	6.45381300	-1.74908500	-5.76328900
H	5.84160500	-4.59007500	-4.37281200
H	6.39184700	-3.07943900	-3.60559700
H	4.89473400	-3.85622500	-3.04742000
C	0.56957900	2.75981400	-2.76956400
C	1.91819600	2.44455900	-3.00783700
C	2.27924300	1.15279200	-3.47704400
C	1.32263100	0.17384900	-3.68984900
C	-0.06595500	0.45483400	-3.53917000
C	-0.43212200	1.80294400	-3.15350700
C	-1.07285500	-0.54062600	-3.79953100
C	-2.40868900	-0.12309300	-3.88664500
C	-2.76186300	1.22906200	-3.61671000
C	-1.81208800	2.14515700	-3.20979400
O	-3.34628200	-1.06601800	-4.20095400
O	2.84458800	3.41722500	-2.76717700
C	-4.70379400	-0.67651700	-4.44357000
C	-5.50702600	-1.91563100	-4.81078900
C	-5.63737000	-2.99112400	-3.71260000
C	-6.37832500	-4.21849700	-4.26275200
C	-6.32788000	-2.45624900	-2.44878800
C	4.22807700	3.18442500	-3.06399800
C	4.99147900	4.48381800	-2.84719800
C	5.03922200	5.01406100	-1.40014400
C	5.66546100	6.41552800	-1.36787100
C	5.78612900	4.05637400	-0.46111600
C	0.19071200	4.07951800	-2.10253200
H	3.32736300	0.91004800	-3.65494200
H	1.64924600	-0.81298300	-4.02162000
H	-3.80247600	1.54750900	-3.68456600
H	-2.13569200	3.15040700	-2.94028200
H	-4.72983800	0.06025000	-5.27079500
H	-5.11987900	-0.18333900	-3.54352600
H	-5.05436400	-2.36675700	-5.71359000
H	-6.51856400	-1.57338500	-5.10661900
H	-4.61533300	-3.31286100	-3.43479400
H	-7.41229600	-3.95697200	-4.55735600
H	-5.87017800	-4.63006200	-5.15321900
H	-6.43824700	-5.02119800	-3.50620600
H	-7.34292900	-2.08162800	-2.68023700
H	-6.43606700	-3.25548300	-1.69343400
H	-5.76609400	-1.62990500	-1.97864900
H	4.32687600	2.86304700	-4.11945500
H	4.61215500	2.36615400	-2.42349300
H	4.54678900	5.25295400	-3.50607000
H	6.02601000	4.32275300	-3.21017400
H	3.99574000	5.10378100	-1.04075000
H	6.70973200	6.38959100	-1.73285400
H	5.10496200	7.12270500	-2.00525400
H	5.67981100	6.82307800	-0.34121200
H	6.84171600	3.94200800	-0.77313100
H	5.78587600	4.43892000	0.57553400
H	5.33537600	3.04997600	-0.44799400
C	0.15294700	-2.85356700	2.80983900
C	1.54761900	-2.88096700	2.96719200
C	2.23336500	-1.74812800	3.49039900
C	1.56177700	-0.57031300	3.76913800
C	0.14569500	-0.48371200	3.65543700
C	-0.55772900	-1.68721700	3.26425800
C	-0.57139400	0.73317900	3.93753800
C	-1.96725700	0.66531300	4.06542800
C	-2.65377300	-0.55477400	3.81362200
C	-1.97425800	-1.67543800	3.37778500
O	-2.63352900	1.80817300	4.41680400
O	2.21118900	-4.01254500	2.58181200
C	-4.02542000	1.74352500	4.74776000
C	-4.50701600	3.12976400	5.14926500
C	-4.50701100	4.20301400	4.04134700
C	-4.96237500	5.55214800	4.61647100
C	-5.37230300	3.80051300	2.83675000
C	3.49272500	-4.34185300	3.14178000
C	4.69122500	-3.77269700	2.37954100
C	4.67897100	-3.96376300	0.85108500
C	4.46060800	-5.42450400	0.43236200

C	5.97564900	-3.40542600	0.24995700
C	-0.58643900	-4.03424400	2.17285900
H	3.31562000	-1.77897500	3.62277300
H	2.13825300	0.29686400	4.09154600
H	-3.73901900	-0.60965000	3.92558300
H	-2.54201200	-2.57533300	3.14850200
H	-4.16662300	1.03050500	5.58416200
H	-4.60082400	1.36164100	3.88161800
H	-3.89265500	3.47930800	6.00001800
H	-5.53847500	3.01315500	5.53734700
H	-3.46468300	4.32508800	3.69022600
H	-6.00632000	5.49546700	4.97870800
H	-4.32892100	5.86154800	5.46702100
H	-4.91534200	6.34879500	3.85253300
H	-6.41722000	3.60863000	3.14606400
H	-5.39727400	4.60897900	2.08375900
H	-4.99809400	2.89134600	2.33345700
H	3.52005600	-5.44446300	3.11880000
H	3.52152200	-4.03273100	4.20427400
H	5.59065700	-4.26511400	2.80183300
H	4.81173000	-2.69499900	2.59168100
H	3.83889500	-3.36074600	0.45630200
H	5.23088100	-6.08264200	0.87714400
H	3.47094700	-5.80580000	0.73683500
H	4.53443200	-5.53356700	-0.66493400
H	6.84987200	-3.99481400	0.58524600
H	5.95086000	-3.43590800	-0.85263000
H	6.13610500	-2.35626000	0.55002600
H	-1.38685900	-4.37754300	2.84545100
H	0.12014400	-4.87137600	2.07687300
H	-0.56395800	4.60938100	-2.70214200
H	1.08015300	4.72659300	-2.07579000
H	-0.55836400	2.81233000	4.46396700
H	0.96928800	1.99492000	4.79693300
H	-1.60099000	-2.54981500	-4.31921400
H	0.04750700	-2.10814300	-4.77746900
C	1.88339200	-0.66605500	0.30187900
H	1.79213700	-0.46542500	1.37568000
H	1.60159100	-1.71305700	0.12795500
N	0.79610400	0.15702200	-0.35051100
C	-0.51740100	-0.05217900	0.36923000
H	-0.61156000	-1.13566700	0.51735100
H	-0.39145300	0.41441700	1.35405300
C	-1.71424400	0.50909600	-0.36558600
H	-1.54854200	1.57044700	-0.59865700
H	-1.83403700	-0.02004000	-1.32342700
C	-2.99655400	0.36657900	0.45978500
H	-3.12594700	-0.68870100	0.75643000
H	-2.88723400	0.93568700	1.39862400
C	-4.22966100	0.84991300	-0.30150800
H	-4.11878200	1.90658900	-0.60166900
H	-4.38030800	0.26395900	-1.22525000
H	1.04330700	1.16477600	-0.33171600
H	0.70234600	-0.08320300	-1.35528000
H	-5.14550900	0.76364500	0.31034200
C	5.96575100	0.09404100	-0.90280700
C	5.08789100	-0.54309900	-1.79160700
C	3.76149900	-0.80021300	-1.41758000
C	3.28945000	-0.39412200	-0.15533900
C	4.18803600	0.21108900	0.74301900
C	5.51771800	0.45393100	0.37684800
H	7.00347300	0.28720800	-1.19706200
H	5.44086100	-0.85657400	-2.77904600
H	3.10062300	-1.32549400	-2.11024200
H	3.83161000	0.47940100	1.74046200
H	6.20579400	0.92097500	1.09018400

1 2 1.5 6 1.5 23 1.0
2 3 1.5 12 1.0
3 4 2.0 24 1.0
4 5 1.5 25 1.0
5 6 1.0 7 1.5
6 10 1.5
7 8 1.5 121 1.0

8 9 1.5 11 1.0
9 10 2.0 26 1.0
10 27 1.0
11 13 1.0
12 18 1.0
13 14 1.0 28 1.0 29 1.0
14 15 1.0 30 1.0 31 1.0
15 16 1.0 17 1.0 32 1.0
16 33 1.0 34 1.0 35 1.0
17 36 1.0 37 1.0 38 1.0
18 19 1.0 39 1.0 40 1.0
19 20 1.0 41 1.0 42 1.0
20 21 1.0 22 1.0 43 1.0
21 44 1.0 45 1.0 46 1.0
22 47 1.0 48 1.0 49 1.0
23 154 1.0 201 1.0 202 1.0
24
25
26
27
28
29
30
31
32
33
34
35
36
37
38
39
40
41
42
43
44
45
46
47
48
49
50 51 1.5 55 1.5 72 1.0
51 52 1.5 61 1.0
52 53 2.0 73 1.0
53 54 1.5 74 1.0
54 55 1.0 56 1.5
55 59 1.5
56 57 1.5 170 1.0
57 58 1.5 60 1.0
58 59 2.0 75 1.0
59 76 1.0
60 62 1.0
61 67 1.0
62 63 1.0 77 1.0 78 1.0
63 64 1.0 79 1.0 80 1.0
64 65 1.0 66 1.0 81 1.0
65 82 1.0 83 1.0 84 1.0
66 85 1.0 86 1.0 87 1.0
67 68 1.0 88 1.0 89 1.0
68 69 1.0 90 1.0 91 1.0
69 70 1.0 71 1.0 92 1.0
70 93 1.0 94 1.0 95 1.0
71 96 1.0 97 1.0 98 1.0
72 105 1.0 203 1.0 204 1.0
73
74
75
76
77
78
79
80
81
82
83
84
85
86
87
88
89
90
91
92
93
94
95
96
97
98
99 100 1.5 104 1.5 121 1.0
100 101 1.5 110 1.0
101 102 2.0 122 1.0
102 103 1.5 123 1.0
103 104 1.0 105 1.5
104 108 1.5
105 106 1.5
106 107 1.5 109 1.0
107 108 2.0 124 1.0
108 125 1.0
109 111 1.0
110 116 1.0
111 112 1.0 126 1.0 127 1.0
112 113 1.0 128 1.0 129 1.0
113 114 1.0 115 1.0 130 1.0
114 131 1.0 132 1.0 133 1.0
115 134 1.0 135 1.0 136 1.0
116 117 1.0 137 1.0 138 1.0
117 118 1.0 139 1.0 140 1.0
118 119 1.0 120 1.0 141 1.0
119 142 1.0 143 1.0 144 1.0
120 145 1.0 146 1.0 147 1.0
121 199 1.0 200 1.0
122
123
124
125
126
127
128
129
130
131
132
133
134
135
136
137
138
139
140
141
142
143
144
145
146
147
148 149 1.5 153 1.5 170 1.0
149 150 1.5 159 1.0
150 151 2.0 171 1.0
151 152 1.5 172 1.0
152 153 1.0 154 1.5
153 157 1.5
154 155 1.5
155 156 1.5 158 1.0

156 157 2.0 173 1.0
157 174 1.0
158 160 1.0
159 165 1.0
160 161 1.0 175 1.0 176 1.0
161 162 1.0 177 1.0 178 1.0
162 163 1.0 164 1.0 179 1.0
163 180 1.0 181 1.0 182 1.0
164 183 1.0 184 1.0 185 1.0
165 166 1.0 186 1.0 187 1.0
166 167 1.0 188 1.0 189 1.0
167 168 1.0 169 1.0 190 1.0
168 191 1.0 192 1.0 193 1.0
169 194 1.0 195 1.0 196 1.0
170 197 1.0 198 1.0
171
172
173
174
175
176
177
178
179
180
181
182
183
184
185
186
187
188
189
190
191
192
193
194
195
196
197
198
199
200
201
202
203
204
205 206 1.0 207 1.0 208 1.0 227 1.0
206
207
208 209 1.0 221 1.0 222 1.0
209 210 1.0 211 1.0 212 1.0
210
211
212 213 1.0 214 1.0 215 1.0
213
214
215 216 1.0 217 1.0 218 1.0
216
217
218 219 1.0 220 1.0 223 1.0
219
220
221
222
223
224 225 1.5 229 1.5 230 1.0
225 226 1.5 231 1.0
226 227 1.5 232 1.0
227 228 1.5
228 229 1.5 233 1.0
229 234 1.0
230
231
232
233
234

---

0 imaginary frequency
Energy: -4349.1994 Hartree

Cartesian coordinates of **3^+ @ PrS[4]^{iPe}**

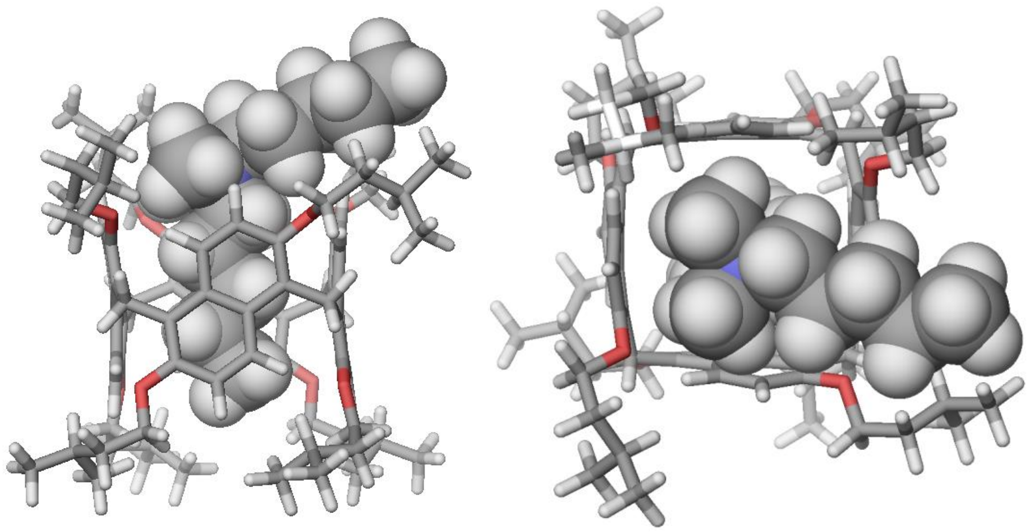

Figure S57: Side and top view of DFT-optimized structure (B97D3/SVP/SVPFIT) of the **3^+ @ PrS[4]^{iPe}** complex.

C	0.37320200	-0.11779100	-4.01137500
C	1.73975900	0.09844200	-4.24916300
C	2.71178000	-0.80656100	-3.74387100
C	2.34138200	-1.91464200	-3.00562200
C	0.97518900	-2.24837000	-2.81347900
C	-0.01380500	-1.35456900	-3.38491400

C	0.58341900	-3.45899000	-2.12809800
C	-0.76199500	-3.82511500	-2.18111300
C	-1.72056000	-3.00860400	-2.84159800
C	-1.37428400	-1.78337300	-3.37470800
O	-1.20136200	-4.96405800	-1.53379800
O	2.09658200	1.20860100	-4.96349300
C	-1.67780500	-6.03626600	-2.36172900
C	-2.22193700	-7.13651600	-1.46234000
C	-3.34238700	-6.70507500	-0.49413500
C	-3.75149600	-7.88030000	0.40531700
C	-4.56046800	-6.12112400	-1.22574400
C	3.45499000	1.37090500	-5.38587000
C	3.56184400	2.62586400	-6.24004100
C	3.31029400	3.96757500	-5.52193700
C	3.35298100	5.12228300	-6.53336400
C	4.30103800	4.20922400	-4.37277300
C	-0.65717700	0.93765100	-4.42009400
H	3.77280300	-0.61626100	-3.90526600
H	3.12225400	-2.55426200	-2.59604500
H	-2.75414700	-3.36134400	-2.91542900
H	-2.14812100	-1.16185200	-3.82693500
H	-0.84209400	-6.41432600	-2.98470500
H	-2.45766900	-5.66040400	-3.05318400
H	-1.38487000	-7.56101800	-0.87616800
H	-2.59093600	-7.95190200	-2.11531400
H	-2.91945000	-5.91302700	0.15482800
H	-4.18014000	-8.70556300	-0.19380900
H	-2.88479700	-8.28435200	0.95884000
H	-4.51388900	-7.57477600	1.14478400
H	-4.99418700	-6.86077800	-1.92467800
H	-5.35552200	-5.84154700	-0.50998700
H	-4.30724100	-5.21930700	-1.81070700
H	3.76244100	0.48178700	-5.97193600
H	4.11961500	1.43291100	-4.50187300
H	2.86091400	2.52628600	-7.09003000
H	4.58067100	2.63632000	-6.67613800
H	2.29018800	3.93555500	-5.09397600
H	4.35459000	5.20523500	-6.99637300
H	2.61962200	4.97314500	-7.34617500
H	3.12748600	6.08831300	-6.04721600
H	5.34334200	4.21755700	-4.74435600
H	4.11416300	5.18673900	-3.89264000
H	4.23037500	3.43527700	-3.58792700
C	1.46055800	0.22515900	3.62110900
C	2.70275300	0.86017300	3.45428700
C	2.79419100	2.10289900	2.77370100
C	1.66592200	2.72753700	2.27637800
C	0.36951300	2.20098500	2.51098100
C	0.27662700	0.94855700	3.23836300
C	-0.81452400	2.90990100	2.08530600
C	-2.04706600	2.44440100	2.54465700
C	-2.13911200	1.26584300	3.33097600
C	-1.02379100	0.50202500	3.62012700
O	-3.22422200	3.09902200	2.22622100
O	3.80891600	0.23454000	3.95803000
C	-3.69277500	4.01367400	3.23625100
C	-4.94705700	4.71454500	2.73904500
C	-4.78096000	5.58292700	1.47491800
C	-3.80161800	6.74709600	1.68611500
C	-6.14844300	6.10398700	1.00881700
C	5.07052200	0.91240800	3.95822800
C	6.10009500	0.03448700	4.65493900
C	6.46438800	-1.28554300	3.94506200
C	7.40352900	-2.11871100	4.82928600
C	7.08437600	-1.05027600	2.55917500
C	1.39531100	-1.18176600	4.21714800
H	3.76599300	2.56946100	2.61250000
H	1.78388800	3.66000200	1.72442100
H	-3.12899800	0.97821100	3.70312100
H	-1.13903800	-0.42958300	4.17733400
H	-2.88448300	4.73487400	3.47028900
H	-3.90796100	3.45130500	4.16816700
H	-5.32462700	5.34520300	3.56831800
H	-5.72226200	3.94724500	2.55526100
H	-4.37453900	4.93262100	0.67493700
H	-3.69443900	7.34253600	0.76214600
H	-4.16435800	7.42534200	2.48159700
H	-2.79271000	6.40400400	1.97343400
H	-6.59793700	6.76634400	1.77257800
H	-6.05782300	6.68546600	0.07408200
H	-6.85719200	5.27697200	0.82654800
H	4.96474100	1.87768800	4.49235500
H	5.37563700	1.13884700	2.91767200
H	5.73133300	-0.18527500	5.67445600
H	7.01774300	0.64307200	4.77998400
H	5.53091100	-1.86446800	3.80934800
H	8.35740600	-1.58592500	5.00449600
H	6.94978800	-2.32627900	5.81506500
H	7.64308100	-3.08775900	4.35611600
H	8.01877500	-0.46293400	2.63885600
H	7.33692700	-2.01023500	2.07399100
H	6.40373700	-0.50472700	1.88135500
C	2.18094200	-3.71390300	-0.12733900
C	3.52103800	-3.29383900	-0.11039900
C	4.02782900	-2.53915700	0.98127000
C	3.20984300	-2.18161200	2.03605000
C	1.87233600	-2.64763400	2.12210300
C	1.39223200	-3.50456900	1.05763400
C	1.01622200	-2.28401600	3.22529100
C	-0.19606500	-2.96298400	3.35062400
C	-0.60206500	-3.93434600	2.39420600
C	0.13731100	-4.15659400	1.24786600
O	-1.08319500	-2.64843300	4.36609400
O	4.30126200	-3.61839300	-1.18464500
C	-1.10111400	-3.54682100	5.48969700
C	-2.17025100	-3.08441500	6.46785800
C	-1.98029200	-1.67041000	7.05514800
C	-0.68843400	-1.54435900	7.87556000
C	-3.20434200	-1.27794100	7.89538300
C	5.70718500	-3.34817300	-1.15008100
C	6.34069700	-3.88238700	-2.42661000
C	5.93220500	-3.17880900	-3.73704800
C	6.53312600	-3.91693100	-4.94205700
C	6.33201000	-1.69540500	-3.75016500
C	1.59127000	-4.34368600	-1.38750400
H	5.06400900	-2.19992800	0.98093200
H	3.61538300	-1.54279100	2.81957200
H	-1.54077900	-4.47432100	2.56517000
H	-0.23828800	-4.84778300	0.49061300
H	-0.09476000	-3.56242800	5.95376500
H	-1.31602700	-4.57634400	5.13769100
H	-2.21075800	-3.82235800	7.29347300
H	-3.15148600	-3.13730600	5.95665100
H	-1.91418600	-0.96275300	6.20574500
H	-0.69523000	-2.24441700	8.73248300
H	0.21324500	-1.75819800	7.27533400
H	-0.57805700	-0.52237600	8.27944000
H	-3.32428400	-1.95714300	8.76056600
H	-3.10633500	-0.25042600	8.28886900
H	-4.13588100	-1.32661000	7.30168400
H	6.15145800	-3.84471200	-0.26446600
H	5.87835600	-2.25889200	-1.04262800
H	6.10810300	-4.96134700	-2.49976200
H	7.43947700	-3.80801300	-2.30318000
H	4.83033500	-3.23825000	-3.82257900
H	7.63895100	-3.89464300	-4.90813200
H	6.21988400	-4.97624700	-4.96258600
H	6.21771100	-3.45229200	-5.89339600
H	7.42760800	-1.58057400	-3.64567800
H	6.03917200	-1.21816600	-4.70268000
H	5.85617400	-1.12428300	-2.93304100
C	-0.28843100	3.83625500	-0.25386700
C	0.95073500	4.30818000	-0.71327900
C	1.43878800	3.92664000	-1.99090300
C	0.73367900	3.03923100	-2.78243600
C	-0.55182500	2.57398700	-2.40454400
C	-1.09668600	3.06266300	-1.15472000
C	-1.30229500	1.66488000	-3.23897200

C	-2.63898200	1.43931100	-2.90786500
C	-3.21772200	2.01826200	-1.74559700
C	-2.46089900	2.76297800	-0.86369100
O	-3.41844500	0.58806500	-3.67059300
O	1.67010800	5.11713300	0.12303300
C	-4.49673300	1.19542600	-4.39862200
C	-5.32933700	0.13031900	-5.09923200
C	-6.14154800	-0.81615900	-4.18781800
C	-5.26144500	-1.87288000	-3.50030600
C	-7.27243200	-1.49111600	-4.97894600
C	2.87114900	5.73609300	-0.34952300
C	3.44306300	6.60822800	0.75861200
C	3.93423200	5.87018100	2.02084000
C	4.38838200	6.88370400	3.08120900
C	5.05292100	4.86402800	1.70986900
C	-0.74744000	4.13397300	1.17134100
H	2.40547400	4.28922700	-2.34038400
H	1.17946600	2.70635100	-3.71918400
H	-4.27414700	1.82467500	-1.53841200
H	-2.92116700	3.13477600	0.05369900
H	-4.07169400	1.90472500	-5.13801900
H	-5.13370300	1.78397700	-3.70575700
H	-4.67672000	-0.46296000	-5.76960300
H	-6.02999500	0.67650100	-5.75910000
H	-6.60767000	-0.19105700	-3.39648100
H	-4.79445100	-2.53515400	-4.25324400
H	-4.44615600	-1.40100200	-2.93160400
H	-5.85940700	-2.50933000	-2.82190000
H	-6.86106300	-2.09670300	-5.80838100
H	-7.86483500	-2.16609000	-4.33520400
H	-7.96224500	-0.74744200	-5.41657200
H	2.63799300	6.35130500	-1.24154700
H	3.59754400	4.96004900	-0.66116400
H	2.67675500	7.35338400	1.04364000
H	4.28648400	7.17950200	0.32219800
H	3.07594000	5.30975500	2.43831200
H	5.25070700	7.47520900	2.71927300
H	3.57903200	7.59206900	3.33396800
H	4.69729000	6.37653900	4.01281700
H	5.92262800	5.36926100	1.24848700
H	5.40886200	4.37483200	2.63457800
H	4.72188200	4.06792100	1.01939000
H	-1.74887500	4.58690600	1.14453000
H	-0.07105000	4.88703300	1.60321500
H	1.08468300	-5.28819100	-1.13652400
H	2.42075600	-4.59941600	-2.06400100
H	-1.46099000	0.47064700	-5.00984800
H	-0.16029100	1.66355500	-5.08092800
H	0.66301000	-1.20049500	5.03740800
H	2.37561500	-1.40836700	4.66348800
C	-2.40335100	-0.61516400	0.19972600
H	-2.63934000	-0.46440000	-0.86384700
H	-2.58096500	0.33127800	0.72567900
N	-3.48251300	-1.58502300	0.71251800
C	-4.84260000	-1.08670100	0.23773800
H	-4.77118700	-1.06752400	-0.85967300
H	-5.56712700	-1.87320500	0.50873700
C	-5.29649600	0.27015200	0.76327900
H	-5.57294400	0.20671700	1.83110800
H	-4.48626500	1.01353300	0.69604300
C	-0.95381500	-1.03359000	0.36282700
H	-0.71159000	-1.19671300	1.42417900
H	-0.74884300	-1.97118700	-0.17465600
C	-6.50100300	0.76684900	-0.05104900
H	-6.23918500	0.74836700	-1.12799300
H	-7.34786300	0.06071900	0.06048600
C	-0.05208900	0.07376900	-0.20476700
H	-0.25592400	0.17956600	-1.28111100
H	-0.32499600	1.03475400	0.25576800
C	-6.95149000	2.18117600	0.32831400
H	-7.23083000	2.20077000	1.39995400
H	-6.09026300	2.86915400	0.22902900
C	1.43613900	-0.18576400	0.01750800
H	1.62655200	-0.22771400	1.09941100
H	1.69011000	-1.18119100	-0.37766900
C	-8.11899500	2.68156700	-0.52528400
H	-8.42482400	3.70132000	-0.23402400
H	-7.84592400	2.70829000	-1.59680400
H	-9.00242300	2.02482500	-0.42341100
C	2.36066100	0.85322000	-0.61147900
H	3.41745900	0.61046200	-0.39452300
H	2.23961900	0.89253300	-1.70475900
H	2.15937900	1.86112500	-0.21729600
C	-3.27289200	-2.94857800	0.12598900
C	-3.46239800	-1.69626300	2.20562400
H	-4.08859700	-3.60451700	0.45967400
H	-3.27417500	-2.86105900	-0.96464300
H	-2.30705100	-3.34128000	0.45881100
H	-4.26805300	-2.37922000	2.51415600
H	-2.49500100	-2.08893800	2.53363000
H	-3.61336200	-0.70386100	2.63823200

1 2 1.5 6 1.5 23 1.0
2 3 1.5 12 1.0
3 4 2.0 24 1.0
4 5 1.5 25 1.0
5 6 1.0 7 1.5
6 10 1.5
7 8 1.5 121 1.0
8 9 1.5 11 1.0
9 10 2.0 26 1.0
10 27 1.0
11 13 1.0
12 18 1.0
13 14 1.0 28 1.0 29 1.0
14 15 1.0 30 1.0 31 1.0
15 16 1.0 17 1.0 32 1.0
16 33 1.0 34 1.0 35 1.0
17 36 1.0 37 1.0 38 1.0
18 19 1.0 39 1.0 40 1.0
19 20 1.0 41 1.0 42 1.0
20 21 1.0 22 1.0 43 1.0
21 44 1.0 45 1.0 46 1.0
22 47 1.0 48 1.0 49 1.0
23 154 1.0 201 1.0 202 1.0
24
25
26
27
28
29
30
31
32
33
34
35
36
37
38
39
40
41
42
43
44
45
46
47
48
49
50 51 1.5 55 1.5 72 1.0
51 52 1.5 61 1.0
52 53 2.0 73 1.0
53 54 1.5 74 1.0
54 55 1.0 56 1.5
55 59 1.5
56 57 1.5 170 1.0

57 58 1.5 60 1.0
58 59 2.0 75 1.0
59 76 1.0
60 62 1.0
61 67 1.0
62 63 1.0 77 1.0 78 1.0
63 64 1.0 79 1.0 80 1.0
64 65 1.0 66 1.0 81 1.0
65 82 1.0 83 1.0 84 1.0
66 85 1.0 86 1.0 87 1.0
67 68 1.0 88 1.0 89 1.0
68 69 1.0 90 1.0 91 1.0
69 70 1.0 71 1.0 92 1.0
70 93 1.0 94 1.0 95 1.0
71 96 1.0 97 1.0 98 1.0
72 105 1.0 203 1.0 204 1.0
73
74
75
76
77
78
79
80
81
82
83
84
85
86
87
88
89
90
91
92
93
94
95
96
97
98
99 100 1.5 104 1.5 121 1.0
100 101 1.5 110 1.0
101 102 2.0 122 1.0
102 103 1.5 123 1.0
103 104 1.0 105 1.5
104 108 1.5
105 106 1.5
106 107 1.5 109 1.0
107 108 2.0 124 1.0
108 125 1.0
109 111 1.0
110 116 1.0
111 112 1.0 126 1.0 127 1.0
112 113 1.0 128 1.0 129 1.0
113 114 1.0 115 1.0 130 1.0
114 131 1.0 132 1.0 133 1.0
115 134 1.0 135 1.0 136 1.0
116 117 1.0 137 1.0 138 1.0
117 118 1.0 139 1.0 140 1.0
118 119 1.0 120 1.0 141 1.0
119 142 1.0 143 1.0 144 1.0
120 145 1.0 146 1.0 147 1.0
121 199 1.0 200 1.0
122
123
124
125
126
127
128
129
130
131
132
133
134
135
136
137
138
139
140
141
142
143
144
145
146
147
148 149 1.5 153 1.5 170 1.0
149 150 1.5 159 1.0
150 151 2.0 171 1.0
151 152 1.5 172 1.0
152 153 1.0 154 1.5
153 157 1.5
154 155 1.5
155 156 1.5 158 1.0
156 157 2.0 173 1.0
157 174 1.0
158 160 1.0
159 165 1.0
160 161 1.0 175 1.0 176 1.0
161 162 1.0 177 1.0 178 1.0
162 163 1.0 164 1.0 179 1.0
163 180 1.0 181 1.0 182 1.0
164 183 1.0 184 1.0 185 1.0
165 166 1.0 186 1.0 187 1.0
166 167 1.0 188 1.0 189 1.0
167 168 1.0 169 1.0 190 1.0
168 191 1.0 192 1.0 193 1.0
169 194 1.0 195 1.0 196 1.0
170 197 1.0 198 1.0
171
172
173
174
175
176
177
178
179
180
181
182
183
184
185
186
187
188
189
190
191
192
193
194
195
196
197
198
199
200
201
202
203
204

205 206 1.0 207 1.0 208 1.0 215 1.0
206
207
208 209 1.0 238 1.0 239 1.0
209 210 1.0 211 1.0 212 1.0
210
211
212 213 1.0 214 1.0 218 1.0
213
214
215 216 1.0 217 1.0 221 1.0
216
217
218 219 1.0 220 1.0 224 1.0
219
220
221 222 1.0 223 1.0 227 1.0
222
223
224 225 1.0 226 1.0 230 1.0
225
226
227 228 1.0 229 1.0 234 1.0
228
229
230 231 1.0 232 1.0 233 1.0
231
232
233
234 235 1.0 236 1.0 237 1.0
235
236
237
238 240 1.0 241 1.0 242 1.0
239 243 1.0 244 1.0 245 1.0
240
241
242
243
244
245

0 imaginary frequency
Energy: -4393.2366 Hartree

Cartesian coordinates of **4^+** @ **PrS[4]*^{iPe}***

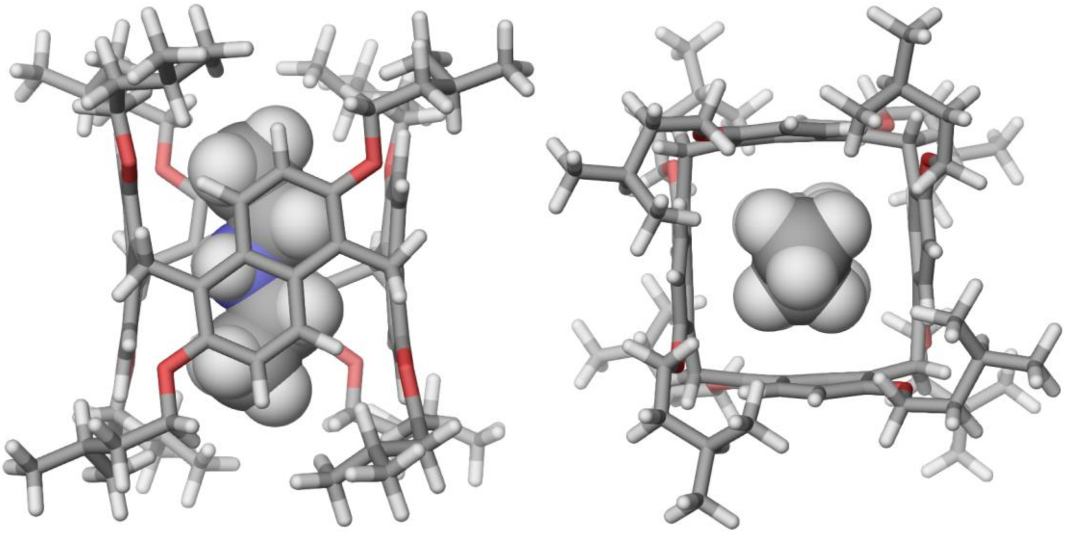

Figure S58: Side and top view of DFT-optimized structure (B97D3/SVP/SVPFIT) of the **4^+** @ **PrS[4]*^{iPe}*** complex.

C	1.11658900	1.47599400	3.48709700
C	2.50586000	1.53196200	3.28949300
C	3.04858500	2.27512600	2.20548600
C	2.23244300	2.97379700	1.33497100
C	0.82475300	3.04246900	1.53568400
C	0.27927800	2.30196400	2.65763800
C	-0.01521300	3.87072200	0.70665400
C	-1.34854600	4.06494000	1.09886500
C	-1.88115300	3.35858700	2.21322100
C	-1.10442600	2.47774900	2.94164800
O	-2.10909800	4.93470800	0.36889700
O	3.30085400	0.84263900	4.16051600
C	-3.41079100	5.30712700	0.83814100
C	-4.00487600	6.33253100	-0.11589800
C	-4.29686800	5.84408400	-1.54970700
C	-4.82453400	7.00599200	-2.40393300
C	-5.27197200	4.65696000	-1.57671900
C	4.72389400	0.99230800	4.09781700
C	5.35318300	0.17045200	5.21317100
C	5.18700800	-1.35956300	5.10879600
C	5.76233200	-2.03926200	6.35979800
C	5.82605000	-1.93068300	3.83365700
C	0.52314500	0.56160100	4.55818700
H	4.12599400	2.29404200	2.03972200
H	2.69474500	3.51440000	0.50912400
H	-2.92374400	3.49642800	2.50075600
H	-1.56428100	1.94223300	3.77203400
H	-3.32167000	5.73429700	1.85677000
H	-4.05685300	4.40997600	0.90835500
H	-3.32583000	7.20466100	-0.15638000
H	-4.94753100	6.69269100	0.34208800
H	-3.33921000	5.51104000	-1.99382200
H	-5.78833400	7.38023100	-2.00992700
H	-4.11510200	7.85274800	-2.41374100
H	-4.98941000	6.69076800	-3.44970800
H	-6.23588900	4.92498000	-1.10408700
H	-5.48878200	4.35116800	-2.61618700
H	-4.87314100	3.77315800	-1.04710700
H	4.98299600	2.06350400	4.21493300
H	5.09334300	0.66659700	3.10516400

H	4.93729700	0.52057900	6.17637800
H	6.43326100	0.41757500	5.23164700
H	4.10260200	-1.57916300	5.07697100
H	6.84924500	-1.85141200	6.44710400
H	5.28184600	-1.66135700	7.27998500
H	5.61288700	-3.13316700	6.32487700
H	6.91218400	-1.72103300	3.80722800
H	5.69957300	-3.02734500	3.78847100
H	5.38165100	-1.50732500	2.91491100
C	-0.00526800	-1.49315500	-3.49430500
C	1.32722000	-1.92129900	-3.59754000
C	1.86430100	-2.83775300	-2.65100000
C	1.09277200	-3.33717500	-1.61904800
C	-0.28888000	-3.01525100	-1.50493100
C	-0.83921400	-2.09806200	-2.48554500
C	-1.11929000	-3.61300500	-0.49300800
C	-2.50644700	-3.41045000	-0.56959100
C	-3.05572300	-2.53656600	-1.54674400
C	-2.24512300	-1.87549700	-2.45113900
O	-3.29257600	-4.07884200	0.32247500
O	2.08280600	-1.41791800	-4.61903700
C	-4.68589600	-3.77258700	0.42778800
C	-5.26438300	-4.56316800	1.59330200
C	-5.08113000	-6.09284800	1.51865800
C	-5.67518800	-6.69247900	0.23589500
C	-5.67742700	-6.75339100	2.76988000
C	3.38377500	-1.96112300	-4.87532500
C	3.97335900	-1.27478800	-6.09843900
C	4.26392500	0.23410200	-5.96275100
C	4.78749200	0.79071300	-7.29475100
C	5.24153200	0.54158000	-4.81800400
C	-0.55630400	-0.43690300	-4.45190200
H	2.90617800	-3.15077800	-2.72278900
H	1.55608500	-4.01748300	-0.90472500
H	-4.13310700	-2.37422900	-1.59223000
H	-2.71085300	-1.20591000	-3.17397800
H	-5.19652800	-4.02853600	-0.52112000
H	-4.81489600	-2.68334200	0.59584700
H	-6.34517600	-4.32331000	1.64606300
H	-4.81674500	-4.18973900	2.53462300
H	-3.99160400	-6.29186500	1.51286800
H	-5.57193600	-7.79197500	0.23406600
H	-6.75402300	-6.45947700	0.15029800
H	-5.17060000	-6.31617100	-0.67154800
H	-6.77099000	-6.59095400	2.81969300
H	-5.50149400	-7.84368700	2.76744700
H	-5.23449000	-6.34157500	3.69509200
H	3.29449100	-3.05167100	-5.05079200
H	4.03278500	-1.81855300	-3.98891900
H	3.29195100	-1.44175300	-6.95351800
H	4.91590400	-1.80364300	-6.34338500
H	3.30621600	0.74304200	-5.74137300
H	5.75110000	0.32054800	-7.56790500
H	4.07617600	0.59970100	-8.11811800
H	4.95135700	1.88151800	-7.23557800
H	6.20570000	0.02021800	-4.96936400
H	5.45718300	1.62410900	-4.76653900
H	4.84545600	0.23477900	-3.83334400
C	0.86305600	3.56607000	-1.66562800
C	2.19092100	3.35309300	-2.06667500
C	2.50363600	2.33064700	-3.00485100
C	1.52182500	1.49686600	-3.50562800
C	0.14858700	1.70768300	-3.19959800
C	-0.17396500	2.81961600	-2.32839000
C	-0.88826500	0.88319700	-3.76258100
C	-2.21677300	1.32111300	-3.64883700

C	-2.52999100	2.47563600	-2.87941600
C	-1.54762500	3.16395500	-2.19302500
O	-3.18349200	0.58360900	-4.27149600
O	3.15786600	4.13582100	-1.50191500
C	-4.53395800	1.06142200	-4.30636100
C	-5.38409400	0.06928900	-5.08591000
C	-5.54774400	-1.32877600	-4.45490000
C	-6.33228800	-2.24688000	-5.40333100
C	-6.21634500	-1.27066000	-3.07276300
C	4.50690500	4.05796800	-1.97851000
C	5.35767100	5.05526500	-1.20622500
C	5.52822400	4.78032000	0.30203200
C	6.31821500	5.92115700	0.95961500
C	6.19554800	3.42376800	0.57636500
C	0.53124300	4.55081400	-0.54823000
H	3.53736400	2.16282600	-3.30825000
H	1.81452900	0.68352900	-4.16957100
H	-3.56421300	2.80793200	-2.78591200
H	-1.84058900	4.00252200	-1.56150400
H	-4.55532200	2.05610000	-4.79434800
H	-4.91486500	1.18810000	-3.27371500
H	-4.95405200	-0.03298000	-6.09975000
H	-6.38434400	0.52800300	-5.21618100
H	-4.53676200	-1.76173800	-4.32936000
H	-7.35683300	-1.86278200	-5.56772600
H	-5.84010300	-2.32163200	-6.38951000
H	-6.41909400	-3.26773900	-4.99036900
H	-7.22086400	-0.81133200	-3.13789200
H	-6.34485200	-2.28589300	-2.65597800
H	-5.62743400	-0.68484400	-2.34410000
H	4.52426600	4.29260900	-3.06138700
H	4.89084800	3.02610800	-1.85449100
H	4.92510100	6.06305200	-1.34944200
H	6.35605600	5.07325600	-1.68666500
H	4.51951600	4.76515700	0.75765500
H	7.34118400	5.98518400	0.54287400
H	5.82766200	6.89721900	0.79521800
H	6.40925800	5.76781000	2.04979200
H	7.19597200	3.37227000	0.10650500
H	6.33312400	3.26625200	1.66135400
H	5.60078800	2.57618300	0.19080000
C	0.27136700	-3.58878900	1.63763700
C	1.66766200	-3.70489300	1.71697300
C	2.42050100	-2.79708300	2.51253600
C	1.80033300	-1.77628700	3.20906700
C	0.38343000	-1.64936400	3.23030700
C	-0.38127600	-2.62484200	2.48392800
C	-0.27012300	-0.60544300	3.97317400
C	-1.66406200	-0.66863500	4.12639500
C	-2.41571000	-1.67815400	3.46100000
C	-1.79564200	-2.60015600	2.63712000
O	-2.25706500	0.27661000	4.91765000
O	2.26636700	-4.69891600	0.99259100
C	-3.64561800	0.16604900	5.24584000
C	-4.01920300	1.30216500	6.18717900
C	-3.93475100	2.72769400	5.60391100
C	-4.22974600	3.76359600	6.69843000
C	-4.86988100	2.92271700	4.40046800
C	3.66240700	-4.96327200	1.16804300
C	4.04919700	-6.15460300	0.30377900
C	3.95588100	-5.94941100	-1.22217900
C	4.25811000	-7.26677700	-1.95106800
C	4.88019900	-4.82637900	-1.71752300
C	-0.52192900	-4.43847400	0.64694800
H	3.50618900	-2.88496300	2.56751800
H	2.42041200	-1.07611700	3.76797500

H	-3.50028500	-1.72955700	3.58551600
H	-2.40888700	-3.33812300	2.12088700
H	-3.82893300	-0.81218500	5.73313000
H	-4.25528200	0.19846200	4.32072300
H	-3.37452000	1.23361000	7.08327300
H	-5.05484800	1.11293100	6.53297400
H	-2.89501800	2.88995400	5.26056800
H	-5.26091600	3.64954600	7.08306600
H	-3.53905500	3.65320700	7.55349300
H	-4.12796200	4.79357000	6.31222100
H	-5.92369600	2.74051000	4.68430400
H	-4.80614600	3.95718800	4.01722300
H	-4.62633900	2.24292400	3.56417300
H	3.86050500	-5.18271300	2.23614500
H	4.25342300	-4.06555200	0.89781500
H	3.41766200	-7.01353300	0.59821100
H	5.08991400	-6.42716900	0.56941500
H	2.91272800	-5.66625000	-1.46087400
H	5.29251200	-7.60273200	-1.74755200
H	3.57446200	-8.07179400	-1.62711800
H	4.15181500	-7.15239200	-3.04467300
H	5.93704700	-5.04891100	-1.47705800
H	4.80855500	-4.71365500	-2.81444200
H	4.63347200	-3.84857700	-1.26602600
H	-1.33983800	-4.96211100	1.16343600
H	0.14178900	-5.21750000	0.24361400
H	-0.21165100	5.28462800	-0.89540700
H	1.44083100	5.11887200	-0.30188700
H	-0.13953600	1.13656300	5.22205100
H	1.34446300	0.18530600	5.18606700
H	-1.46683900	-0.81303600	-4.94206800
H	0.18377200	-0.27364400	-5.24970800
C	-1.25414000	-0.06715600	0.47050500
H	-1.33723300	-1.15505900	0.35142200
H	-1.03882000	0.14497400	1.52679400
N	-0.01736900	0.33268400	-0.29053100
C	1.22477800	-0.30724800	0.27183500
H	1.31370600	0.06992500	1.29853700
H	1.01161600	-1.38392400	0.32093400
C	2.47208100	-0.02464500	-0.53868800
H	2.39097300	-0.41685600	-1.56345400
H	2.69401700	1.05160200	-0.58195500
C	-2.50605000	0.64576500	0.00451300
H	-2.43048000	1.73483800	0.14107800
H	-2.72760900	0.42988300	-1.05075300
H	-0.12812200	0.07768000	-1.28823200
H	0.08925300	1.36284500	-0.27873600
H	-3.34940100	0.27970800	0.61408100
H	3.31764100	-0.52969400	-0.04192200

1 2 1.5 6 1.5 23 1.0
2 3 1.5 12 1.0
3 4 2.0 24 1.0
4 5 1.5 25 1.0
5 6 1.0 7 1.5
6 10 1.5
7 8 1.5 121 1.0
8 9 1.5 11 1.0
9 10 2.0 26 1.0
10 27 1.0
11 13 1.0
12 18 1.0
13 14 1.0 28 1.0 29 1.0
14 15 1.0 30 1.0 31 1.0
15 16 1.0 17 1.0 32 1.0
16 33 1.0 34 1.0 35 1.0
17 36 1.0 37 1.0 38 1.0
18 19 1.0 39 1.0 40 1.0
19 20 1.0 41 1.0 42 1.0
20 21 1.0 22 1.0 43 1.0
21 44 1.0 45 1.0 46 1.0
22 47 1.0 48 1.0 49 1.0
23 154 1.0 201 1.0 202 1.0
24
25
26
27
28
29
30
31
32
33
34
35
36
37
38
39
40
41
42
43
44
45
46
47
48
49
50 51 1.5 55 1.5 72 1.0
51 52 1.5 61 1.0
52 53 2.0 73 1.0
53 54 1.5 74 1.0
54 55 1.0 56 1.5
55 59 1.5
56 57 1.5 170 1.0
57 58 1.5 60 1.0
58 59 2.0 75 1.0
59 76 1.0
60 62 1.0
61 67 1.0
62 63 1.0 77 1.0 78 1.0
63 64 1.0 79 1.0 80 1.0
64 65 1.0 66 1.0 81 1.0
65 82 1.0 83 1.0 84 1.0
66 85 1.0 86 1.0 87 1.0
67 68 1.0 88 1.0 89 1.0
68 69 1.0 90 1.0 91 1.0
69 70 1.0 71 1.0 92 1.0
70 93 1.0 94 1.0 95 1.0
71 96 1.0 97 1.0 98 1.0
72 105 1.0 203 1.0 204 1.0
73
74
75
76
77
78
79
80
81
82

83
84
85
86
87
88
89
90
91
92
93
94
95
96
97
98
99 100 1.5 104 1.5 121 1.0
100 101 1.5 110 1.0
101 102 2.0 122 1.0
102 103 1.5 123 1.0
103 104 1.0 105 1.5
104 108 1.5
105 106 1.5
106 107 1.5 109 1.0
107 108 2.0 124 1.0
108 125 1.0
109 111 1.0
110 116 1.0
111 112 1.0 126 1.0 127 1.0
112 113 1.0 128 1.0 129 1.0
113 114 1.0 115 1.0 130 1.0
114 131 1.0 132 1.0 133 1.0
115 134 1.0 135 1.0 136 1.0
116 117 1.0 137 1.0 138 1.0
117 118 1.0 139 1.0 140 1.0
118 119 1.0 120 1.0 141 1.0
119 142 1.0 143 1.0 144 1.0
120 145 1.0 146 1.0 147 1.0
121 199 1.0 200 1.0
122
123
124
125
126
127
128
129
130
131
132
133
134
135
136
137
138
139
140
141
142
143
144
145
146
147
148 149 1.5 153 1.5 170 1.0
149 150 1.5 159 1.0
150 151 2.0 171 1.0
151 152 1.5 172 1.0
152 153 1.5 154 1.5
153 157 1.5
154 155 1.5
155 156 1.5 158 1.0
156 157 2.0 173 1.0
157 174 1.0
158 160 1.0
159 165 1.0
160 161 1.0 175 1.0 176 1.0
161 162 1.0 177 1.0 178 1.0
162 163 1.0 164 1.0 179 1.0
163 180 1.0 181 1.0 182 1.0
164 183 1.0 184 1.0 185 1.0
165 166 1.0 186 1.0 187 1.0
166 167 1.0 188 1.0 189 1.0
167 168 1.0 169 1.0 190 1.0
168 191 1.0 192 1.0 193 1.0
169 194 1.0 195 1.0 196 1.0
170 197 1.0 198 1.0
171
172
173
174
175
176
177
178
179
180
181
182
183
184
185
186
187
188
189
190
191
192
193
194
195
196
197
198
199
200
201
202
203
204
205 206 1.0 207 1.0 208 1.0 215 1.0
206
207
208 209 1.0 218 1.0 219 1.0
209 210 1.0 211 1.0 212 1.0
210
211
212 213 1.0 214 1.0 221 1.0
213
214

215 216 1.0 217 1.0 220 1.0
216
217
218
219
220
221

---

0 imaginary frequency
Energy: -4079.1829 Hartree

Cartesian coordinates of (*S*)-**7**$^{+}$ @ *pR*-**PrS[4]**^{iPe}

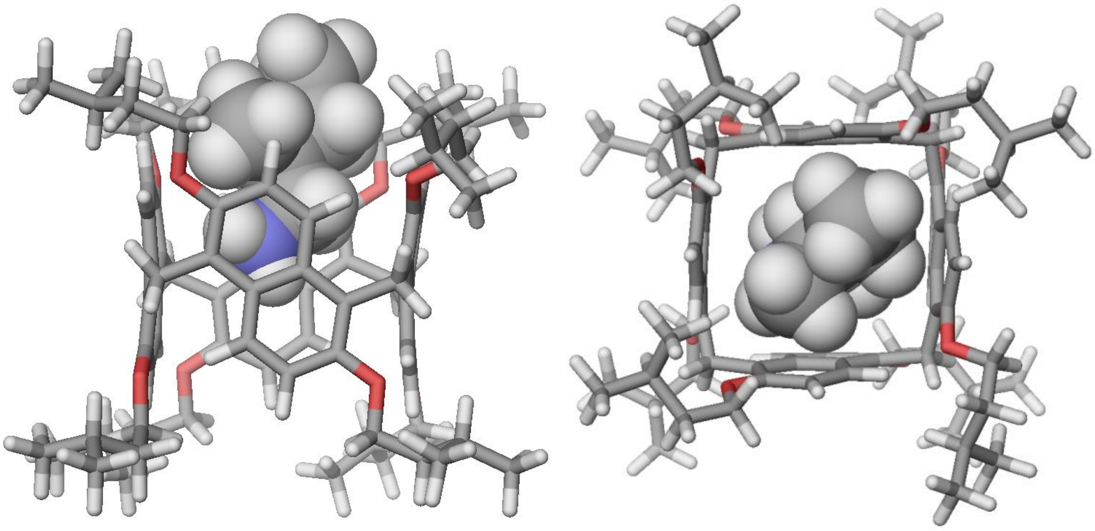

Figure S59: Side and top view of DFT-optimized structure (B97D3/SVP/SVPFIT) of the (*S*)-**7**$^{+}$ @ *pR*-**PrS[4]**^{iPe} complex.

C	-2.28482200	0.63505900	-3.03724200
C	-1.32260800	0.61058300	-4.05861200
C	-0.21053100	1.49944600	-4.02122800
C	-0.03649800	2.38504600	-2.97349900
C	-1.01709400	2.51570700	-1.94565100
C	-2.17603100	1.64762500	-2.01773600
C	-0.91122800	3.52400600	-0.92566600
C	-2.02548500	3.76104200	-0.10525500
C	-3.17341100	2.92654400	-0.18597000
C	-3.23140800	1.88412200	-1.09214700
O	-1.95898700	4.82085500	0.74992400
O	-1.49414600	-0.30114400	-5.06161600
C	-2.98452800	5.04141600	1.72276800
C	-2.55703800	6.20175100	2.61185500
C	-2.23671300	7.52181500	1.88109800
C	-3.41242000	8.02175200	1.02933600
C	-1.79107500	8.58613700	2.89409800
C	-0.70191700	-0.23090500	-6.25346000
C	-1.24814300	-1.24053500	-7.25391500
C	-1.23636300	-2.71669500	-6.80647500
C	-1.89777900	-3.59732800	-7.87653100
C	0.17659000	-3.22065600	-6.47769400
C	-3.42771300	-0.37919600	-3.02733800
H	0.53391100	1.47685200	-4.81979200
H	0.85637800	3.01312700	-2.96655300
H	-4.03436400	3.11117200	0.46161900
H	-4.13679500	1.28091600	-1.13487800
H	-3.94114600	5.26086400	1.20961200
H	-3.12663400	4.12290400	2.32804600
H	-3.37412800	6.37259900	3.34089700
H	-1.67341900	5.89113500	3.20217000
H	-1.38654500	7.32072600	1.20019200
H	-4.31771600	8.17032700	1.64881900
H	-3.66565200	7.31828400	0.21655800
H	-3.16987300	8.99084500	0.55845300
H	-2.61056200	8.83121900	3.59628500
H	-1.49569400	9.52133600	2.38619100
H	-0.92889500	8.23942700	3.49264300
H	-0.75825400	0.79290500	-6.67246100
H	0.35871500	-0.43822100	-6.01184800
H	-2.28339600	-0.94826700	-7.51110700
H	-0.65341300	-1.13466200	-8.18286400
H	-1.84831300	-2.79048600	-5.88656200
H	-1.32759700	-3.56255700	-8.82405500
H	-2.92823700	-3.26308300	-8.09300600
H	-1.94518100	-4.65225000	-7.55244400
H	0.83794100	-3.15105300	-7.36200600
H	0.15192900	-4.28002500	-6.16463300
H	0.64613600	-2.64350100	-5.66300300
C	2.90481700	-0.87893600	2.62774400
C	3.84742500	-1.43802700	1.75329800
C	3.49737200	-2.53905400	0.92504900
C	2.22640800	-3.08762400	0.96185200
C	1.25199100	-2.62995400	1.89736900
C	1.62639700	-1.52417900	2.75731200
C	-0.01269200	-3.29883200	2.06317500
C	-0.80264300	-2.96607900	3.17331600
C	-0.43889100	-1.88272700	4.01783200
C	0.71779100	-1.16219600	3.79204900
O	-1.92616500	-3.71211800	3.39969400
O	5.08987700	-0.86906600	1.71872200
C	-2.67039400	-3.53557400	4.61077800
C	-3.80727200	-4.54718800	4.63507700
C	-4.89207100	-4.37631900	3.55172800
C	-5.88641500	-5.54502700	3.61022600
C	-5.62385200	-3.03038600	3.66639300
C	6.18356100	-1.55343700	1.09847900
C	7.46866500	-0.80726000	1.43160100
C	7.51913300	0.67625700	1.01192200
C	8.85196500	1.30122900	1.44875300
C	7.28451500	0.87264800	-0.49260800
C	3.24300100	0.37865600	3.42457700
H	4.23310300	-2.95791900	0.23689300
H	1.99820600	-3.92225200	0.29728000

H	-1.07199900	-1.60855800	4.86270300
H	0.95694600	-0.34264800	4.46771700
H	-1.99760400	-3.69018200	5.47765600
H	-3.05599200	-2.49853000	4.66837000
H	-3.36862100	-5.55974500	4.55917300
H	-4.27985100	-4.48602800	5.63547300
H	-4.39066300	-4.41167500	2.56535700
H	-6.64861300	-5.46175900	2.81497700
H	-6.41583400	-5.56510600	4.58161700
H	-5.37401200	-6.51610600	3.48773400
H	-6.13723500	-2.94155200	4.64249900
H	-6.39229400	-2.93247900	2.87846700
H	-4.93802000	-2.16939000	3.57130600
H	6.23174100	-2.59314500	1.47851100
H	6.02423400	-1.60497000	0.00401000
H	7.64233900	-0.87960000	2.52156800
H	8.29985600	-1.35519000	0.94468200
H	6.70765800	1.20097900	1.55275600
H	9.70282800	0.81878100	0.93140200
H	9.01338800	1.18836300	2.53587500
H	8.88385900	2.37969900	1.21213100
H	8.04034500	0.32629600	-1.08848600
H	7.35598000	1.94107900	-0.76404800
H	6.28803500	0.51956000	-0.80531700
C	1.51953200	3.53656800	-0.15068900
C	2.65821700	3.25868900	-0.90951500
C	3.71270200	2.46159800	-0.38616300
C	3.60648100	1.86694100	0.85492500
C	2.47952100	2.12643300	1.69364800
C	1.46788400	3.03639800	1.20209300
C	2.37399900	1.55882500	3.00847700
C	1.41148300	2.07863900	3.88783900
C	0.46420200	3.03544300	3.43360800
C	0.46644000	3.46111200	2.11735100
O	1.41611900	1.60712500	5.17036700
O	2.74541400	3.69623300	-2.21429800
C	0.53486900	2.16900100	6.14783200
C	0.79201900	1.48102800	7.48128000
C	0.43576800	-0.01844600	7.54788000
C	0.92391800	-0.62117200	8.87308400
C	-1.06812600	-0.26762600	7.35658100
C	3.70382600	4.73525100	-2.48070300
C	3.58733800	5.13975100	-3.94234200
C	2.21658300	5.69775100	-4.37855200
C	1.82797000	6.96940900	-3.61040300
C	2.20592700	5.94617300	-5.89394500
C	0.36542600	4.33872300	-0.74491000
H	4.59771000	2.28987000	-1.00556700
H	4.40644200	1.21107200	1.20103400
H	-0.28956500	3.43495800	4.11689200
H	-0.28919700	4.17676300	1.79771000
H	0.72766000	3.25705300	6.23218700
H	-0.51713500	2.03853500	5.82418300
H	1.85998000	1.61713500	7.73484400
H	0.21554900	2.02777400	8.25358900
H	0.97579500	-0.52851200	6.72698000
H	0.42532300	-0.13767300	9.73448400
H	2.01346000	-0.48821100	8.99859700
H	0.70679000	-1.70312300	8.92452300
H	-1.65399600	0.21907300	8.15916000
H	-1.29512800	-1.34845300	7.38907700
H	-1.43909300	0.12125400	6.39123000
H	3.50752400	5.58756200	-1.80016500
H	4.72766900	4.36839600	-2.26341600
H	4.37224200	5.89580400	-4.14294100
H	3.83680100	4.25912600	-4.56537200
H	1.45670700	4.92177700	-4.16047200
H	2.57179500	7.77225800	-3.77382600
H	1.75410300	6.79711700	-2.52214800
H	0.84758900	7.34869500	-3.94950900
H	2.95058600	6.71536000	-6.17352100
H	1.21640100	6.30006500	-6.23431100
H	2.44921200	5.02601100	-6.45628800
C	-0.85666600	-3.78350100	-0.28578600

C	-0.10155500	-4.10894100	-1.42656200
C	-0.38911900	-3.49855800	-2.67848700
C	-1.41314500	-2.57409600	-2.80320100
C	-2.25865400	-2.25215000	-1.70215300
C	-1.99597200	-2.91765000	-0.44215000
C	-3.33992200	-1.31444500	-1.82823400
C	-4.27019100	-1.22761600	-0.78348500
C	-4.05315600	-1.92914900	0.43395900
C	-2.92718200	-2.70899800	0.61488800
O	-5.35667900	-0.42766200	-0.97611000
O	0.90109200	-5.02031900	-1.27414200
C	-6.25788500	-0.15655500	0.10382100
C	-7.28595900	0.85813000	-0.37616900
C	-8.10308500	0.44597900	-1.61782000
C	-8.85692800	-0.87592600	-1.41183900
C	-9.06320100	1.57750100	-2.01201800
C	1.59094200	-5.54067200	-2.41626600
C	2.43357500	-6.72795300	-1.96928900
C	3.48948400	-6.44348800	-0.88231700
C	4.19297000	-7.74577000	-0.47439000
C	4.50589800	-5.38223700	-1.32352600
C	-0.48407100	-4.36128900	1.07628100
H	-4.77249800	-1.84736900	1.25007000
H	-2.78537000	-3.20329800	1.57474500
H	-6.74288400	-1.09826300	0.42652900
H	-5.69041600	0.24461200	0.96822000
H	-7.97411600	1.05144700	0.47085900
H	-6.76932500	1.81545100	-0.58255000
H	-7.38494400	0.30100100	-2.44850900
H	-9.54021500	-0.81301000	-0.54317000
H	-8.17079900	-1.72539700	-1.24630700
H	-9.46878300	-1.11935400	-2.29840200
H	-9.80955200	1.75806400	-1.21511500
H	-9.61476300	1.32880400	-2.93597200
H	-8.52211900	2.52553300	-2.18582600
H	0.85536600	-5.86570500	-3.17770900
H	2.21298500	-4.74217800	-2.86960200
H	1.74930400	-7.51961100	-1.61102800
H	2.93422500	-7.13179700	-2.87163500
H	2.95567900	-6.05500000	0.00664500
H	4.74534800	-8.18106300	-1.32856700
H	3.46829900	-8.50206700	-0.12320200
H	4.91871700	-7.56911800	0.33930800
H	5.05494900	-5.70613800	-2.22787500
H	5.25102900	-5.19443000	-0.53009400
H	4.01891000	-4.41959100	-1.55477400
H	-1.35236400	-4.87996700	1.50996500
H	0.29606700	-5.12293100	0.93171400
H	0.14133800	5.20200300	-0.10048300
H	0.69179400	4.74319500	-1.71377700
H	-4.39551300	0.14355600	-2.99948400
H	-3.40488900	-0.94475900	-3.97029600
H	3.10119300	0.19651000	4.50048800
H	4.30968400	0.60700900	3.28352900
H	0.20677200	-3.74537800	-3.55867000
H	-1.58131900	-2.10890000	-3.77569900
C	1.64941800	-0.14851000	-1.07659900
H	1.62072100	0.89255500	-1.42847400
H	2.10189300	-0.16302700	-0.07473100
N	0.21684700	-0.56845700	-0.88929200
C	2.42401300	-1.05581300	-2.03714700
H	2.34182900	-2.08961900	-1.64920800
C	1.89531200	-1.01962600	-3.47513400
C	3.90852200	-0.64010100	-1.98379400
H	3.98886300	0.39632700	-2.36141900
H	4.22295200	-0.59799300	-0.92845200
C	4.85664300	-1.54905600	-2.76703300
H	4.77707100	-2.59814600	-2.43186000
H	4.65487900	-1.52794900	-3.85184200
H	-0.32594500	-0.56933100	-1.77213400
H	0.13716200	-1.52442200	-0.49203300
H	5.90419000	-1.23195600	-2.62522400
H	-0.27821900	0.07305500	-0.25498000
H	2.35469500	-1.82071700	-4.07671100

H 0.80677500 -1.16292700 -3.53336700
H 2.13706900 -0.05425700 -3.94922400

1 2 1.5 6 1.5 23 1.0
2 3 1.5 12 1.0
3 4 2.0 24 1.0
4 5 1.5 25 1.0
5 6 1.0 7 1.5
6 10 1.5
7 8 1.5 121 1.0
8 9 1.5 11 1.0
9 10 2.0 26 1.0
10 27 1.0
11 13 1.0
12 18 1.0
13 14 1.0 28 1.0 29 1.0
14 15 1.0 30 1.0 31 1.0
15 16 1.0 17 1.0 32 1.0
16 33 1.0 34 1.0 35 1.0
17 36 1.0 37 1.0 38 1.0
18 19 1.0 39 1.0 40 1.0
19 20 1.0 41 1.0 42 1.0
20 21 1.0 22 1.0 43 1.0
21 44 1.0 45 1.0 46 1.0
22 47 1.0 48 1.0 49 1.0
23 154 1.0 199 1.0 200 1.0
24
25
26
27
28
29
30
31
32
33
34
35
36
37
38
39
40
41
42
43
44
45
46
47
48
49
50 51 1.5 55 1.5 72 1.0
51 52 1.5 61 1.0
52 53 2.0 73 1.0
53 54 1.5 74 1.0
54 55 1.0 56 1.5
55 59 1.5
56 57 1.5 170 1.0
57 58 1.5 60 1.0
58 59 2.0 75 1.0
59 76 1.0
60 62 1.0
61 67 1.0
62 63 1.0 77 1.0 78 1.0
63 64 1.0 79 1.0 80 1.0
64 65 1.0 66 1.0 81 1.0
65 82 1.0 83 1.0 84 1.0
66 85 1.0 86 1.0 87 1.0
67 68 1.0 88 1.0 89 1.0
68 69 1.0 90 1.0 91 1.0
69 70 1.0 71 1.0 92 1.0
70 93 1.0 94 1.0 95 1.0
71 96 1.0 97 1.0 98 1.0
72 105 1.0 201 1.0 202 1.0
73
74
75
76
77
78
79
80
81
82
83
84
85
86
87
88
89
90
91
92
93
94
95
96
97
98
99 100 1.5 104 1.5 121 1.0
100 101 1.5 110 1.0
101 102 2.0 122 1.0
102 103 1.5 123 1.0
103 104 1.5 105 1.5
104 108 1.5
105 106 1.5
106 107 1.5 109 1.0
107 108 2.0 124 1.0
108 125 1.0
109 111 1.0
110 116 1.0
111 112 1.0 126 1.0 127 1.0
112 113 1.0 128 1.0 129 1.0
113 114 1.0 115 1.0 130 1.0
114 131 1.0 132 1.0 133 1.0
115 134 1.0 135 1.0 136 1.0
116 117 1.0 137 1.0 138 1.0
117 118 1.0 139 1.0 140 1.0
118 119 1.0 120 1.0 141 1.0
119 142 1.0 143 1.0 144 1.0
120 145 1.0 146 1.0 147 1.0
121 197 1.0 198 1.0
122
123
124
125
126
127
128
129
130
131
132
133
134
135
136
137
138
139
140
141
142
143
144
145

146
147
148 149 1.5 153 1.5 170 1.0
149 150 1.5 159 1.0
150 151 2.0 203 1.0
151 152 1.5 204 1.0
152 153 1.0 154 1.5
153 157 1.5
154 155 1.5
155 156 1.5 158 1.0
156 157 2.0 171 1.0
157 172 1.0
158 160 1.0
159 165 1.0
160 161 1.0 173 1.0 174 1.0
161 162 1.0 175 1.0 176 1.0
162 163 1.0 164 1.0 177 1.0
163 178 1.0 179 1.0 180 1.0
164 181 1.0 182 1.0 183 1.0
165 166 1.0 184 1.0 185 1.0
166 167 1.0 186 1.0 187 1.0
167 168 1.0 169 1.0 188 1.0
168 189 1.0 190 1.0 191 1.0
169 192 1.0 193 1.0 194 1.0
170 195 1.0 196 1.0
171
172
173
174
175
176
177
178
179
180
181
182
183
184
185
186
187
188
189
190
191
192
193
194
195
196
197
198
199
200
201
202
203
204
205 206 1.0 207 1.0 208 1.0 209 1.0
206
207
208 218 1.0 219 1.0 221 1.0
209 210 1.0 211 1.0 212 1.0
210
211 222 1.0 223 1.0 224 1.0
212 213 1.0 214 1.0 215 1.0
213
214
215 216 1.0 217 1.0 220 1.0
216
217
218
219
220
221
222
223
224

---

0 imaginary frequency
Energy: -4118.4266 Hartree

Cartesian coordinates of (*S*)-**7**$^+$ @ *pS*-**PrS[4]**^{iPe}

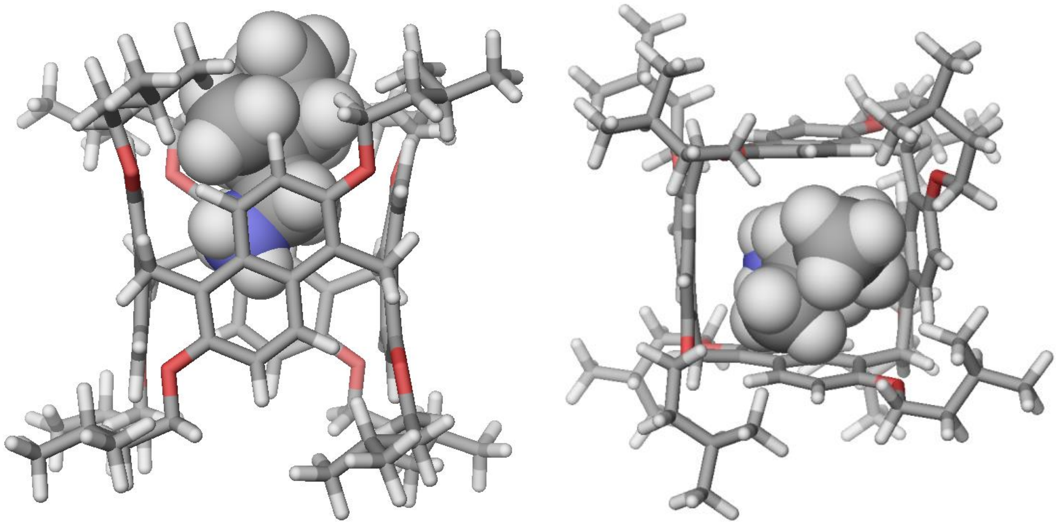

Figure S60: Side and top view of DFT-optimized structure (B97D3/SVP/SVPFIT) of the (*S*)-**7**$^+$ @ *pS*-**PrS[4]**^{iPe} complex.

C	-1.49750500	-1.08593200	-3.44653200
C	-0.36761400	-0.87897500	-4.25518000
C	0.83957100	-1.59330900	-4.00336200
C	0.93155400	-2.48876400	-2.95299400
C	-0.20206700	-2.80123200	-2.14460200
C	-1.44600500	-2.12552000	-2.45070800
C	-0.13281500	-3.76923600	-1.08415600
C	-1.33266200	-4.20698200	-0.50234400

C	-2.56738100	-3.59174700	-0.84695700
C	-2.61368600	-2.55460300	-1.75982800
O	-1.25406200	-5.22538100	0.39891100
O	-0.47258700	0.04483500	-5.25375300
C	-2.39774800	-5.60654500	1.17183200
C	-1.95794000	-6.64599700	2.19395100
C	-1.28241800	-7.90725700	1.61773600
C	-2.17268800	-8.63818000	0.60234100
C	-0.85830600	-8.84187600	2.75977700
C	0.52234700	0.12495900	-6.28322400
C	0.02770600	1.08821700	-7.35348700
C	-0.23907500	2.53635400	-6.89361600
C	-0.82458600	3.35708300	-8.05188000
C	1.01538700	3.21222500	-6.32088700
C	-2.74604400	-0.22612000	-3.62549600
H	1.71863300	-1.41867400	-4.62833800
H	1.88706600	-2.98087600	-2.76668500
H	-3.49858700	-3.93206800	-0.38676300
H	-3.57641900	-2.09873700	-1.98640700
H	-3.18026800	-6.01003800	0.50024300
H	-2.81415400	-4.71409100	1.68196000
H	-2.85884100	-6.93986800	2.76871500
H	-1.27081700	-6.16491700	2.91659100
H	-0.36714200	-7.57566100	1.08944400
H	-3.13821500	-8.93079300	1.05786700
H	-2.38750000	-8.01750100	-0.28552200
H	-1.68161200	-9.56031300	0.24438900
H	-1.74029700	-9.20998400	3.31772300
H	-0.31610800	-9.72303200	2.37333200
H	-0.19667200	-8.32672700	3.47994600
H	0.68330700	-0.88211600	-6.71519900
H	1.48290900	0.46502100	-5.85044800
H	-0.89708300	0.67101200	-7.79387300
H	0.78457400	1.09414400	-8.16278100
H	-1.00166000	2.49610600	-6.09189800
H	-0.10330300	3.43138500	-8.88756900
H	-1.74809100	2.89640200	-8.44601600
H	-1.07027000	4.38439700	-7.72887000
H	1.82178300	3.25771500	-7.07716800
H	0.79408400	4.24927000	-6.01040900
H	1.41169700	2.67955700	-5.43992000
C	2.00166300	1.42761100	3.16113100
C	3.00576700	2.16948500	2.52446400
C	2.67054200	3.26434100	1.68096000
C	1.34912100	3.60126500	1.44545900
C	0.28578500	2.93830500	2.12410400
C	0.64049700	1.87191800	3.03622700
C	-1.08238800	3.35834700	1.98233500
C	-2.02464200	2.87688600	2.90244700
C	-1.67313300	1.85630800	3.82627000
C	-0.39355900	1.33513100	3.85437400
O	-3.28582500	3.40557300	2.85185500
O	4.29985800	1.77509900	2.73119300
C	-4.25039800	3.05338900	3.84901800
C	-5.53135200	3.82952800	3.57728600
C	-6.27536700	3.46870500	2.27487200
C	-7.42711400	4.45430800	2.03055500
C	-6.78934000	2.02086200	2.28154500
C	5.39063000	2.61126700	2.33750300
C	6.66883000	2.04576700	2.94488700
C	6.99055700	0.57712500	2.59983900
C	8.26341200	0.12926700	3.33248400
C	7.11242300	0.34040000	1.08846800
C	2.35296700	0.18601700	3.97427700
H	3.45894600	3.83455000	1.18745500
H	1.12959700	4.41355900	0.75091500
H	-2.41629100	1.46353800	4.52157200
H	-0.17222000	0.54097400	4.56577100
H	-3.84967500	3.30659500	4.85084000
H	-4.43197300	1.96034700	3.82885600
H	-5.28324500	4.90738100	3.57329000
H	-6.20717600	3.66755900	4.44026800
H	-5.55824600	3.57527300	1.43818200
H	-7.95165700	4.22998000	1.08453800
H	-8.17168100	4.40221100	2.84742400
H	-7.06126800	5.49534200	1.97636600
H	-7.52152200	1.86591400	3.09646500
H	-7.29579400	1.77841600	1.32989000
H	-5.97589900	1.28635700	2.42231700
H	5.21918400	3.64354700	2.70143000
H	5.45245500	2.65344700	1.23180800
H	6.60658100	2.15001800	4.04423900
H	7.50490900	2.69415100	2.61517700
H	6.15071800	-0.04075800	2.97283600
H	9.14016800	0.71378100	2.99482400
H	8.16961400	0.26681600	4.42465800
H	8.47876500	-0.93748300	3.14302300
H	7.91815800	0.96004500	0.65087400
H	7.35090500	-0.71679800	0.87393600
H	6.17802000	0.58311900	0.55691700
C	1.98870800	-3.18869600	0.17946800
C	3.18572100	-2.68313700	-0.35038400
C	3.89238300	-1.64568700	0.31912300
C	3.40462100	-1.09094100	1.48864700
C	2.22308500	-1.59143200	2.10918400
C	1.54254400	-2.69161900	1.45625100
C	1.76822500	-1.08807900	3.37635500
C	0.76069700	-1.79192400	4.05295000
C	0.11442600	-2.89660200	3.43307000
C	0.47104700	-3.30778900	2.16233100
O	0.42843600	-1.36360400	5.30669400
O	3.63069800	-3.22399200	-1.52371800
C	-0.50351000	-2.10545100	6.09969100
C	-0.63491100	-1.42502700	7.45502900
C	-1.25249900	-0.01143000	7.44619000
C	-1.17273400	0.60942100	8.84832800
C	-2.69955700	-0.01096100	6.93023100
C	4.89393600	-2.84296400	-2.07295200
C	5.09675200	-3.60429700	-3.37587100
C	5.02114600	-5.14138100	-3.27326300
C	6.02116900	-5.71095200	-2.25693000
C	5.21808800	-5.76912300	-4.66061600
C	1.20954600	-4.27181200	-0.56679500
H	4.83303200	-1.27207100	-0.08867000
H	3.96885900	-0.28405500	1.95870500
H	-0.67796600	-3.43668400	3.95758700
H	-0.04605500	-4.16046500	1.72601300
H	-0.13442200	-3.14303900	6.22415000
H	-1.48134000	-2.15634600	5.58065200
H	0.36931100	-1.38444000	7.91689600
H	-1.24804600	-2.08676100	8.09849700
H	-0.64542600	0.61595000	6.76553100
H	-1.75733500	0.01649600	9.57710000
H	-0.13000500	0.65241700	9.21086700
H	-1.57548200	1.63804500	8.85346100
H	-3.34937400	-0.63214600	7.57543300
H	-3.11539200	1.01263300	6.92828100
H	-2.77865900	-0.40313700	5.90036500
H	5.69640300	-3.06509100	-1.34272000
H	4.90959700	-1.75140300	-2.26520100
H	6.08720200	-3.30902100	-3.77586600
H	4.34778600	-3.25256600	-4.11219400
H	4.00121600	-5.39707200	-2.92480600
H	7.05826600	-5.42259900	-2.51487900
H	5.81455000	-5.36191400	-1.22980000
H	5.97751800	-6.81431100	-2.24186900
H	6.22965600	-5.55158700	-5.05298100
H	5.10402800	-6.86688600	-4.62134500
H	4.48446200	-5.37899700	-5.38970000
C	-1.44189500	3.59961900	-0.51656100
C	-0.54164500	4.05731300	-1.49203300
C	-0.43135400	3.38378300	-2.73937500
C	-1.18127800	2.25276500	-3.01419700
C	-2.15534600	1.77068900	-2.09375600
C	-2.30148500	2.49307100	-0.84453100
C	-3.01457000	0.66085100	-2.41363400
C	-4.10526000	0.39897000	-1.57277700
C	-4.26803200	1.11853100	-0.35805800

C	-3.37560200	2.10744100	0.00830200
O	-4.98727900	-0.56453200	-1.96664300
O	0.21609200	5.15473300	-1.19092200
C	-6.05016600	-0.97264000	-1.09867600
C	-6.79982600	-2.11677100	-1.76682100
C	-7.38005200	-1.81079100	-3.16288800
C	-8.32871900	-0.60332900	-3.15205800
C	-8.07480000	-3.05872700	-3.72641900
C	1.03627300	5.75239000	-2.19889300
C	1.67938600	7.00886300	-1.62945600
C	2.64626700	6.80759200	-0.44494000
C	3.14389400	8.16773000	0.06541700
C	3.82556700	5.89086700	-0.80215900
C	-1.51242700	4.28068800	0.84876900
H	-5.11109600	0.90226300	0.29959300
H	-3.54590600	2.63422900	0.94537800
H	-6.72083400	-0.11328500	-0.90437800
H	-5.63054200	-1.29935800	-0.12531900
H	-7.62050100	-2.41235200	-1.08310700
H	-6.12443700	-2.99138300	-1.83799700
H	-6.52700500	-1.56540100	-3.82546600
H	-9.16618800	-0.76175300	-2.44569500
H	-7.81167100	0.32960400	-2.86574100
H	-8.76533200	-0.44010800	-4.15323100
H	-8.94402200	-3.34255000	-3.10306300
H	-8.44336100	-2.88111700	-4.75228100
H	-7.38841600	-3.92454300	-3.75874900
H	0.41237300	6.00932400	-3.07811100
H	1.80459300	5.02756700	-2.53657600
H	0.87298500	7.70345800	-1.32851900
H	2.22158900	7.50185800	-2.46074900
H	2.07733600	6.32952300	0.37545200
H	3.72605400	8.69372600	-0.71475800
H	2.30172000	8.82198500	0.35357100
H	3.79705900	8.04857700	0.94821900
H	4.40440600	6.30051000	-1.65159100
H	4.51887700	5.79016000	0.05216500
H	3.49475900	4.87477900	-1.08042100
H	-2.54162400	4.61947400	1.04275100
H	-0.88577100	5.18404900	0.82079800
H	1.03283500	-5.12937900	0.09917500
H	1.82910700	-4.64304500	-1.39618900
H	-3.62341000	-0.86874500	-3.79251400
H	-2.62683800	0.38307200	-4.53325900
H	1.98138900	0.29148800	5.00473100
H	3.44823400	0.11015800	4.04452800
C	1.47299100	1.00739900	-0.64516000
H	1.01383500	2.00425100	-0.70093500
H	1.81648000	0.84171300	0.38636100
N	0.36262400	0.01524000	-0.87072100
C	2.62233100	0.84647700	-1.64610200
H	2.87211200	-0.23108000	-1.69014000
C	2.25994200	1.33210000	-3.05385900
C	3.85199300	1.59751300	-1.09863900
H	3.57626100	2.65978500	-0.96682900
H	4.07208300	1.21763200	-0.08759300
C	5.10387800	1.50178000	-1.97139600
H	5.39454900	0.44961800	-2.14503000
H	4.96334400	1.97812400	-2.95671800
H	-0.04789400	0.07668500	-1.82209100
H	0.67900600	-0.96387600	-0.74541200
H	0.25792100	3.74924400	-3.50154700
H	-1.04914400	1.76166600	-3.97917100
H	5.95785000	2.00270100	-1.48449300
H	-0.41815600	0.18092400	-0.22028400
H	3.00234000	0.98831100	-3.79199300
H	1.27690300	0.97387700	-3.38723100
H	2.23311300	2.43415200	-3.08330500

1 2 1.5 6 1.5 23 1.0
2 3 1.5 12 1.0
3 4 2.0 24 1.0
4 5 1.5 25 1.0
5 6 1.0 7 1.5
6 10 1.5
7 8 1.5 121 1.0
8 9 1.5 11 1.0
9 10 2.0 26 1.0
10 27 1.0
11 13 1.0
12 18 1.0
13 14 1.0 28 1.0 29 1.0
14 15 1.0 30 1.0 31 1.0
15 16 1.0 17 1.0 32 1.0
16 33 1.0 34 1.0 35 1.0
17 36 1.0 37 1.0 38 1.0
18 19 1.0 39 1.0 40 1.0
19 20 1.0 41 1.0 42 1.0
20 21 1.0 22 1.0 43 1.0
21 44 1.0 45 1.0 46 1.0
22 47 1.0 48 1.0 49 1.0
23 154 1.0 199 1.0 200 1.0
24
25
26
27
28
29
30
31
32
33
34
35
36
37
38
39
40
41
42
43
44
45
46
47
48
49
50 51 1.5 55 1.5 72 1.0
51 52 1.5 61 1.0
52 53 2.0 73 1.0
53 54 1.5 74 1.0
54 55 1.5 56 1.5
55 59 1.5
56 57 1.5 170 1.0
57 58 1.5 60 1.0
58 59 2.0 75 1.0
59 76 1.0
60 62 1.0
61 67 1.0
62 63 1.0 77 1.0 78 1.0
63 64 1.0 79 1.0 80 1.0
64 65 1.0 66 1.0 81 1.0
65 82 1.0 83 1.0 84 1.0
66 85 1.0 86 1.0 87 1.0
67 68 1.0 88 1.0 89 1.0
68 69 1.0 90 1.0 91 1.0
69 70 1.0 71 1.0 92 1.0
70 93 1.0 94 1.0 95 1.0
71 96 1.0 97 1.0 98 1.0
72 105 1.0 201 1.0 202 1.0
73
74
75
76
77
78
79

80
81
82
83
84
85
86
87
88
89
90
91
92
93
94
95
96
97
98
99 100 1.5 104 1.5 121 1.0
100 101 1.5 110 1.0
101 102 2.0 122 1.0
102 103 1.5 123 1.0
103 104 1.0 105 1.5
104 108 1.5
105 106 1.5
106 107 1.5 109 1.0
107 108 2.0 124 1.0
108 125 1.0
109 111 1.0
110 116 1.0
111 112 1.0 126 1.0 127 1.0
112 113 1.0 128 1.0 129 1.0
113 114 1.0 115 1.0 130 1.0
114 131 1.0 132 1.0 133 1.0
115 134 1.0 135 1.0 136 1.0
116 117 1.0 137 1.0 138 1.0
117 118 1.0 139 1.0 140 1.0
118 119 1.0 120 1.0 141 1.0
119 142 1.0 143 1.0 144 1.0
120 145 1.0 146 1.0 147 1.0
121 197 1.0 198 1.0
122
123
124
125
126
127
128
129
130
131
132
133
134
135
136
137
138
139
140
141
142
143
144
145
146
147
148 149 1.5 153 1.5 170 1.0
149 150 1.5 159 1.0
150 151 2.0 218 1.0
151 152 1.5 219 1.0
152 153 1.0 154 1.5
153 157 1.5
154 155 1.5
155 156 1.5 158 1.0
156 157 2.0 171 1.0
157 172 1.0
158 160 1.0
159 165 1.0
160 161 1.0 173 1.0 174 1.0
161 162 1.0 175 1.0 176 1.0
162 163 1.0 164 1.0 177 1.0
163 178 1.0 179 1.0 180 1.0
164 181 1.0 182 1.0 183 1.0
165 166 1.0 184 1.0 185 1.0
166 167 1.0 186 1.0 187 1.0
167 168 1.0 169 1.0 188 1.0
168 189 1.0 190 1.0 191 1.0
169 192 1.0 193 1.0 194 1.0
170 195 1.0 196 1.0
171
172
173
174
175
176
177
178
179
180
181
182
183
184
185
186
187
188
189
190
191
192
193
194
195
196
197
198
199
200
201
202
203 204 1.0 205 1.0 206 1.0 207 1.0
204
205
206 216 1.0 217 1.0 221 1.0
207 208 1.0 209 1.0 210 1.0
208
209 222 1.0 223 1.0 224 1.0
210 211 1.0 212 1.0 213 1.0
211
212
213 214 1.0 215 1.0 220 1.0
214
215
216
217
218
219
220
221
222
223
224

---

0 imaginary frequency
Energy: -4118.4285 Hartree

Cartesian coordinates of (*S*)-**9⁺** @ *pR*-**PrS[4]**^{iPe}

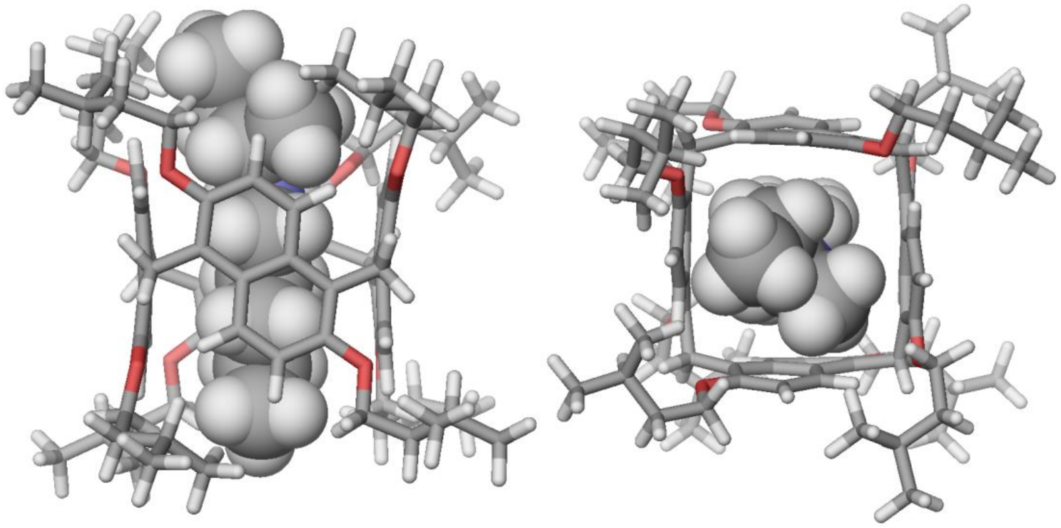

Figure S61: Side and top view of DFT-optimized structure (B97D3/SVP/SVPFIT) of the (*S*)-**9⁺** @ *pR*-**PrS[4]**^{iPe} complex.

C	3.12272700	0.66631000	-1.23167100
H	3.32159400	-0.40674100	-1.07533200
C	2.84494600	0.93768400	-2.70070800
N	1.88992300	0.93535700	-0.38362200
C	4.28459400	1.50037200	-0.67647500
H	4.38459800	1.30922400	0.40615800
C	5.62148300	1.21932500	-1.36550000
H	2.17968900	0.90675500	0.60973700
H	1.56971500	1.90898900	-0.52359200
C	0.71227300	0.00487800	-0.55197900
H	6.42723000	1.79136900	-0.87776200
H	5.89059700	0.15080800	-1.30227700
H	5.61017100	1.50555600	-2.43092800
C	-0.29574500	0.24318000	0.56075300
H	0.28372300	0.19169700	-1.54498400
H	1.11828400	-1.01647900	-0.53045200
C	-1.43069200	-0.77550800	0.58368500
H	0.22693000	0.20839100	1.52870200
H	-0.71292200	1.25535100	0.45964600
C	-2.43464500	-0.51052600	1.70736700
H	-1.95288800	-0.76902300	-0.38604700
H	-1.01120200	-1.78586900	0.70286300
C	-3.58127100	-1.52021500	1.72650200
H	-1.90550200	-0.52781500	2.67709200
H	-2.83685600	0.51324400	1.60339700
H	-4.28292700	-1.33167300	2.55962500
H	-4.15567000	-1.48530500	0.78336700
H	-3.19730600	-2.55039600	1.83274100
H	4.02630100	2.56993700	-0.78812100
H	2.76005800	2.01998700	-2.88716400
H	3.68137700	0.54863900	-3.30135000
H	1.93051000	0.44131600	-3.04807500
C	-1.94519800	3.31504100	-0.41799200
C	-3.20664600	2.90800100	0.04066800
C	-3.88471100	1.81810100	-0.57279000
C	-3.32371800	1.13929700	-1.63710800
C	-2.08734900	1.55331300	-2.20765900
C	-1.42069800	2.69163100	-1.60469500
C	-1.54504300	0.90308400	-3.37105000
C	-0.45977900	1.50928800	-4.02248200
C	0.16025100	2.66275200	-3.47017000
C	-0.26597800	3.19849200	-2.26637900
O	-0.01759300	0.92955100	-5.18054000
O	-3.74704500	3.59561800	1.08908500
C	0.92177500	1.60708500	-6.01907800
C	1.07930400	0.81054600	-7.30754400
C	1.59879100	-0.63365800	-7.15396400
C	1.61084900	-1.33820300	-8.51824100
C	2.98444200	-0.69236700	-6.49456000
C	-4.97551300	3.17041000	1.68591300
C	-5.24951600	4.05245800	2.89702700
C	-5.29192900	5.57012300	2.62511600
C	-6.32525400	5.94540600	1.55324700
C	-5.54655800	6.33003200	3.93479500
C	-1.16402100	4.39973200	0.32578100
H	-4.86017800	1.49802800	-0.19913800
H	-3.86719200	0.29663600	-2.06555200
H	1.00156000	3.13127200	-3.98601300
H	0.27170900	4.05607700	-1.85776500
H	0.55040300	2.62703400	-6.24193000
H	1.89133700	1.71155600	-5.49261700
H	0.10100100	0.79063900	-7.82308900
H	1.76735400	1.37997000	-7.96359000
H	0.88767400	-1.17608000	-6.50169400
H	2.30770700	-0.83667200	-9.21623900
H	0.60872100	-1.33222800	-8.98316500
H	1.93252100	-2.39051800	-8.42152300
H	3.73423600	-0.14459800	-7.09644400
H	3.33086500	-1.73737800	-6.40226600
H	2.98105600	-0.25329500	-5.48213100
H	-5.79334100	3.24451500	0.94295100
H	-4.89379000	2.10846500	1.99423000
H	-6.21900900	3.72742500	3.32442900
H	-4.48507900	3.84489300	3.67074200
H	-4.29229600	5.86359700	2.24874500
H	-7.33909700	5.60844600	1.84287500
H	-6.08462800	5.50146200	0.57122000
H	-6.36610200	7.04047100	1.41583400
H	-6.54102900	6.07917800	4.35041000
H	-5.51769200	7.42241200	3.77444600
H	-4.79013900	6.08062600	4.70136500
C	0.91236600	-3.64036500	1.21136700
C	-0.16856200	-3.95401200	2.05242800
C	-0.45840500	-3.14738400	3.18499500
C	0.31248300	-2.04149800	3.49139900
C	1.47438700	-1.72277900	2.74093600
C	1.79050600	-2.57326300	1.60850200
C	2.33978600	-0.62816600	3.11935100
C	3.55685500	-0.50110300	2.44577200
C	3.89915500	-1.36604500	1.37099100
C	3.02989400	-2.34659500	0.93619600
O	4.46073400	0.48510300	2.78876200
O	-0.93070200	-5.03838800	1.72896600
C	5.58919700	0.04731800	3.57101600
C	6.57243800	1.19913800	3.69984600
C	7.14799300	1.73399100	2.37260200

C	8.05637800	2.94313500	2.63826200
C	7.89110800	0.64901200	1.57905300
C	-1.92895700	-5.50939800	2.64339100
C	-2.53727500	-6.78908500	2.08851800
C	-3.32750200	-6.65575900	0.77062100
C	-3.77533000	-8.04153400	0.28348100
C	-4.52853100	-5.70687300	0.90173700
C	1.12725400	-4.42035700	-0.08620100
H	-1.31968700	-3.37642000	3.81197700
H	0.02789100	-1.42504200	4.34363600
H	4.87034600	-1.23079700	0.88733900
H	3.31498500	-2.97094900	0.08886300
H	5.22948300	-0.27965300	4.56769900
H	6.05336100	-0.83222400	3.08375700
H	6.07673100	2.02645900	4.24284700
H	7.40268800	0.85558800	4.34816700
H	6.29179000	2.08042200	1.76039700
H	8.43291000	3.37657500	1.69429500
H	8.93419300	2.65153200	3.24513500
H	7.52005800	3.73842700	3.18701100
H	8.70879500	0.20949400	2.18098900
H	8.34386900	1.06832100	0.66316300
H	7.22337000	-0.17280500	1.26646100
H	-1.45815100	-5.70315000	3.62762400
H	-2.70131700	-4.72940000	2.78962600
H	-1.72485300	-7.52804100	1.95627500
H	-3.20596800	-7.19777100	2.87204300
H	-2.64242200	-6.23836700	0.00801300
H	-4.46542300	-8.51148000	1.00962900
H	-2.91372400	-8.72048800	0.15202100
H	-4.30315000	-7.97317000	-0.68443200
H	-5.23347900	-6.06573700	1.67544900
H	-5.08564800	-5.64644900	-0.05049900
H	-4.22658100	-4.68048300	1.17376000
C	-1.94387000	-1.60360900	-3.00082200
C	-3.04919800	-2.20421500	-2.38143900
C	-2.87089000	-3.25237000	-1.43794200
C	-1.60537200	-3.66443900	-1.06804000
C	-0.44324200	-3.13501200	-1.69440000
C	-0.63969800	-2.14876000	-2.73663700
C	0.87664900	-3.58506900	-1.34088000
C	1.94419100	-3.22625200	-2.17740100
C	1.74341300	-2.32749000	-3.26013600
C	0.49934300	-1.77010900	-3.50051800
O	3.17400400	-3.76416200	-1.90021500
O	-4.28598800	-1.71870300	-2.69991200
C	4.24068000	-3.63634400	-2.84225000
C	5.39182500	-4.52747400	-2.39450700
C	5.98792500	-4.22000800	-1.00607600
C	7.07717400	-5.24421700	-0.65721300
C	6.52929400	-2.78726800	-0.91041000
C	-5.46412000	-2.29726400	-2.13333300
C	-6.66929500	-1.52219000	-2.64822300
C	-6.81845300	-1.45663400	-4.18194700
C	-6.87282300	-2.84931000	-4.82632700
C	-8.05323300	-0.62313100	-4.55355500
C	-2.13622800	-0.39678200	-3.91670700
H	-3.73607900	-3.71461300	-0.96164000
H	-1.50801500	-4.41988500	-0.28893200
H	2.57893200	-2.05012100	-3.90532100
H	0.39449800	-1.05113500	-4.31406900
H	3.88892200	-3.94557800	-3.84636200
H	4.55547200	-2.57455100	-2.91384400
H	5.04353700	-5.57700000	-2.41487600
H	6.18763000	-4.44628200	-3.16141300
H	5.17379700	-4.32658200	-0.26317800
H	7.92080600	-5.18254000	-1.37046600
H	6.68450900	-6.27624000	-0.69093400
H	7.48110300	-5.06859900	0.35584600
H	7.33463400	-2.61367000	-1.64905200
H	6.94804000	-2.58720100	0.09180600
H	5.73988900	-2.03836500	-1.09493400
H	-5.52135300	-3.36746700	-2.41346400
H	-5.41864900	-2.24310600	-1.02704300

H	-7.57222400	-1.99149500	-2.20907800
H	-6.62635600	-0.49212900	-2.24393400
H	-5.92364000	-0.93605500	-4.57578100
H	-7.70884700	-3.44621700	-4.41373800
H	-5.93819100	-3.41621900	-4.66962100
H	-7.02809500	-2.77011000	-5.91694500
H	-8.98023500	-1.10597200	-4.18982100
H	-8.14344400	-0.50949400	-5.64850700
H	-8.00660200	0.38883200	-4.11122400
C	0.84102900	1.32257400	3.83324500
C	-0.42369600	1.21525500	4.43421500
C	-1.51020400	2.00800700	3.97200500
C	-1.36413200	2.84571800	2.88396800
C	-0.10467500	3.02766900	2.25636900
C	1.02972400	2.32667000	2.82090700
C	0.04692500	3.87659000	1.09971600
C	1.34722600	4.17872600	0.68079300
C	2.47669400	3.62467700	1.34431600
C	2.32835400	2.68121100	2.34523300
O	1.57111800	4.98813600	-0.41340400
O	-0.56993000	0.31502000	5.45013500
C	1.89997700	6.36033100	-0.11781100
C	2.04679800	7.12046700	-1.42633100
C	0.77612000	7.21694000	-2.29631200
C	-0.35374600	7.99016300	-1.60045900
C	1.11318900	7.84571500	-3.65625300
C	-1.79040900	0.28432100	6.20290000
C	-1.67728600	-0.77134800	7.29225400
C	-1.59182900	-2.23747300	6.82056900
C	-1.41383400	-3.16829900	8.02895400
C	-2.81108200	-2.65394800	5.98285900
C	1.96533100	0.36466100	4.22214300
H	3.47488200	3.93257000	1.01696300
H	3.21832700	2.22669800	2.78392700
H	1.10504900	6.78992900	0.52381100
H	2.84561900	6.39540400	0.46071300
H	2.40214100	8.14117500	-1.18214700
H	2.85562400	6.64120400	-2.01100300
H	0.41920000	6.18538300	-2.48371000
H	-0.04440100	9.03034500	-1.38431800
H	-0.65661300	7.52599100	-0.64524500
H	-1.25189700	8.03482800	-2.24161900
H	1.48532800	8.88028300	-3.53186900
H	0.22300900	7.88724200	-4.30891000
H	1.89540600	7.27065500	-4.18496500
H	-1.96259300	1.28259700	6.65143800
H	-2.64049600	0.06737800	5.52738300
H	-0.79680900	-0.53289200	7.91788200
H	-2.56406500	-0.65524700	7.94658900
H	-0.68951900	-2.33663900	6.18757900
H	-2.29270500	-3.11848900	8.69925700
H	-0.52375200	-2.89232000	8.62234100
H	-1.29498000	-4.21911600	7.70964200
H	-3.74876900	-2.51899000	6.55454800
H	-2.74898500	-3.72126000	5.70356700
H	-2.90053200	-2.07293900	5.04781900
H	2.86921000	0.93640300	4.48329200
H	1.66058000	-0.17991700	5.12787100
H	-1.68269700	-0.59349600	-4.89964400
H	-3.21430000	-0.26626600	-4.09378800
H	-0.80989100	5.15534000	-0.38983900
H	-1.85130600	4.91318000	1.01470000
H	2.15520800	-4.81047900	-0.12756700
H	0.46190300	-5.29627000	-0.07412800
H	-2.49121900	1.92700400	4.44505100
H	-2.23609700	3.38256800	2.51315300

1 2 1.0 3 1.0 4 1.0 5 1.0
2
3 30 1.0 31 1.0 32 1.0
4 8 1.0 9 1.0 10 1.0
5 6 1.0 7 1.0 29 1.0
6
7 11 1.0 12 1.0 13 1.0

8
9
10 14 1.0 15 1.0 16 1.0
11
12
13
14 17 1.0 18 1.0 19 1.0
15
16
17 20 1.0 21 1.0 22 1.0
18
19
20 23 1.0 24 1.0 25 1.0
21
22
23 26 1.0 27 1.0 28 1.0
24
25
26
27
28
29
30
31
32
33 34 1.5 38 1.5 55 1.0
34 35 1.5 44 1.0
35 36 2.0 56 1.0
36 37 1.5 57 1.0
37 38 1.0 39 1.5
38 42 1.5
39 40 1.5 153 1.0
40 41 1.5 43 1.0
41 42 2.0 58 1.0
42 59 1.0
43 45 1.0
44 50 1.0
45 46 1.0 60 1.0 61 1.0
46 47 1.0 62 1.0 63 1.0
47 48 1.0 49 1.0 64 1.0
48 65 1.0 66 1.0 67 1.0
49 68 1.0 69 1.0 70 1.0
50 51 1.0 71 1.0 72 1.0
51 52 1.0 73 1.0 74 1.0
52 53 1.0 54 1.0 75 1.0
53 76 1.0 77 1.0 78 1.0
54 79 1.0 80 1.0 81 1.0
55 186 1.0 231 1.0 232 1.0
56
57
58
59
60
61
62
63
64
65
66
67
68
69
70
71
72
73
74
75
76
77
78
79
80
81
82 83 1.5 87 1.5 104 1.0
83 84 1.5 93 1.0
84 85 2.0 105 1.0
85 86 1.5 106 1.0
86 87 1.0 88 1.5
87 91 1.5
88 89 1.5 202 1.0
89 90 1.5 92 1.0
90 91 2.0 107 1.0
91 108 1.0
92 94 1.0
93 99 1.0
94 95 1.0 109 1.0 110 1.0
95 96 1.0 111 1.0 112 1.0
96 97 1.0 98 1.0 113 1.0
97 114 1.0 115 1.0 116 1.0
98 117 1.0 118 1.0 119 1.0
99 100 1.0 120 1.0 121 1.0
100 101 1.0 122 1.0 123 1.0
101 102 1.0 103 1.0 124 1.0
102 125 1.0 126 1.0 127 1.0
103 128 1.0 129 1.0 130 1.0
104 137 1.0 233 1.0 234 1.0
105
106
107
108
109
110
111
112
113
114
115
116
117
118
119
120
121
122
123
124
125
126
127
128
129
130
131 132 1.5 136 1.5 153 1.0
132 133 1.5 142 1.0
133 134 2.0 154 1.0
134 135 1.5 155 1.0
135 136 1.0 137 1.5
136 140 1.5
137 138 1.5
138 139 1.5 141 1.0
139 140 2.0 156 1.0
140 157 1.0
141 143 1.0
142 148 1.0
143 144 1.0 158 1.0 159 1.0
144 145 1.0 160 1.0 161 1.0
145 146 1.0 147 1.0 162 1.0
146 163 1.0 164 1.0 165 1.0
147 166 1.0 167 1.0 168 1.0
148 149 1.0 169 1.0 170 1.0
149 150 1.0 171 1.0 172 1.0
150 151 1.0 152 1.0 173 1.0
151 174 1.0 175 1.0 176 1.0
152 177 1.0 178 1.0 179 1.0
153 229 1.0 230 1.0
154
155

156
157
158
159
160
161
162
163
164
165
166
167
168
169
170
171
172
173
174
175
176
177
178
179
180 181 1.5 185 1.5 202 1.0
181 182 1.5 191 1.0
182 183 2.0 235 1.0
183 184 1.5 236 1.0
184 185 1.0 186 1.5
185 189 1.5
186 187 1.5
187 188 1.5 190 1.0
188 189 2.0 203 1.0
189 204 1.0
190 192 1.0
191 197 1.0
192 193 1.0 205 1.0 206 1.0
193 194 1.0 207 1.0 208 1.0
194 195 1.0 196 1.0 209 1.0
195 210 1.0 211 1.0 212 1.0
196 213 1.0 214 1.0 215 1.0
197 198 1.0 216 1.0 217 1.0
198 199 1.0 218 1.0 219 1.0
199 200 1.0 201 1.0 220 1.0
200 221 1.0 222 1.0 223 1.0
201 224 1.0 225 1.0 226 1.0
202 227 1.0 228 1.0
203
204
205
206
207
208
209
210
211
212
213
214
215
216
217
218
219
220
221
222
223
224
225
226
227
228
229
230
231
232
233
234
235
236

0 imaginary frequency
Energy: -4275.4936 Hartree

Cartesian coordinates of (*S*)-**9**$^+$ @ *pS*-**PrS[4]**^{iPe}

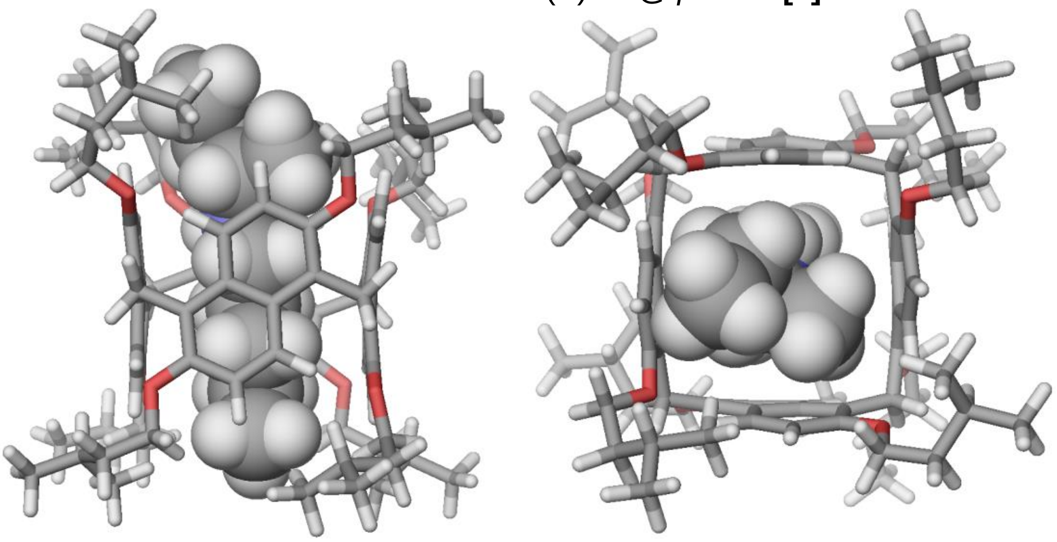

Figure S62: Side and top view of DFT-optimized structure (B97D3/SVP/SVPFIT) of the (*S*)-**9**$^+$ @ *pS*-**PrS[4]**^{iPe} complex.

C	-0.80538900	-2.59886900	-2.81979400
C	-2.17987900	-2.36234800	-2.91087800
C	-2.67764000	-1.09478900	-3.32971400
C	-1.82153200	-0.03840500	-3.58788000

C	-0.40523800	-0.22425600	-3.55736200
C	0.08732100	-1.55284600	-3.25876700
C	0.50580700	0.85787100	-3.81424400
C	1.86514000	0.55085000	-3.99535500
C	2.33312600	-0.78006400	-3.82104400
C	1.47734700	-1.78634300	-3.41745400
O	2.70872500	1.57342000	-4.32062200
O	-3.07427500	-3.31864600	-2.49043800
C	4.07023200	1.28987700	-4.66985100
C	4.77392100	2.59054300	-5.02669300
C	4.97512900	3.59959200	-3.87753300
C	5.63423600	4.87937600	-4.41231900
C	5.78934600	3.00790900	-2.71625700
C	-4.01813900	-3.85685500	-3.43563700
C	-5.33049700	-4.15840800	-2.72320000
C	-5.21396600	-4.99124700	-1.42929200
C	-4.44874600	-6.30605900	-1.63463200
C	-6.60752500	-5.24572000	-0.83697100
C	-0.27380400	-3.90504800	-2.22437300
H	-3.76080000	-0.94990200	-3.39787200
H	-2.24531200	0.93711200	-3.83327100
H	3.38974400	-1.01565200	-3.96546200
H	1.88411800	-2.77976700	-3.23505600
H	4.08436800	0.59588500	-5.53345900
H	4.57550200	0.78497100	-3.82364700
H	4.21418700	3.07428400	-5.84901200
H	5.76395700	2.31744400	-5.44307500
H	3.97574300	3.87563500	-3.49105200
H	6.64573300	4.66781800	-4.80781800
H	5.04119600	5.32624300	-5.23034200
H	5.73745400	5.63806900	-3.61608000
H	6.78140900	2.66247200	-3.06397400
H	5.96301100	3.76706300	-1.93242900
H	5.28282400	2.15066500	-2.23825400
H	-3.57469500	-4.77053000	-3.87795600
H	-4.17988900	-3.13641200	-4.25901000
H	-5.98346600	-4.68906300	-3.44437000
H	-5.83831400	-3.20300200	-2.48856400
H	-4.64524800	-4.38194500	-0.69984400
H	-4.93708500	-6.93379100	-2.40389000
H	-3.40465600	-6.13369300	-1.94916400
H	-4.41769800	-6.89103300	-0.69834600
H	-7.21623000	-5.87351700	-1.51436500
H	-6.53961100	-5.77021600	0.13296500
H	-7.15891500	-4.30163500	-0.67397300
C	-0.18844300	2.80505100	2.98824700
C	-1.56687100	2.74746600	3.24006000
C	-2.17069100	1.53922400	3.68595900
C	-1.42349100	0.38717500	3.85602200
C	-0.00989700	0.39646500	3.68522400
C	0.60856800	1.65526900	3.32429800
C	0.78702500	-0.78456200	3.88706700
C	2.17995800	-0.63771100	3.96410200
C	2.78771500	0.62535200	3.72715900
C	2.02865200	1.71954600	3.36099500
O	2.92209500	-1.75576800	4.23671200
O	-2.30165100	3.88335700	3.02169800
C	4.32659300	-1.63600800	4.48770000
C	4.88358500	-3.01475300	4.81107800
C	4.82396600	-4.05505100	3.67361800
C	5.31518400	-5.41910700	4.17966500
C	5.61795800	-3.61301300	2.43485100
C	-3.63446100	3.98359200	3.53111600
C	-4.13389900	5.40442300	3.30860700
C	-4.22957100	5.87001400	1.84123000
C	-4.65863300	7.34323800	1.78247800
C	-5.17274400	4.99041500	1.00729100
C	0.43623900	4.05509500	2.36870600
H	-3.24482300	1.50372200	3.87713900
H	-1.93509700	-0.52744700	4.15856000
H	3.87116000	0.73304200	3.78385600
H	2.53887900	2.64939900	3.11093000
H	4.48791200	-0.94486100	5.33880300
H	4.83229900	-1.19969200	3.60420400

H	4.34620300	-3.40691400	5.69479600
H	5.93810500	-2.87717400	5.12249800
H	3.76328500	-4.16886500	3.37831100
H	5.23458800	-6.19025100	3.39268600
H	6.37573600	-5.36676700	4.49085400
H	4.72726100	-5.76124800	5.05022800
H	6.68330200	-3.44943300	2.68486700
H	5.57943300	-4.38747100	1.64745600
H	5.23037500	-2.67572800	1.99800800
H	-3.63394300	3.74033900	4.61215200
H	-4.29035100	3.24776200	3.02491800
H	-3.47431900	6.09464400	3.86706900
H	-5.13418900	5.47742200	3.78002900
H	-3.21667300	5.79765000	1.40054300
H	-5.67017500	7.47759800	2.21034100
H	-3.96548300	7.98750200	2.35252200
H	-4.68359400	7.71119500	0.74117800
H	-6.19728400	4.99979800	1.42543500
H	-5.23619700	5.35867700	-0.03250500
H	-4.83629500	3.94022400	0.96639000
C	-0.42941200	2.87606600	-2.51908400
C	-1.78236800	3.15172200	-2.30452100
C	-2.24133200	3.65713200	-1.05940700
C	-1.37839600	3.83148700	0.00284200
C	0.02079100	3.59771200	-0.14664400
C	0.49592200	3.17248700	-1.44930900
C	0.93324400	3.83301100	0.93963300
C	2.30841800	3.84027400	0.65596500
C	2.77860300	3.48705200	-0.63734400
C	1.90168400	3.11600300	-1.64051200
O	3.16432000	4.17203700	1.66778100
O	-2.71153500	2.90569300	-3.29502800
C	4.55178100	4.38941900	1.38706500
C	5.23977200	4.86476500	2.65864700
C	5.27762100	3.86301500	3.83101400
C	5.89907300	4.52566100	5.06913800
C	6.02152400	2.56792700	3.47136500
C	-3.31646900	4.06069600	-3.90422500
C	-4.23201800	3.59563100	-5.02624300
C	-3.55202500	2.82325800	-6.17576200
C	-2.48670500	3.66106600	-6.89790200
C	-4.60953700	2.30949200	-7.16371600
C	0.03136600	2.30830900	-3.86341100
H	-3.30500800	3.88416300	-0.94594900
H	-1.77565400	4.17544000	0.95771100
H	3.84877600	3.46699300	-0.84203400
H	2.30800300	2.80181200	-2.60158900
H	4.64749500	5.15327100	0.58984700
H	5.01083700	3.45458800	1.01009300
H	4.74631000	5.79765300	2.98995000
H	6.27738200	5.14093200	2.38436600
H	4.23222800	3.59936300	4.08166400
H	6.95030200	4.81310600	4.87751900
H	5.34872600	5.43961200	5.35623200
H	5.89175700	3.84014100	5.93532300
H	7.06768600	2.78031500	3.18008600
H	6.05252300	1.88105800	4.33638000
H	5.54432300	2.02678600	2.63608300
H	-2.51579400	4.73053700	-4.27559800
H	-3.89514400	4.62685100	-3.14628500
H	-4.73827400	4.49205400	-5.43617700
H	-5.02715600	2.96470900	-4.58382300
H	-3.04985400	1.94048400	-5.73337700
H	-2.93345200	4.57230600	-7.33896300
H	-1.67276600	3.97961500	-6.22297600
H	-2.02475600	3.08488700	-7.71920500
H	-5.14850000	3.15126100	-7.63828300
H	-4.14719300	1.71218600	-7.96970900
H	-5.36027900	1.67442400	-6.65863200
C	-0.50976200	-2.68802400	2.73109400
C	-1.89803200	-2.85582800	2.67173100
C	-2.52873400	-3.30067500	1.47870100
C	-1.80184100	-3.54852300	0.32887900
C	-0.38001300	-3.43052100	0.32371100

C	0.26329000	-3.03936400	1.56415800
C	0.38945800	-3.72301400	-0.85623800
C	1.78429000	-3.81147300	-0.72475900
C	2.41839700	-3.47871200	0.50312800
C	1.68374500	-3.06461300	1.59735900
O	2.49697500	-4.21788200	-1.81244400
O	-2.67500300	-2.52028500	3.75597000
C	3.92921700	-4.20127300	-1.79013200
C	4.43409200	-4.59454600	-3.17144000
C	3.94449400	-5.95827800	-3.70001300
C	4.29676400	-7.11454500	-2.75301100
C	4.50083600	-6.20413100	-5.10980600
C	-3.53577500	-3.53329700	4.30549900
C	-4.46902900	-2.92419200	5.34226200
C	-5.60762800	-2.03023500	4.80475400
C	-5.10787100	-0.67860200	4.27063300
C	-6.67568100	-1.81746700	5.88874600
C	0.14858400	-2.16682200	4.00958900
H	3.50401800	-3.52955600	0.58951400
H	2.21500500	-2.78691300	2.50734200
H	4.29482600	-4.90234000	-1.01499500
H	4.28493500	-3.18618400	-1.52234100
H	5.54140600	-4.59281100	-3.12590500
H	4.15266600	-3.80337500	-3.89311000
H	2.84046900	-5.90428300	-3.77258500
H	5.38919300	-7.17090500	-2.58351300
H	3.80308100	-7.01253300	-1.77042700
H	3.97682800	-8.08135600	-3.18024100
H	5.60504300	-6.27514700	-5.09215500
H	4.11233000	-7.14729800	-5.53318800
H	4.22848600	-5.38559700	-5.80097600
H	-2.90052600	-4.31794400	4.76375800
H	-4.12542900	-4.01274200	3.49875000
H	-3.86926500	-2.36577100	6.08761400
H	-4.91804400	-3.77607200	5.88762800
H	-6.08396500	-2.57680000	3.96276400
H	-4.61820900	-0.10468000	5.07937300
H	-4.37078500	-0.80557000	3.46421500
H	-5.94806300	-0.07124500	3.88730300
H	-6.24400200	-1.30003300	6.76622200
H	-7.50921100	-1.19773200	5.51258000
H	-7.09717100	-2.77683000	6.23871400
H	0.92629300	-2.87552800	4.33233800
H	-0.61857800	-2.15638300	4.79908100
H	0.84961000	2.92495700	-4.26397400
H	-0.80780100	2.39760100	-4.56885100
H	0.46028400	-4.35632400	-2.90801800
H	-1.11334200	-4.61262400	-2.14929500
H	1.28495900	4.39686000	2.97939200
H	-0.30954600	4.86379200	2.39137000
C	-3.39238200	-0.23557500	0.44831900
H	-3.20306800	-0.69544300	1.43229100
C	-3.79528500	1.21995300	0.61804500
N	-2.06033200	-0.35084400	-0.27156900
C	-4.44566600	-1.05308800	-0.31396900
H	-4.00304600	-2.00181600	-0.65740200
C	-5.69685100	-1.34959900	0.51623000
H	-1.89751700	-1.35935400	-0.44611300
H	-2.12477700	0.06755500	-1.21789100
H	-3.61478900	-3.43167200	1.47018200
H	-2.32721600	-3.87417800	-0.56970300
C	-0.84348500	0.17335600	0.44821100
H	-6.41066700	-1.95235800	-0.06961300
H	-5.44741100	-1.91760700	1.42906900
H	-6.21899700	-0.42987300	0.83075700
C	0.41401100	-0.10297100	-0.35946000
H	-0.99126600	1.24754800	0.62398400
H	-0.82696500	-0.33827000	1.41895600
C	1.69711500	0.21192400	0.40300900
H	0.42703700	-1.16262700	-0.65647800
H	0.38629500	0.48800800	-1.28569600
C	2.95725700	-0.07650400	-0.41515400
H	1.69563900	1.26800300	0.71135100
H	1.72218100	-0.38049900	1.33146800
C	4.24109500	0.16595500	0.37742800
H	2.92850700	-1.12180600	-0.77287600
H	2.95299100	0.55155700	-1.32374000
H	5.14479600	-0.02271000	-0.23047400
H	4.28717900	1.20752200	0.74072400
H	4.28675700	-0.48733400	1.26721500
H	-4.72621000	-0.49927800	-1.22925000
H	-3.99208300	1.68420900	-0.36207600
H	-4.71935100	1.26698300	1.21486500
H	-3.03118700	1.80989300	1.13952700

1 2 1.5 6 1.5 23 1.0
2 3 1.5 12 1.0
3 4 2.0 24 1.0
4 5 1.5 25 1.0
5 6 1.0 7 1.5
6 10 1.5
7 8 1.5 121 1.0
8 9 1.5 11 1.0
9 10 2.0 26 1.0
10 27 1.0
11 13 1.0
12 18 1.0
13 14 1.0 28 1.0 29 1.0
14 15 1.0 30 1.0 31 1.0
15 16 1.0 17 1.0 32 1.0
16 33 1.0 34 1.0 35 1.0
17 36 1.0 37 1.0 38 1.0
18 19 1.0 39 1.0 40 1.0
19 20 1.0 41 1.0 42 1.0
20 21 1.0 22 1.0 43 1.0
21 44 1.0 45 1.0 46 1.0
22 47 1.0 48 1.0 49 1.0
23 154 1.0 199 1.0 200 1.0
24
25
26
27
28
29
30
31
32
33
34
35
36
37
38
39
40
41
42
43
44
45
46
47
48
49
50 51 1.5 55 1.5 72 1.0
51 52 1.5 61 1.0
52 53 2.0 73 1.0
53 54 1.5 74 1.0
54 55 1.0 56 1.5
55 59 1.5
56 57 1.5 170 1.0
57 58 1.5 60 1.0
58 59 2.0 75 1.0
59 76 1.0
60 62 1.0
61 67 1.0
62 63 1.0 77 1.0 78 1.0
63 64 1.0 79 1.0 80 1.0

64 65 1.0 66 1.0 81 1.0
65 82 1.0 83 1.0 84 1.0
66 85 1.0 86 1.0 87 1.0
67 68 1.0 88 1.0 89 1.0
68 69 1.0 90 1.0 91 1.0
69 70 1.0 71 1.0 92 1.0
70 93 1.0 94 1.0 95 1.0
71 96 1.0 97 1.0 98 1.0
72 105 1.0 201 1.0 202 1.0
73
74
75
76
77
78
79
80
81
82
83
84
85
86
87
88
89
90
91
92
93
94
95
96
97
98
99 100 1.5 104 1.5 121 1.0
100 101 1.5 110 1.0
101 102 2.0 122 1.0
102 103 1.5 123 1.0
103 104 1.0 105 1.5
104 108 1.5
105 106 1.5
106 107 1.5 109 1.0
107 108 2.0 124 1.0
108 125 1.0
109 111 1.0
110 116 1.0
111 112 1.0 126 1.0 127 1.0
112 113 1.0 128 1.0 129 1.0
113 114 1.0 115 1.0 130 1.0
114 131 1.0 132 1.0 133 1.0
115 134 1.0 135 1.0 136 1.0
116 117 1.0 137 1.0 138 1.0
117 118 1.0 139 1.0 140 1.0
118 119 1.0 120 1.0 141 1.0
119 142 1.0 143 1.0 144 1.0
120 145 1.0 146 1.0 147 1.0
121 197 1.0 198 1.0
122
123
124
125
126
127
128
129
130
131
132
133
134
135
136
137
138
139
140
141
142
143
144
145
146
147
148 149 1.5 153 1.5 170 1.0
149 150 1.5 159 1.0
150 151 2.0 212 1.0
151 152 1.5 213 1.0
152 153 1.0 154 1.5
153 157 1.5
154 155 1.5
155 156 1.5 158 1.0
156 157 2.0 171 1.0
157 172 1.0
158 160 1.0
159 165 1.0
160 161 1.0 173 1.0 174 1.0
161 162 1.0 175 1.0 176 1.0
162 163 1.0 164 1.0 177 1.0
163 178 1.0 179 1.0 180 1.0
164 181 1.0 182 1.0 183 1.0
165 166 1.0 184 1.0 185 1.0
166 167 1.0 186 1.0 187 1.0
167 168 1.0 169 1.0 188 1.0
168 189 1.0 190 1.0 191 1.0
169 192 1.0 193 1.0 194 1.0
170 195 1.0 196 1.0
171
172
173
174
175
176
177
178
179
180
181
182
183
184
185
186
187
188
189
190
191
192
193
194
195
196
197
198
199
200
201
202
203 204 1.0 205 1.0 206 1.0 207 1.0
204
205 234 1.0 235 1.0 236 1.0
206 210 1.0 211 1.0 214 1.0
207 208 1.0 209 1.0 233 1.0
208
209 215 1.0 216 1.0 217 1.0
210
211

212
213
214 218 1.0 219 1.0 220 1.0
215
216
217
218 221 1.0 222 1.0 223 1.0
219
220
221 224 1.0 225 1.0 226 1.0
222
223
224 227 1.0 228 1.0 229 1.0
225
226
227 230 1.0 231 1.0 232 1.0
228
229
230
231
232
233
234
235
236

0 imaginary frequency
Energy: -4275.4937 Hartree

Cartesian coordinates of (*S*)-**6**$^{2+}$ @ *pR*-**PrS[4]**^{iPe}

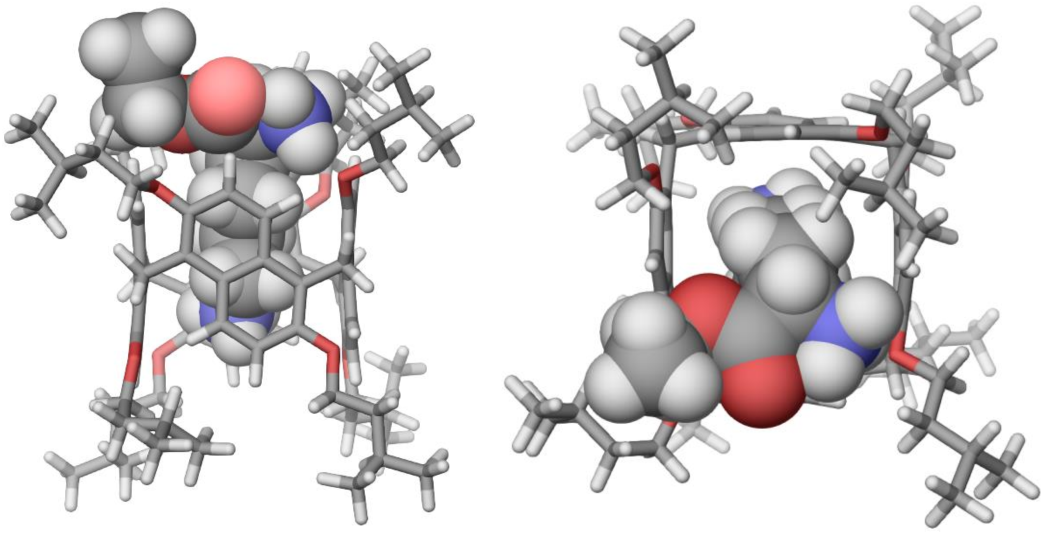

Figure S63: Side and top view of DFT-optimized structure (B97D3/SVP/SVPFIT) of the (*S*)-**6**$^{2+}$ @ *pR*-**PrS[4]**^{iPe} complex.

O	-2.35381200	-4.74564600	-0.29079300
O	2.63064400	-1.07382800	4.41156100
O	-2.92214200	0.21268500	-4.41566600
C	-1.56060900	-2.57362300	-2.41872100
H	-1.92705100	-3.46038300	-1.90217100
C	-0.17692100	-0.95518900	4.64361200
H	-0.84884700	-1.63241400	5.19129300
C	0.44351300	-3.02948200	1.37078800
C	1.86107400	-2.90258100	1.29018400
H	2.40479800	-3.32779600	0.44446700
C	2.57853500	-2.28624400	2.29883400
H	3.66440100	-2.23478200	2.21904400
C	1.93117300	-1.69312900	3.41961900
C	0.53274400	-1.71008700	3.51981900
C	-0.20743000	-2.44754500	2.53018200
C	-1.60476000	-2.68252200	2.68019600
H	-2.11624400	-2.32035800	3.57046000
C	-2.31685600	-3.43951400	1.76387100
H	-3.37307900	-3.63834000	1.95687400
C	-1.68042700	-3.98439600	0.61448100
C	-0.31314500	-3.74615000	0.38041600
C	4.05365600	-1.23723800	4.49989900
H	4.53773600	-0.76608300	3.62047600
H	4.29813300	-2.31726400	4.47999400
C	4.53652400	-0.61039600	5.79912400
H	4.07369600	-1.15567700	6.64222700
H	5.62596600	-0.80066500	5.86624200
C	4.27191300	0.89952400	5.96455200
H	3.17554700	1.05260700	5.93244700
C	4.89810100	1.72989300	4.83479100
H	4.49304700	1.46005200	3.84236500
H	4.70789400	2.80660200	4.99264300
H	5.99522100	1.59172900	4.79820000
C	4.76965200	1.37588100	7.33678700
H	5.86514600	1.25137900	7.42507100
H	4.53876100	2.44382800	7.49740800
H	4.30036800	0.80218700	8.15538200
C	-3.70088800	-5.17036300	-0.01786500
H	-3.70877500	-5.75489400	0.92275500
H	-4.34741900	-4.28356500	0.13131300
C	-4.19491000	-6.01847400	-1.17916200
H	-5.17672900	-6.43447500	-0.87889700
H	-3.51487300	-6.88300000	-1.29313500
C	-4.34422400	-5.29970800	-2.53647200
H	-3.34951200	-4.90382800	-2.81971700
C	-4.76981100	-6.30322800	-3.61826200
H	-5.76272500	-6.73331600	-3.38938600
H	-4.83425900	-5.81944800	-4.60893500
H	-4.05247800	-7.13942300	-3.69611300
C	-5.32590600	-4.11914400	-2.46970500
H	-5.42913400	-3.63815500	-3.45920500
H	-6.33304700	-4.45768700	-2.16218200
H	-5.00308700	-3.34114400	-1.75351700
C	-0.16535100	-2.31311900	-2.48705000
C	0.26714100	-1.13492000	-3.20969500
C	1.66734300	-0.97561200	-3.43441300
H	2.02992200	-0.12023900	-4.00518300
C	-2.47449900	-1.76126800	-3.06512300

H	-3.52725700	-2.04702000	-3.06509700
C	-2.05082300	-0.58238100	-3.73747500
C	-0.69874100	-0.19699100	-3.71517000
C	0.35187300	-4.27755400	-0.88628500
H	1.23802200	-4.86271100	-0.60219300
H	-0.34340000	-4.97406000	-1.37909800
C	0.79171800	-3.19109300	-1.86387100
C	2.57029000	-1.91265100	-2.96939000
H	3.63920000	-1.82248000	-3.18456700
C	2.14306800	-2.99622800	-2.15606300
O	3.11458500	-3.81357400	-1.61698000
C	3.41450700	-5.00916400	-2.37652800
H	2.49214200	-5.61456900	-2.47412100
H	3.72760300	-4.71475900	-3.39764300
C	4.52261100	-5.77673000	-1.67306000
H	4.75282700	-6.66778800	-2.28899400
H	5.44025500	-5.15786600	-1.68621800
C	4.21576500	-6.21721600	-0.22634400
H	3.86516500	-5.32277300	0.32624800
C	5.49324900	-6.72363700	0.45908300
H	6.28326000	-5.94963200	0.46938300
H	5.29673000	-7.02111800	1.50429000
H	5.90172900	-7.60581500	-0.06778200
C	3.10477500	-7.27598400	-0.16448100
H	3.41358200	-8.19980400	-0.68805100
H	2.87449600	-7.54560600	0.88125200
H	2.16512700	-6.92901700	-0.62946100
C	-4.33081000	-0.06657000	-4.37901800
H	-4.51357300	-1.06898300	-4.80782100
H	-4.67541500	-0.08037500	-3.32336600
C	-5.05283600	1.01645000	-5.16539400
H	-4.71857800	2.00251700	-4.79007900
H	-4.73895800	0.96140700	-6.22528000
C	-6.59033800	0.93225500	-5.07108400
H	-6.86273900	1.02159900	-3.99713100
C	-7.14115500	-0.40718100	-5.58709300
H	-6.83297500	-0.58059000	-6.63529100
H	-6.79584100	-1.26762500	-4.98567300
H	-8.24475300	-0.41298100	-5.55989500
C	-7.22878500	2.11354400	-5.81647600
H	-8.32758000	2.10313000	-5.71055400
H	-6.85909800	3.08243600	-5.43464300
H	-6.99598700	2.06724300	-6.89647900
C	-0.29143000	1.19445100	-4.19421600
H	-1.10840800	1.60988500	-4.80350800
O	1.48317800	4.84169800	1.97222500
O	-2.98961300	-0.86845300	4.77658600
O	2.41128000	2.11687100	-3.56998200
C	0.71224300	3.86011400	-0.83476500
H	1.00543600	4.51118400	-0.01184800
H	0.57775300	-0.61356500	5.36649200
C	-1.11809700	2.39707400	2.86947400
C	-2.53511200	2.23899000	2.83004500
H	-3.15320300	2.98836100	2.33475800
C	-3.16023600	1.18054900	3.47283500
H	-4.25260600	1.13925200	3.48348100
C	-2.40111700	0.17370100	4.13040600
C	-0.99343000	0.22646500	4.12377600
C	-0.35354400	1.39436100	3.58292700
C	1.04668400	1.62145400	3.73078500
H	1.66955600	0.88839700	4.24400400
C	1.63585500	2.77257000	3.24828100
H	2.70522900	2.92516200	3.40745300
C	0.88649800	3.73934800	2.51907800
C	-0.47360300	3.53132500	2.25709800
C	-4.35845800	-1.20545100	4.49876300
H	-4.49989700	-1.23865700	3.39942000
H	-5.02989400	-0.42303800	4.90330300
C	-4.65905400	-2.55307400	5.13972100
H	-3.87552000	-3.27803100	4.84566100
H	-4.58255800	-2.44035300	6.23624200
C	-6.04394600	-3.12103300	4.76348000
H	-6.79295100	-2.31400200	4.90360200
C	-6.41930900	-4.27818500	5.70080000

H	-6.44436400	-3.95109700	6.75506100
H	-7.41272100	-4.68891100	5.44939900
H	-5.68446100	-5.10124200	5.62064100
C	-6.10377800	-3.57450800	3.29427400
H	-5.39672000	-4.40822700	3.11874700
H	-7.11296100	-3.93808300	3.03354200
H	-5.85622100	-2.76185500	2.58638000
C	2.57503100	5.53262300	2.60556800
H	2.15795000	6.42121900	3.11519500
H	3.04735600	4.90059500	3.37636800
C	3.57177500	5.94868100	1.52938600
H	4.30755400	6.64045400	1.98231000
H	3.02264600	6.53459400	0.76881600
C	4.30641000	4.77300500	0.85161100
H	3.53420000	4.01242700	0.62006800
C	4.94534800	5.21644000	-0.47209400
H	5.69871300	6.00814400	-0.30830500
H	5.48431700	4.37967200	-0.96518200
H	4.19617800	5.61306000	-1.18045900
C	5.35053600	4.13484700	1.77839300
H	5.83636900	3.26091700	1.30430700
H	6.15108700	4.85561000	2.02498300
H	4.91238800	3.78615800	2.72747100
C	-0.63957400	3.42404700	-0.96775400
C	-0.98855700	2.64532400	-2.14112600
C	-2.36693700	2.37496400	-2.35285600
H	-2.68089900	1.84445700	-3.24997300
C	1.65876200	3.48367900	-1.76123200
H	2.68306400	3.86012000	-1.67931400
C	1.33356600	2.59745800	-2.81922600
C	0.03080100	2.16447200	-3.05105700
C	-1.24268900	4.46059000	1.32305600
H	-2.15768000	4.82248800	1.81349900
H	-0.62338700	5.34834100	1.12706900
C	-1.63020900	3.78797800	0.00566900
C	-3.33664000	2.81098100	-1.46598700
H	-4.38791700	2.62346400	-1.69965600
C	-2.98084000	3.51286200	-0.28016600
O	-3.90410500	3.95026500	0.61165400
C	-5.30930100	3.75112000	0.38876900
H	-5.60384100	4.24362100	-0.55933300
H	-5.50505200	2.66396500	0.28547500
C	-6.09113600	4.31942000	1.56576700
H	-7.11059500	3.89462200	1.49502800
H	-5.65921300	3.92040700	2.50578200
C	-6.20727800	5.85585900	1.65142000
H	-6.64119700	6.20175600	0.68979300
C	-4.85709000	6.56538000	1.84131100
H	-4.17666700	6.40322000	0.98962100
H	-5.00690400	7.65361800	1.95383700
H	-4.34882100	6.20257600	2.75517100
C	-7.18579200	6.23809600	2.77285900
H	-6.80165200	5.91054000	3.75751800
H	-7.32884800	7.33162600	2.81832900
H	-8.17727200	5.77323900	2.62607600
C	2.80807600	2.93660000	-4.70622800
H	1.91688600	3.08489200	-5.34030100
H	3.12881800	3.93258000	-4.33895500
C	3.92359700	2.22567900	-5.45299400
H	4.75992500	2.03376600	-4.75016000
H	3.55991700	1.23362200	-5.78494500
C	4.47235000	3.00583500	-6.66745500
H	4.81841500	3.99469000	-6.30042700
C	3.39736200	3.24524200	-7.73959500
H	2.97406800	2.28599800	-8.09286300
H	2.56484800	3.87224800	-7.37426100
H	3.83068300	3.75927700	-8.61461900
C	5.68472400	2.26986700	-7.25675200
H	6.12016500	2.83705600	-8.09710500
H	6.47884900	2.12199800	-6.50221600
H	5.39231500	1.27456800	-7.64020700
H	0.58622800	1.12630700	-4.85327800
C	-0.43307400	-0.09449000	-0.11168500
H	-0.70959500	0.47260300	-1.01055800

C	0.80511800	0.46997700	0.55868000
H	-0.29954900	-1.14531300	-0.39246000
N	-1.61954600	-0.05701700	0.80784300
H	0.96343300	-0.02892600	1.52716100
C	2.04909900	0.28575700	-0.31281900
H	0.65295000	1.54186600	0.76846500
H	-1.49601400	-0.72205200	1.60102600
H	-1.77909200	0.88330300	1.22737800
C	3.25154900	1.00344600	0.29542900
H	2.26649400	-0.78870900	-0.42656700
H	1.84014800	0.66929500	-1.32321400
C	4.55254000	0.92734700	-0.50576800
H	3.01211300	2.06725900	0.46583300
H	3.47581200	0.58103700	1.29012900
C	5.10838700	-0.48089300	-0.80078300
N	4.51114200	1.60659200	-1.85477900
H	5.34214700	1.44601800	0.06784700
H	3.58465100	1.64913900	-2.37512600
H	4.85663800	2.57288800	-1.80452800
O	5.10791900	-1.25161100	0.26570700
O	5.54262600	-0.75878800	-1.90782300
C	5.68738700	-2.60646400	0.13213100
C	7.19977300	-2.57286400	0.21085800
H	5.23885900	-3.15618200	0.97224700
H	5.30603800	-3.02943100	-0.80745300
H	7.57698100	-3.61044700	0.18987300
H	7.63762400	-2.03767400	-0.64750800
H	7.54501400	-2.10561900	1.14915300
H	-2.47607900	-0.33359800	0.31406900
H	5.14048900	1.03173700	-2.45982200

1 20 1.0 38 1.0
2 13 1.0 22 1.0
3 60 1.0 86 1.0
4 5 1.0 54 1.5 58 2.0
5
6 7 1.0 14 1.0 109 1.0 116 1.0
7
8 9 1.5 15 1.0 21 1.5
9 10 1.0 11 2.0
10
11 12 1.0 13 1.5
12
13 14 1.5
14 15 1.5
15 16 1.5
16 17 1.0 18 2.0
17
18 19 1.0 20 1.5
19
20 21 1.5
21 62 1.0
22 23 1.0 24 1.0 25 1.0
23
24
25 26 1.0 27 1.0 28 1.0
26
27
28 29 1.0 30 1.0 34 1.0
29
30 31 1.0 32 1.0 33 1.0
31
32
33
34 35 1.0 36 1.0 37 1.0
35
36
37
38 39 1.0 40 1.0 41 1.0
39
40
41 42 1.0 43 1.0 44 1.0
42
43
44 45 1.0 46 1.0 50 1.0
45
46 47 1.0 48 1.0 49 1.0
47
48
49
50 51 1.0 52 1.0 53 1.0
51
52
53
54 55 1.0 65 1.5
55 56 1.5 61 1.5
56 57 1.0 66 2.0
57
58 59 1.0 60 1.5
59
60 61 1.5
61 102 1.0
62 63 1.0 64 1.0 65 1.0
63
64
65 68 1.5
66 67 1.0 68 1.5
67
68 69 1.0
69 70 1.0
70 71 1.0 72 1.0 73 1.0
71
72
73 74 1.0 75 1.0 76 1.0
74
75
76 77 1.0 78 1.0 82 1.0
77
78 79 1.0 80 1.0 81 1.0
79
80
81
82 83 1.0 84 1.0 85 1.0
83
84
85
86 87 1.0 88 1.0 89 1.0
87
88
89 90 1.0 91 1.0 92 1.0
90
91
92 93 1.0 94 1.0 98 1.0
93
94 95 1.0 96 1.0 97 1.0
95
96
97
98 99 1.0 100 1.0 101 1.0
99
100
101
102 103 1.0 163 1.0 204 1.0
103
104 122 1.0 140 1.0
105 115 1.0 124 1.0
106 162 1.0 188 1.0
107 108 1.0 156 1.5 160 2.0
108
109
110 111 1.5 117 1.0 123 1.5
111 112 1.0 113 1.5
112
113 114 1.0 115 1.5
114
115 116 1.5
116 117 1.5
117 118 1.5

118 119 1.0 120 2.0
119
120 121 1.0 122 1.5
121
122 123 1.5
123 164 1.0
124 125 1.0 126 1.0 127 1.0
125
126
127 128 1.0 129 1.0 130 1.0
128
129
130 131 1.0 132 1.0 136 1.0
131
132 133 1.0 134 1.0 135 1.0
133
134
135
136 137 1.0 138 1.0 139 1.0
137
138
139
140 141 1.0 142 1.0 143 1.0
141
142
143 144 1.0 145 1.0 146 1.0
144
145
146 147 1.0 148 1.0 152 1.0
147
148 149 1.0 150 1.0 151 1.0
149
150
151
152 153 1.0 154 1.0 155 1.0
153
154
155
156 157 1.0 167 1.5
157 158 1.5 163 1.0
158 159 1.0 168 2.0
159
160 161 1.0 162 1.5
161
162 163 1.5
163
164 165 1.0 166 1.0 167 1.0
165
166
167 170 1.5
168 169 1.0 170 1.5
169
170 171 1.0
171 172 1.0
172 173 1.0 174 1.0 175 1.0
173
174
175 176 1.0 177 1.0 178 1.0
176
177
178 179 1.0 180 1.0 184 1.0
179
180 181 1.0 182 1.0 183 1.0
181
182
183
184 185 1.0 186 1.0 187 1.0
185
186
187
188 189 1.0 190 1.0 191 1.0
189
190
191 192 1.0 193 1.0 194 1.0
192
193
194 195 1.0 196 1.0 200 1.0
195
196 197 1.0 198 1.0 199 1.0
197
198
199
200 201 1.0 202 1.0 203 1.0
201
202
203
204
205 206 1.0 207 1.0 208 1.0 209 1.0
206
207 210 1.0 211 1.0 212 1.0
208
209 213 1.0 214 1.0 235 1.0
210
211 215 1.0 216 1.0 217 1.0
212
213
214
215 218 1.0 219 1.0 220 1.0
216
217
218 221 1.0 222 1.0 223 1.0
219
220
221 226 1.5 227 2.0
222 225 1.0 236 1.0
223
224
225
226 228 1.0
227
228 229 1.0 230 1.0 231 1.0
229 232 1.0 233 1.0 234 1.0
230
231
232
233
234
235
236

---

0 imaginary frequency
Energy: -4440.9063 Hartree

Cartesian coordinates of (*S*)-**6**$^{2+}$ @ *pS*-**PrS[4]**^{iPe}

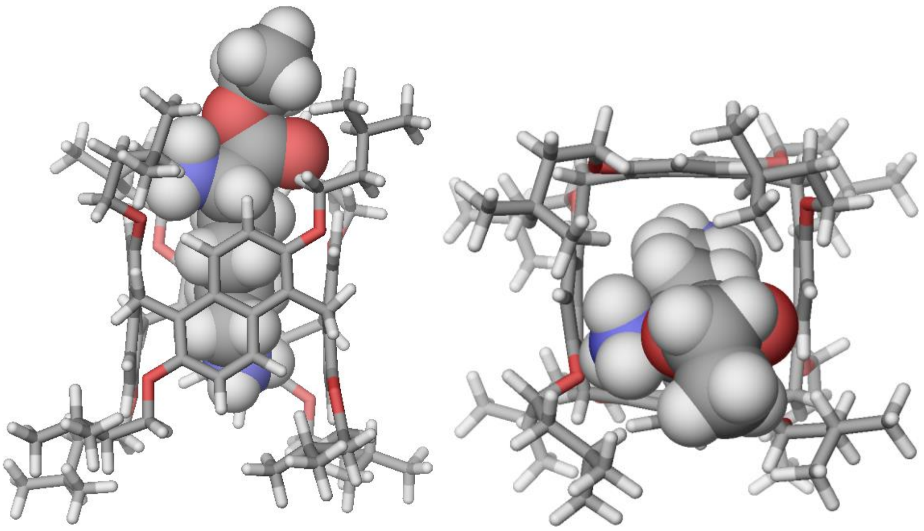

Figure S64: Side and top view of DFT-optimized structure (B97D3/SVP/SVPFIT) of the (*S*)-**6**$^{2+}$ @ *pS*-**PrS[4]**^{iPe} complex.

O	2.69445400	-1.71255000	3.97992400
O	-3.62197800	2.63332700	2.74028200
O	2.91109200	-3.68931900	-2.33301100
C	1.82609500	-3.08377400	1.14592400
H	2.27289000	-2.79014100	2.09652500
C	-0.98809400	3.45197500	2.14466600
H	-0.19312800	3.92241000	2.74152700
C	-0.60754000	-0.25291200	3.32134700
C	-1.98428500	-0.61237600	3.37008200
H	-2.27424800	-1.63817000	3.59635600
C	-2.97808200	0.33424200	3.20941200
H	-4.01809400	0.03486500	3.34833300
C	-2.65804400	1.68236800	2.88680900
C	-1.32011800	2.07289200	2.71000000
C	-0.28606600	1.13575900	3.06612300
C	1.06841400	1.53008100	3.25078700
H	1.34497100	2.57777100	3.13608600
C	2.04189300	0.61609600	3.62172300
H	3.04921400	0.97457400	3.85233700
C	1.74184000	-0.77376600	3.71873100
C	0.42611600	-1.22655400	3.53371300
C	-5.01181700	2.26807900	2.74345200
H	-5.17425000	1.43565000	2.02859100
H	-5.29186200	1.90744600	3.75140600
C	-5.83581200	3.48027000	2.33392100
H	-6.89913800	3.16957600	2.35965500
H	-5.61379100	3.72293000	1.27696100
C	-5.65199600	4.74766200	3.19280700
H	-4.58983900	5.05205400	3.10732200
C	-6.51785900	5.88430200	2.63014500
H	-6.28770000	6.08127400	1.56679100
H	-6.35773200	6.82237300	3.18977900
H	-7.59295000	5.63302900	2.69991800
C	-5.95314500	4.49778000	4.67746700
H	-6.98503800	4.12290200	4.81591400
H	-5.85989600	5.43237600	5.25764400
H	-5.25938500	3.76485000	5.12641900
C	4.08027200	-1.35627000	3.96870200
H	4.29068000	-0.66134300	4.80415800
H	4.31846900	-0.83632900	3.01837900
C	4.90530400	-2.63028000	4.07881200
H	4.75003100	-3.07979000	5.07805900
H	4.51301400	-3.35582300	3.34199100
C	6.41113300	-2.41801500	3.82306100
H	6.51196200	-2.01688900	2.79280700
C	7.15205300	-3.76176100	3.87582000
H	7.08730100	-4.20769100	4.88550600
H	8.22224600	-3.63650200	3.63394300
H	6.72423500	-4.48702800	3.16059800
C	7.03897500	-1.40581800	4.79515300
H	8.12510700	-1.31147100	4.61833500
H	6.90167800	-1.72849200	5.84403900
H	6.60111500	-0.39525900	4.69698600
C	0.42623100	-3.34220900	1.06670700
C	-0.10057600	-3.75195600	-0.22121700
C	-1.47589700	-4.12267100	-0.27402200
H	-1.91198100	-4.48971500	-1.20441700
C	2.64762200	-3.24502200	0.04823400
H	3.71863900	-3.06422700	0.16587600
C	2.11931500	-3.60791800	-1.22520400
C	0.74115200	-3.81931100	-1.38831300
C	0.10837900	-2.71995500	3.55486500
H	-0.63899800	-2.93217600	4.33429300
H	1.01891300	-3.26551500	3.84274300
C	-0.41779600	-3.25758100	2.22554400
C	-2.26767200	-4.12190500	0.86669800
H	-3.29623300	-4.47775000	0.78870000
C	-1.74840900	-3.70496000	2.12165900
O	-2.50077900	-3.71671500	3.25596600
C	-3.79874000	-4.33363800	3.25617800
H	-3.69603400	-5.38918400	2.93671100
H	-4.45358200	-3.82208100	2.52320300
C	-4.37818200	-4.25312600	4.65987000
H	-3.69405900	-4.78216100	5.34891600
H	-5.32232300	-4.83254000	4.65418600
C	-4.65589400	-2.83537500	5.20151400
H	-3.69661400	-2.28183700	5.20226000
C	-5.65328300	-2.05933000	4.32754600
H	-5.29328000	-1.93006300	3.29008200
H	-5.83894200	-1.05278000	4.74367600
H	-6.62744600	-2.58108000	4.27902100
C	-5.14810300	-2.91394300	6.65405900
H	-6.10812500	-3.45959400	6.71683000
H	-5.30683400	-1.90629500	7.07726800
H	-4.42079100	-3.44038700	7.29723300
C	4.31244500	-4.01352000	-2.26018000
H	4.40981800	-5.11045200	-2.15141100
H	4.78129400	-3.54121900	-1.37984100
C	4.96479000	-3.51371300	-3.54178300
H	4.36896000	-3.88757500	-4.39489000
H	5.96376500	-3.98088200	-3.63340500
C	5.09661400	-1.97609000	-3.61509600
H	4.16062800	-1.55237100	-3.19946300
C	6.26270500	-1.46221200	-2.75693300

H	7.23181400	-1.79260400	-3.17468900
H	6.20058600	-1.82194600	-1.71613900
H	6.27477600	-0.35593800	-2.72628800
C	5.22992200	-1.49948900	-5.06784700
H	5.32235500	-0.39878700	-5.12381500
H	4.35762700	-1.80059200	-5.67486200
H	6.13122700	-1.92874700	-5.54292100
C	0.14774800	-4.10752500	-2.76877700
H	0.97569100	-4.29755200	-3.46754700
O	-4.05051000	1.88258800	-3.50372200
O	1.77451400	3.93633000	1.25674100
O	-2.62357000	-4.40426900	-3.20802800
C	-2.42974900	-0.77662600	-3.92901100
H	-3.11084600	0.04819800	-4.14473300
H	-1.87431300	4.09724200	2.24523600
C	-0.97918800	2.85948900	-1.74591500
C	0.36984100	3.21593900	-2.03782300
H	0.75247000	3.14078600	-3.05533000
C	1.19608600	3.68559800	-1.04126400
H	2.20759400	4.02404100	-1.28448300
C	0.76467100	3.72475800	0.30961500
C	-0.54523900	3.43229000	0.67678100
C	-1.45314200	3.06996600	-0.39197000
C	-2.84246600	2.89320300	-0.15991000
H	-3.25358600	3.09132300	0.82779500
C	-3.70338300	2.51252700	-1.17314000
H	-4.77064000	2.44137400	-0.95229400
C	-3.22400700	2.25001000	-2.48886300
C	-1.85180000	2.37079100	-2.77756600
C	2.09699500	5.30601800	1.63005700
H	1.98536000	5.95443300	0.74180900
H	1.36507800	5.63629900	2.38927500
C	3.52554200	5.33746800	2.15641900
H	3.73773200	6.36955300	2.49437300
H	4.21880200	5.16156800	1.30804800
C	3.83869600	4.34890000	3.30214200
H	3.50983000	3.33954100	2.98064600
C	5.35193800	4.28334300	3.55738000
H	5.91247400	3.98291100	2.64944000
H	5.59173500	3.55959500	4.35606600
H	5.74550800	5.26767400	3.86916700
C	3.06587400	4.68408200	4.58526500
H	3.35573700	5.67904100	4.96940700
H	3.27670400	3.94326200	5.37568400
H	1.97372400	4.68876500	4.42491500
C	-5.48085100	1.95645300	-3.34396100
H	-5.77894300	1.42179300	-2.42247000
H	-5.87823900	1.38156500	-4.19673800
C	-6.00112700	3.39265200	-3.37314900
H	-5.40297600	4.02192100	-2.68672600
H	-5.83233100	3.79687800	-4.38771300
C	-7.49214500	3.51792000	-2.99348300
H	-8.05720600	2.75868600	-3.57364000
C	-8.02785600	4.90288800	-3.38479500
H	-7.47958500	5.70038300	-2.84868600
H	-9.09759300	5.00233700	-3.13090700
H	-7.91767100	5.08750600	-4.46795600
C	-7.72900800	3.24831800	-1.49734500
H	-8.80192900	3.32171100	-1.24826500
H	-7.19307600	3.99233800	-0.87773500
H	-7.39329500	2.24179600	-1.18591200
C	-1.02091100	-0.55366000	-3.90450800
C	-0.17766500	-1.70531200	-3.65494900
C	1.22563800	-1.53623600	-3.81712600
H	1.90148800	-2.37755400	-3.66559100
C	-2.96145600	-2.04849400	-3.74988500
H	-4.04110800	-2.18540100	-3.85053900
C	-2.12765300	-3.15665200	-3.42745100
C	-0.73711200	-2.97772000	-3.29426000
C	-1.31771700	2.00255100	-4.16275700
H	-0.72434000	2.83375300	-4.57218300
H	-2.17385400	1.87015600	-4.84027200
C	-0.44926700	0.74736800	-4.15321600
C	1.75453000	-0.32544100	-4.21112500

H	2.82692700	-0.26136600	-4.39450000
C	0.93512000	0.82821400	-4.36389500
O	1.47744500	2.04478100	-4.68466400
C	2.79024700	2.10439700	-5.26447900
H	2.76887900	1.60547300	-6.25267800
H	3.51317800	1.55376200	-4.63442800
C	3.22392500	3.55514300	-5.39652300
H	2.50438900	4.09391000	-6.03965900
H	4.18568800	3.54873000	-5.94619000
C	3.40462700	4.33565600	-4.07653000
H	2.40601200	4.44613300	-3.61420600
C	4.31140000	3.59862600	-3.07393900
H	3.86717700	2.63649800	-2.75178300
H	4.49011600	4.22952100	-2.18076200
H	5.30210400	3.37478700	-3.51145500
C	3.94183600	5.74530400	-4.36324600
H	4.95473800	5.70178200	-4.80473500
H	4.00120400	6.34961800	-3.44018400
H	3.29022700	6.28173900	-5.07463900
C	-3.99908700	-4.70334700	-3.50289700
H	-4.20180900	-4.45498000	-4.56265000
H	-4.65872800	-4.07256900	-2.87414100
C	-4.24154600	-6.18225700	-3.24398300
H	-3.56018100	-6.76263700	-3.89320100
H	-5.26876800	-6.40840800	-3.59129000
C	-4.08360300	-6.65285600	-1.78315700
H	-3.05381700	-6.40474600	-1.45935000
C	-5.06573200	-5.95075900	-0.83269800
H	-6.11309700	-6.15849300	-1.12105100
H	-4.93577200	-4.85255600	-0.82583900
H	-4.93225400	-6.30836500	0.20404300
C	-4.24149000	-8.17821400	-1.70133900
H	-4.08771400	-8.54104600	-0.66970700
H	-3.51397100	-8.69339200	-2.35328400
H	-5.25473100	-8.48775800	-2.01868600
H	-0.45023300	-5.03038000	-2.73446800
C	-1.01506700	-0.33119000	-0.01804800
H	-0.94004400	-0.85920800	0.94149700
C	0.32168600	-0.28874600	-0.73776800
H	-1.41171600	0.67575900	0.17162300
N	-2.04819800	-1.06877200	-0.81781300
H	0.23614900	0.31471900	-1.65557700
C	1.38822900	0.29316900	0.17960800
H	0.61329300	-1.30550600	-1.04107300
H	-2.12012800	-0.73285900	-1.80294600
H	-1.82171400	-2.08689400	-0.85594300
C	2.78738200	0.29164600	-0.43214600
H	1.09898000	1.31232300	0.47413600
H	1.42411600	-0.29920800	1.10740200
C	3.81666500	0.92806600	0.51397500
H	3.10457100	-0.74717000	-0.61558100
H	2.80636900	0.80401200	-1.41348400
C	5.21712600	0.31790600	0.40292000
N	3.86466500	2.42524900	0.36684800
H	3.49587900	0.74238700	1.55546100
O	6.16149600	1.25666800	0.23214900
O	5.40022300	-0.87298600	0.51145500
C	7.57341100	0.83344700	0.24067900
C	8.12374900	0.84710700	1.65361400
H	7.62698600	-0.16474400	-0.22062100
H	8.07042800	1.56796800	-0.41187500
H	9.19678600	0.58870800	1.62651700
H	8.02741400	1.84617600	2.11324600
H	7.61455800	0.10518800	2.28990300
H	-2.97750800	-0.98499500	-0.39029700
H	3.01560400	2.94069900	0.76722700
H	4.70465800	2.79197300	0.83514300
H	3.95065900	2.68721900	-0.62625300

1 20 1.0 38 1.0
2 13 1.0 22 1.0
3 60 1.0 86 1.0
4 5 1.0 54 1.5 58 2.0
5

6 7 1.0 14 1.0 109 1.0 116 1.0
7
8 9 1.5 15 1.0 21 1.5
9 10 1.0 11 2.0
10
11 12 1.0 13 1.5
12
13 14 1.5
14 15 1.5
15 16 1.5
16 17 1.0 18 2.0
17
18 19 1.0 20 1.5
19
20 21 1.5
21 62 1.0
22 23 1.0 24 1.0 25 1.0
23
24
25 26 1.0 27 1.0 28 1.0
26
27
28 29 1.0 30 1.0 34 1.0
29
30 31 1.0 32 1.0 33 1.0
31
32
33
34 35 1.0 36 1.0 37 1.0
35
36
37
38 39 1.0 40 1.0 41 1.0
39
40
41 42 1.0 43 1.0 44 1.0
42
43
44 45 1.0 46 1.0 50 1.0
45
46 47 1.0 48 1.0 49 1.0
47
48
49
50 51 1.0 52 1.0 53 1.0
51
52
53
54 55 1.0 65 1.5
55 56 1.5 61 1.5
56 57 1.0 66 1.5
57
58 59 1.0 60 1.5
59
60 61 1.5
61 102 1.0
62 63 1.0 64 1.0 65 1.0
63
64
65 68 1.5
66 67 1.0 68 1.5
67
68 69 1.0
69 70 1.0
70 71 1.0 72 1.0 73 1.0
71
72
73 74 1.0 75 1.0 76 1.0
74
75
76 77 1.0 78 1.0 82 1.0
77
78 79 1.0 80 1.0 81 1.0
79
80
81
82 83 1.0 84 1.0 85 1.0
83
84
85
86 87 1.0 88 1.0 89 1.0
87
88
89 90 1.0 91 1.0 92 1.0
90
91
92 93 1.0 94 1.0 98 1.0
93
94 95 1.0 96 1.0 97 1.0
95
96
97
98 99 1.0 100 1.0 101 1.0
99
100
101
102 103 1.0 163 1.0 204 1.0
103
104 122 1.0 140 1.0
105 115 1.0 124 1.0
106 162 1.0 188 1.0
107 108 1.0 156 1.5 160 1.5
108
109
110 111 1.5 117 1.0 123 1.5
111 112 1.0 113 2.0
112
113 114 1.0 115 1.5
114
115 116 1.5
116 117 1.0
117 118 1.5
118 119 1.0 120 2.0
119
120 121 1.0 122 1.5
121
122 123 1.5
123 164 1.0
124 125 1.0 126 1.0 127 1.0
125
126
127 128 1.0 129 1.0 130 1.0
128
129
130 131 1.0 132 1.0 136 1.0
131
132 133 1.0 134 1.0 135 1.0
133
134
135
136 137 1.0 138 1.0 139 1.0
137
138
139
140 141 1.0 142 1.0 143 1.0
141
142
143 144 1.0 145 1.0 146 1.0
144
145
146 147 1.0 148 1.0 152 1.0
147
148 149 1.0 150 1.0 151 1.0
149
150
151
152 153 1.0 154 1.0 155 1.0
153

154
155
156 157 1.0 167 1.5
157 158 1.5 163 1.5
158 159 1.0 168 2.0
159
160 161 1.0 162 1.5
161
162 163 1.5
163
164 165 1.0 166 1.0 167 1.0
165
166
167 170 1.5
168 169 1.0 170 1.5
169
170 171 1.0
171 172 1.0
172 173 1.0 174 1.0 175 1.0
173
174
175 176 1.0 177 1.0 178 1.0
176
177
178 179 1.0 180 1.0 184 1.0
179
180 181 1.0 182 1.0 183 1.0
181
182
183
184 185 1.0 186 1.0 187 1.0
185
186
187
188 189 1.0 190 1.0 191 1.0
189
190
191 192 1.0 193 1.0 194 1.0
192
193
194 195 1.0 196 1.0 200 1.0
195
196 197 1.0 198 1.0 199 1.0
197
198
199
200 201 1.0 202 1.0 203 1.0
201
202
203
204
205 206 1.0 207 1.0 208 1.0 209 1.0
206
207 210 1.0 211 1.0 212 1.0
208
209 213 1.0 214 1.0 233 1.0
210
211 215 1.0 216 1.0 217 1.0
212
213
214
215 218 1.0 219 1.0 220 1.0
216
217
218 221 1.0 222 1.0 223 1.0
219
220
221 224 1.5 225 2.0
222 235 1.0 236 1.0
223
224 226 1.0
225
226 227 1.0 228 1.0 229 1.0
227 230 1.0 231 1.0 232 1.0
228
229
230
231
232
233
234
235
236

0 imaginary frequency
Energy: -4440.9084 Hartree

Cartesian coordinates of (*S*)-**8**$^+$ @ *pR*-**PrS[4]**^{iPe}

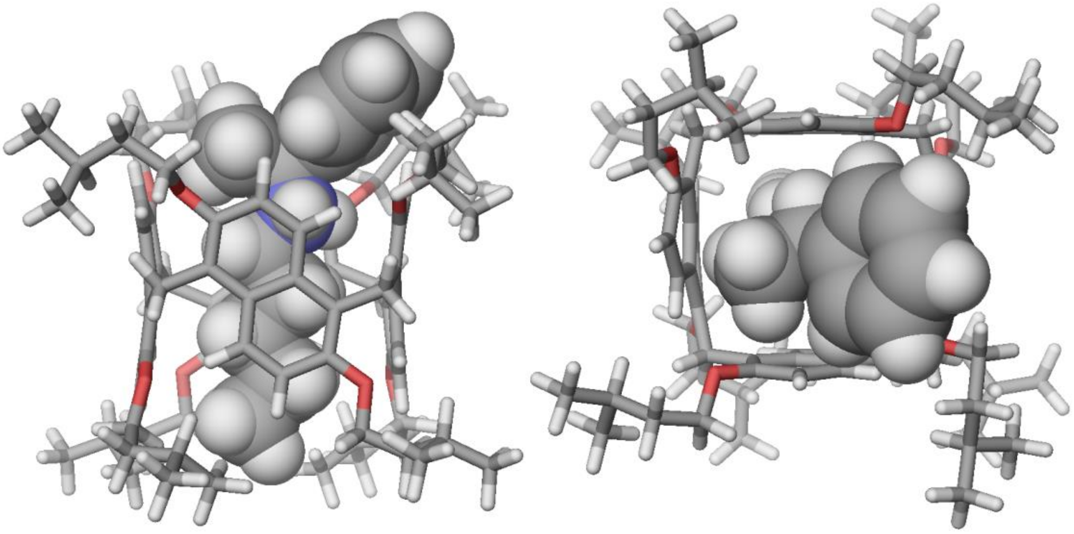

Figure S65: Side and top view of DFT-optimized structure (B97D3/SVP/SVPFIT) of the (*S*)-**8**$^+$ @ *pS*-**PrS[4]**^{iPe} complex.

C 1.53502500 0.26144500 3.57596500
C 0.48677300 0.85280700 4.30001500

C	0.00230000	2.14205800	3.94519300
C	0.51230700	2.81532900	2.85235300
C	1.60895000	2.29862900	2.11489400
C	2.18195600	1.04529600	2.55852100
C	2.14462300	2.99565300	0.97223000
C	3.36963900	2.56280800	0.45632100
C	4.01584000	1.40953800	0.98069700
C	3.40782900	0.63228400	1.95314500
O	3.91633300	3.21762100	-0.62243100
O	-0.05546700	0.13902600	5.33067900
C	5.22860200	3.78068900	-0.45322900
C	5.53674900	4.65705500	-1.65775100
C	4.53793200	5.80001600	-1.93267700
C	4.37325500	6.74135500	-0.73097300
C	4.95800100	6.57510500	-3.18992400
C	-1.00698100	0.76216700	6.20351300
C	-1.40745900	-0.22466800	7.28981000
C	-2.20774000	-1.46127100	6.83206600
C	-2.46889100	-2.38978900	8.02701800
C	-3.52410800	-1.08062500	6.13630400
C	1.95278000	-1.18068400	3.85563900
H	-0.81464700	2.59742000	4.50918100
H	0.07376900	3.77181200	2.57074700
H	4.97815500	1.10438400	0.56454300
H	3.89335000	-0.29217100	2.26990800
H	5.24743300	4.36138600	0.48933100
H	5.97976400	2.97161700	-0.36697000
H	6.54974300	5.08022900	-1.50617800
H	5.60099900	4.01338700	-2.55610600
H	3.55364600	5.33459200	-2.13822200
H	5.34139500	7.19774100	-0.44969900
H	3.97506100	6.22004500	0.15728200
H	3.67489300	7.56294300	-0.97013700
H	5.93575700	7.07094300	-3.04013700
H	4.22113000	7.35848400	-3.44152400
H	5.05281800	5.90615500	-4.06486900
H	-0.54715100	1.66382700	6.65421300
H	-1.89006900	1.08980000	5.62123700
H	-0.49163500	-0.55183500	7.81684000
H	-2.00848100	0.33836700	8.03133300
H	-1.58412500	-2.01798300	6.10708200
H	-3.09708800	-1.88846400	8.78748400
H	-1.52551900	-2.69060200	8.51703700
H	-2.99412700	-3.30890000	7.71130800
H	-4.16390200	-0.47381400	6.80473600
H	-4.09908600	-1.98399700	5.86382000
H	-3.36096700	-0.50148400	5.20988900
C	-2.89267000	-0.32241100	-2.90658600
C	-4.08706300	-0.18729800	-2.18513300
C	-4.48104900	-1.18417400	-1.25016700
C	-3.67132200	-2.27197000	-0.98710100
C	-2.48454800	-2.50622900	-1.73481900
C	-2.16354800	-1.55680500	-2.77891200
C	-1.65384200	-3.65745900	-1.50162500
C	-0.68631300	-3.98030400	-2.46538300
C	-0.48447900	-3.13417200	-3.59084100
C	-1.14848300	-1.92500400	-3.70281300
O	0.05566600	-5.11631200	-2.27085700
O	-4.82977500	0.94534300	-2.38836700
C	0.91385900	-5.58837600	-3.31318800
C	1.64553500	-6.83245500	-2.83219100
C	2.62794200	-6.63443200	-1.65908300
C	3.28174300	-7.97371700	-1.28896300
C	3.69273700	-5.56760700	-1.95907000
C	-6.14843200	1.03985100	-1.83783600
C	-6.76994600	2.35661000	-2.27995100
C	-6.08908400	3.63905100	-1.75913400
C	-6.76164000	4.87785800	-2.36834700
C	-6.08510900	3.71765400	-0.22473300
C	-2.38315900	0.80955900	-3.79633100
H	-5.40860900	-1.07258900	-0.68836200
H	-3.96903000	-2.96872500	-0.20389700
H	0.23145000	-3.41110900	-4.36751600
H	-0.89077300	-1.26240700	-4.52995800

H	0.30567900	-5.81931300	-4.21060600
H	1.63664900	-4.79833100	-3.59624600
H	0.89640300	-7.59854400	-2.55795800
H	2.20031400	-7.23629600	-3.70243500
H	2.04214600	-6.29568900	-0.78344700
H	3.94013500	-7.86779100	-0.40813900
H	3.89735500	-8.35761600	-2.12436200
H	2.52202800	-8.74022700	-1.05299800
H	4.25928700	-5.81735200	-2.87619000
H	4.42265100	-5.49561800	-1.13260900
H	3.25217100	-4.56443800	-2.09888200
H	-6.75580400	0.18588900	-2.19822700
H	-6.10289200	0.97314000	-0.73335900
H	-6.79105000	2.37622100	-3.38572200
H	-7.82702600	2.34676600	-1.94759700
H	-5.03764900	3.62481600	-2.10459600
H	-7.82051100	4.94722100	-2.05481000
H	-6.74074300	4.84551200	-3.47248700
H	-6.25572100	5.80605000	-2.04739300
H	-7.11630300	3.69414100	0.17571200
H	-5.62051700	4.65988300	0.11836500
H	-5.52777300	2.88471400	0.23895300
C	0.09564200	3.70199800	-0.38523300
C	-1.14798100	4.05079300	0.16551600
C	-2.35353300	3.53054700	-0.37602900
C	-2.33450200	2.67305700	-1.45847200
C	-1.11984900	2.34005500	-2.11530600
C	0.10625700	2.90965800	-1.58569200
C	-1.11345400	1.49881300	-3.28856900
C	0.09717000	1.33984400	-3.96996800
C	1.29181300	1.94366500	-3.48994900
C	1.31287800	2.67674800	-2.31541700
O	0.16902900	0.56205800	-5.10376100
O	-1.14831100	4.89205900	1.23720200
C	0.31779900	1.27097300	-6.34912200
C	0.29507600	0.26553900	-7.48972600
C	-1.00736000	-0.54583800	-7.64997200
C	-2.22354700	0.34601000	-7.93930900
C	-0.83407700	-1.61000600	-8.74346500
C	-2.36432800	5.17212200	1.94005400
C	-2.02928100	6.03373200	3.14968200
C	-1.28989700	7.35307900	2.84680900
C	-2.06121100	8.24496400	1.86322200
C	-0.98935200	8.09777300	4.15546900
C	1.38739700	4.14303000	0.30444500
H	-3.31293000	3.79025400	0.07191300
H	-3.28009800	2.26933100	-1.81900000
H	2.20851700	1.81821300	-4.07700600
H	2.25592900	3.11262300	-1.97783800
H	-0.49934600	2.01413700	-6.43743500
H	1.27487700	1.83151800	-6.34852200
H	0.49718600	0.82169900	-8.42667200
H	1.14467100	-0.43084100	-7.35201100
H	-1.19165100	-1.07491500	-6.69468100
H	-2.08194300	0.91411500	-8.87832800
H	-2.41298300	1.07502900	-7.13168700
H	-3.13828400	-0.26255600	-8.05182600
H	-0.64243600	-1.13903900	-9.72622500
H	-1.74107900	-2.23256200	-8.84235500
H	0.01582400	-2.28157000	-8.52301900
H	-3.07238400	5.68873700	1.26336300
H	-2.83596100	4.22118400	2.25989000
H	-2.98440400	6.25630500	3.66586400
H	-1.42605500	5.43434700	3.85879300
H	-0.32357700	7.08616000	2.37562900
H	-3.07039200	8.48376700	2.25034300
H	-2.17830800	7.76843500	0.87388900
H	-1.53182600	9.20109700	1.70441900
H	-1.92554500	8.39669400	4.66423500
H	-0.40411100	9.01527100	3.96706700
H	-0.41312100	7.46711300	4.85696300
C	-1.44126300	-3.78866200	1.04493300
C	-2.43239700	-3.45732300	1.98369200
C	-2.11295400	-2.66917100	3.12107800

C	-0.82713400	-2.20732100	3.33275700
C	0.24012500	-2.57763600	2.47371900
C	-0.08194600	-3.42351500	1.33936900
C	1.60181900	-2.17488000	2.74465200
C	2.61100000	-2.74508100	1.96655600
C	2.30805600	-3.62778000	0.89466000
C	1.00196200	-3.92471600	0.55744700
O	3.94424600	-2.45339100	2.18640400
O	-3.70268100	-3.90001700	1.75012700
C	4.66512500	-3.38963800	3.00949000
C	6.15639200	-3.10639100	2.89812600
C	6.59893700	-1.66283100	3.21471500
C	6.15859300	-1.19780700	4.60967300
C	8.12038500	-1.53165100	3.05113900
C	-4.71115000	-3.74116600	2.75547200
C	-5.99358900	-4.40262400	2.27206900
C	-6.65985800	-3.77600400	1.03001300
C	-7.87043200	-4.61807300	0.60152500
C	-7.06477100	-2.31176800	1.25866900
C	-1.81599200	-4.51430600	-0.24686700
H	3.13991600	-4.06568500	0.33543300
H	0.80557700	-4.57188400	-0.29819200
H	4.30701300	-3.30066000	4.05503100
H	4.44198100	-4.42173900	2.67284800
H	6.67478600	-3.80797800	3.58159300
H	6.49090300	-3.36881300	1.87577300
H	6.11963500	-1.00066100	2.46772300
H	6.56656900	-1.86016800	5.39647600
H	5.06049300	-1.18283300	4.71826800
H	6.51990600	-0.17435000	4.81376500
H	8.65235600	-2.14817000	3.79982900
H	8.44840300	-0.48542100	3.18482100
H	8.45526100	-1.86707000	2.05230300
H	-4.36525100	-4.21409500	3.69613400
H	-4.87007000	-2.66470600	2.96156600
H	-5.77836300	-5.46939000	2.07402300
H	-6.70997400	-4.38062500	3.11731300
H	-5.92401400	-3.80311200	0.20366900
H	-8.64393100	-4.62895100	1.39271700
H	-7.58195100	-5.66539000	0.40031100
H	-8.33455500	-4.21278300	-0.31528500
H	-7.78116700	-2.22583600	2.09760200
H	-7.55644900	-1.89668100	0.36043600
H	-6.19931800	-1.66666300	1.48984400
H	-1.20205200	-5.41992100	-0.36380800
H	-2.85836100	-4.85555900	-0.15820600
H	2.06178800	4.61634800	-0.42324600
H	1.13686700	4.91252200	1.04951700
H	3.04084700	-1.22067500	4.00998600
H	1.48746900	-1.50011100	4.79997900
H	-2.17306600	0.42358300	-4.80452800
H	-3.18620300	1.55453100	-3.90229000
C	0.50773500	-0.61877800	-0.75284300
H	0.49233300	-1.70594300	-0.58909300
H	0.02488900	-0.40519500	-1.71582800
N	1.95764800	-0.20681900	-0.88606600
C	2.88954800	-1.18345300	-1.59395400
H	2.80365900	-2.10057100	-0.99170100
C	2.41917300	-1.46388900	-3.01341100
C	-0.14010300	0.13433400	0.39885400
H	0.03586500	1.21190200	0.26862400
H	0.35993900	-0.15826600	1.33306500
C	-1.63834000	-0.11286200	0.53812600
H	-1.82682500	-1.19018100	0.65760300
H	-2.14659700	0.18957600	-0.39078300
C	-2.24808400	0.64260000	1.72127500
H	-2.01127900	1.71766200	1.62857300
H	-1.76093600	0.30751000	2.65368800
C	-3.76018800	0.45447200	1.83287800
H	-4.17694300	0.98513200	2.70868500
H	-4.01871200	-0.61481500	1.92806300
H	-4.27377700	0.83112000	0.93066900
H	2.35953200	-0.03398600	0.05598400
H	1.98534900	0.71467100	-1.36100400
H	-2.89292000	-2.39076600	3.82916600
H	-0.63603800	-1.56892500	4.19477900
H	2.40646900	-0.56005800	-3.64046300
H	3.11034800	-2.19027400	-3.47114000
H	1.40988100	-1.89407500	-3.02048500
C	6.99079700	0.20092100	-1.36914500
C	6.07809500	0.85277900	-2.21290900
C	4.74963700	0.41529400	-2.28399300
C	4.31763300	-0.68077600	-1.51340900
C	5.23689200	-1.32898200	-0.66882500
C	6.56642500	-0.89055600	-0.59669200
H	8.03072900	0.54266200	-1.31537800
H	6.40276800	1.70502300	-2.81942500
H	4.04847500	0.93858900	-2.94102400
H	4.90215800	-2.15382000	-0.03279000
H	7.27135600	-1.40024600	0.06783400

1 2 1.5 6 1.5 23 1.0
2 3 1.5 12 1.0
3 4 2.0 24 1.0
4 5 1.5 25 1.0
5 6 1.0 7 1.5
6 10 1.5
7 8 1.5 121 1.0
8 9 1.5 11 1.0
9 10 2.0 26 1.0
10 27 1.0
11 13 1.0
12 18 1.0
13 14 1.0 28 1.0 29 1.0
14 15 1.0 30 1.0 31 1.0
15 16 1.0 17 1.0 32 1.0
16 33 1.0 34 1.0 35 1.0
17 36 1.0 37 1.0 38 1.0
18 19 1.0 39 1.0 40 1.0
19 20 1.0 41 1.0 42 1.0
20 21 1.0 22 1.0 43 1.0
21 44 1.0 45 1.0 46 1.0
22 47 1.0 48 1.0 49 1.0
23 154 1.0 199 1.0 200 1.0
24
25
26
27
28
29
30
31
32
33
34
35
36
37
38
39
40
41
42
43
44
45
46
47
48
49
50 51 1.5 55 1.5 72 1.0
51 52 1.5 61 1.0
52 53 2.0 73 1.0
53 54 1.5 74 1.0
54 55 1.5 56 1.5
55 59 1.5
56 57 1.5 170 1.0
57 58 1.5 60 1.0

58 59 2.0 75 1.0
59 76 1.0
60 62 1.0
61 67 1.0
62 63 1.0 77 1.0 78 1.0
63 64 1.0 79 1.0 80 1.0
64 65 1.0 66 1.0 81 1.0
65 82 1.0 83 1.0 84 1.0
66 85 1.0 86 1.0 87 1.0
67 68 1.0 88 1.0 89 1.0
68 69 1.0 90 1.0 91 1.0
69 70 1.0 71 1.0 92 1.0
70 93 1.0 94 1.0 95 1.0
71 96 1.0 97 1.0 98 1.0
72 105 1.0 201 1.0 202 1.0
73
74
75
76
77
78
79
80
81
82
83
84
85
86
87
88
89
90
91
92
93
94
95
96
97
98
99 100 1.5 104 1.5 121 1.0
100 101 1.5 110 1.0
101 102 2.0 122 1.0
102 103 1.5 123 1.0
103 104 1.0 105 1.5
104 108 1.5
105 106 1.5
106 107 1.5 109 1.0
107 108 2.0 124 1.0
108 125 1.0
109 111 1.0
110 116 1.0
111 112 1.0 126 1.0 127 1.0
112 113 1.0 128 1.0 129 1.0
113 114 1.0 115 1.0 130 1.0
114 131 1.0 132 1.0 133 1.0
115 134 1.0 135 1.0 136 1.0
116 117 1.0 137 1.0 138 1.0
117 118 1.0 139 1.0 140 1.0
118 119 1.0 120 1.0 141 1.0
119 142 1.0 143 1.0 144 1.0
120 145 1.0 146 1.0 147 1.0
121 197 1.0 198 1.0
122
123
124
125
126
127
128
129
130
131
132
133
134
135
136
137
138
139
140
141
142
143
144
145
146
147
148 149 1.5 153 1.5 170 1.0
149 150 1.5 159 1.0
150 151 2.0 225 1.0
151 152 1.5 226 1.0
152 153 1.0 154 1.5
153 157 1.5
154 155 1.5
155 156 1.5 158 1.0
156 157 2.0 171 1.0
157 172 1.0
158 160 1.0
159 165 1.0
160 161 1.0 173 1.0 174 1.0
161 162 1.0 175 1.0 176 1.0
162 163 1.0 164 1.0 177 1.0
163 178 1.0 179 1.0 180 1.0
164 181 1.0 182 1.0 183 1.0
165 166 1.0 184 1.0 185 1.0
166 167 1.0 186 1.0 187 1.0
167 168 1.0 169 1.0 188 1.0
168 189 1.0 190 1.0 191 1.0
169 192 1.0 193 1.0 194 1.0
170 195 1.0 196 1.0
171
172
173
174
175
176
177
178
179
180
181
182
183
184
185
186
187
188
189
190
191
192
193
194
195
196
197
198
199
200
201
202
203 204 1.0 205 1.0 206 1.0 210 1.0
204
205

206 207 1.0 223 1.0 224 1.0
207 208 1.0 209 1.0 233 1.0
208
209 227 1.0 228 1.0 229 1.0
210 211 1.0 212 1.0 213 1.0
211
212
213 214 1.0 215 1.0 216 1.0
214
215
216 217 1.0 218 1.0 219 1.0
217
218
219 220 1.0 221 1.0 222 1.0
220
221
222
223
224
225
226
227
228
229
230 231 1.5 235 1.5 236 1.0
231 232 1.5 237 1.0
232 233 1.5 238 1.0
233 234 1.5
234 235 1.5 239 1.0
235 240 1.0
236
237
238
239
240

0 imaginary frequency
Energy: -4427.7133 Hartree

Cartesian coordinates of (*S*)-**8**$^+$ @ *pS*-**PrS[4]**^{iPe}

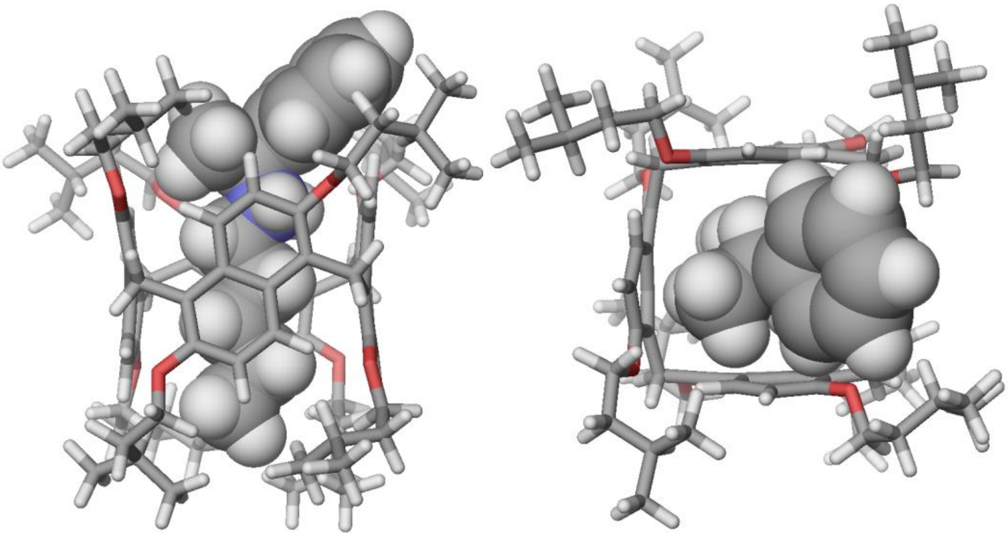

Figure S66: Side and top view of DFT-optimized structure (B97D3/SVP/SVPFIT) of the (*S*)-**8**$^+$ @ *pS*-**PrS[4]**^{iPe} complex.

C	1.36238700	-2.83634800	-2.41672900
C	0.21904200	-3.06932200	-3.19744300
C	-0.30781800	-2.04273900	-4.02737100
C	0.25353600	-0.78230400	-4.04365400
C	1.44296400	-0.49917100	-3.32456800
C	2.05228000	-1.58371100	-2.58331500
C	2.03055100	0.81597500	-3.33837800
C	3.31752200	0.96197500	-2.80903900
C	3.98913600	-0.13983700	-2.20824200
C	3.35455700	-1.35953700	-2.04707400
O	3.91383600	2.19985400	-2.82237300
O	-0.38014500	-4.29555800	-3.11347900
C	5.17013500	2.32071800	-3.51049400
C	5.53625800	3.79520800	-3.58728100
C	4.50243800	4.71124500	-4.27401000
C	4.18358700	4.26789500	-5.70888300
C	4.98527500	6.16845100	-4.23902000
C	-1.41768900	-4.63502400	-4.04469700
C	-1.91334200	-6.04323100	-3.75621700
C	-2.66538100	-6.24400800	-2.42457000
C	-3.07657500	-7.71546300	-2.27002200
C	-3.88453400	-5.31660400	-2.29581600
C	1.83428100	-3.87519900	-1.40108600
H	-1.20190700	-2.22833600	-4.62399200
H	-0.21672600	-0.00137300	-4.64032100
H	5.00206400	0.00140300	-1.82547700
H	3.86975500	-2.15354000	-1.50474000
H	5.06891000	1.87182300	-4.51772500
H	5.95577200	1.76003200	-2.96759800
H	6.50516900	3.86671300	-4.12057300
H	5.71892200	4.16724600	-2.56087800
H	3.56716300	4.65250000	-3.68302800
H	5.09722100	4.26034900	-6.33335700
H	3.74086200	3.25701800	-5.74582300
H	3.46303000	4.95892800	-6.18124400
H	5.91808100	6.29000400	-4.82168100
H	4.23068400	6.84997500	-4.67037800
H	5.19076100	6.50191000	-3.20525900
H	-1.01111500	-4.57080900	-5.07344200
H	-2.24690900	-3.90638100	-3.96720000
H	-1.05273600	-6.73648000	-3.80354900
H	-2.58457900	-6.32326900	-4.59240400
H	-1.96415200	-6.00627600	-1.60233800
H	-3.79197500	-8.01117100	-3.06057300
H	-2.20250600	-8.38742000	-2.34070200
H	-3.56250500	-7.89431400	-1.29415000
H	-4.58502100	-5.46328500	-3.13976400
H	-4.44318300	-5.52931100	-1.36641800
H	-3.60307300	-4.24890200	-2.27391500
C	-2.50858700	2.45422200	1.95717700

C	-3.77222500	1.97989000	1.57858400
C	-4.18825300	0.66720100	1.93063600
C	-3.34370400	-0.18176000	2.61975400
C	-2.08615700	0.26695200	3.10894100
C	-1.71704400	1.64039800	2.84061000
C	-1.22366700	-0.59067700	3.87645800
C	-0.16899100	-0.00127500	4.58752900
C	0.11585200	1.38516600	4.43752400
C	-0.58865200	2.16195800	3.53369100
O	0.58256300	-0.81882900	5.38900200
O	-4.56181900	2.81480000	0.83616100
C	1.53166600	-0.26481900	6.30378600
C	2.07125300	-1.39111500	7.17556200
C	2.80673200	-2.52772600	6.43649100
C	3.17019000	-3.64982400	7.41974400
C	4.05280900	-2.03086300	5.68928800
C	-5.93568400	2.48810500	0.60793600
C	-6.61162300	3.68578000	-0.04498200
C	-6.01951000	4.13905500	-1.39442100
C	-6.73791700	5.40161400	-1.89163200
C	-6.06254400	3.02922600	-2.45309500
C	-2.00285100	3.79693500	1.43850000
H	-5.16518200	0.29900700	1.61558100
H	-3.66603200	-1.20617400	2.80515500
H	0.92391500	1.84627900	5.01054800
H	-0.28069300	3.19780600	3.38298000
H	1.03634400	0.50134900	6.93236400
H	2.34675700	0.23705500	5.74509700
H	1.22609500	-1.81786700	7.74732300
H	2.75416500	-0.93509700	7.91955200
H	2.10484800	-2.94831800	5.69054300
H	3.65934000	-4.49342200	6.90059800
H	3.86814700	-3.28395700	8.19644700
H	2.27367300	-4.04185400	7.93262700
H	4.78554100	-1.58820300	6.39022500
H	4.55580000	-2.86330400	5.16507700
H	3.80862600	-1.26241900	4.93564200
H	-6.42501600	2.25170300	1.57350800
H	-6.00668200	1.58559100	-0.03146800
H	-6.58171700	4.53194400	0.66687000
H	-7.68068400	3.42733900	-0.18054000
H	-4.95789400	4.40090900	-1.21986600
H	-7.80826000	5.19752300	-2.08441800
H	-6.68113300	6.21683200	-1.14804400
H	-6.29238400	5.77049300	-2.83285800
H	-7.10206500	2.70922700	-2.65608200
H	-5.63291600	3.38300800	-3.40688300
H	-5.49355900	2.13492000	-2.14540600
C	0.05128900	2.40657500	-3.03944600
C	-1.24416000	2.21404600	-3.54757300
C	-2.37752500	2.40290300	-2.71058800
C	-2.23878800	2.82966100	-1.40284100
C	-0.96459900	3.15748000	-0.86349800
C	0.19092700	2.96714400	-1.72102500
C	-0.82393500	3.69557800	0.46960000
C	0.44007300	4.14571800	0.86208800
C	1.56488300	4.01261500	0.00472100
C	1.46046500	3.40132800	-1.23132100
O	0.63690300	4.68800200	2.11364800
O	-1.35461100	1.85582700	-4.86262000
C	0.78532300	6.11937100	2.16199200
C	0.90390200	6.54880300	3.61605100
C	-0.32045500	6.25465900	4.50770300
C	-1.58022900	6.99693600	4.03804300
C	-0.00030100	6.59016200	5.97166400
C	-2.62946800	1.54614500	-5.44464300
C	-3.02346900	0.09223600	-5.18767100
C	-4.40524800	-0.33286200	-5.71843800
C	-5.55610000	0.37761800	-4.99010300
C	-4.55112500	-1.85945200	-5.60922100
C	1.27071200	2.02622700	-3.87943700
H	-3.37989400	2.20112800	-3.08973600
H	-3.13584200	2.93851200	-0.79289500
H	2.52738400	4.40436200	0.35307100

H	2.35622000	3.29404600	-1.84556400
H	-0.08592300	6.58833900	1.66268900
H	1.69037900	6.41973300	1.59509400
H	1.11232200	7.63710100	3.62739500
H	1.79668900	6.05611600	4.04718300
H	-0.52268400	5.16722600	4.44982700
H	-1.42521200	8.09230700	4.06447100
H	-1.87053100	6.72448400	3.00798700
H	-2.43931400	6.76517300	4.69237700
H	0.21796500	7.66835500	6.09119400
H	-0.85078700	6.34614400	6.63293100
H	0.88127000	6.02868700	6.33156100
H	-2.49389600	1.72487800	-6.52508200
H	-3.39445500	2.25929600	-5.09011300
H	-2.97816200	-0.10834000	-4.10199200
H	-2.25199700	-0.54543100	-5.65497700
H	-4.45830700	-0.06290900	-6.79365800
H	-5.52516500	0.16049700	-3.90611000
H	-5.51889700	1.47330300	-5.11326000
H	-6.53521600	0.03867000	-5.37215400
H	-4.48405400	-2.18299000	-4.55267300
H	-5.52541300	-2.20025000	-6.00181900
H	-3.75727400	-2.38320700	-6.17261900
C	-1.19175900	-2.79923900	2.58826700
C	-2.26609200	-3.37649800	1.89064300
C	-2.07423600	-3.92133000	0.59357300
C	-0.83325300	-3.90106700	-0.01443500
C	0.31268900	-3.41100400	0.66430300
C	0.12033400	-2.88952000	2.00587800
C	1.62535700	-3.46775800	0.06062000
C	2.72096500	-3.13998400	0.86448900
C	2.54418400	-2.68503000	2.20100200
C	1.28443600	-2.51764400	2.74394100
O	4.01676400	-3.21358800	0.39055400
O	-3.48944100	-3.37432000	2.49416100
C	4.72503600	-4.43365700	0.68388500
C	6.20141000	-4.23675600	0.37268200
C	6.53438500	-3.75380300	-1.05413000
C	5.98285900	-4.68704900	-2.14080400
C	8.05143500	-3.57111200	-1.20821800
C	-4.58119400	-4.08941800	1.90155600
C	-5.78394800	-4.00515800	2.82989200
C	-6.38535100	-2.60111300	3.04492100
C	-7.50963600	-2.66498700	4.08897700
C	-6.88828900	-1.97333100	1.73610200
C	-1.43712200	-2.10202300	3.92625600
H	3.43801700	-2.47645800	2.79715500
H	1.19008200	-2.13255500	3.76016600
H	4.28429400	-5.25753800	0.08727300
H	4.58577600	-4.69025300	1.75304200
H	6.70908200	-5.20333200	0.56215200
H	6.62170200	-3.51948500	1.10393400
H	6.06003300	-2.76179100	-1.18431900
H	6.38864900	-5.71031000	-2.02920500
H	4.88175600	-4.75687800	-2.11273700
H	6.26056700	-4.32483900	-3.14639500
H	8.57792100	-4.53896500	-1.10986000
H	8.30459400	-3.15130900	-2.19801600
H	8.46249300	-2.89292000	-0.43784600
H	-4.28512000	-5.14665600	1.75150000
H	-4.81244500	-3.66203500	0.90636900
H	-5.49594200	-4.43495900	3.80756900
H	-6.56383100	-4.67321800	2.41326500
H	-5.58700700	-1.95159200	3.45218100
H	-8.34046700	-3.30499100	3.73613900
H	-7.14792500	-3.08236900	5.04581300
H	-7.92312900	-1.66112300	4.29253400
H	-7.67184900	-2.60163200	1.27166700
H	-7.33053200	-0.97832100	1.92360900
H	-6.08111400	-1.84179400	0.99465800
H	-0.77263300	-2.52096700	4.69665600
H	-2.46513000	-2.32868500	4.24612500
H	1.97209200	2.87099600	-3.93440900
H	0.93451100	1.82902900	-4.90807600

H	2.90698800	-4.06913800	-1.54602000
H	1.31063000	-4.82170500	-1.60200200
H	-1.68735700	4.42555400	2.28379300
H	-2.84017900	4.31708300	0.94974800
C	0.71466200	0.39838900	0.75446000
H	0.51810700	-0.12543700	1.70045600
H	0.48605200	1.46408500	0.89124900
N	2.19887500	0.29380200	0.48648800
C	3.12345400	1.16150700	1.33346800
H	2.73641400	2.17889500	1.17429700
C	3.02112700	0.79987600	2.80700900
C	-0.05090100	-0.20880900	-0.41112600
H	0.11415800	0.41480200	-1.30231000
H	0.36769600	-1.19996600	-0.63712400
C	-1.54827700	-0.35342300	-0.16369500
H	-1.71408000	-1.01259600	0.70093900
H	-1.97511000	0.62529100	0.10485500
C	-2.28515400	-0.91346200	-1.38077900
H	-2.06832100	-0.27368500	-2.25355900
H	-1.87399700	-1.90773400	-1.62895200
C	-3.79535700	-1.01607700	-1.17576400
H	-4.29848500	-1.45705900	-2.05534100
H	-4.03335900	-1.64365500	-0.29905500
H	-4.24018700	-0.02157500	-0.99330200
H	2.47575700	-0.69982500	0.56793100
H	2.37951600	0.53393700	-0.50387400
H	-2.91952800	-4.33757000	0.04642900
H	-0.74171700	-4.29237100	-1.02717600
H	3.39907500	-0.21342700	3.00167500
H	1.98631200	0.86912400	3.16576100
H	3.63439800	1.51140900	3.38307000
C	7.19437500	0.95679600	-0.14065400
C	6.46430500	2.14948300	-0.24188000
C	5.14075700	2.20453500	0.21818300
C	4.53947100	1.07415400	0.79850600
C	5.27980500	-0.11983700	0.90057700
C	6.59608200	-0.17923500	0.42767200
H	8.22769900	0.91079000	-0.50289000
H	6.92434700	3.04033100	-0.68295500
H	4.56917900	3.13281200	0.12004900
H	4.82863500	-1.02711900	1.31303600
H	7.15754900	-1.11537900	0.50366400

1 2 1.5 6 1.5 23 1.0
2 3 1.5 12 1.0
3 4 2.0 24 1.0
4 5 1.5 25 1.0
5 6 1.0 7 1.5
6 10 1.5
7 8 1.5 121 1.0
8 9 1.5 11 1.0
9 10 2.0 26 1.0
10 27 1.0
11 13 1.0
12 18 1.0
13 14 1.0 28 1.0 29 1.0
14 15 1.0 30 1.0 31 1.0
15 16 1.0 17 1.0 32 1.0
16 33 1.0 34 1.0 35 1.0
17 36 1.0 37 1.0 38 1.0
18 19 1.0 39 1.0 40 1.0
19 20 1.0 41 1.0 42 1.0
20 21 1.0 22 1.0 43 1.0
21 44 1.0 45 1.0 46 1.0
22 47 1.0 48 1.0 49 1.0
23 154 1.0 199 1.0 200 1.0
24
25
26
27
28
29
30
31
32
33
34
35
36
37
38
39
40
41
42
43
44
45
46
47
48
49
50 51 1.5 55 1.5 72 1.0
51 52 1.5 61 1.0
52 53 2.0 73 1.0
53 54 1.5 74 1.0
54 55 1.5 56 1.5
55 59 1.5
56 57 1.5 170 1.0
57 58 1.5 60 1.0
58 59 2.0 75 1.0
59 76 1.0
60 62 1.0
61 67 1.0
62 63 1.0 77 1.0 78 1.0
63 64 1.0 79 1.0 80 1.0
64 65 1.0 66 1.0 81 1.0
65 82 1.0 83 1.0 84 1.0
66 85 1.0 86 1.0 87 1.0
67 68 1.0 88 1.0 89 1.0
68 69 1.0 90 1.0 91 1.0
69 70 1.0 71 1.0 92 1.0
70 93 1.0 94 1.0 95 1.0
71 96 1.0 97 1.0 98 1.0
72 105 1.0 201 1.0 202 1.0
73
74
75
76
77
78
79
80
81
82
83
84
85
86
87
88
89
90
91
92
93
94
95
96
97
98
99 100 1.5 104 1.5 121 1.0
100 101 1.5 110 1.0
101 102 2.0 122 1.0
102 103 1.5 123 1.0
103 104 1.0 105 1.5
104 108 1.5
105 106 1.5

106 107 1.5 109 1.0
107 108 2.0 124 1.0
108 125 1.0
109 111 1.0
110 116 1.0
111 112 1.0 126 1.0 127 1.0
112 113 1.0 128 1.0 129 1.0
113 114 1.0 115 1.0 130 1.0
114 131 1.0 132 1.0 133 1.0
115 134 1.0 135 1.0 136 1.0
116 117 1.0 137 1.0 138 1.0
117 118 1.0 139 1.0 140 1.0
118 119 1.0 120 1.0 141 1.0
119 142 1.0 143 1.0 144 1.0
120 145 1.0 146 1.0 147 1.0
121 197 1.0 198 1.0
122
123
124
125
126
127
128
129
130
131
132
133
134
135
136
137
138
139
140
141
142
143
144
145
146
147
148 149 1.5 153 1.5 170 1.0
149 150 1.5 159 1.0
150 151 2.0 225 1.0
151 152 1.5 226 1.0
152 153 1.0 154 1.5
153 157 1.5
154 155 1.5
155 156 1.5 158 1.0
156 157 2.0 171 1.0
157 172 1.0
158 160 1.0
159 165 1.0
160 161 1.0 173 1.0 174 1.0
161 162 1.0 175 1.0 176 1.0
162 163 1.0 164 1.0 177 1.0
163 178 1.0 179 1.0 180 1.0
164 181 1.0 182 1.0 183 1.0
165 166 1.0 184 1.0 185 1.0
166 167 1.0 186 1.0 187 1.0
167 168 1.0 169 1.0 188 1.0
168 189 1.0 190 1.0 191 1.0
169 192 1.0 193 1.0 194 1.0
170 195 1.0 196 1.0
171
172
173
174
175
176
177
178
179
180
181
182
183
184
185
186
187
188
189
190
191
192
193
194
195
196
197
198
199
200
201
202
203 204 1.0 205 1.0 206 1.0 210 1.0
204
205
206 207 1.0 223 1.0 224 1.0
207 208 1.0 209 1.0 233 1.0
208
209 227 1.0 228 1.0 229 1.0
210 211 1.0 212 1.0 213 1.0
211
212
213 214 1.0 215 1.0 216 1.0
214
215
216 217 1.0 218 1.0 219 1.0
217
218
219 220 1.0 221 1.0 222 1.0
220
221
222
223
224
225
226
227
228
229
230 231 1.5 235 1.5 236 1.0
231 232 1.5 237 1.0
232 233 1.5 238 1.0
233 234 1.5
234 235 1.5 239 1.0
235 240 1.0
236
237
238
239
240

---

0 imaginary frequency
Energy: -4427.7141 Hartree

NBO and NCI analysis

Natural bond orbital (NBO)[4] studies were performed with NBO 3.1 version implemented in Gaussian 16[2] and second-order perturbation theory analysis was performed on optimized structures using the B97D3/SVP/SVPFIT level of theory. The non-covalent interaction (NCI) investigations were carried out with the Multiwfn program[5] and its plot was graphed with ChemCraft program. Plots describe the RDG values versus the electron density multiplied by the sign of the second Hessian eigenvalue (s = 0.5 a.u.; left) and gradient isosurfaces (s = 0.4 a.u.; right) for the complexes. The coloring scheme was chosen to assist in distinguishing the amplitude of the electron density corresponding to different types of interactions. Marked in green represent medium-strong (cation···π, Van der Waals and C-H···π) interactions whereas the red color represents the repulsive ones.

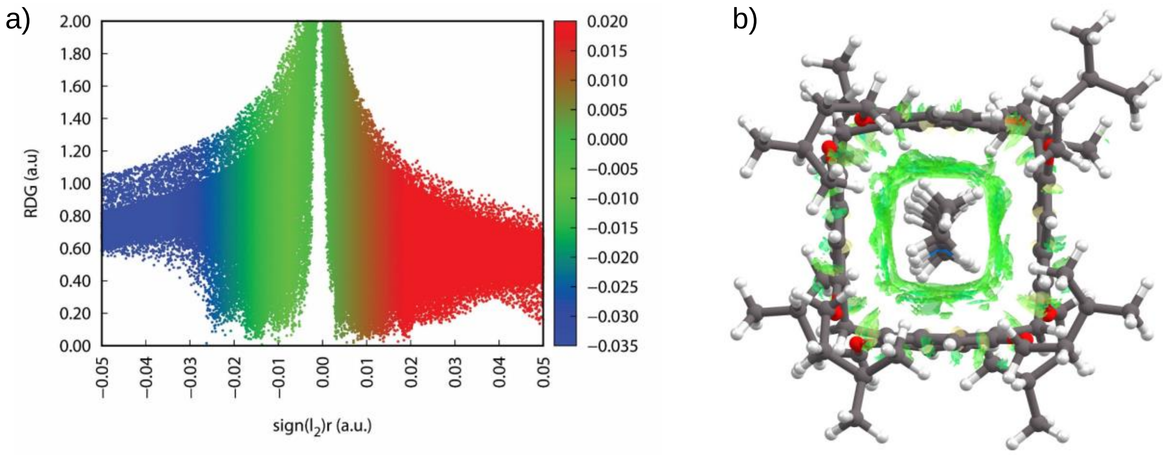

Figure S67: a) Plot of RDG versus sign(I2)r for **2**$^{+}$ @ **PrS[4]**^{iPe} complex (NCI-RDG isosurfaces with S= 0.25). b) Gradient RDG isosurfaces (0.25) for the noncovalent interaction (NCI) regions **2**$^{+}$ @ **PrS[4]**^{iPe} complexes.

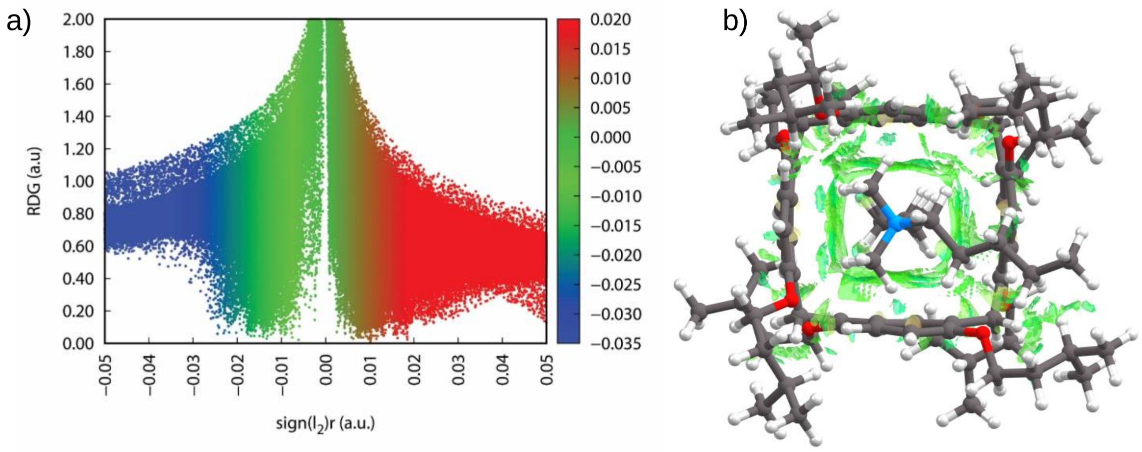

Figure S68: a) Plot of RDG versus sign(I2)r for **3**$^{+}$ @ **PrS[4]**^{iPe} complex (NCI-RDG isosurfaces with S= 0.25). b) Gradient RDG isosurfaces (0.25) for the noncovalent interaction (NCI) regions **3**$^{+}$ @ **PrS[4]**^{iPe} complexes.

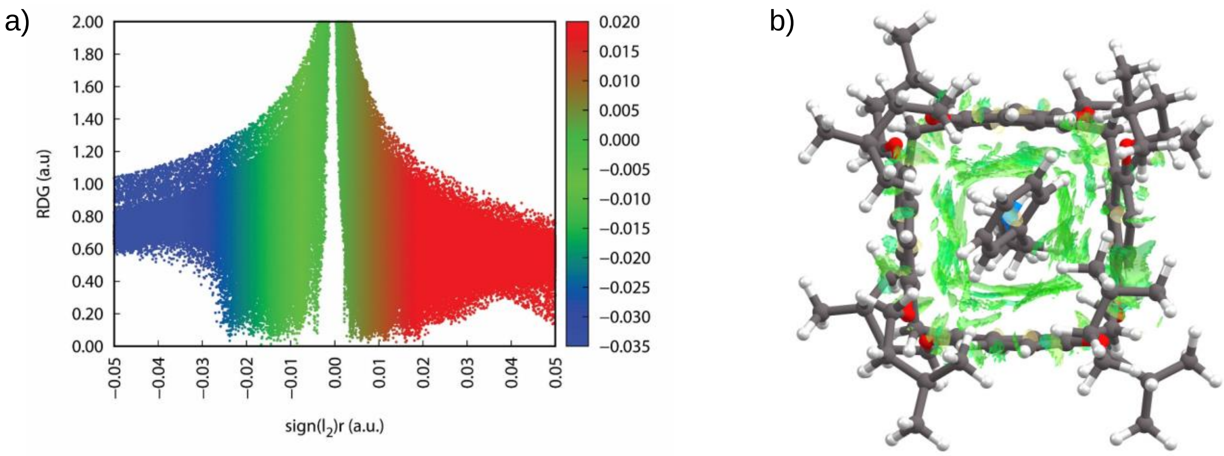

Figure S69: a) Plot of RDG versus sign(I2)r for **5$^+$** @ **PrS[4]^{iPe}** complex (NCI-RDG isosurfaces with S= 0.25). b) Gradient RDG isosurfaces (0.25) for the noncovalent interaction (NCI) regions **5$^+$** @ **PrS[4]^{iPe}** complexes.

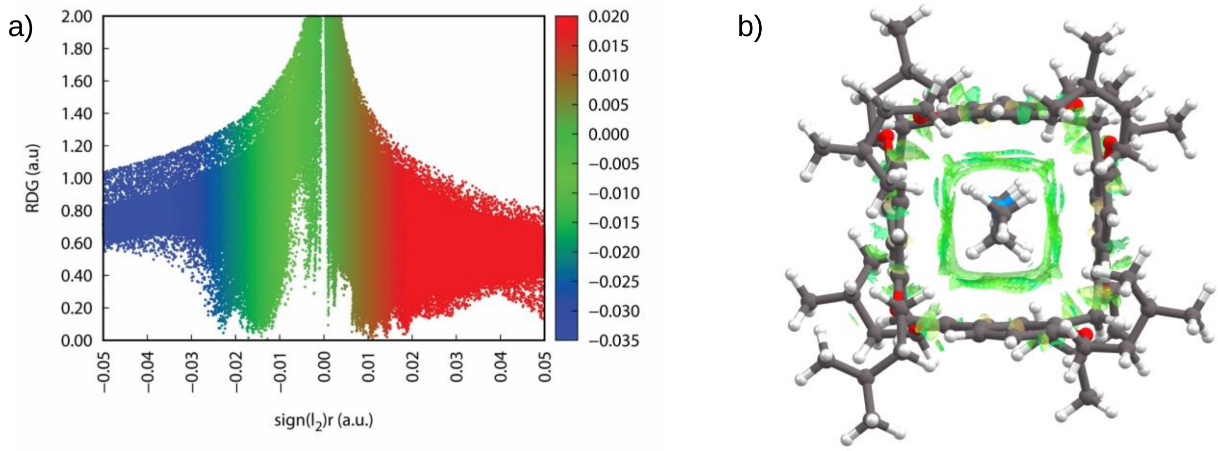

Figure S70: a) Plot of RDG versus sign(I2)r for **4$^+$** @ **PrS[4]^{iPe}** complex (NCI-RDG isosurfaces with S= 0.25). b) Gradient RDG isosurfaces (0.25) for the noncovalent interaction (NCI) regions **5$^+$** @ **PrS[4]^{iPe}** complexes.

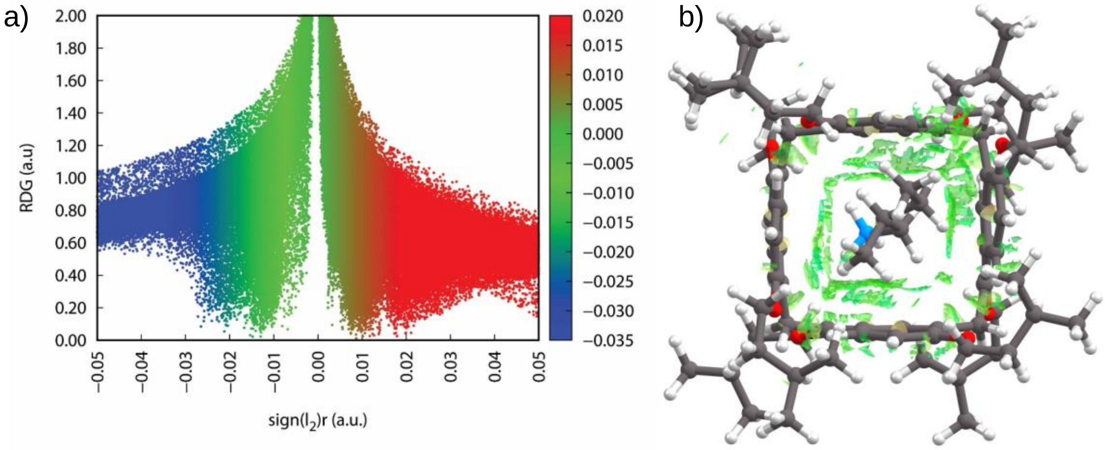

Figure S71: a) Plot of RDG versus sign(I2)r for (*S*)**-7$^+$** @ *pS*-**PrS[4]^{iPe}** complex (NCI-RDG isosurfaces with S= 0.25). b) Gradient RDG isosurfaces (0.25) for the noncovalent interaction (NCI) regions (*S*)**-7$^+$** @ *pS*-**PrS[4]^{iPe}** complexes.

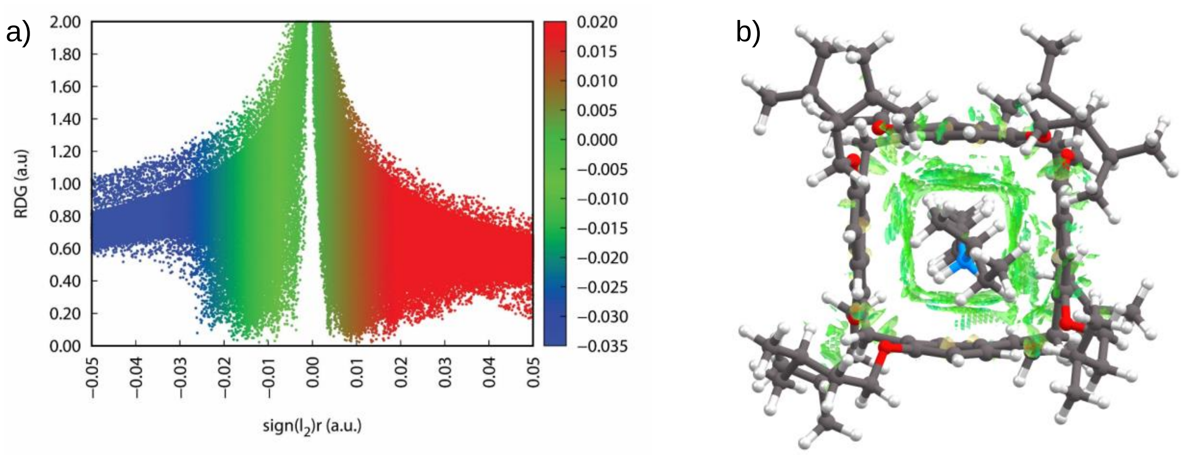

Figure S72: a) Plot of RDG versus sign(I2)r for (*S*)-**9⁺** @ *pR*-**PrS[4]*^iPe^*** complex (NCI-RDG isosurfaces with S= 0.25). b) Gradient RDG isosurfaces (0.25) for the noncovalent interaction (NCI) regions (*S*)-**9⁺** @ *pR*-**PrS[4]*^iPe^*** complexes.

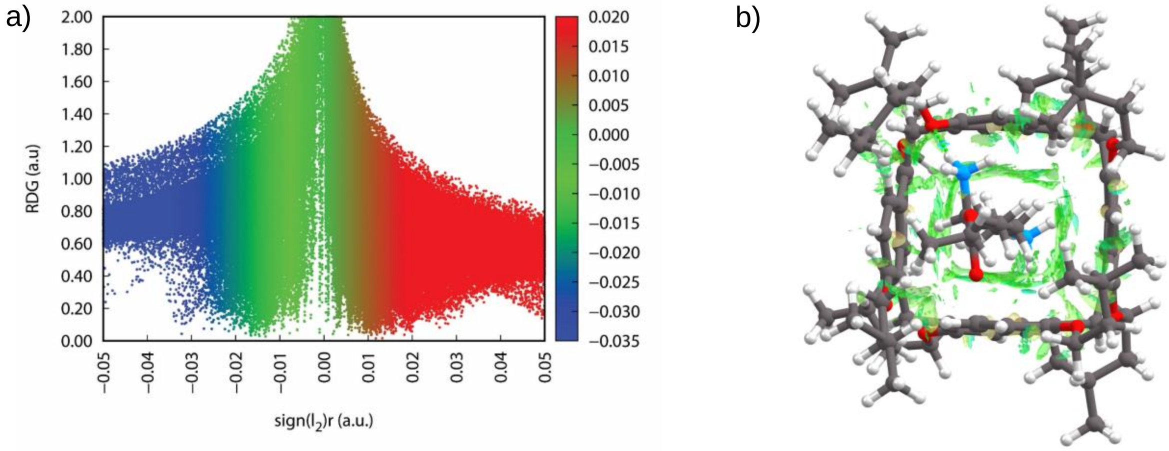

Figure S73: a) Plot of RDG versus sign(I2)r for (*S*)-$\mathbf{6}^{2+}$ @ *pS*-**PrS[4]*^iPe^*** complex (NCI-RDG isosurfaces with S= 0.25). b) Gradient RDG isosurfaces (0.25) for the noncovalent interaction (NCI) regions (*S*)-$\mathbf{6}^{2+}$ @ *pS*-**PrS[4]*^iPe^*** complexes.

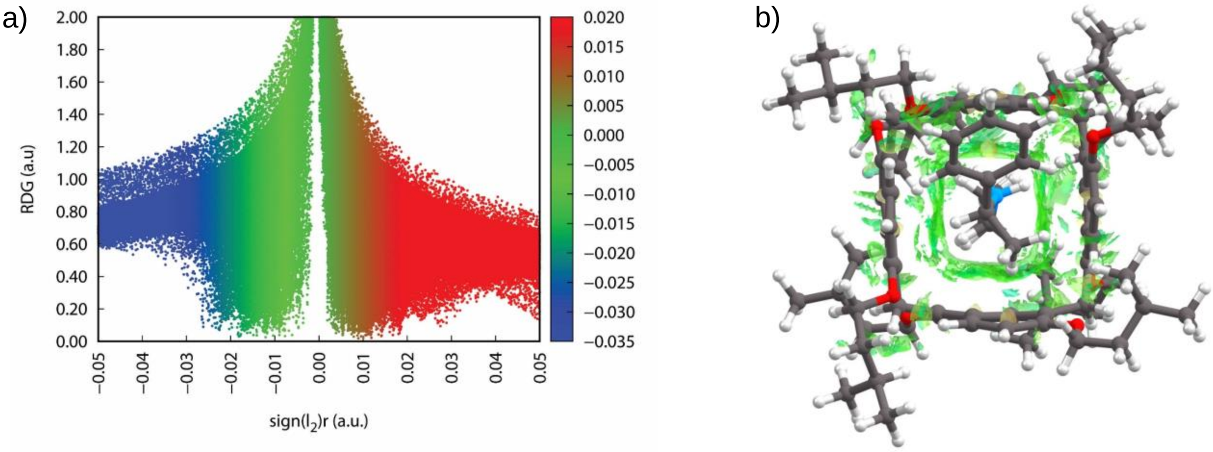

Figure S74: a) Plot of RDG versus sign(I2)r for (*S*)-$\mathbf{8}^{+}$ @ *pS*-**PrS[4]*^iPe^*** complex (NCI-RDG isosurfaces with S= 0.25). b) Gradient RDG isosurfaces (0.25) for the noncovalent interaction (NCI) regions (*S*)-$\mathbf{8}^{+}$ @ *pS*-**PrS[4]*^iPe^*** complexes.

Chiral HPLC separation of enantiomers PrS[4]^{EtCy} and PrS[4]^{iPe}

PrS[4]^{iPe} was analyzed on 250 × 10 mm Phenomenex Cellulose-1 using Hexane / Ethanol 99.8/0.2 v/v as the mobile phase at a flow rate of 2.5 ml/min and injecting 20 µL of 10 µg/mL solution of macrocycle in hexane. The retention times of enantiomers are 9.5 min and 10.7 min.

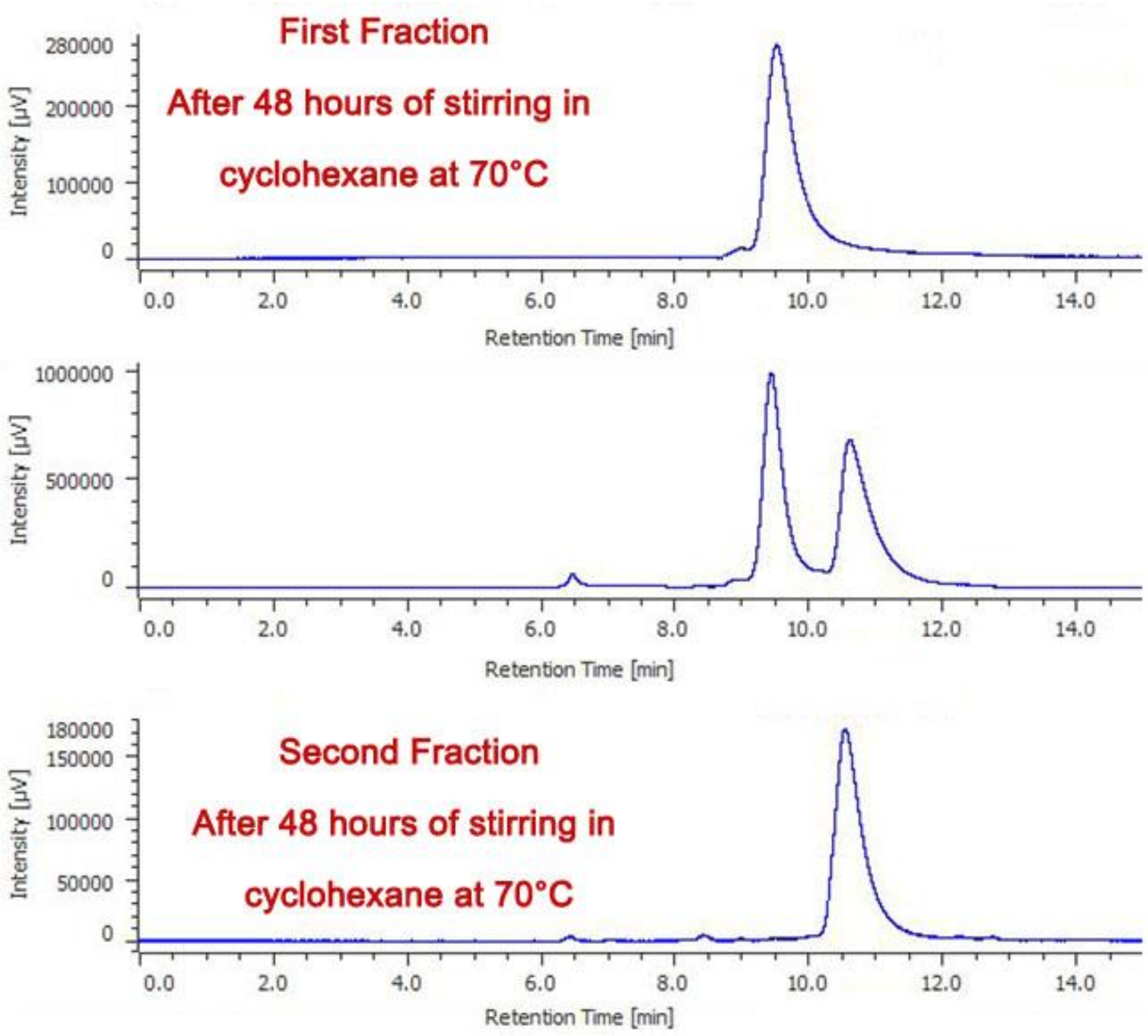

Figure S75: Chiral HPLC traces of **PrS[4]**^{iPe}.

PrS[4]^{EtCy} was analyzed on 250 × 10 mm Phenomenex Cellulose-1 using Hexane / Ethanol 99.8/0.2 v/v as the mobile phase at a flow rate of 3.0 ml/min and injecting 20 µL of 10 µg/mL solution of macrocycle in hexane. The retention times of enantiomers are 10.5 min and 12.9 min.

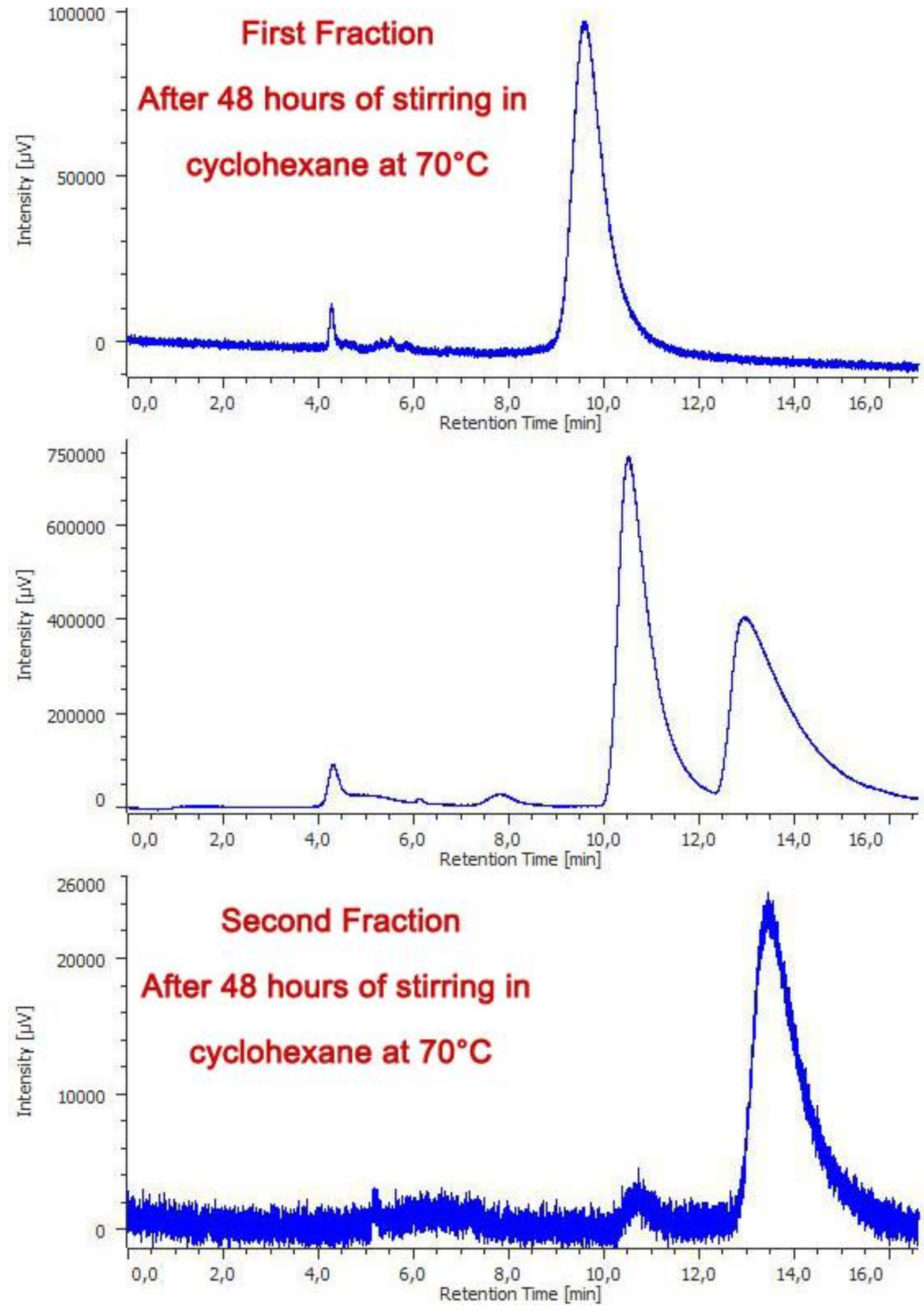

Figure S76: Chiral HPLC traces of **PrS[4]**^{EtCy}.

Crystallographic structure determination of PrS[4]^{iPe}

Single crystals suitable for X-ray diffraction (XRD) analysis were obtained through the slow evaporation of a methanol/dichloromethane solution containing **PrS[4]^{iPe}**. The data collection was performed at the Macromolecular Crystallography XRD1 beamline of the Elettra Synchrotron in Trieste, Italy. The rotating crystal method was utilized in conjunction with a Dectris Pilatus 2M area detector. The single crystals were dipped in paratone cryoprotectant, mounted on a nylon loop, and flash-frozen under a nitrogen vapour stream at 100 K.

Diffraction data were indexed and integrated using the XDS software package, while scaling was performed with XSCALE. The structures were solved using the SHELXT program and refined with SHELXL-19/3 through full-matrix least-squares methods on F^2. Non-hydrogen atoms were refined anisotropically, except for low-occupancy disordered groups. Hydrogen atoms were positioned at calculated locations and refined using the riding model. Crystallographic data and refinement details are presented in Table 3.

The asymmetric unit of the trigonal crystals of **PrS[4]^{iPe}** (space group P -3c1) comprises half of a prismarene molecule and a disordered co-crystallized methanol molecule with an occupancy factor of 0.1 (Figure 78). The **PrS[4]^{iPe}** molecules lie on crystallographic twofold axes passing through two opposite methylene bridges and orthogonal to the cavity central axes (Figure 79). Two-position disorder is observed in the electron density maps for all four *i*Pe side chains (one with 0.80:0.20, two with 0.70:0.30, and one with 0.90:0.10 occupancy factors) (Figure 79). The two methyl groups of one position of the *i*Pe side chain with a 0.70 occupancy factor are further split into two positions with 0.55 and 0.15 occupancy factors. The side chain with a 0.90 occupancy factor involves an *i*Pe group hosted in another crystallographically symmetry-related prismarene molecule. The other conformation of the *i*Pe chain with a 0.1 occupancy factor, which involves a more extensive disorder including part of the naphthalene ring, is outside the cavity, displaced by the co-crystallized methanol molecule from the core of the host prismarene.

While the prismarene molecule shows a C_2 crystallographic symmetry, the **PrS[4]** scaffold shows a pseudo D_4 point symmetry (Figure 80). In fact, all the naphthalene units show the same planar chirality. Therefore, the centrosymmetric crystal is composed of a racemic mixture of the all *pR* and all *pS* enantiomeric pairs. The pseudo D4 point symmetry is stabilized by eight weak intramolecular CH-O hydrogen bonds (C-O distances ranging from 3.05 to 3.22 Å), each with the hydrogen atom of the naphthalene C4 atoms pointing towards the oxygen lone pair of the alkoxy groups.

The crystal packing shows the formation of host-guest interactions between the **PrS[4]^{iPe}** molecules (Figure 81). In particular, the **PrS[4]^{iPe}** macrocycles form a linear homo-polymeric assembly along the c-axis by mutual host-guest interactions, in which each cavity hosts two *i*Pe arms from adjacent prismarenes related by translation of the unit cell and therefore with the same planar chirality (Figure 82). The hexagonal arrangement of these linear homo-chiral polymeric assemblies along the rotoinversion -3 axes is characterized by the alternate disposition of all-*pR* and all-*pS* chains (Figure 83). The four crystallographically independent *i*Pe side chains can be subdivided into three types: the side chains involved in the host–guest interactions; the side chains packed along the rotoinversion -3 axes and the side chains packed along the threefold axes. Of these, the two crystallographically independent *i*Pe side chains of the same naphthalene moiety packed along the threefold axes are the most disordered (Figure 84).

The analysis of the **PrS[4]** scaffold shows only a small deviation from a regular square prism, as indicated by the dihedral angles between the mean planes of the naphthalene rings. These angles are 93° and 96° for the naphthalene moieties related by symmetry, while the two angles between the independent naphthalene moieties are 86°. This deformation, with two opposite dihedral angles being obtuse and two being acute, is apparent in the two diagonal distances between the opposite

methylene bridges, which are 9.24 and 8.86 Å, respectively. All naphthalene planes are slightly bent (with a dihedral angle of 9-10° between the two fused aromatic rings) outward from the cavity forming a saddle geometry. The distances between the adjacent methylene bridges, which define the base of the prism, are 6.36 and 6.44 Å, respectively.

The surface area and volume of the regular square prism enclosed by the aromatic walls of **PrS[4]**, based on the geometrical calculation reported in a previous paper (doi: 10.1039/D1SC02199K), have been evaluated and reported in Figure 85. In particular, the volume (V) of the regular square prism enclosed by the macrocycle was calculated from the area of the square base (B), which represents the cavity opening, and the geometric height (h). In addition, the potential contact surface area (A) was calculated as the total area of the four rectangular prism faces. The calculated internal volume of 87 Å^3 for the **PrS[4]** scaffold is approximately 1/3 of the volume enclosed by **PrS[5]** (255 Å^3) and less than 1/5 of that of **PrS[6]** (490 Å^3). This volume is also less than half of the enclosed volume of the analogous **pagoda[4]arene** based on 2,6-dialkoxylanthracene (206 Å^3). The cavity opening is strictly related to the number of monomers in the macrocycle. Thus, **PrS[4]** shows a narrower cavity (9.3 Å^2) than **PrS[5]** and **PrS[6]** (27.3 and 52.4 Å^2, respectively). The comparison with the **pagoda[4]arene** tetramer shows that the smaller enclosed volume of **PrS[4]** is mainly due to the smaller opening of the cavity (19.1 Å^2 in **pagoda[4]arene**), while the depth of the cavity is similar (9.35 Å and 10.78 Å in prismarene and pagodarene, respectively). Another important geometric feature is the potential contact surface area (A) derived from the total area of the rectangular prism faces. In this case, the **PrS[4]** also exhibits a smaller potential contact area (114 Å^2) than **PrS[5]** (186 Å^2, similar to 188 Å^2 calculated for **pagoda[4]arene**) and **PrS[6]** (252 Å^2).

Table S3 - Crystal Data and Details of the Structure Determination for PrS[4]^{iPe} (CCDC deposit number = 2371538)

Crystal Data	
Formula	C84 H112 O8, 0.2(C1 H4 O1)
Formula Weight	1256.14
Crystal System	trigonal
Space group	P-3c1 (No.165)
a, b, c [Å]	32.736(4) 32.736(4) 11.9950(16)
V [Å^3]	11132(3)
Z	6
D(calc) [g/cm^3]	1.124
μ [mm^{-1}]	0.067
F(000)	4102
**Data Collection**	
Temperature (K)	100
Wavelength [Å]	0.70000
θ Min-Max [°]	0.7, 29.5
Dataset HKL range	- 46: 46 ; -46: 46 ; -16: 16
Reflections Tot., Uniq., R(int)	206561, 10841, 0.047
Observed Data [I > 2.0 σ(I)]	7150
**Refinement**	
Nref, Npar	10841, 547
R, wR2, S	0.0803, 0.2728, 1.07
Min., Max. Resd. Dens. [e/Å^3]	-0.40, 0.34

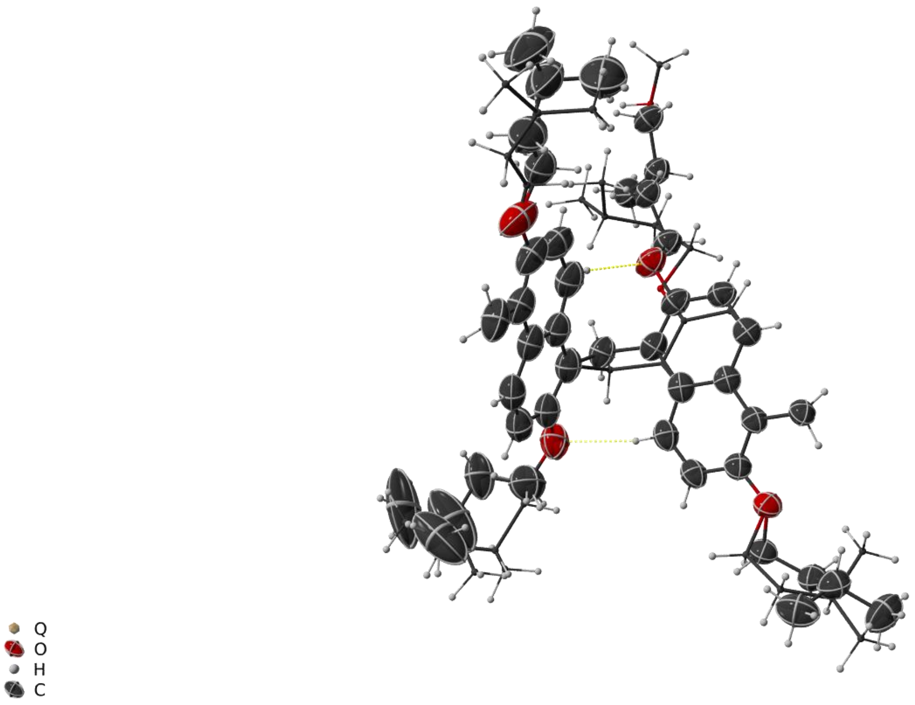

Figure S77: ORTEP drawing of **PrS[4]**^{iPe}. Ellipsoids at 50% probability for the anisotropic thermal factors. Hydrogen atoms and low-occupancy disordered atoms refined isotropically are shown in ball-and-stick representation.

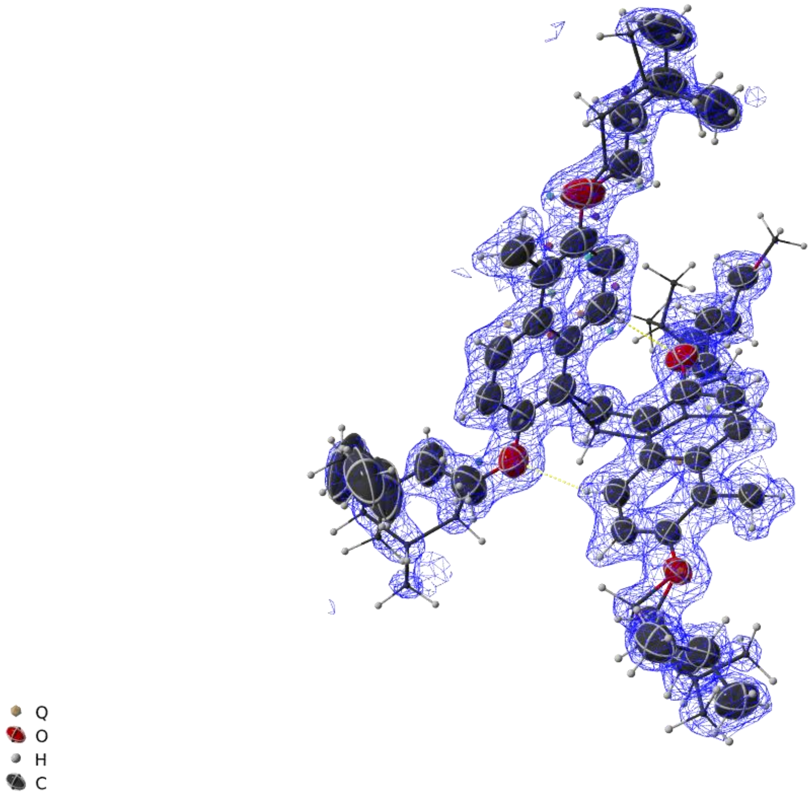

Figure S78: ORTEP drawing of **PrS[4]**^{iPe}, showing the atomic electron density map. Ellipsoids at 50% probability for the anisotropic thermal factors. Hydrogen atoms and low-occupancy disordered atoms refined isotropically are shown in ball-and-stick representation.

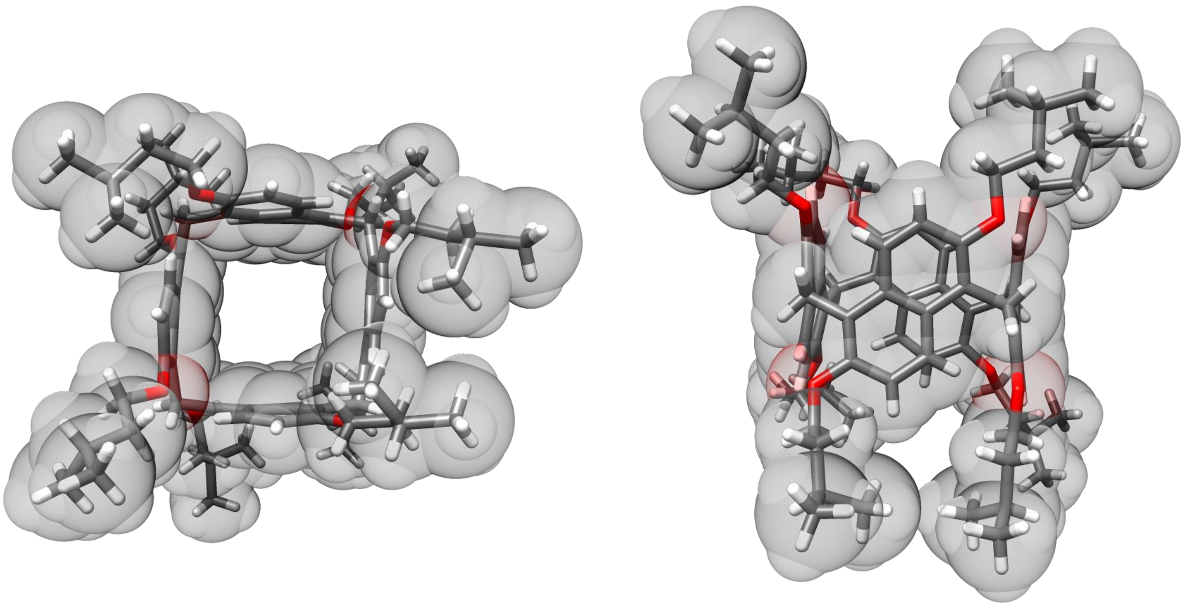

Figure S79: Top view (a) and side view (b) of **PrS[4]^{iPe}**. The molecule is show as a capped stick representation inside its van der Waals surface, which is transparent to aid visualisation. Disordered atoms and solvent molecules have been removed for clarity.

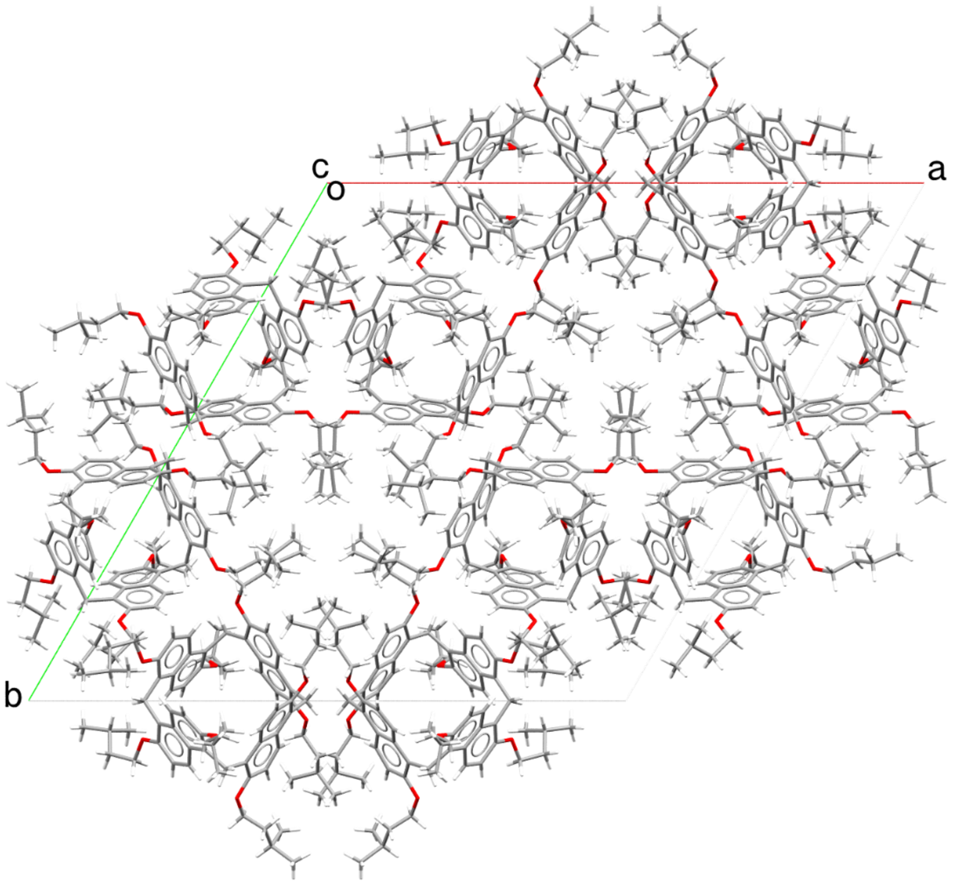

Figure S80: Crystal packing of **PrS[4]^{iPe}** structure, as viewed along the c-axis, with CPK colours.

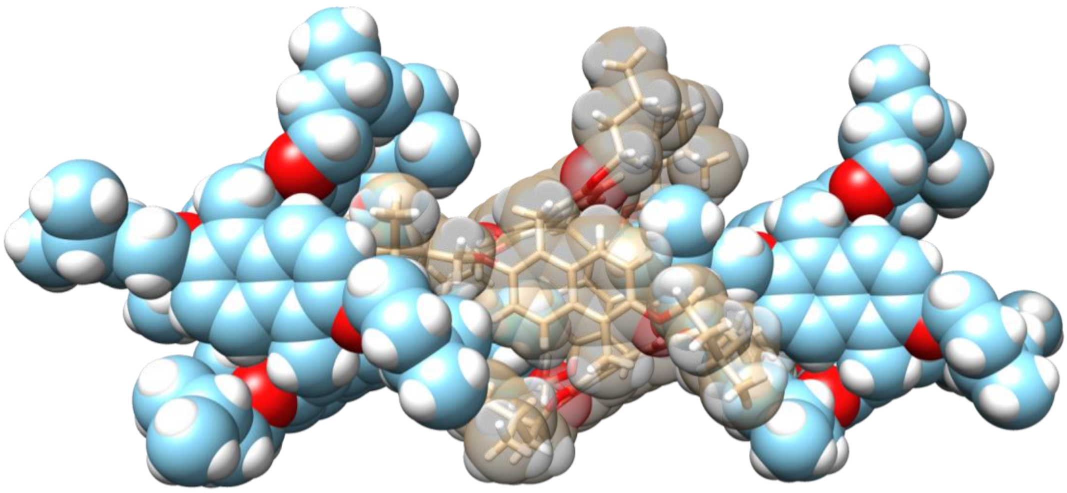

Figure S81: Host-guest interactions in **PrS[4]^{iPe}** crystal structure. The **PrS[4]^{iPe}** macrocycles form a linear homo-polymeric assembly along the c-axis by mutual host-guest interactions, in which each cavity (central brown molecule) hosts two *iPe* arms from adjacent prismarenes (lateral cyan molecules). The central molecule is shown as a capped stick representation inside its van der Waals surface, which is transparent to aid visualisation of the encapsulated *iPe* arms of the adjacent molecules, also represented by van der Waals spheres.

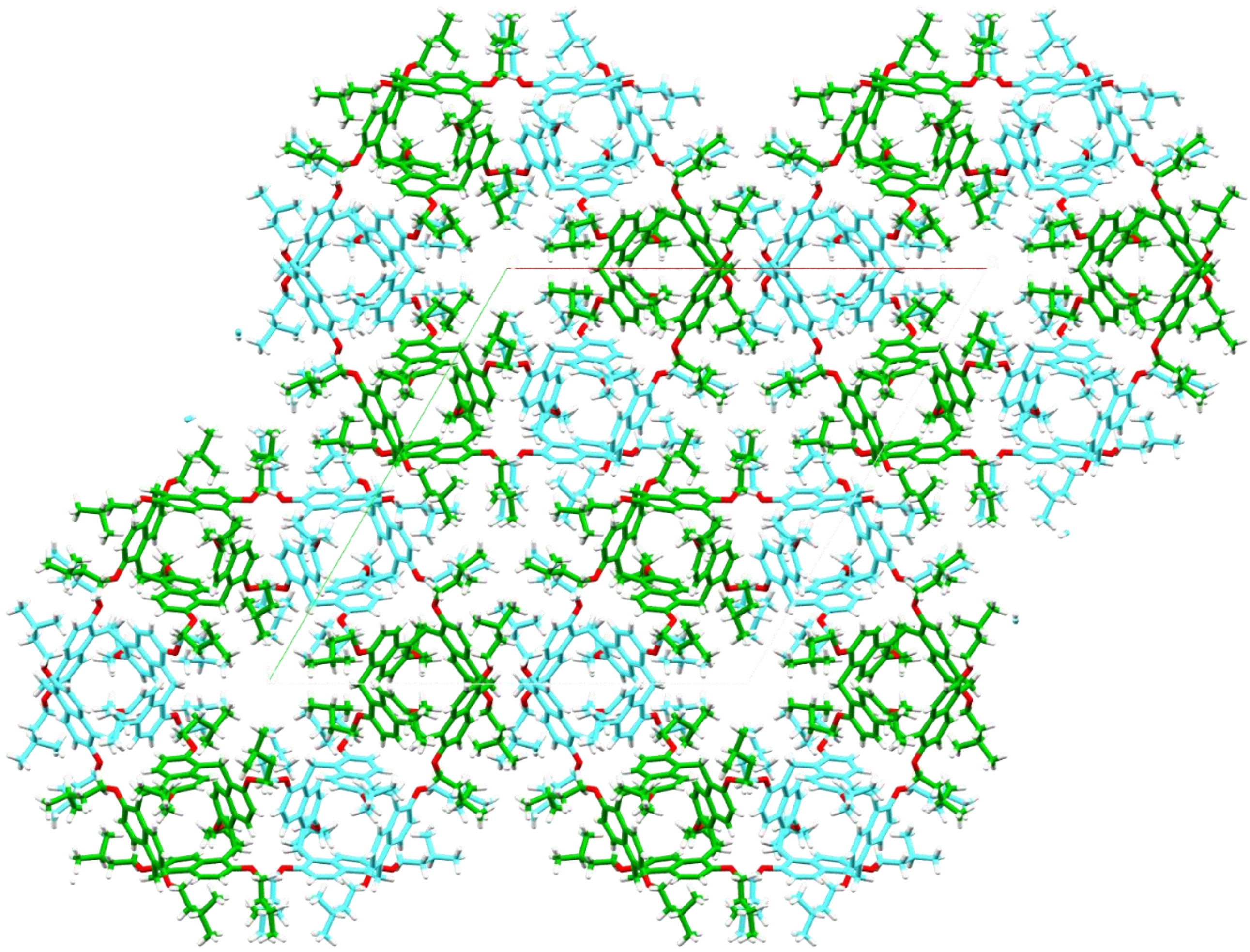

Figure S82: Crystal packing of **PrS[4]^{iPe}** structure, as viewed along the c-axis. Carbon atoms of **PrS[4]** antiomeric pairs, all *pR* and all *pS*, are coloured in cyan and green, respectively.

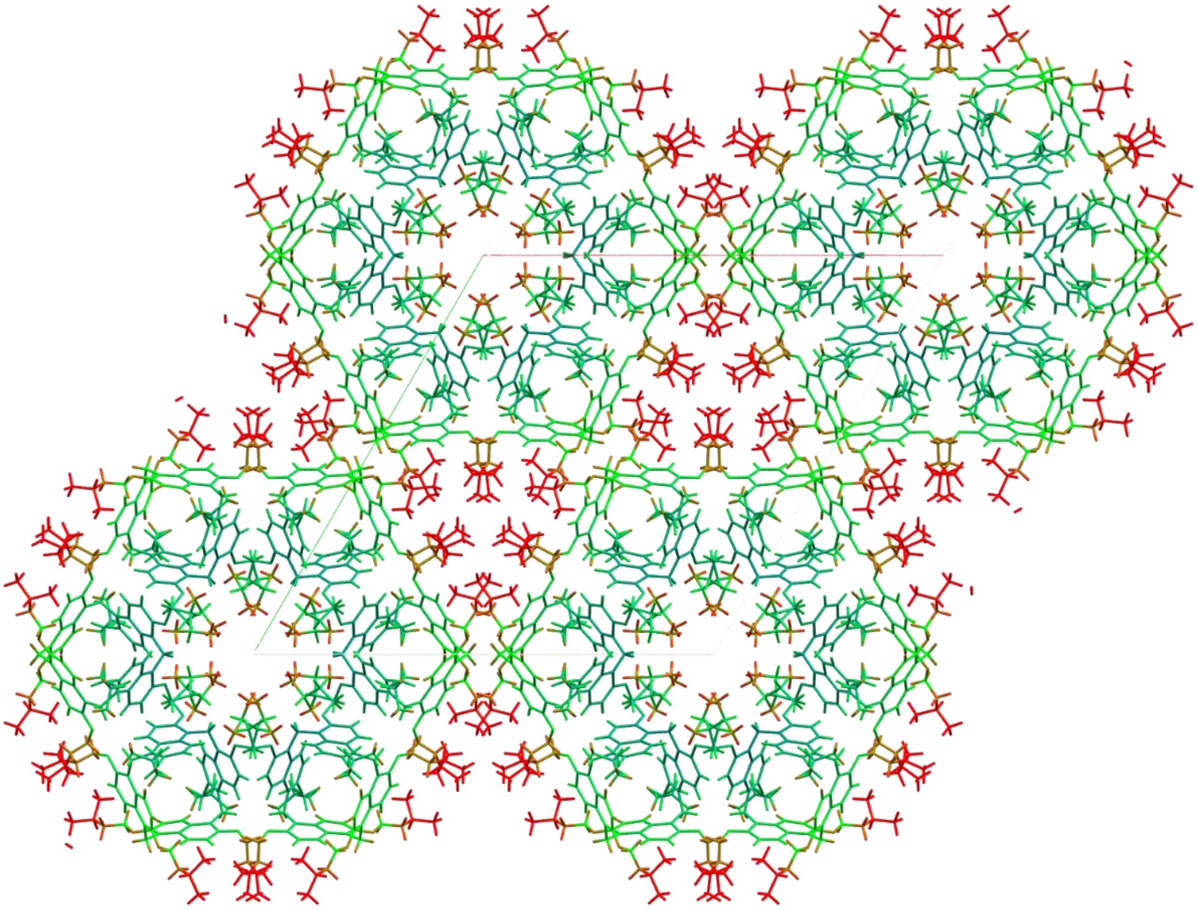

Figure S83: Crystal packing of **PrS[4]**^{iPe} structure, as viewed along the c-axis, by atomic displacement colours (red high thermal factors; blue low thermal factors).

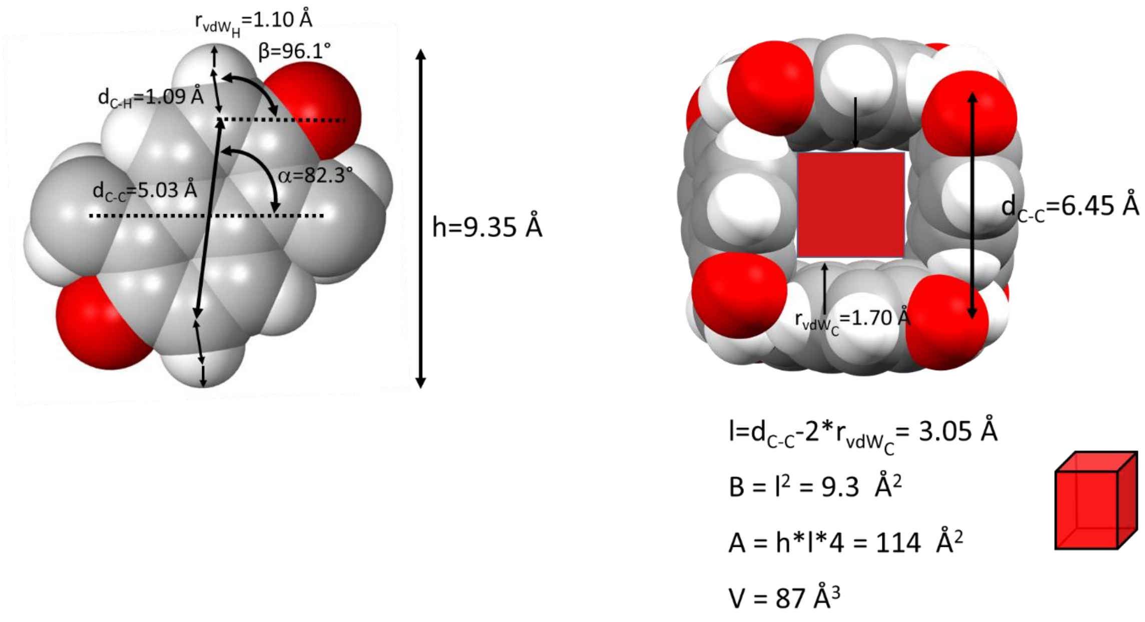

Figure S84. Calculation of the potential void volume in **PrS[4]**.

UV-Vis and fluorescence characterization

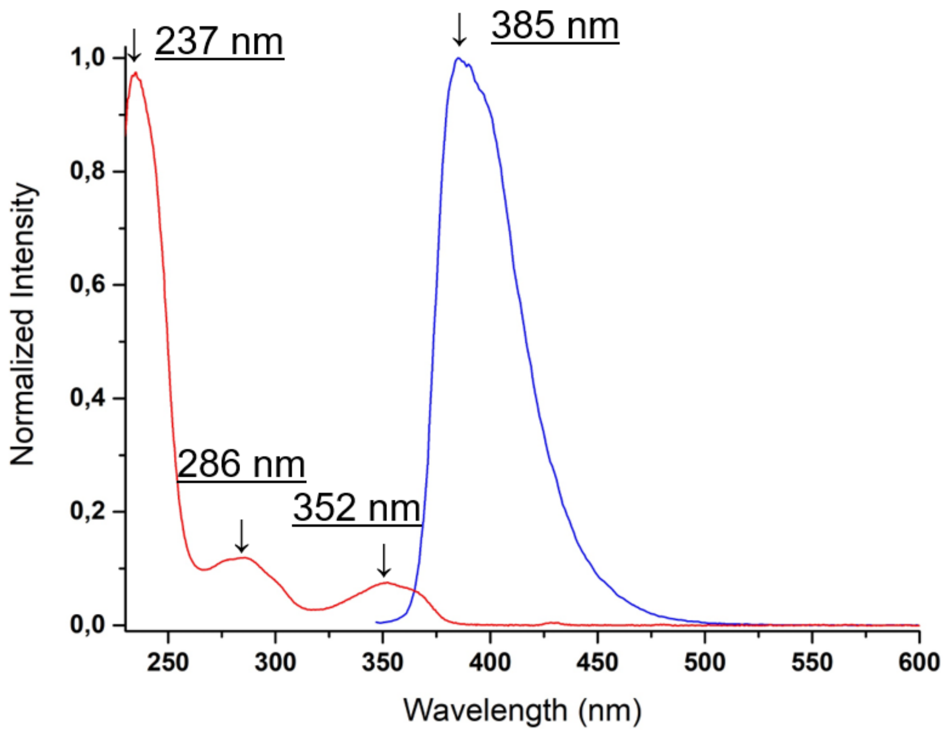

Figure S85: Absorption spectrum of **PrS[4]*iPe*** (red line) and emission spectra of **PrS[4]*iPe*** λ_{exc} = 237 nm (blue line) in dichloromethane.

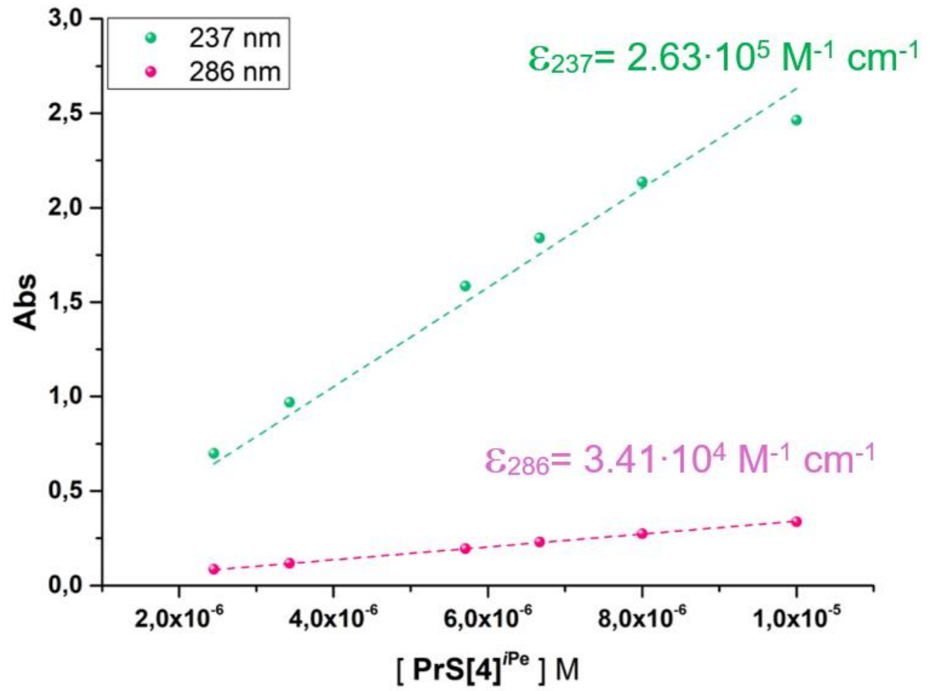

Figure S86: Beer-Lambert plots for the determination of the extinction coefficient of **PrS[4]*iPe*.**

ECD Studies

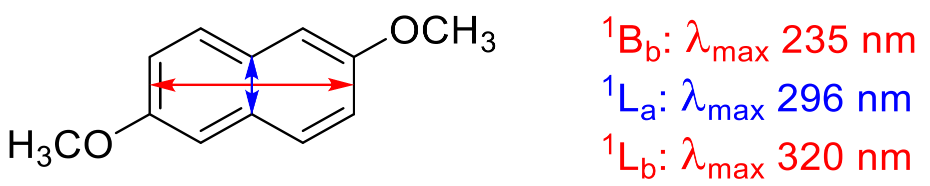

Figure S87. Main electronic transitions of the 2,6-dimethoxynaphtalene chromophore, the polarization directions and wavelength their maximum absorbance.

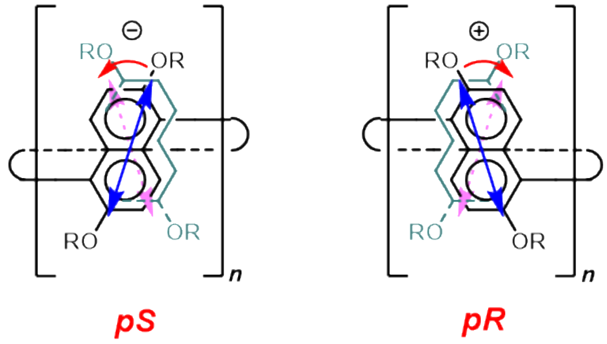

Figure S88. Schematic representation of the naphthalene transition directed along the O-O axis.The red arrow depict the chirality sense.

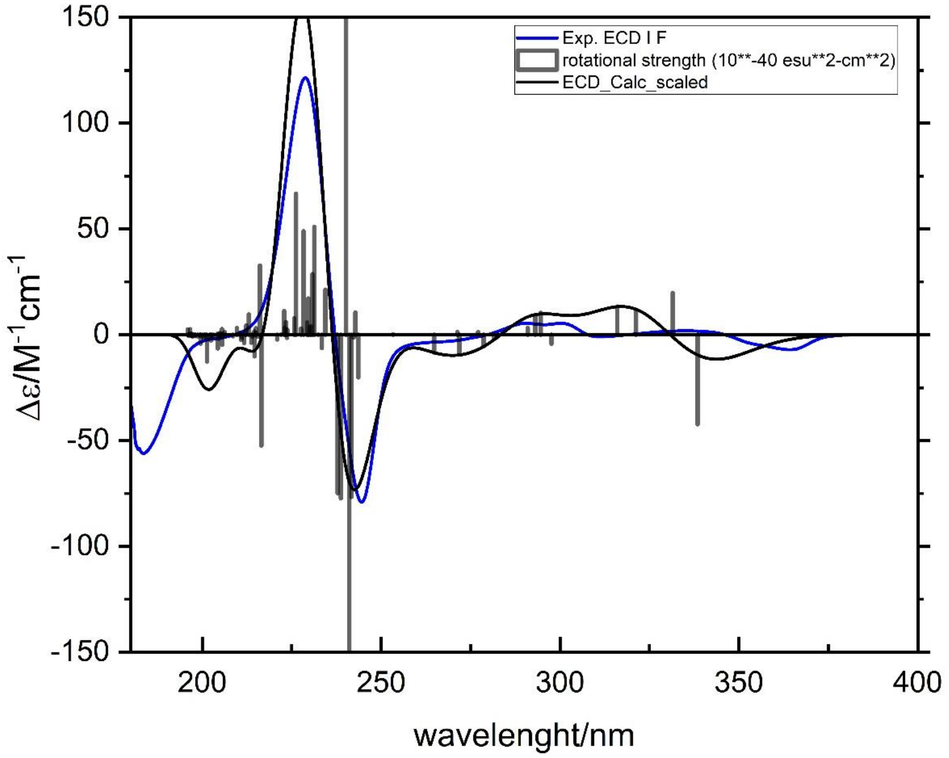

Figure S89. Experimental ECD spectrum of (–)-**PrS[4]**^{iPe} (blue trace) and computed ECD spectrum of all-p*S*- **PrS[4]**^{iPe} (black trace, TDDFT/CAM/B3LYP/6-311G(d,p)/ gas phase) with bars referring to computed rotational strengths (shift +18nm).

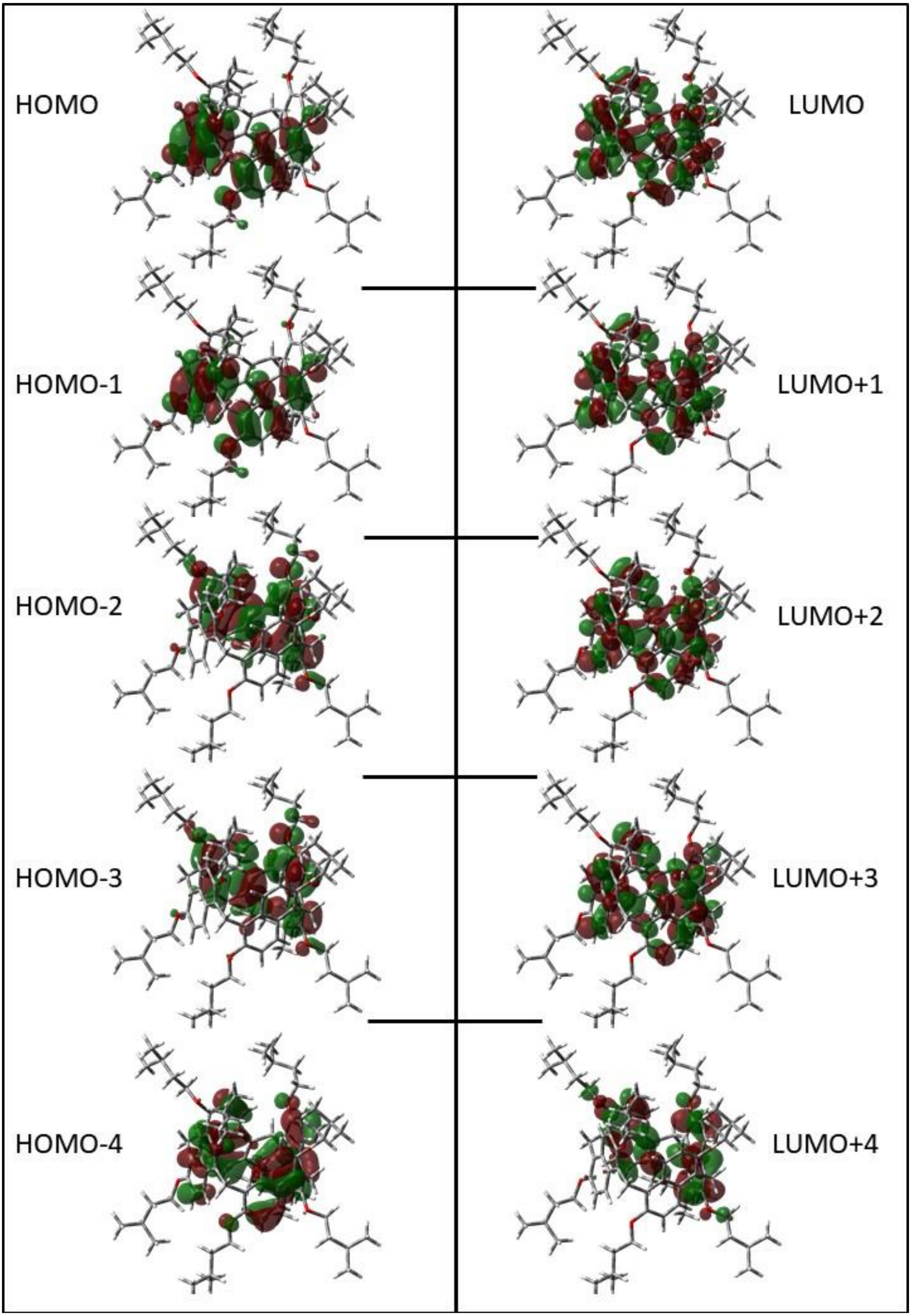
HOMO
LUMO
HOMO-1
LUMO+1
HOMO-2
LUMO+2
HOMO-3
LUMO+3
HOMO-4
LUMO+4

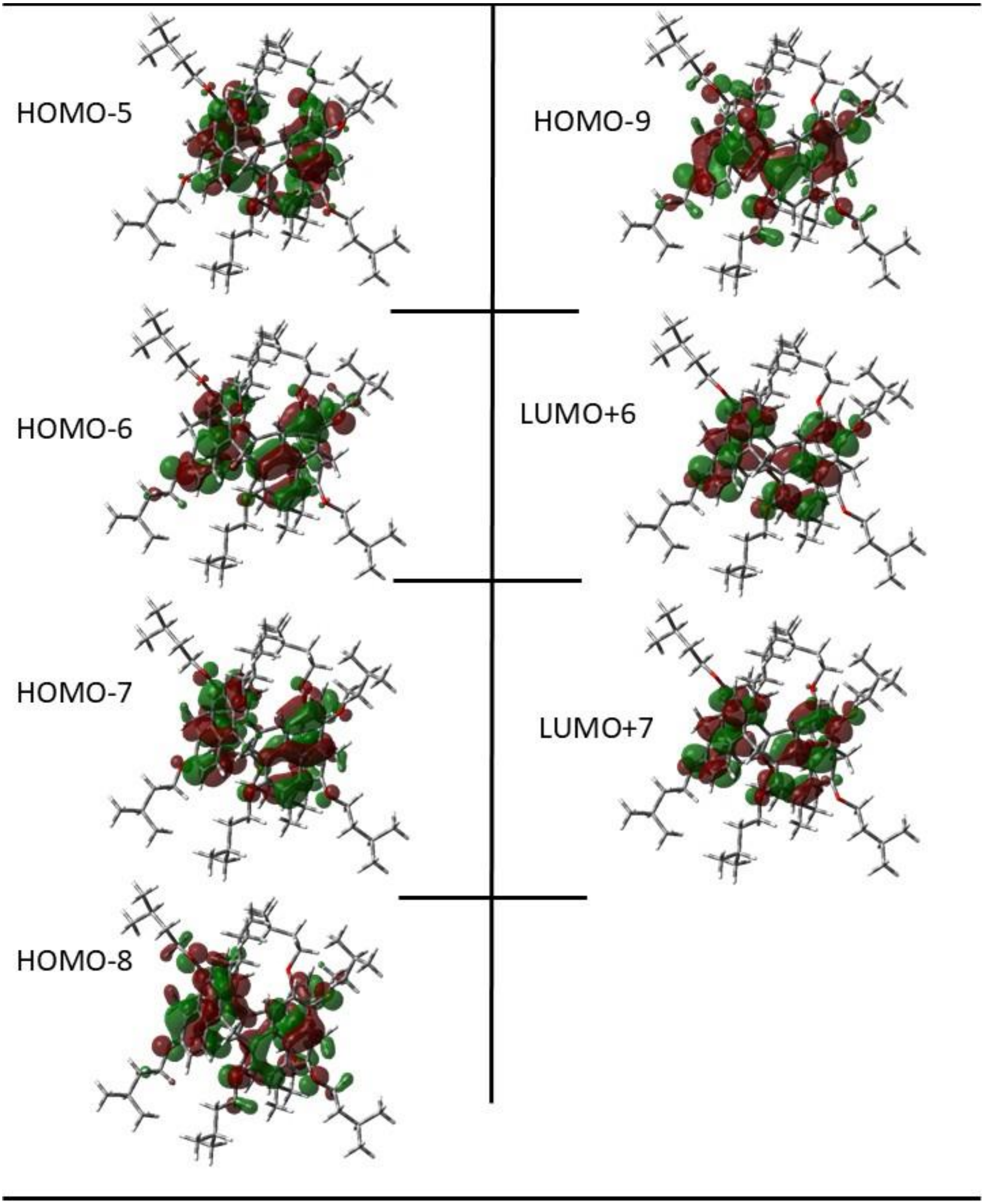

Figure S90. Graphical representation of the MOs allied to the main transitions involved in the ECD CE generation. TDDFT/CAM-B3LYP/6-311G(d,p), iso value=0.2.

Table S4. Rotatory Strengths (R) in cgs (10**-40erg-esu-cm/Gauss), velocity formalism.

state	XX	YY	ZZ	R(velocity)	E-M Angle	nm	state	XX	YY	ZZ	R(velocity)	E-M Angle	nm
1	-40.4521	-728.437	-499.059	-422.649	125.48	320.4	51	-46.682	10.6145	0.0254	-12.014	172.97	197.47
2	178.2186	414.8123	0	197.677	90	313.5	52	16.0835	-115.166	107.4894	2.8023	88.94	197.46
3	-449.109	283.1696	515.4447	116.5017	78.46	303.2	53	-62.4537	62.391	94.1614	31.3662	76.46	196.98
4	272.3828	128.379	0	133.5873	90	298	54	-3.7154	33.8354	0.0108	10.0436	3.79	196.91
5	1.5085	-74.642	-53.9844	-42.3727	125.03	279.6	55	-74.8224	-94.9801	-134.98	-101.594	115.74	196.6
6	159.4299	150.6143	0	103.3481	90	276.6	56	-149.141	196.2902	-0.0004	15.7163	0.64	196.5
7	-71.1757	114.2163	237.6579	93.5662	52.46	275.1	57	-74.5087	-32.3799	81.7654	-8.3744	98.22	196
8	54.4402	42.2292	0	32.2231	90	273	58	59.3039	-171.056	0	-37.2508	90	195.7
9	-4.4201	1.9917	-32.4692	-11.6325	146.69	264.4	59	49.2767	27.194	211.5922	96.021	35.48	195
10	-41.5382	-18.075	0	-19.8711	90	263.6	60	61.2756	70.7281	0	44.0012	90	194.3
11	-155.798	-8.0843	0	-54.6275	90	260.6	61	11.3894	67.7038	-31.2852	15.936	1.09	194
12	150.0195	-34.2713	-77.231	12.8391	86.31	259.1	62	-60.2258	31.3073	-0.0021	-9.6402	176.1	193.73
13	-80.9003	-175.327	0	-85.4089	90	253.8	63	-29.2386	7.3603	-68.4608	-30.113	115.12	193.69
14	14.2608	26.4055	-2.4916	12.7249	67.82	253.3	64	-19.3567	-0.3089	-21.5346	-13.7334	177.29	193
15	-55.764	-165.229	-0.011	-73.668	178.71	246.8	65	-31.1473	-41.5547	0	-24.234	90	192.9
16	-7.4985	123.1477	-147.515	-10.622	92.31	246.78	66	3.1632	19.8955	0	7.6862	90	192.5
17	-2.1988	5.3076	1.7245	1.6111	70.68	235.3	67	25.4727	-1.2972	73.8007	32.6587	3.58	191.7
18	0.2459	-0.8353	0	-0.1965	90	234.9	68	-6.768	-13.4535	0	-6.7405	90	190.6
19	-692.722	88.9773	0	-201.248	179.94	225.6	69	-2.8468	6.9139	-16.2066	-4.0465	111.11	188.5
20	275.7196	11.3124	28.5135	105.1818	67.48	224.8	70	9.4115	26.8046	0	12.072	90	188.3
21	58.9579	-96.5138	-0.0001	-12.5187	178.91	224.3	71	-2.7424	-17.3949	0	-6.7124	90	188.1
22	62.3479	286.3479	-2647.84	-766.383	158.73	223.7	72	-16.918	-61.2786	-63.5541	-47.2502	160.81	187.6
23	-2570.27	-2511.6	0	-1693.96	179.99	223.1	73	16.9486	60.0639	4.5023	27.1716	57.82	187.5
24	3081.159	1427.1	3250.257	2586.172	39.25	222.2	74	8.8006	9.0449	0	5.9485	90	187.4
25	-907.201	-1411.57	0	-772.923	179.97	220.7	75	42.5119	-20.8219	0	7.23	90	186.8
26	-157.58	1246.648	-3333.44	-748.123	107.22	219.8	76	16.5326	-54.9156	-16.8644	-18.4158	106.9	186.6
27	33.761	136.8664	467.627	212.7515	34.86	216.4	77	-30.2554	-43.2731	-121.149	-64.8926	165.1	186.3
28	-73.5243	-115.093	0.0001	-62.8724	179.81	215.4	78	19.0205	-6.8286	-0.0003	4.0639	90	186.2
29	-11.3194	13.0995	0.0001	0.5934	90	214.4	79	-14.1011	-8.9975	0	-7.6995	90	185.1
30	323.7965	288.3501	918.78	510.3089	2	213.3	80	-7.4774	0.5394	-19.8213	-8.9198	148.11	184.7
31	255.6205	83.5931	519.0841	286.0992	40.14	212.8	81	-5.8355	2.43	-10.1318	-4.5124	98.19	184.38
32	67.744	55.3178	0.0002	41.0207	0.34	212.4	82	-31.9659	-32.3176	-0.0002	-21.4279	179.08	184.35
33	116.1557	-10.8323	0.0001	35.1079	90	212	83	-1.1893	-23.5273	0	-8.2389	90	184.1
34	327.4355	147.9608	38.2158	171.204	62.17	211.6	84	-274.893	7.6717	-108.97	-125.397	178.87	183.3
35	95.3384	77.0061	0.0001	57.4482	90	211.3	85	-0.4735	-24.6885	-6.3127	-10.4916	179.23	183
36	401.3796	113.9079	953.4118	489.5664	22.22	210.3	86	-20.2471	-18.5376	0	-12.9282	90	182.5
37	-12.25	264.7866	-186.451	22.0285	78.2	209.6	87	-2.9843	-29.0846	2.5208	-9.8494	141.97	182
38	11.5347	10.1647	-0.009	7.2302	2.35	209.58	88	14.7565	-37.9574	-101.106	-41.4358	119.5	181.5
39	149.3599	463.4539	1386.575	666.4628	25.61	208.2	89	0.7502	-3.1701	-0.0001	-0.8066	90	181.4
40	136.7049	97.6261	0	78.1103	90	207.7	90	-4.9991	20.3608	-24.4713	-3.0365	155.78	181.3
41	66.1992	-30.195	35.118	23.7074	63.19	205.8	91	-1.1329	0.9781	-0.0001	-0.0516	90	181.1
42	-62.6831	18.6389	0.0003	-14.6813	179.48	205.7	92	-3.2648	-19.9067	0	-7.7238	90	180.7
43	108.1795	-10.0977	79.5509	59.2109	58.03	205.4	93	3.3945	12.544	-0.0031	5.3118	3.2	180.48
44	-20.6573	-0.8795	26.3899	1.6177	83.9	205.2	94	-11.2584	-0.2122	-4.2855	-5.252	163.07	180.47
45	73.0774	25.6894	0.002	32.9229	90	205.1	95	-7.3626	1.2559	-18.3502	-8.1523	125.38	179.3
46	-1.4371	185.9561	152.2558	112.2582	43.24	204.9	96	-15.4322	2.4719	0	-4.3201	90	178.9
47	3.937	0.9605	0	1.6325	90	204	97	-0.6561	2.6572	13.0144	5.0052	17.61	178.7
48	-14.0147	-52.4561	0	-22.1569	90	202.9	98	-8.0155	-8.1086	0	-5.3747	90	178.6
49	-639.013	-277.217	-655.124	-523.785	148.31	198.5	99	49.6524	-9.8844	-49.3478	-3.1933	97.54	178
50	761.7028	218.8291	0	326.844	0.03	198.1	100	114.6668	-11.4333	-25.8043	25.8097	33.64	177.9

Table S5. Main representative transitions involved in the ECD couplet bands. See **Figure S90** for the MOs involved.

Excited	State		1: Singlet-A 3.8702 eV 320.35 nm f=0.0794 <S**2>=0.000		
337	->	341	0.11979	HOMO-3	LUMO
338	->	342	0.14655		
339	->	341	0.26517	HOMO-1	LUMO
339	->	343	0.32506	HOMO-1	LUMO+2
340	->	342	0.25581	HOMO	LUMO+2
340	->	344	0.41869	HOMO	LUMO+3
Excited	State		2: Singlet-A 3.9553 eV 313.46 nm f=0.0769 <S**2>=0.000		
339	->	342	0.25072		
339	->	344	0.3446		
340	->	341	0.34998	HOMO	LUMO
340	->	343	0.35479	HOMO	LUMO+2
Excited	State		4: Singlet-A 4.1599 eV 298.05 nm f=0.0554 <S**2>=0.000		
333	->	341	0.12547		
333	->	345	0.10151		
334	->	342	-0.10971		
334	->	346	-0.10828		
337	->	342	0.31709	HOMO-3	LUMO+1
337	->	344	-0.2408		
338	->	341	0.32717	HOMO-2	LUMO
338	->	343	-0.2887		
338	->	345	-0.11838		
339	->	342	-0.12057		
340	->	341	-0.15331		
Excited	State		5: Singlet-A 4.4341 eV 279.61 nm f=0.0024 <S**2>=0.000		
333	->	344	0.1246		
334	->	343	-0.14632		
335	->	342	-0.2047		
335	->	344	-0.1662		
336	->	341	0.24881	HOMO-4	LUMO
336	->	343	0.18818		
337	->	341	0.18833		
339	->	341	0.30112	HOMO-1	LUMO
339	->	347	0.13518		
339	->	349	-0.13851		
340	->	344	-0.21523		
340	->	350	-0.15879		

Excited	State		23: Singlet-A 5.5567 eV 223.13 nm f=0.6237 <S**2>=0.000		
331	->	341	0.16421		
331	->	343	0.11854		
332	->	342	-0.18625		
332	->	344	-0.18392		
333	->	341	0.13869		
333	->	343	-0.11844		
334	->	342	-0.13608		
335	->	341	0.26829	HOMO-5	LUMO
335	->	343	0.1017		
337	->	342	0.10066		
337	->	344	0.10271		
337	->	346	0.17897		
338	->	345	0.22185	HOMO-2	LUMO+4
339	->	350	-0.12101		
340	->	347	0.18604		
340	->	349	-0.1577		
Excited	State		39: Singlet-A 5.9550 eV 208.20 nm f=0.4940 <S**2>=0.000		
329	->	342	-0.11711		
330	->	341	-0.19222		
332	->	343	-0.15416		
333	->	342	-0.19089		
333	->	344	-0.30268		
334	->	341	0.12024		
334	->	343	0.39377	HOMO-6	LUMO+2
336	->	343	0.10176		
338	->	346	0.189	HOMO-2	LUMO+5

Table S6. Cartesian coordinates of all-*pS*-**PrS[4]**^{iPe} used for ECD calculation

0 1

O	1.40203300	-5.11483700	-0.45172800
O	2.37293200	1.48289300	-4.41240200
O	-1.35451800	-2.44786300	4.83039300
C	1.04732800	-3.34643300	2.18235600
H	1.22873500	-4.16637800	1.50383400
C	0.00012900	0.00016600	-4.37806100
H	-0.28231600	-0.82487000	-5.03167900
C	2.29576400	-1.81878400	-1.79842700
C	3.37469500	-0.90095300	-1.78059600
H	4.20174000	-1.04090700	-1.10138100
C	3.41953300	0.15856300	-2.65095600
H	4.28253900	0.81004900	-2.63342700
C	2.34953500	0.41828100	-3.53615100
C	1.21889600	-0.38622000	-3.53899000
C	1.23365600	-1.57091300	-2.73209100
C	0.23649400	-2.56982500	-2.86230200
H	-0.57567400	-2.43376200	-3.56020000
C	0.30540700	-3.74227700	-2.15464700
H	-0.44950800	-4.49810800	-2.32424200
C	1.32781200	-3.95813900	-1.20282400
C	2.28256400	-2.98255500	-0.95883200
C	3.55727900	2.27006400	-4.53258300
H	3.76259400	2.78025400	-3.58455000
H	4.41353900	1.62333900	-4.76395200
C	3.32818200	3.26546600	-5.66239700
H	2.49346600	3.92473600	-5.39586200
H	3.00958800	2.69851800	-6.54306700

C	4.56215600	4.11368000	-6.02187700
H	5.38720800	3.42438300	-6.24822600
C	4.28690200	4.94001900	-7.28580600
H	4.00760000	4.29998000	-8.12745400
H	5.16833000	5.51685900	-7.58008700
H	3.46727600	5.64719600	-7.11866100
C	5.01156800	5.02457200	-4.86915400
H	4.20620700	5.70816800	-4.57846100
H	5.87054500	5.63275200	-5.16676100
H	5.30463600	4.45817600	-3.98164900
C	0.59767100	-6.25186000	-0.77427000
H	-0.44902100	-5.95714300	-0.89497500
H	0.65708800	-6.88080200	0.11766700
C	1.12886800	-7.02287300	-1.98330000
H	1.06064700	-6.40147000	-2.88298800
H	2.19510600	-7.20205600	-1.80912900
C	0.42074400	-8.36817000	-2.23269600
H	0.45194700	-8.94197200	-1.29589400
C	1.17309400	-9.17687300	-3.29856900
H	1.17983900	-8.64693500	-4.25704300
H	0.70250200	-10.15093600	-3.46060600
H	2.21276600	-9.35008900	-3.00692100
C	-1.05344300	-8.19421100	-2.62999200
H	-1.52385800	-9.16424600	-2.81441700
H	-1.13913200	-7.60449300	-3.54928600
H	-1.63690300	-7.69143700	-1.85441100
C	1.90503800	-2.22167400	2.17865500
C	1.61306600	-1.16054600	3.10100300
C	2.55152400	-0.09734600	3.20559700
H	2.36577500	0.72353700	3.88084100
C	0.00191300	-3.44079500	3.06664500
H	-0.60564300	-4.33556400	3.06783000
C	-0.30155000	-2.37674800	3.94397600
C	0.43967100	-1.20232000	3.92147300
C	3.28237700	-3.15404000	0.18525000
H	4.29803200	-3.02067500	-0.18886900
H	3.21335100	-4.17825500	0.55039300
C	3.06593300	-2.16334300	1.32907600
C	3.71603000	-0.12072000	2.48749800
H	4.44676300	0.67387700	2.58984900
C	3.97720300	-1.15169200	1.55835200
O	5.16231700	-1.07855200	0.83518000
C	6.32001000	-1.55778400	1.53627800
H	6.17274800	-2.61409200	1.79047700
H	6.43958400	-1.00103700	2.47533300
C	7.53063800	-1.35342500	0.63726000
H	7.53691000	-0.30333500	0.32607200
H	7.40233900	-1.94880800	-0.27469600
C	8.88029700	-1.69810500	1.29360700
H	8.96258900	-1.11044600	2.21835100
C	8.99369800	-3.18386800	1.66761200
H	8.23731800	-3.48717800	2.39560000
H	9.97316500	-3.40135300	2.10328000
H	8.87681400	-3.81680700	0.78103900
C	10.03801000	-1.28367800	0.37473400
H	10.00546800	-1.84002400	-0.56824600
H	11.00570700	-1.48248500	0.84424600
H	9.99393800	-0.21763900	0.13389200
C	-1.97892800	-3.71017100	5.07552400
H	-1.21203000	-4.46261200	5.29329700
H	-2.52833600	-4.03798800	4.18495300
C	-2.90661300	-3.56141300	6.27384500
H	-2.30199000	-3.23536100	7.12688200
H	-3.28702100	-4.56107400	6.52283300
C	-4.09565200	-2.59809400	6.09741000
H	-3.69345400	-1.62606900	5.79234700
C	-5.07540400	-3.07003900	5.01386600
H	-5.49439900	-4.05120000	5.26503700
H	-4.60027500	-3.15009400	4.03276800
H	-5.90975400	-2.36987300	4.91522000
C	-4.82117800	-2.40781300	7.43651800

H	-5.64716700	-1.69742500	7.34042400
H	-4.14272700	-2.02922600	8.20648700
H	-5.23827600	-3.35548500	7.79565500
C	-0.00007800	-0.00022400	4.75782200
H	-0.81203200	-0.31859400	5.41140700
O	-1.40195500	5.11490600	-0.45145200
O	-2.37268300	-1.48254200	-4.41261900
O	1.35436400	2.44741500	4.83070300
C	-1.04729600	3.34620900	2.18257500
H	-1.22865300	4.16622000	1.50412200
H	0.28259800	0.82525100	-5.03160500
C	-2.29559100	1.81891600	-1.79836700
C	-3.37452100	0.90108000	-1.78064000
H	-4.20158400	1.04097500	-1.10143600
C	-3.41933300	-0.15836500	-2.65108600
H	-4.28233500	-0.80985900	-2.63363000
C	-2.34931000	-0.41800800	-3.53627400
C	-1.21866900	0.38648800	-3.53900900
C	-1.23345100	1.57111600	-2.73201200
C	-0.23628200	2.57003500	-2.86211200
H	0.57592800	2.43401300	-3.55997000
C	-0.30523800	3.74244700	-2.15439600
H	0.44967700	4.49829500	-2.32391000
C	-1.32768500	3.95824500	-1.20260200
C	-2.28242400	2.98262900	-0.95869100
C	-3.55697900	-2.26979000	-4.53280600
H	-3.76218800	-2.78009100	-3.58480900
H	-4.41330600	-1.62311200	-4.76405400
C	-3.32787300	-3.26505000	-5.66274100
H	-2.49303100	-3.92422600	-5.39636700
H	-3.00946000	-2.69797000	-6.54339200
C	-4.56176500	-4.11341100	-6.02216400
H	-5.38695700	-3.42421600	-6.24831200
C	-4.28654900	-4.93954200	-7.28623700
H	-4.00747500	-4.29934900	-8.12784400
H	-5.16792000	-5.51649400	-7.58046900
H	-3.46678000	-5.64660000	-7.11929700
C	-5.01087100	-5.02452400	-4.86949700
H	-4.20536300	-5.70803800	-4.57901800
H	-5.86980000	-5.63279500	-5.16705900
H	-5.30389400	-4.45829300	-3.98187300
C	-0.59773400	6.25202400	-0.77402000
H	0.44898000	5.95742800	-0.89480800
H	-0.65715900	6.88093400	0.11794000
C	-1.12911400	7.02300300	-1.98299400
H	-1.06082000	6.40165300	-2.88271300
H	-2.19537700	7.20198500	-1.80876600
C	-0.42125300	8.36844100	-2.23237500
H	-0.45254900	8.94221800	-1.29556100
C	-1.17378100	9.17701700	-3.29821700
H	-1.18044800	8.64709300	-4.25669900
H	-0.70337800	10.15117300	-3.46024900
H	-2.21347900	9.35003100	-3.00654200
C	1.05296100	8.19477100	-2.62969900
H	1.52318900	9.16489800	-2.81411500
H	1.13875100	7.60508300	-3.54900300
H	1.63652700	7.69209500	-1.85413300
C	-1.90503400	2.22147100	2.17874800
C	-1.61312700	1.16026700	3.10103000
C	-2.55162500	0.09709200	3.20551600
H	-2.36593400	-0.72384500	3.88071300
C	-0.00192500	3.44048300	3.06692600
H	0.60564700	4.33524100	3.06820600
C	0.30147500	2.37636200	3.94418900
C	-0.43976300	1.20194700	3.92154900
C	-3.28227800	3.15403100	0.18536800
H	-4.29791900	3.02071200	-0.18880400
H	-3.21325600	4.17821500	0.55060100
C	-3.06589900	2.16323800	1.32912100
C	-3.71610700	0.12055600	2.48738200
H	-4.44687300	-0.67402000	2.58965800

C	-3.97721400	1.15160300	1.55830100
O	-5.16232300	1.07857300	0.83510800
C	-6.32000000	1.55780500	1.53623100
H	-6.17267600	2.61407700	1.79053800
H	-6.43962600	1.00097100	2.47522900
C	-7.53062400	1.35361700	0.63716800
H	-7.53694900	0.30356500	0.32585400
H	-7.40227200	1.94910200	-0.27471500
C	-8.88027700	1.69829300	1.29352900
H	-8.96263100	1.11050600	2.21818700
C	-8.99359100	3.18400900	1.66774700
H	-8.23721500	3.48716200	2.39580300
H	-9.97305800	3.40149500	2.10341600
H	-8.87663800	3.81707000	0.78127100
C	-10.03799200	1.28407400	0.37456500
H	-10.00538600	1.84055500	-0.56833300
H	-11.00568800	1.48287600	0.84407900
H	-9.99398400	0.21806700	0.13356900
C	1.97883600	3.70968600	5.07586900
H	1.21196400	4.46220400	5.29346800
H	2.52842100	4.03740300	4.18536900
C	2.90628900	3.56093400	6.27437000
H	2.30147200	3.23506100	7.12733800
H	3.28678900	4.56057100	6.52331200
C	4.09522800	2.59743400	6.09826300
H	3.69295300	1.62542100	5.79326600
C	5.07522100	3.06909800	5.01481500
H	5.49431100	4.05023600	5.26591800
H	4.60026100	3.14908200	4.03363000
H	5.90949100	2.36880300	4.91640100
C	4.82050600	2.40723800	7.43751800
H	5.64641300	1.69672300	7.34165700
H	4.14187600	2.02885100	8.20742700
H	5.23767800	3.35490200	7.79659400
H	0.81182800	0.31808800	5.41149300

CPL Studies

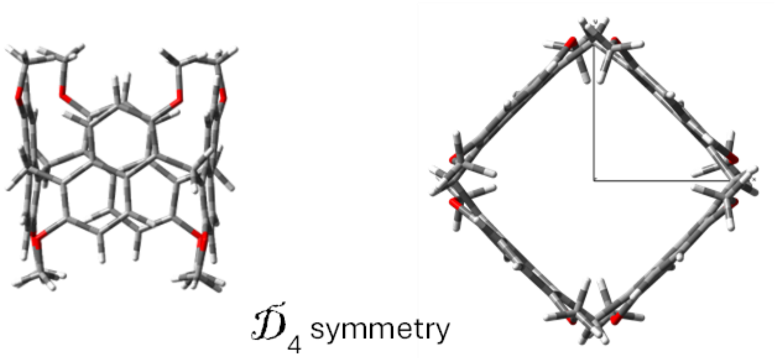

Figure S91: Model molecule: all p*S*-**PrS[4]** *Me*

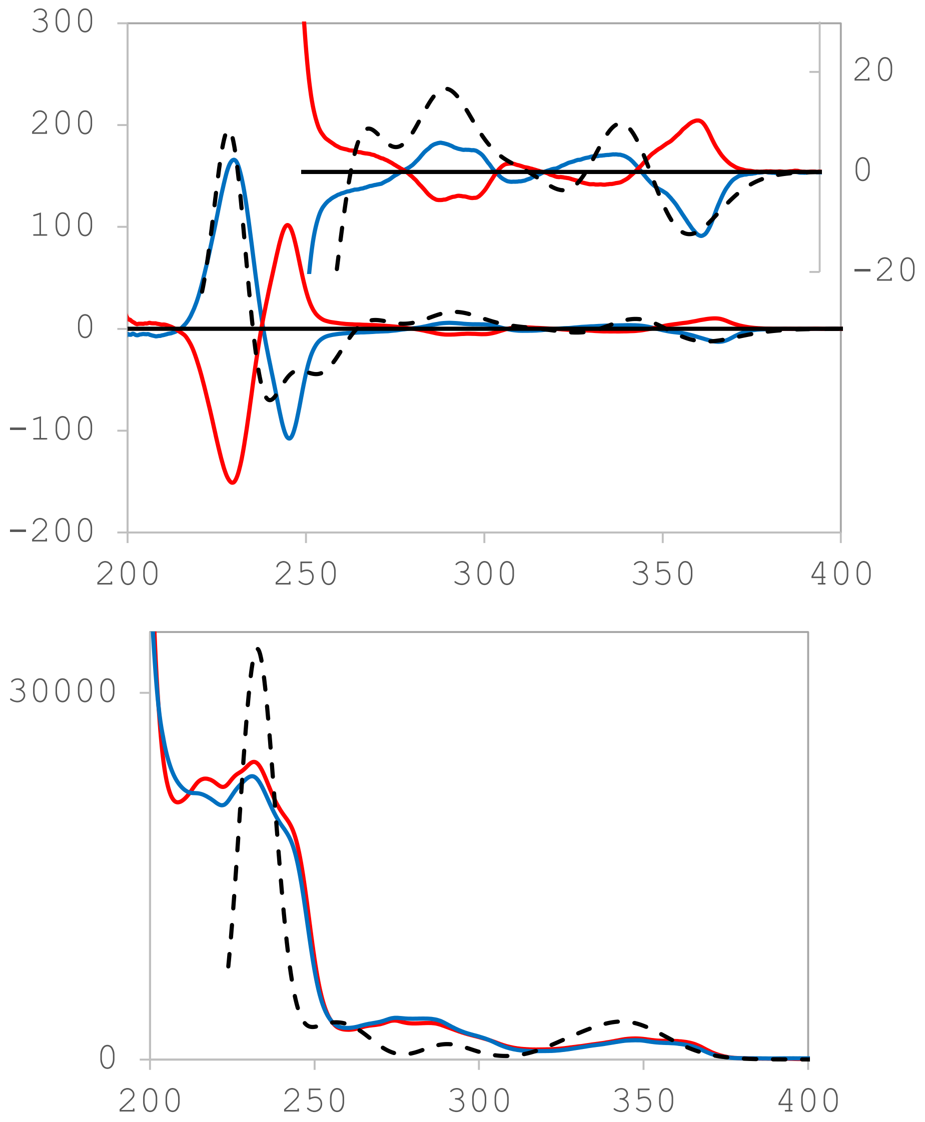

Figure S92: Absorption and CD calculated spectra of the D_4 all *pS*-**PrS[4]*Me*** model molecule superimposed with experimental CD and absorption spectrum of (-)-**PrS[4]*EtCy*** (blu) *and* (+)-**PrS[4] *EtCy*** (red). TD-DFT calculations have been performed at M06/6-311g(d,p) level, gaussian bandwidth 0.2 eV, +4 nm wavelength shift.

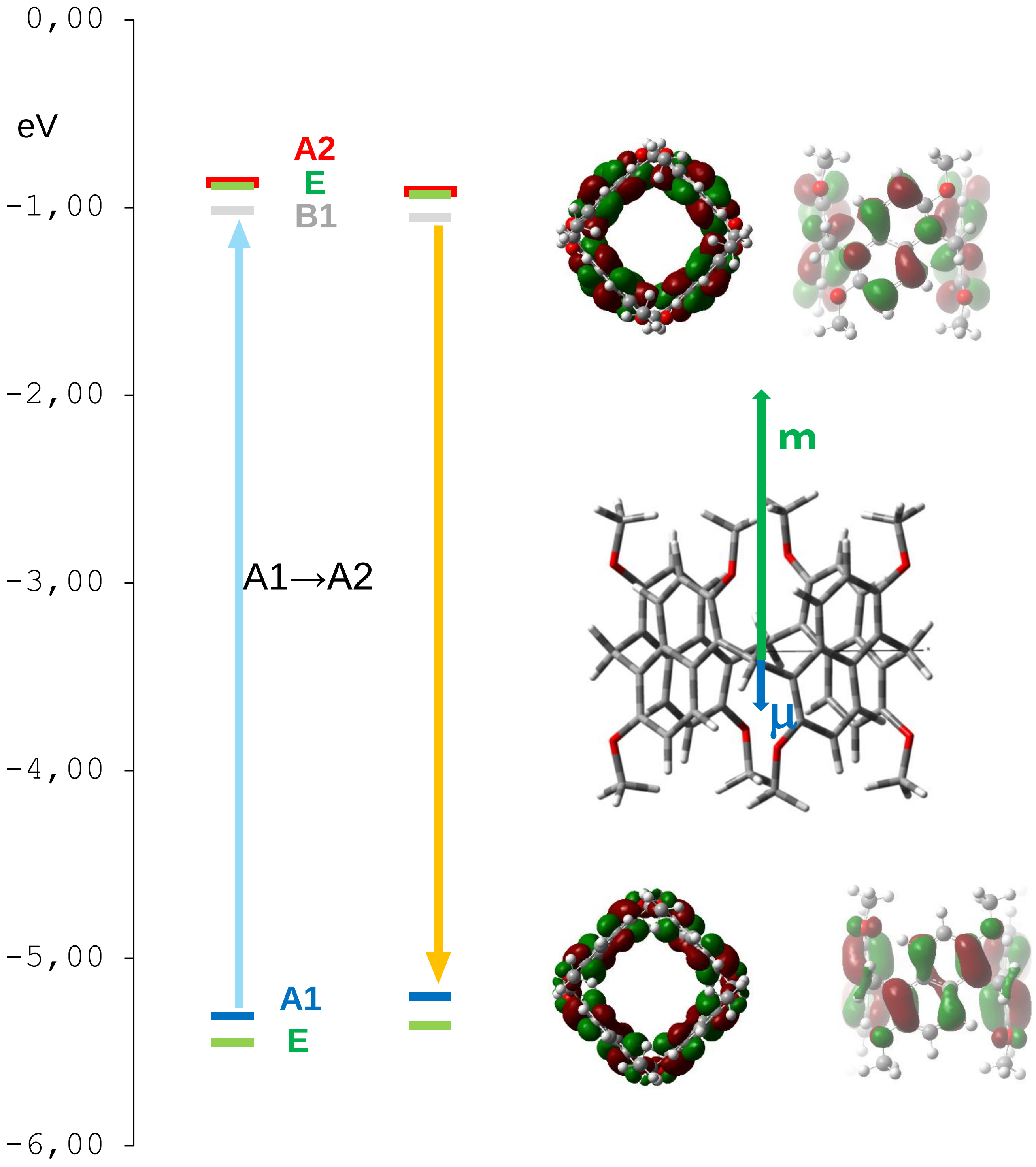

Figure S93: Electronic energy states involved in the first electronic transition for the all-*pS*-**PrS[4]**^{Me} model molecule.

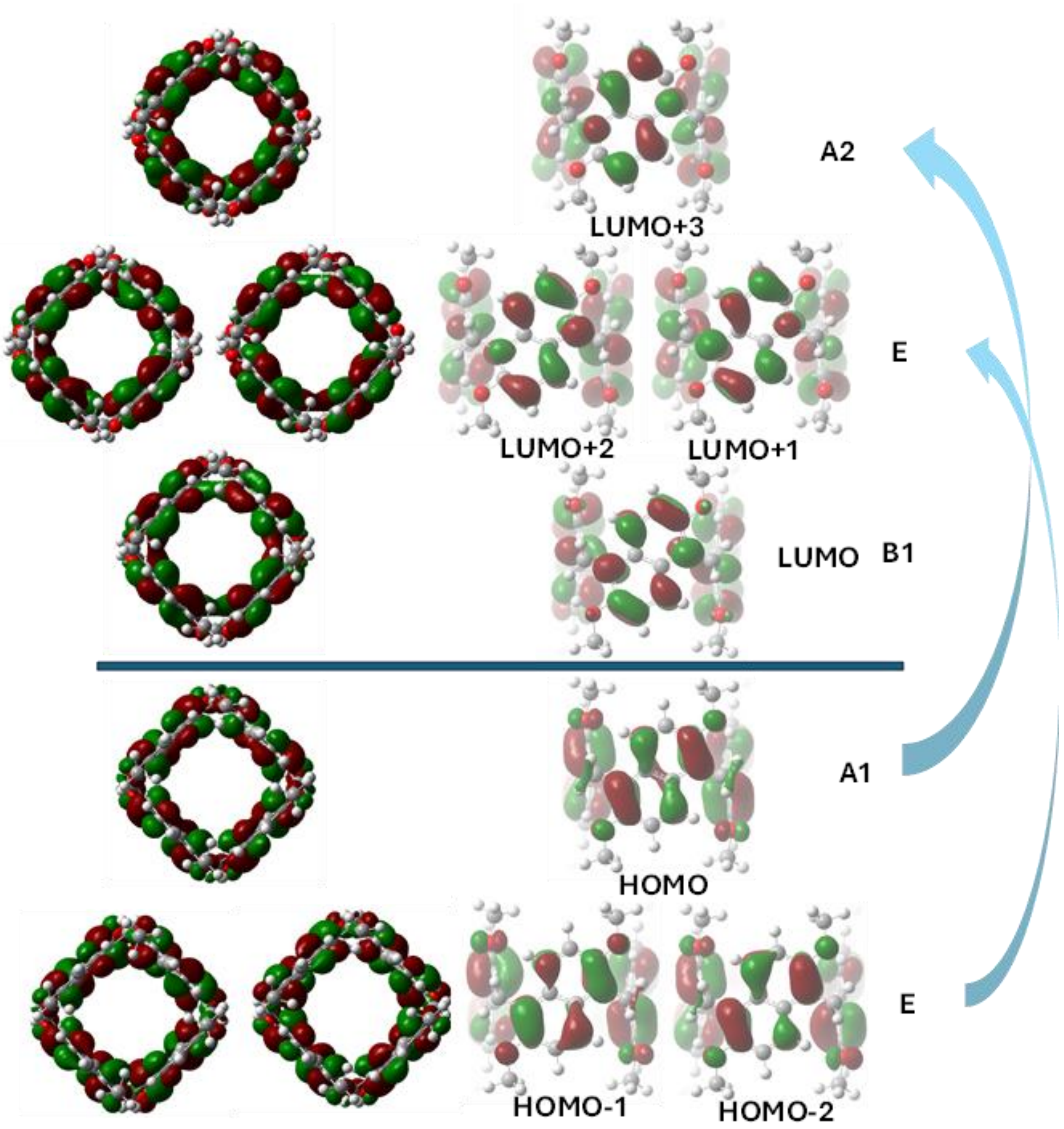

Figure S94: Orbitals involved in the first electronic transition, responsible for the first CD band and the CPL band.

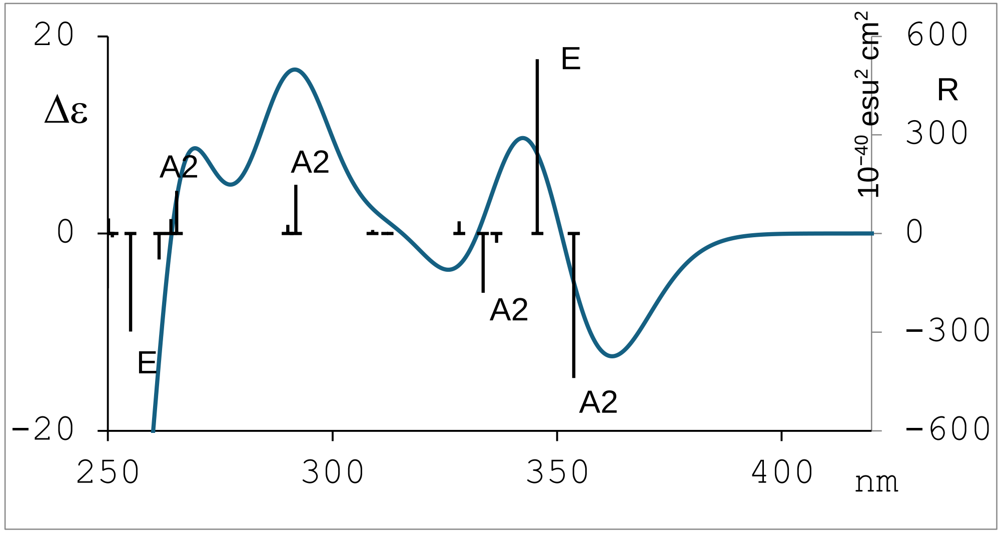

Figure S95: Calculated CD spectrum for *all-pS*-**PrS[4]**^{Me} model compound, sticks represent calculated Rotational Strengths

Table S7: Principal calculated transition characteristics: transition wavelength (nm) and energy (eV), Dipole Strength (D 10^{-40} esu^2 cm^2), Rotational Strengths (R 10^{-40} esu^2 cm^2), electric and magnetic dipole transition moments (atomic units), transition symmetry and orbital components (212=HOMO, 213=LUMO)
The emissive transition characteristics calculated in the emissive excited state are reported in red.

	nm	eV	D	R	µx	µy	µz	mx	my	mz													
1	350	3.55	23724	-465	0.00	0.00	-0.61	0.00	0.00	-3.07	A2	212	->	216	0.54	211	->	214	0.28	210	->	215	-0.28
3	342	3.63	57109	295	0.00	-0.94	0.00	0.00	1.20	0.00	E	212	->	214	0.54	210	->	213	-0.39				
4	342	3.63	57109	295	-0.94	0.00	0.00	1.20	0.00	0.00	E	212	->	215	0.54	211	->	213	0.39				
7	329	3.76	19060	-179	0.00	0.00	-0.54	0.00	0.00	-1.41	A2	209	->	213	0.53	212	->	216	-0.35				
10	324	3.82	24136	21	0.00	-0.61	0.00	0.00	0.13	0.00	E	209	->	215	0.39	211	->	216	0.50	212	->	214	-0.27
11	324	3.82	24136	21	0.61	0.00	0.00	-0.13	0.00	0.00	E	209	->	214	-0.39	210	->	216	0.50	212	->	215	0.27
17	288	4.31	7773	150	0.00	0.00	0.35	0.00	0.00	-1.82	A2	208	->	213	0.43	206	->	214	0.24	207	->	215	-0.24
18	286	4.34	22506	13	-0.59	0.00	0.00	0.10	0.00	0.00	E	206	->	213	0.33	208	->	214	0.29	212	->	218	-0.28
19	286	4.34	22506	13	0.00	0.59	0.00	0.00	-0.10	0.00	E	207	->	213	0.33	208	->	215	-0.29	212	->	219	0.28
21	261	4.74	8225	130	0.00	0.00	0.36	0.00	0.00	-1.55	A2	208	->	213	0.52	205	->	216	0.36				

29	251	4.94	27062	-150	-0.65	0.00	0.00	-0.98	0.00	0.00	E	212	->	218	0.59	209	->	219	-0.30				
30	251	4.94	27062	-150	0.00	-0.65	0.00	0.00	-0.98	0.00	E	212	->	219	0.59	209	->	218	-0.30				
47	236	5.25	18521	124	0.00	0.00	-0.54	0.00	0.00	0.97	A2	209	->	217	0.38	210	->	218	0.37	211	->	219	-0.37
48	235	5.29	17207	-398	-0.52	0.00	0.00	-3.17	0.00	0.00	E	209	->	219	0.42	211	->	221	-0.24	204	->	214	0.24
49	235	5.29	17207	-398	0.00	-0.52	0.00	0.00	-3.17	0.00	E	209	->	218	0.42	210	->	221	-0.24	204	->	215	-0.24
1	364	3.4	21434	-432	0.00	0.00	-0.58	0.00	0.00	-2.99	A2	212	->	216	0.57	210	->	215	-0.27	211	->	214	0.27

Collision-induced dissociation (CID) experiment and chiral selectivity

To investigate the non-covalent interaction between enantiopure **PrS[4]**^{iPe} and chiral guests **(*S*)-6**$^{2+}$, **(*S*)-7**$^{+}$, **(*S*)-8**$^{+}$, **(*S*)-9**$^{+}$, host and guest ware mixed in 1:1 ratio (1×10^{-4} mol/L in dichloromethane and 10% of methanol).
All spectra were recorded on a Bruker Solaris XR Fourier transform ion cyclotron resonance mass spectrometer equipped with a 7T refrigerated actively shielded superconducting magnet.
An electrospray ionization (ESI) source was used to generate charged ions by spraying the sample solution at a flow rate of 100 $\mu L \cdot h^{-1}$. The ionization conditions were as follows: capillary voltage: –4500 V; skimmer voltage: 50 V; drying gas temperature: 100 °C, with a flow rate of 4.0 $L \cdot min^{-1}$. For each sample, 50 scans were collected with a digitized resolution of 16 Mb. Nitrogen was used as collision gas.

The center-of-mass collision energy (ECM) is the maximum amount of kinetic energy that can be converted into internal energy upon collisional activation under single collision conditions. ECM is given by:

$$E_{CM} = E_{LAB}[m_g/(m_g+m_p)]$$

where m_g is the mass of the stationary target gas, m_p is the mass of the projectile ion and E_{LAB} is the ion kinetic energy in the laboratory frame of reference.

Table S8: Intensity of monoisotopic peak of complex and ***pR*-PrS[4]**^{iPe}

	**(*S*)-6^{2+}**	**(*S*)-7^{+}**	**(*S*)-8^{+}**	**(*S*)-9^{+}**
**G^{+}@*pR*-PrS[4]^{iPe}**	515539616	671051584	166123856	236452944
***pR*- PrS[4]^{iPe}**	150658016	193572816	16703448	135482000
Normalized intensity	**3.41**	**3.46**	**10.54**	**1.74**

Table S9: Intensity of monoisotopic peak of complex and ***pS*-PrS[4]**^{iPe}

	**(*S*)-6^{2+}**	**(*S*)-7^{+}**	**(*S*)-8^{+}**	**(*S*)-9^{+}**
**G^{+}@*pS*-PrS[4]^{iPe}**	342146688	366468896	99329312	76797872
***pS*-PrS[4]^{iPe}**	49284568	86649976	10082687	61849028
Normalized intensity	**6.94**	**4.23**	**9.85**	**1.24**

Table S10: Chiral selectivity values

	**(*S*)-6^{2+}**	**(*S*)-7^{+}**	**(*S*)-8^{+}**	**(*S*)-9^{+}**
Chiral Selectivity pS/pR	6.94/3.41= **2.01**	4.23/3.46= **1.22**	9.85/10.54= **0.94**	1.24/1.74= **0.70**

Reference

[1] (a) Hickey, N.; Geremia, S.; De Rosa, M.; Spinella, A.; Soriente, A.; Spinella, A.; Neri, P.; Gaeta, C. Prismarenes: A New Class of Macrocyclic Hosts Obtained by Templation in a Thermodynamically Controlled Synthesis. *J. Am. Chem. Soc*., **2020**, *142*, 1752–1756. (b) Della Sala, P.; Del Regno, R.; Di Marino, L.; Calabrese, C.; Palo, C.; Geremia, S.; Talotta, C.; Geremia, S.; Hick-ey, N.; Capobianco, A.; Neri, P.; Gaeta, C. An intramolecularly self-templated synthesis of macrocycles: self-filling effects on the formation of prismarenes. *Chem. Sci*., **2021**, *12*, 9952−9961. (c) Hirose, K. in "Analytical Methods in Supramolecular Chemistry" (Ed.: Schalley, C. A.) Wiley-VCH, Weinheim, 2007, pp. 17–54

[2]Gaussian 16, Revision C.01, Frisch, M. J.; Trucks, G. W.; Schlegel, H. B.; Scuseria, G. E.; Robb, M. A.; Cheeseman, J. R.; Scalmani, G.; Barone, V.; Petersson, G. A.; Nakatsuji, H.; Li, X.; Caricato, M.; Marenich, A. V.; Bloino, J.; Janesko, B. G.; Gomperts, R.; Mennucci, B.; Hratchian, H. P.; Ortiz, J. V.; Izmaylov, A. F.; Sonnenberg, J. L.; Williams-Young, D.; Ding, F.; Lipparini, F.; Egidi, F.; Goings, J.; Peng, B.; Petrone, A.; Henderson, T.; Ranasinghe, D.; Zakrzewski, V. G.; Gao, J.; Rega, N.; Zheng, G.; Liang, W.; Hada, M.; Ehara, M.; Toyota, K.; Fukuda, R.; Hasegawa, J.; Ishida, M.; Nakajima, T.; Honda, Y.; Kitao, O.; Nakai, H.; Vreven, T.; Throssell, K.; Montgomery, J. A., Jr.; Peralta, J. E.; Ogliaro, F.; Bearpark, M. J.; Heyd, J. J.; Brothers, E. N.; Kudin, K. N.; Staroverov, V. N.; Keith, T. A.; Kobayashi, R.; Normand, J.; Raghavachari, K.; Rendell, A. P.; Burant, J. C.; Iyengar, S. S.; Tomasi, J.; Cossi, M.; Millam, J. M.; Klene, M.; Adamo, C.; Cammi, R.; Ochterski, J. W.; Martin, R. L.; Morokuma, K.; Farkas, O.; Foresman, J. B.; Fox, D. J. Gaussian, Inc., Wallingford CT, 2016.

[3]Krieger, E.; Vriend, G. YASARA View-Molecular Graphics for All Devices—from Smartphones to Workstations. *Bioinformatics* **2014**, *30* (20), 2981–2982.

[4]NBO Version 3.1, Glendening, E. D.; Reed, A. E.; Carpenter, J. E.; Weinhold., F..

[5]Lu, T.; Chen, F. Multiwfn: A Multifunctional Wavefunction Analyzer. *J. Comput. Chem.* **2012**, *33*, 580–592.
